# Supplementary material for: Individualized discovery of rare cancer drivers in global network context
Source: eLife. 2022 May 20;11:e74010. doi: 10.7554/eLife.74010 (PMC9159755; doi:10.7554/eLife.74010)
Supplement: Figure 7—source data 1. [file elife-74010-fig7-data1.zip › Source data.docx]

| **Cohort** | **Patient** | **Survival**  **event** | **Survival**  **time** | **NEA scores** | |
| --- | --- | --- | --- | --- | --- |
|  |  |  |  | **DGS** | **gene expression based AGS** |
| MB | SJMB037 | No | 26 months | 5.44 | -2.67 |
| MB | SJMB016 | No | 59 months | -1.51 | 2.78 |
| LUAD | TCGA-64-5775 | Yes | 62 days | 4.57 | 11.22 |
| LUAD | TCGA-75-7025 | No | 3305 days | 7.84 | -2.28 |

Figure 7-source data
