# Supplementary material for: Individualized discovery of rare cancer drivers in global network context
Source: eLife. 2022 May 20;11:e74010. doi: 10.7554/eLife.74010 (PMC9159755; doi:10.7554/eLife.74010)
Supplement: Supplementary file 1. — Observed vs. predicted values of anchor.summary values represents performance of created models on continuous scale. In absence of a strict cut-off, performance was measured as a correlation between anchor.summary observed for each gene in the given cohort versus the a value predicted by the multiple regression model. In heatmaps, values next to gene names indicate number of samples with mutations in the given gene. [file elife-74010-supp1.zip › SupplementaryFigure1.Models/SupplementaryFigure1.Models.pdf]

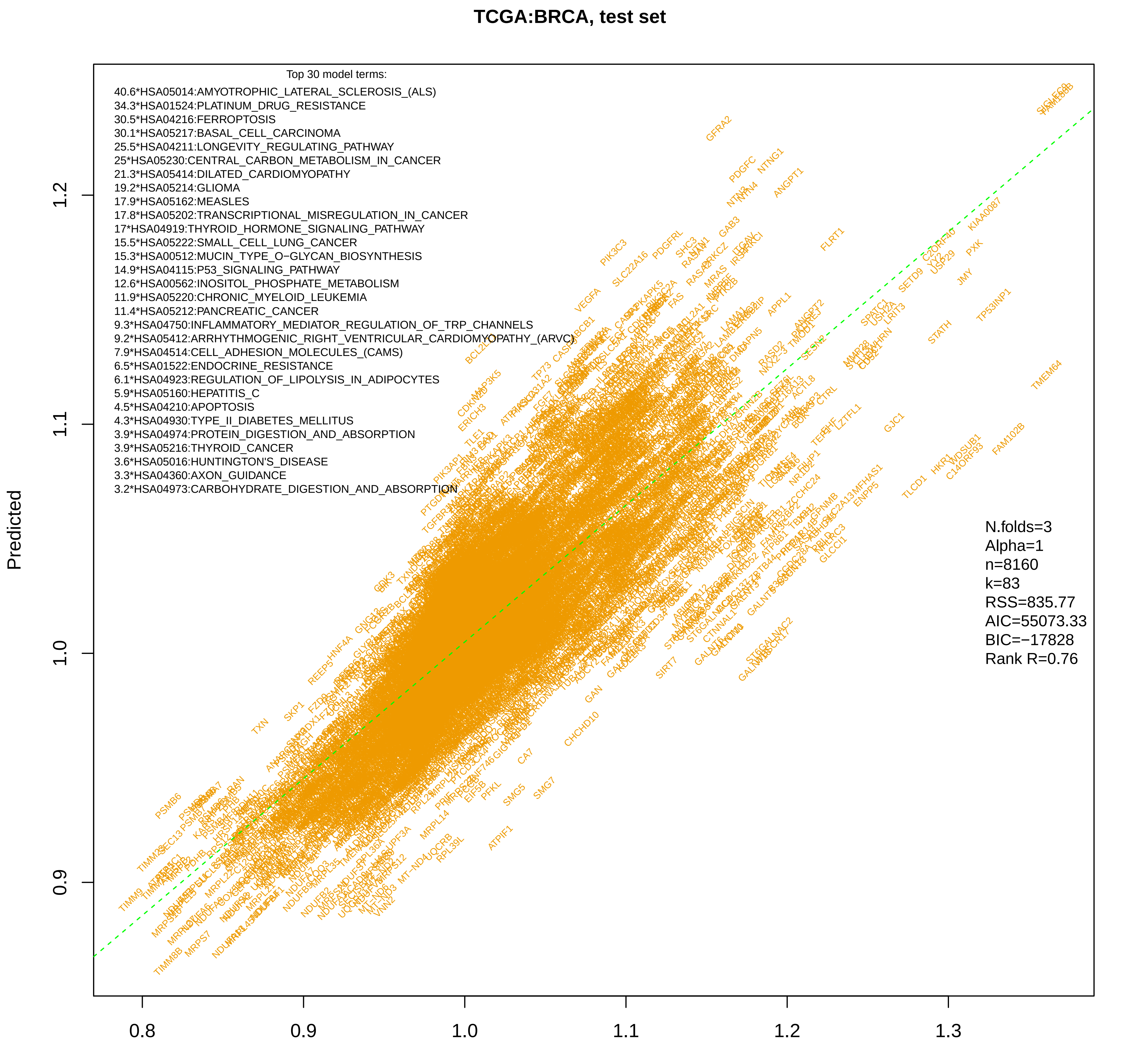


**Supplementary Figure 1A**

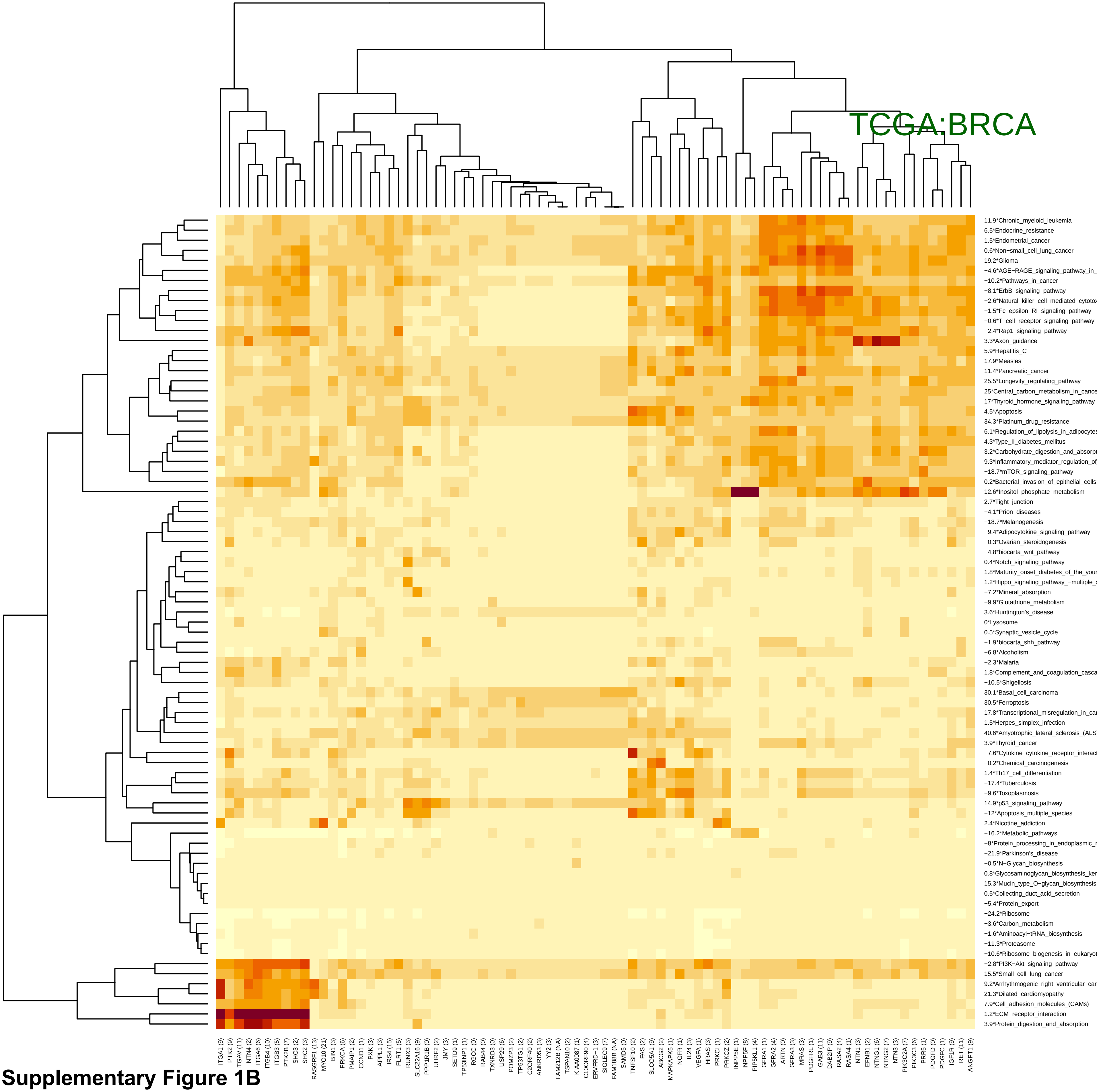


**Supplementary Figure 1B**

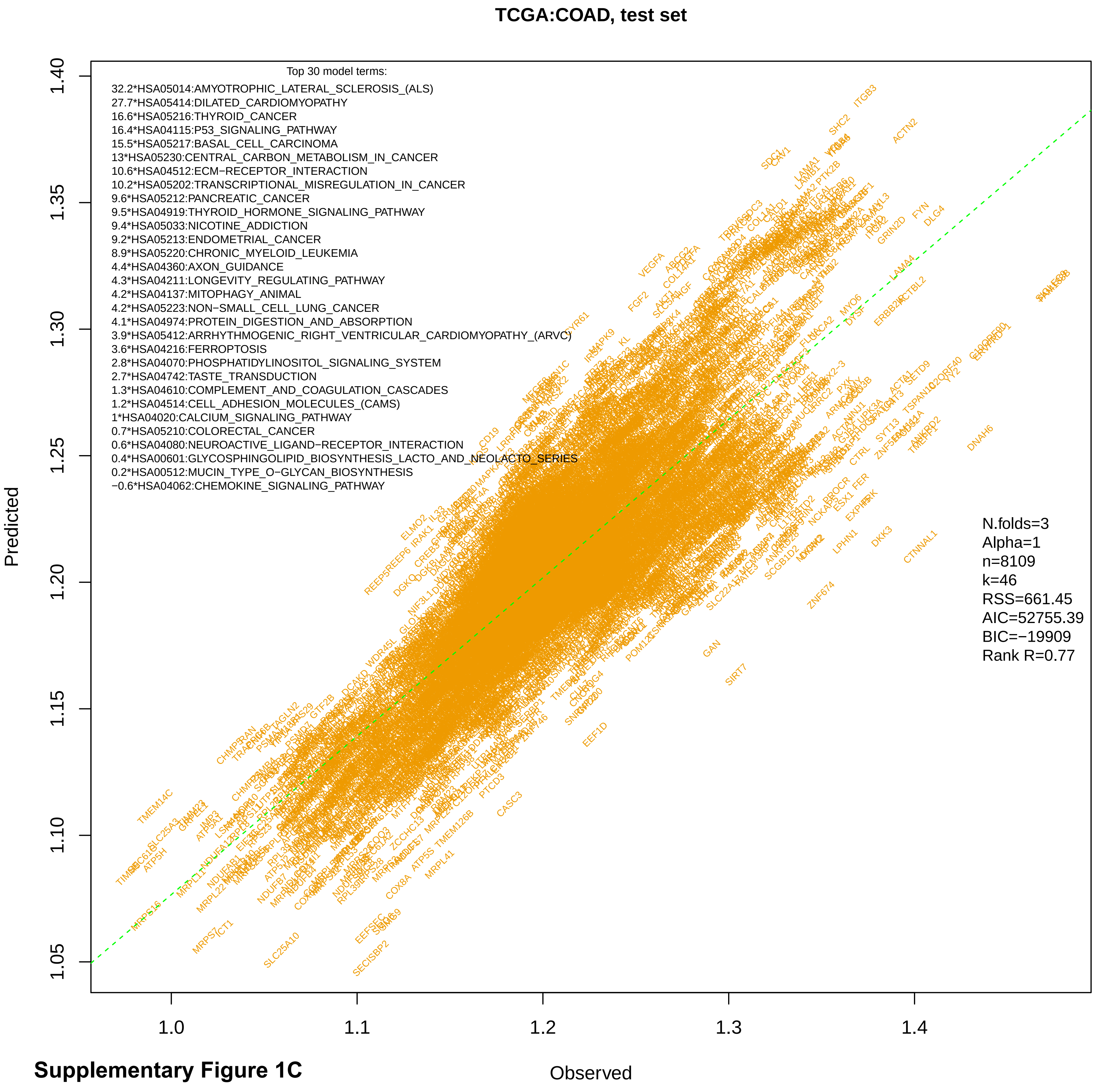


**Supplementary Figure 1C**

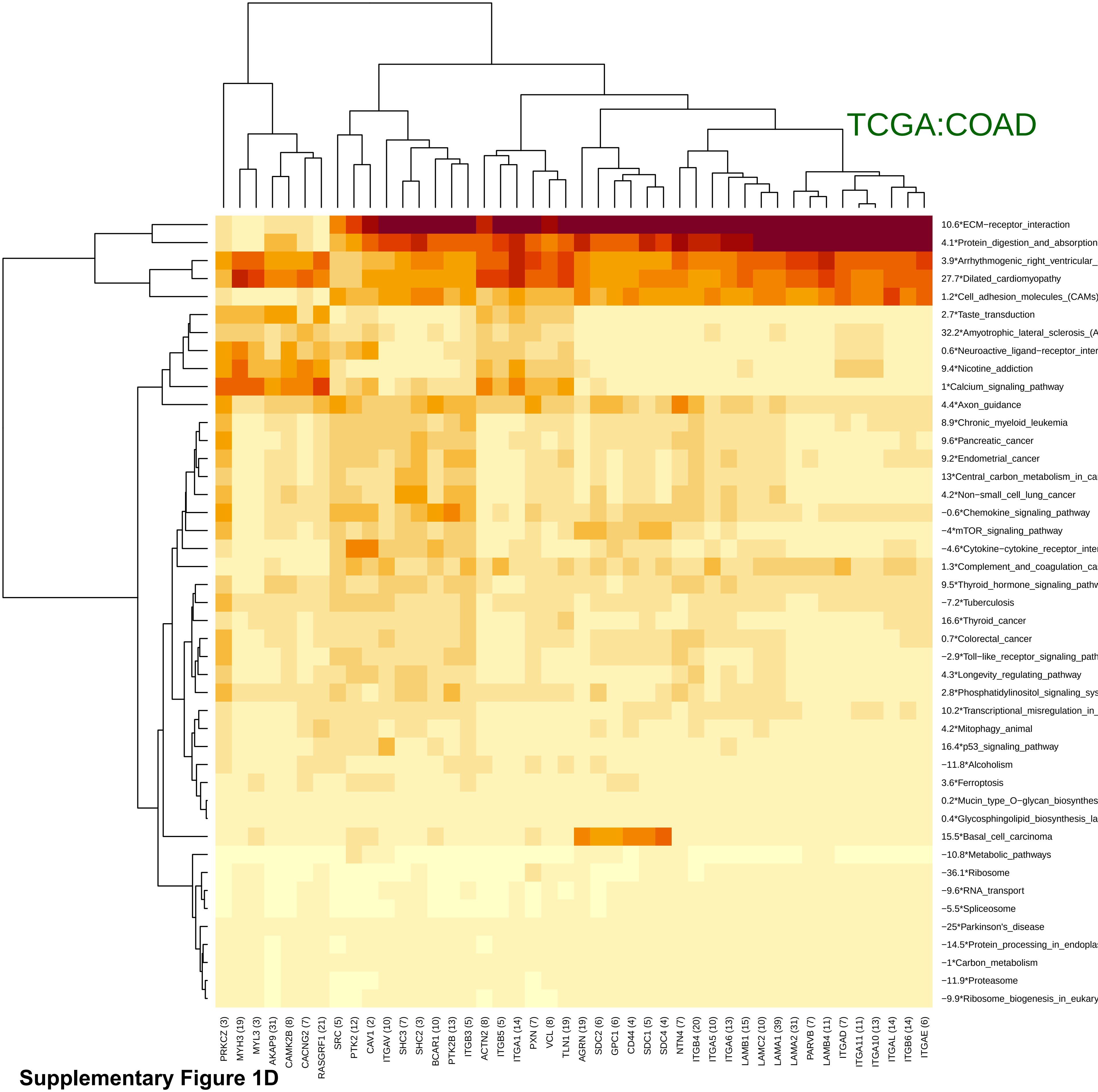


**Supplementary Figure 1D**

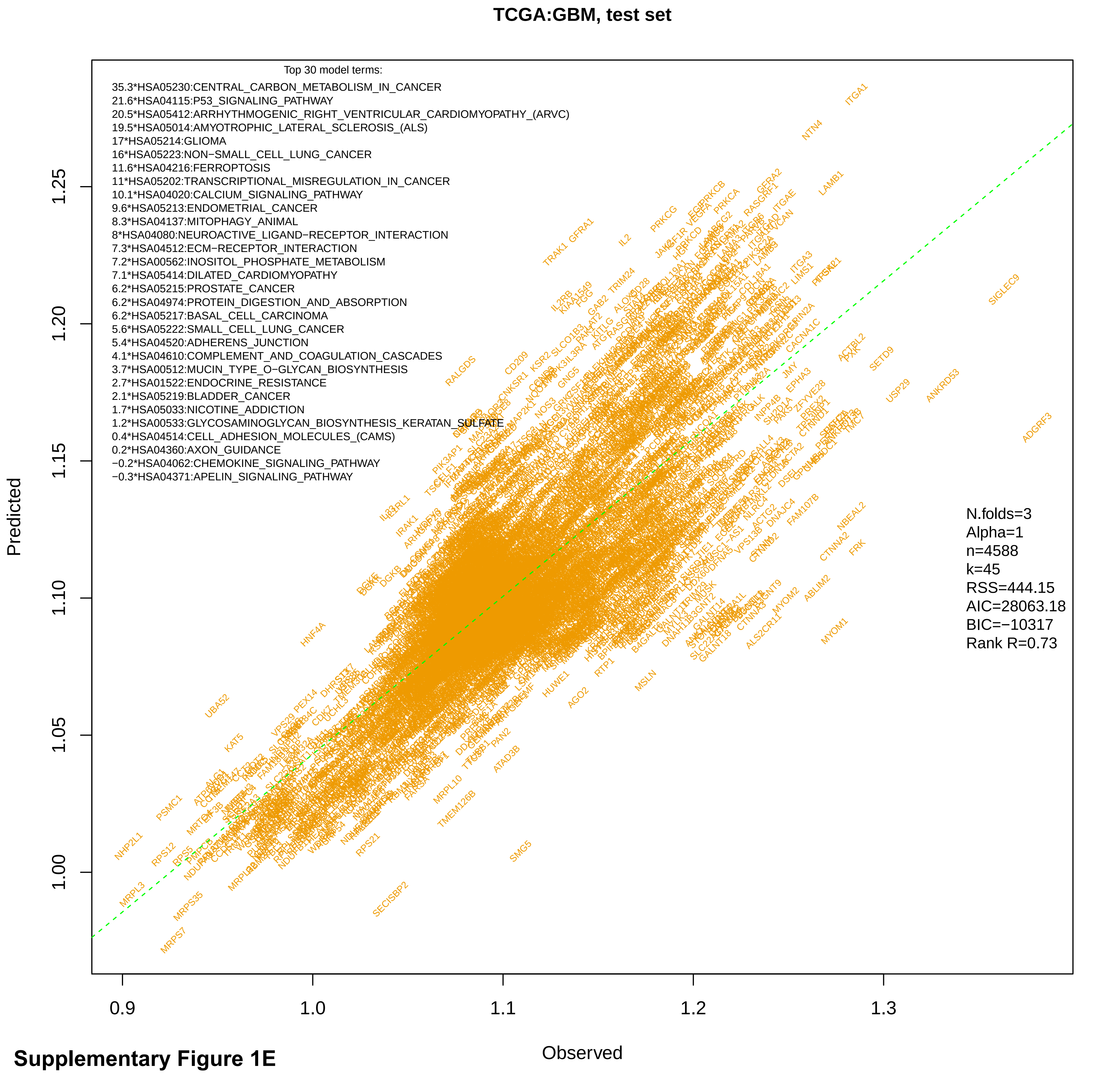


**Supplementary Figure 1E**

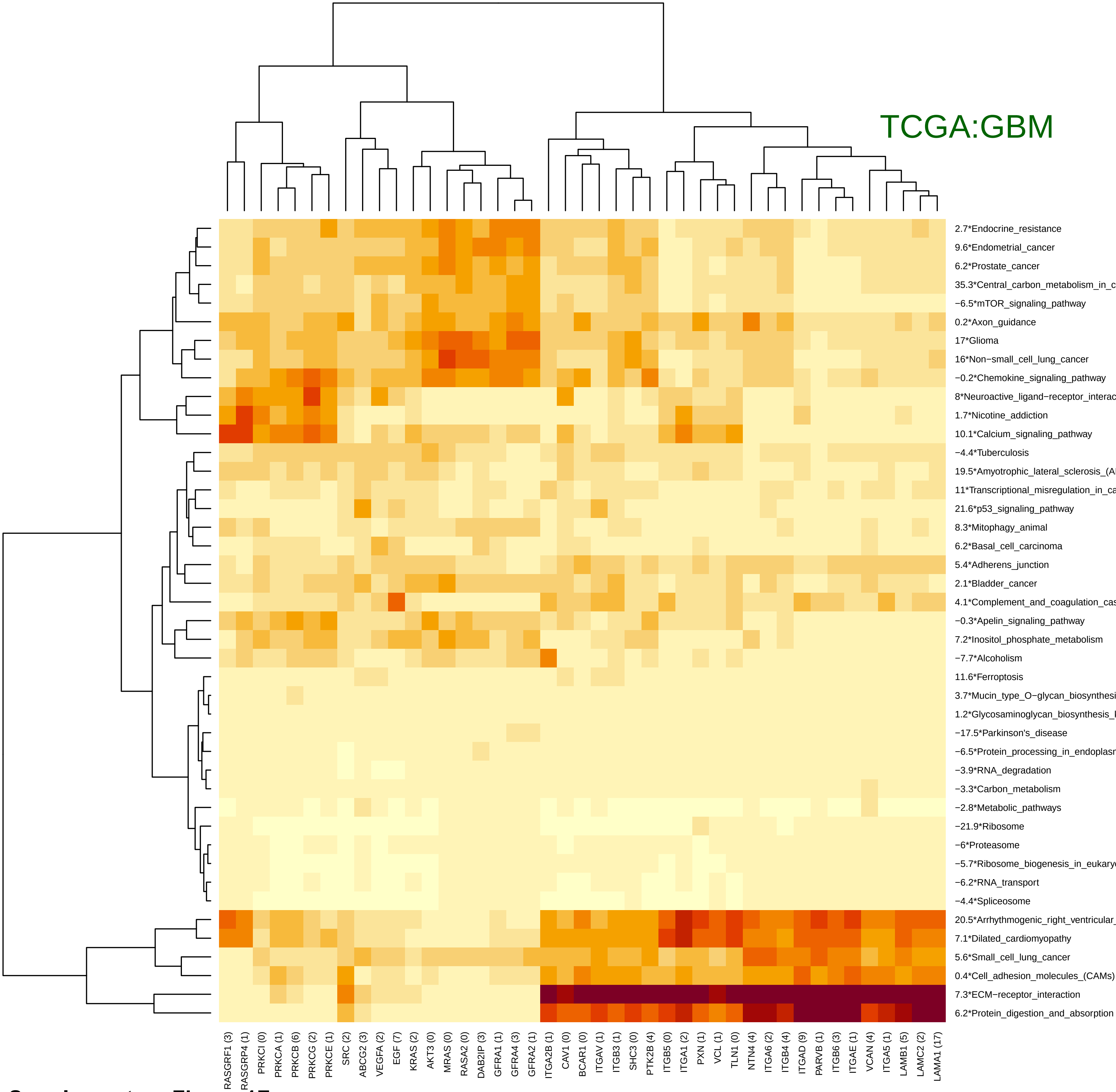


**Supplementary Figure 1F**

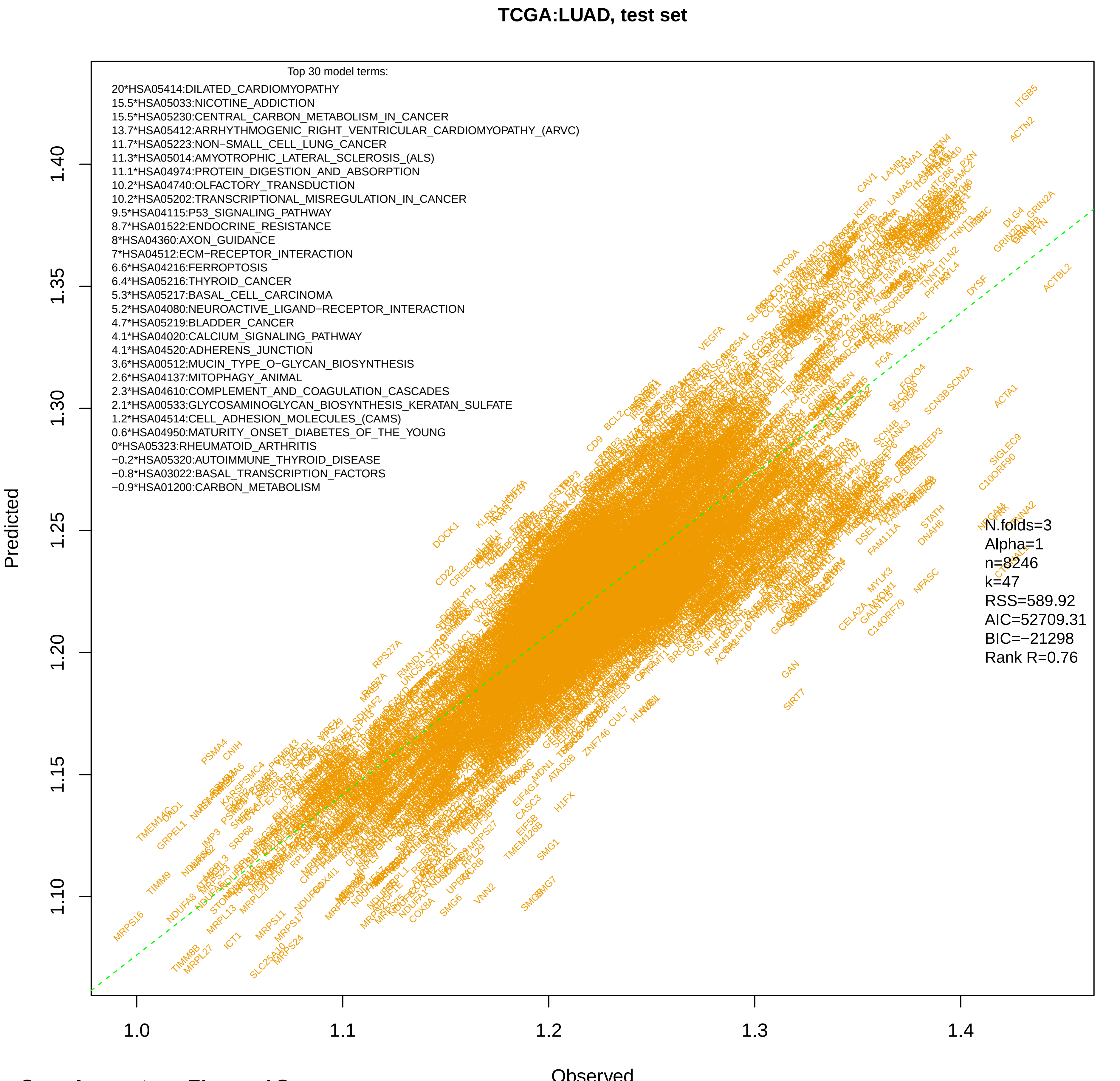


**Supplementary Figure 1G**

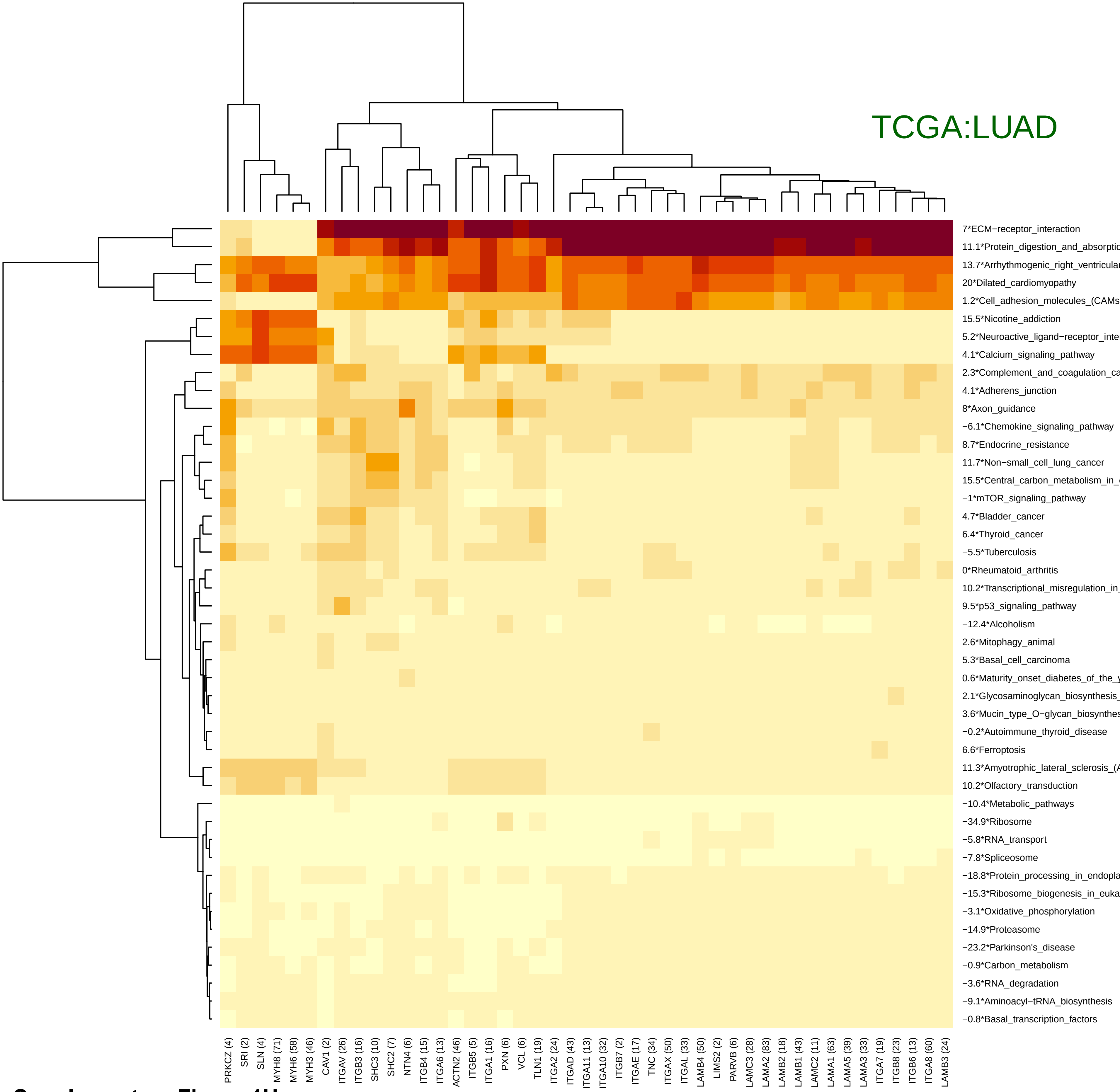


**Supplementary Figure 1H**

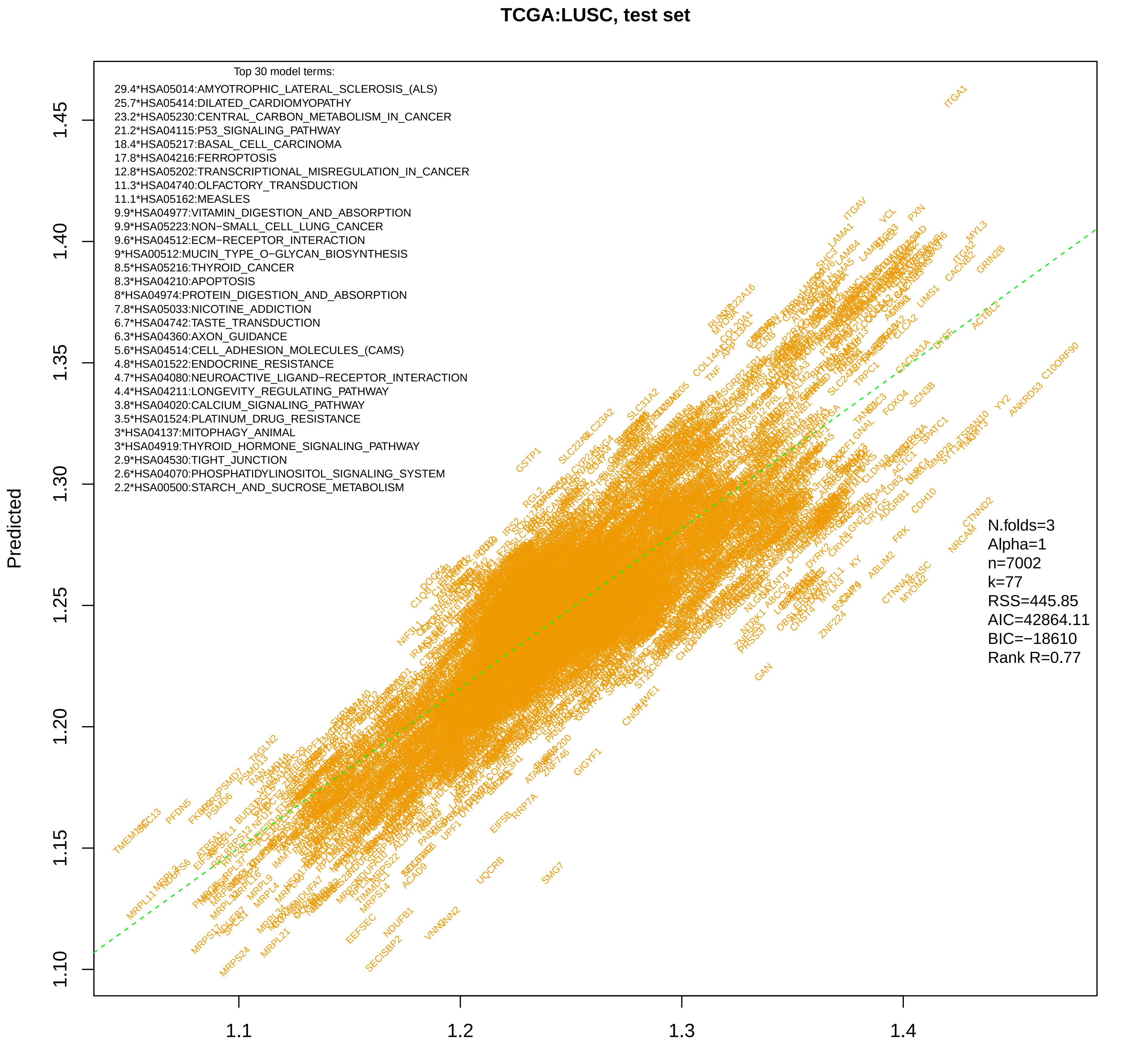


**Supplementary Figure 1I**

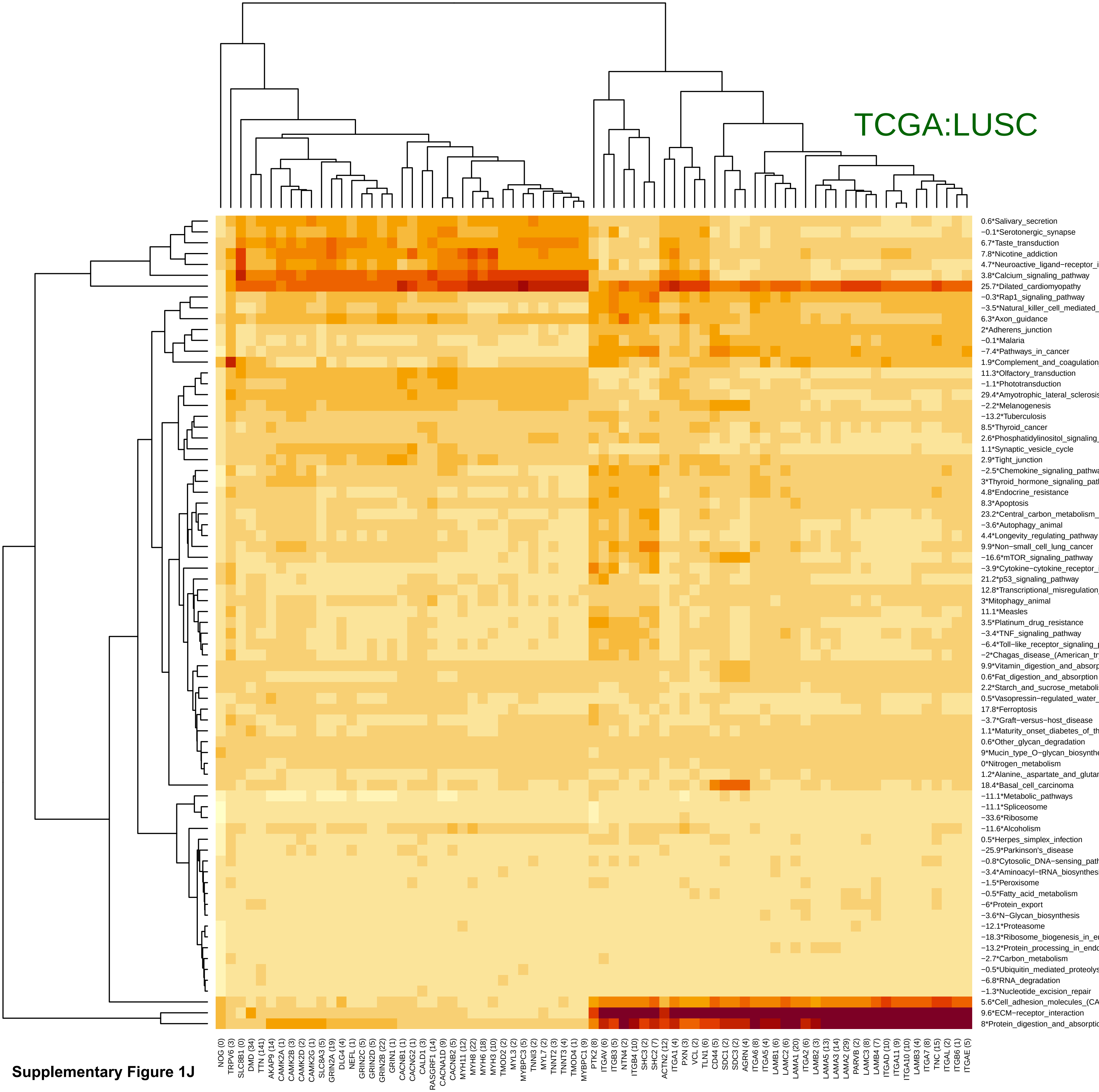


**Supplementary Figure 1J**

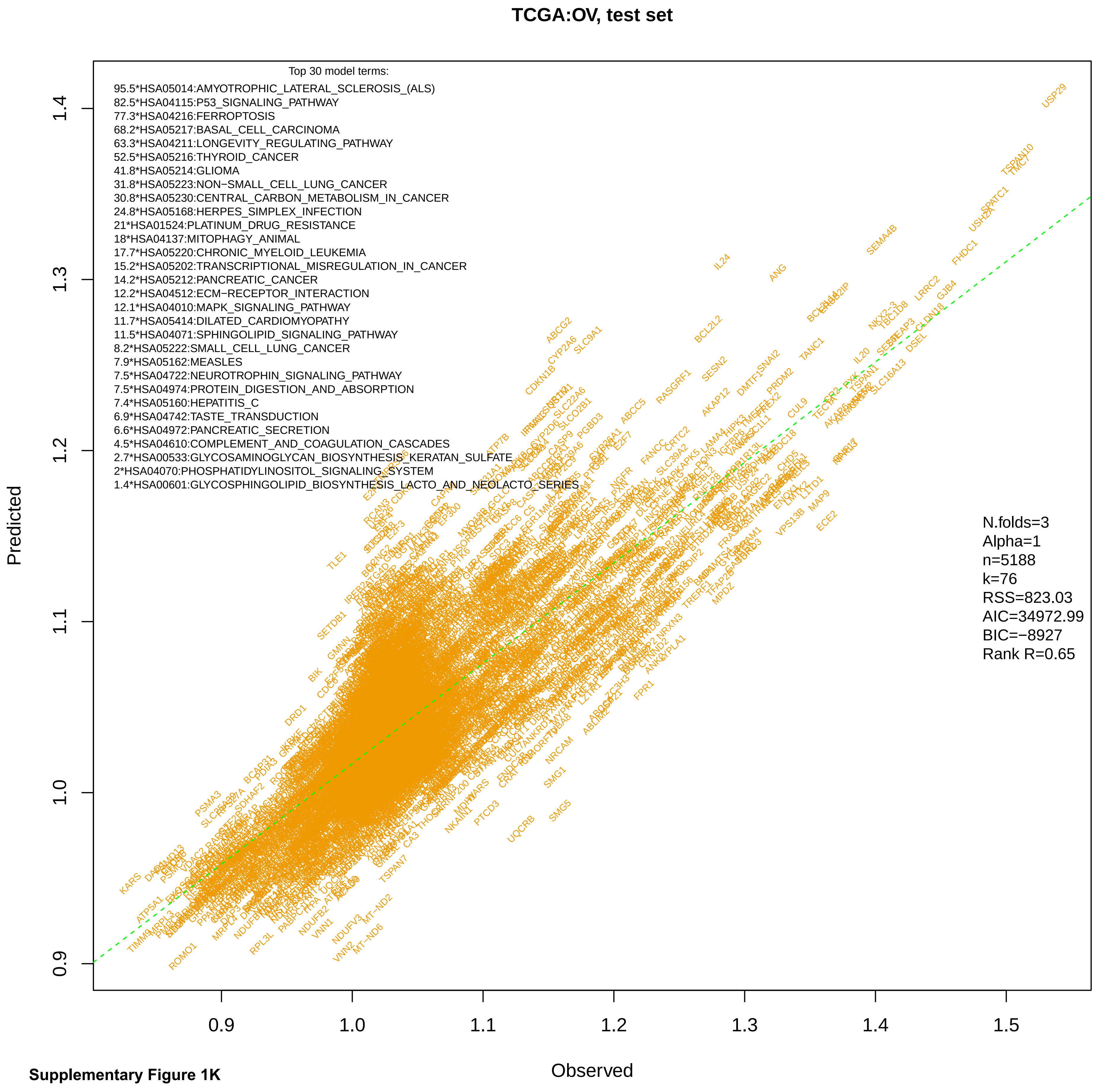


**Supplementary Figure 1K**

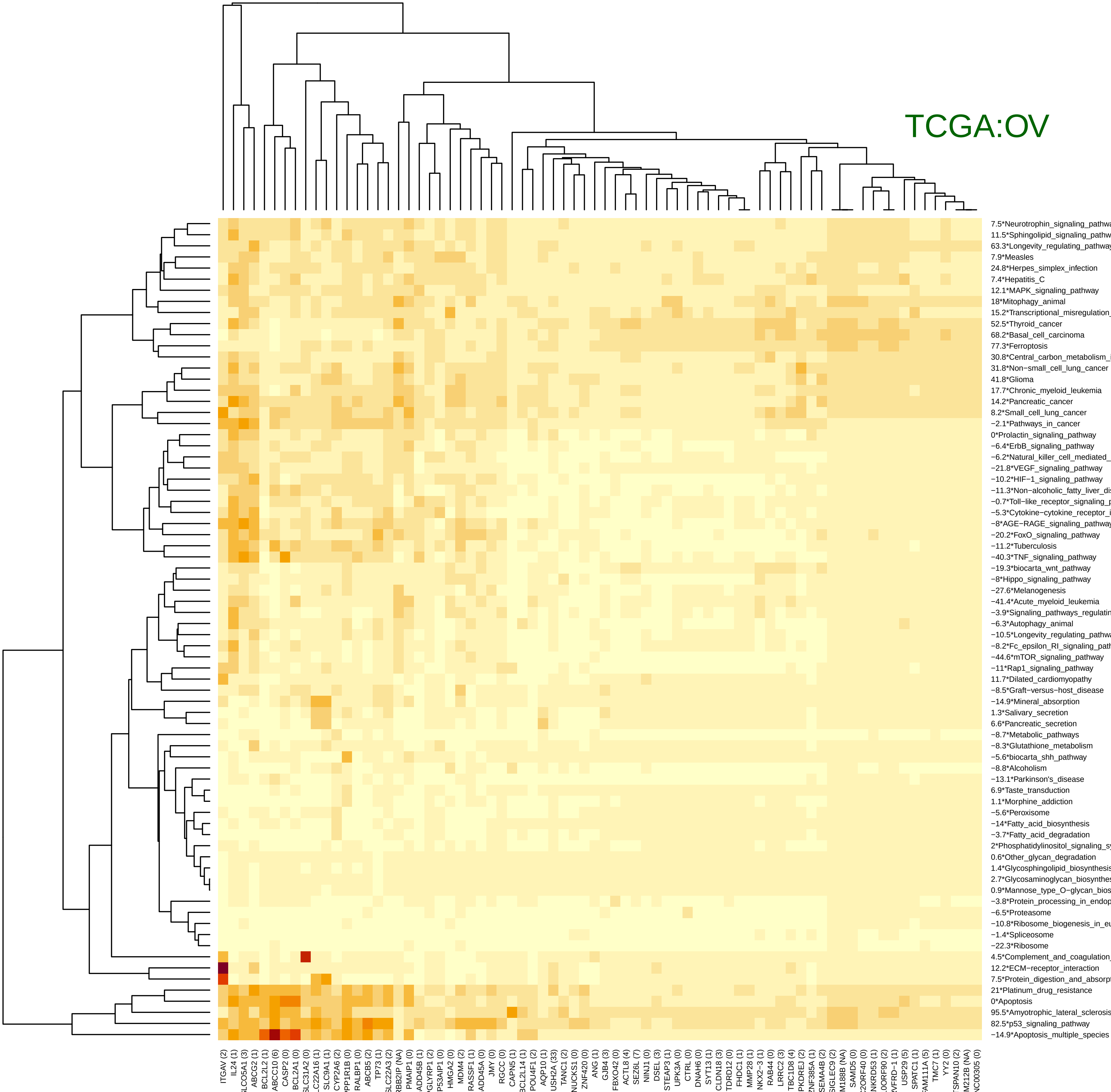


**Supplementary Figure 1L**

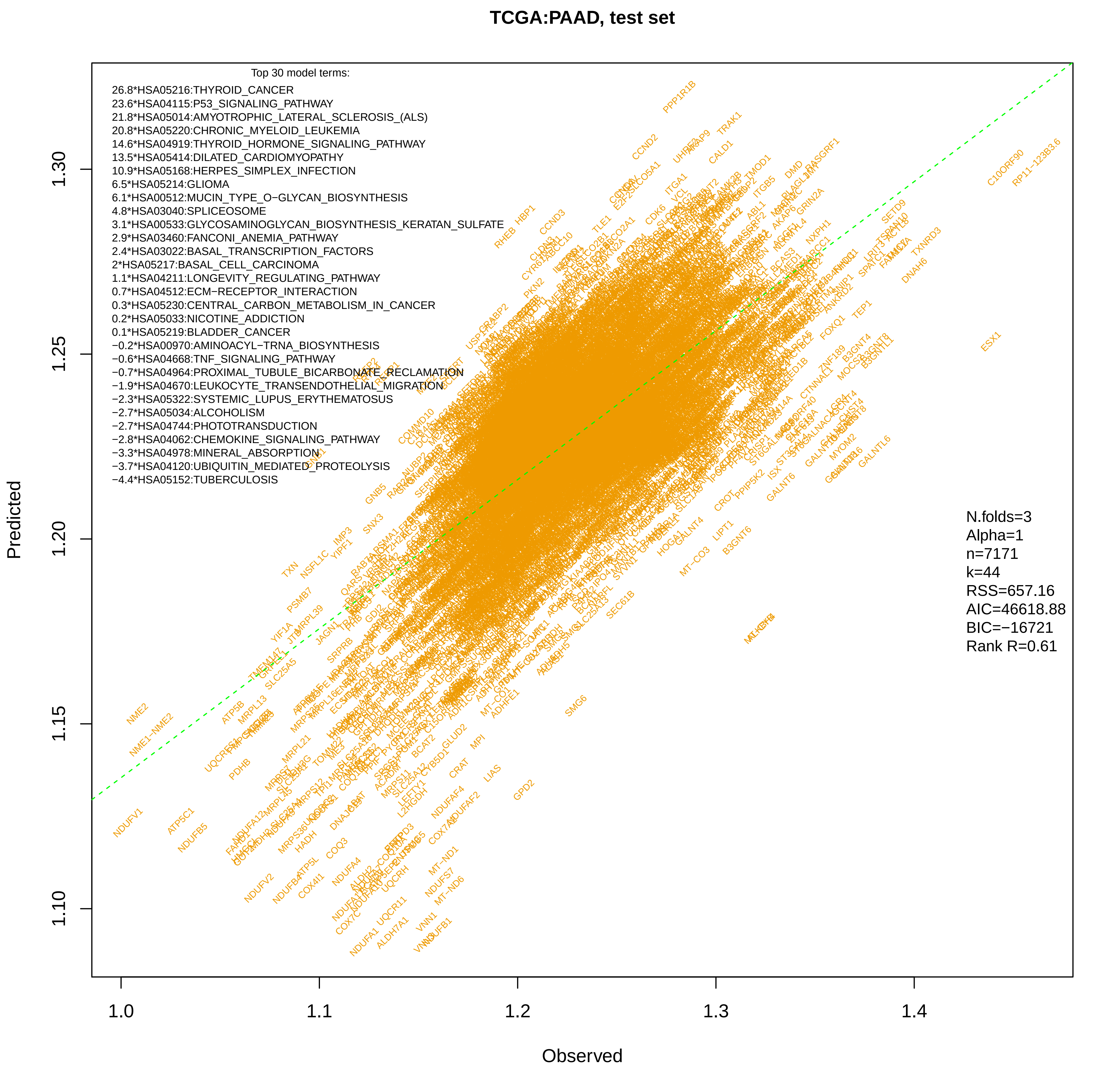


**Supplementary Figure 1M**

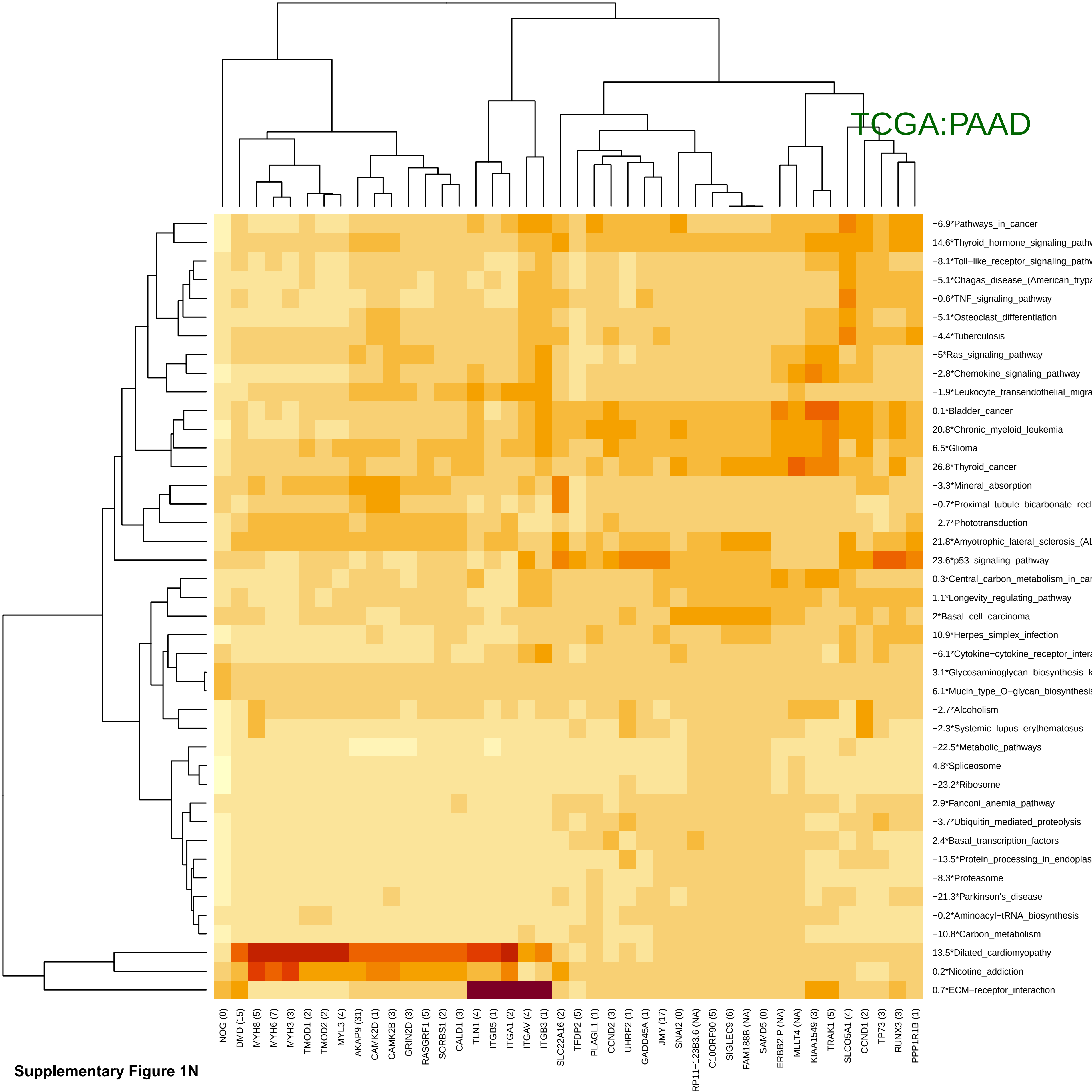


**Supplementary Figure 1N**

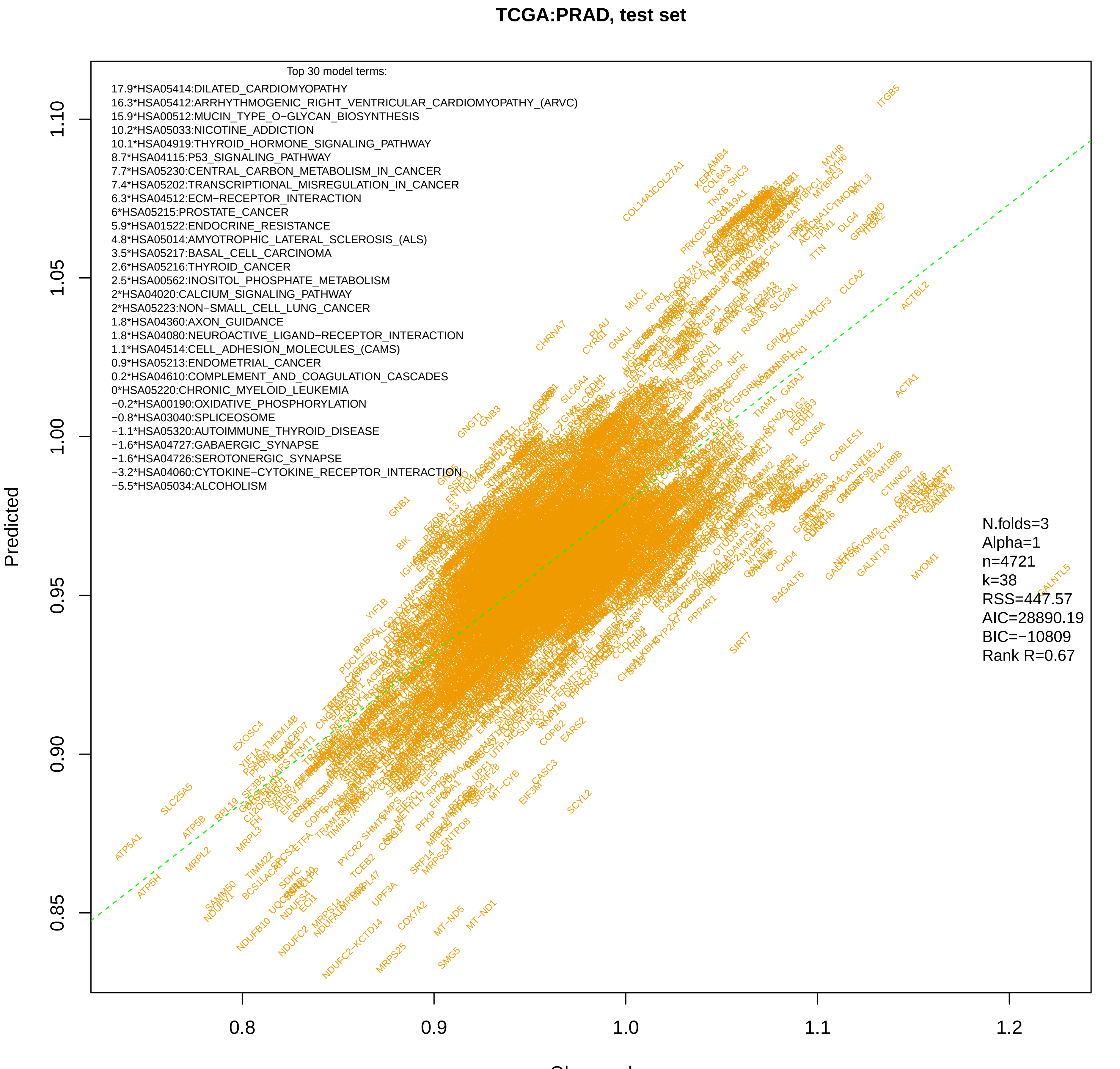


**Supplementary Figure 1O**

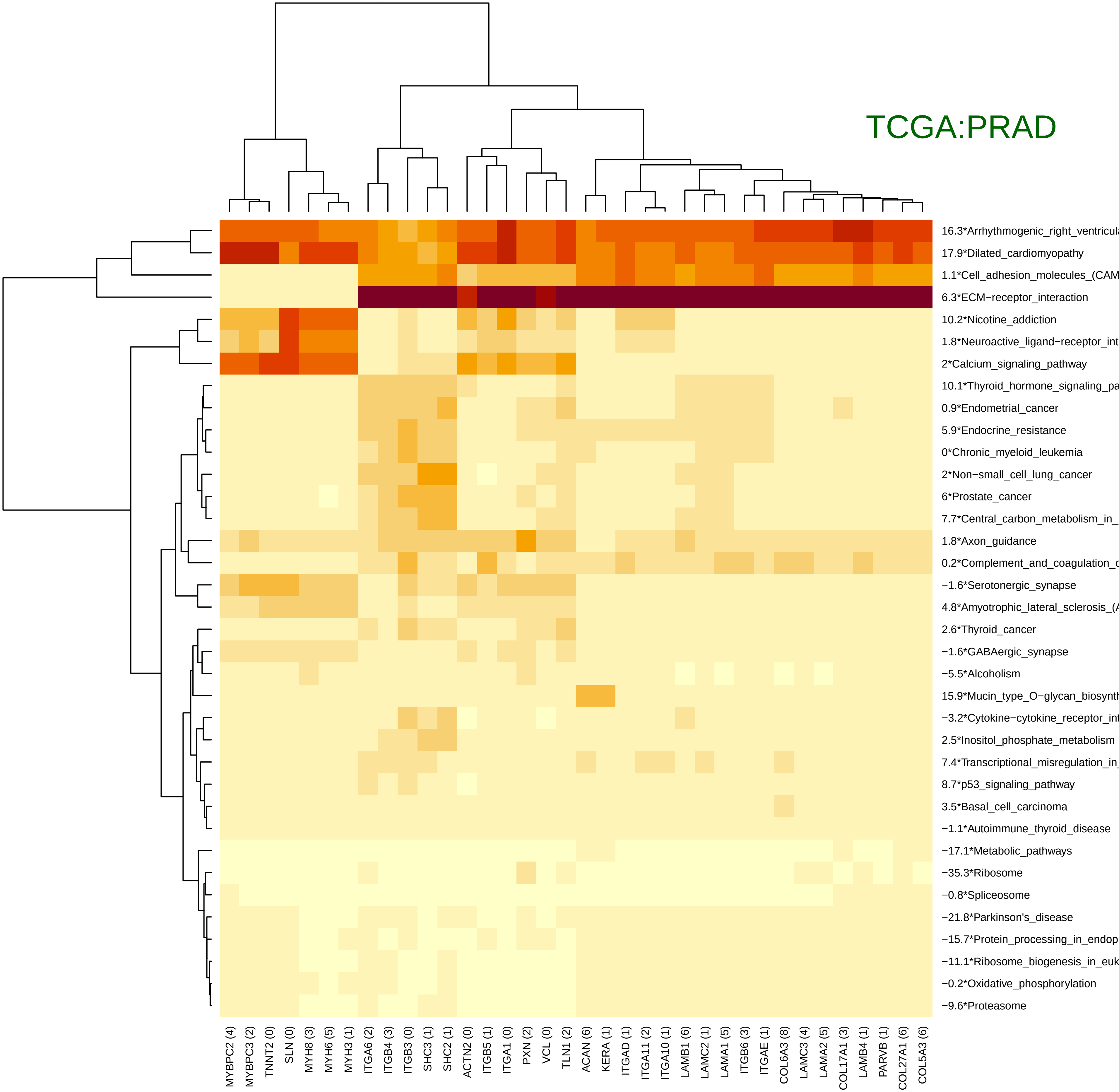


**Supplementary Figure 1P**

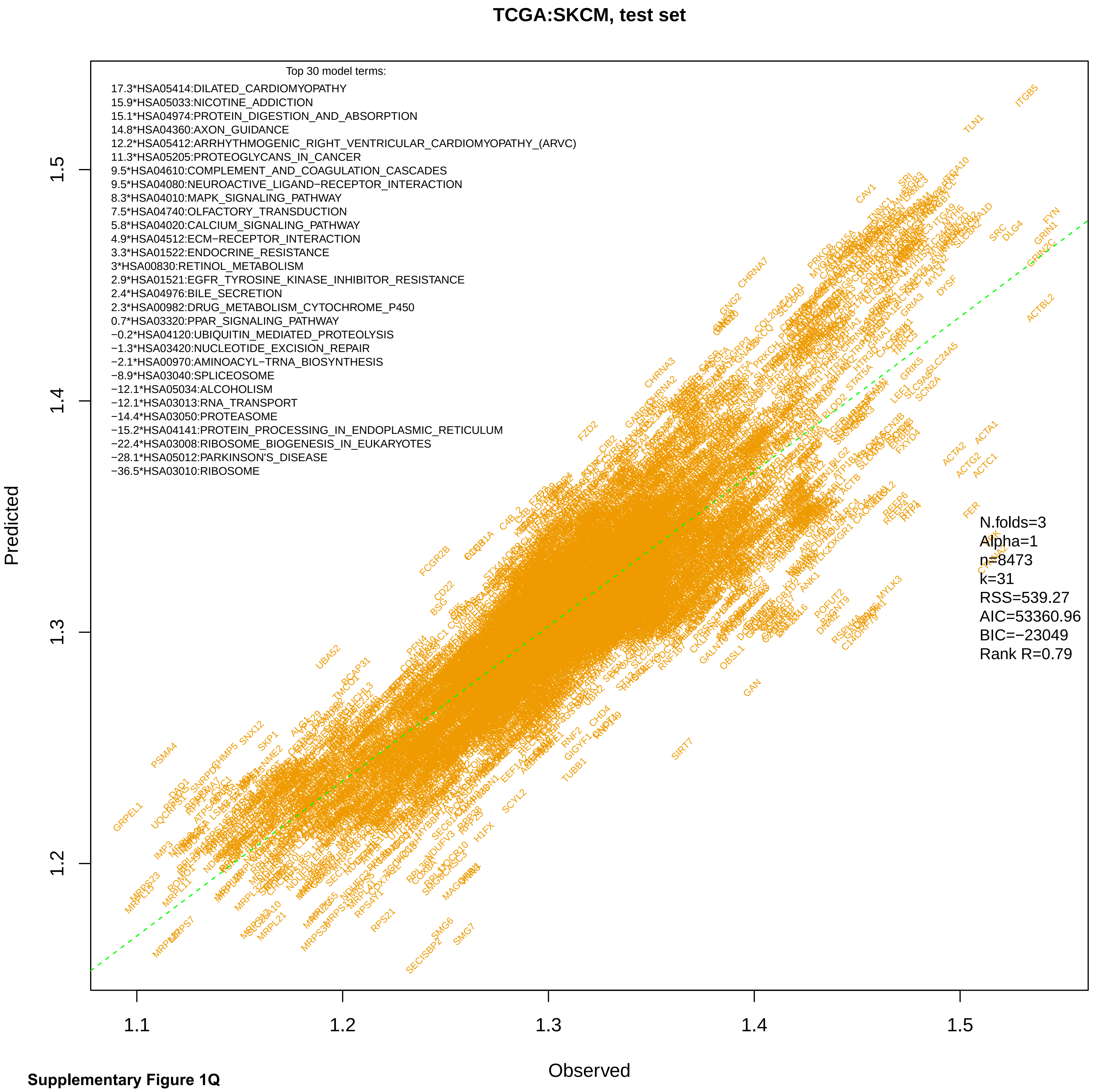


**Supplementary Figure 1Q**

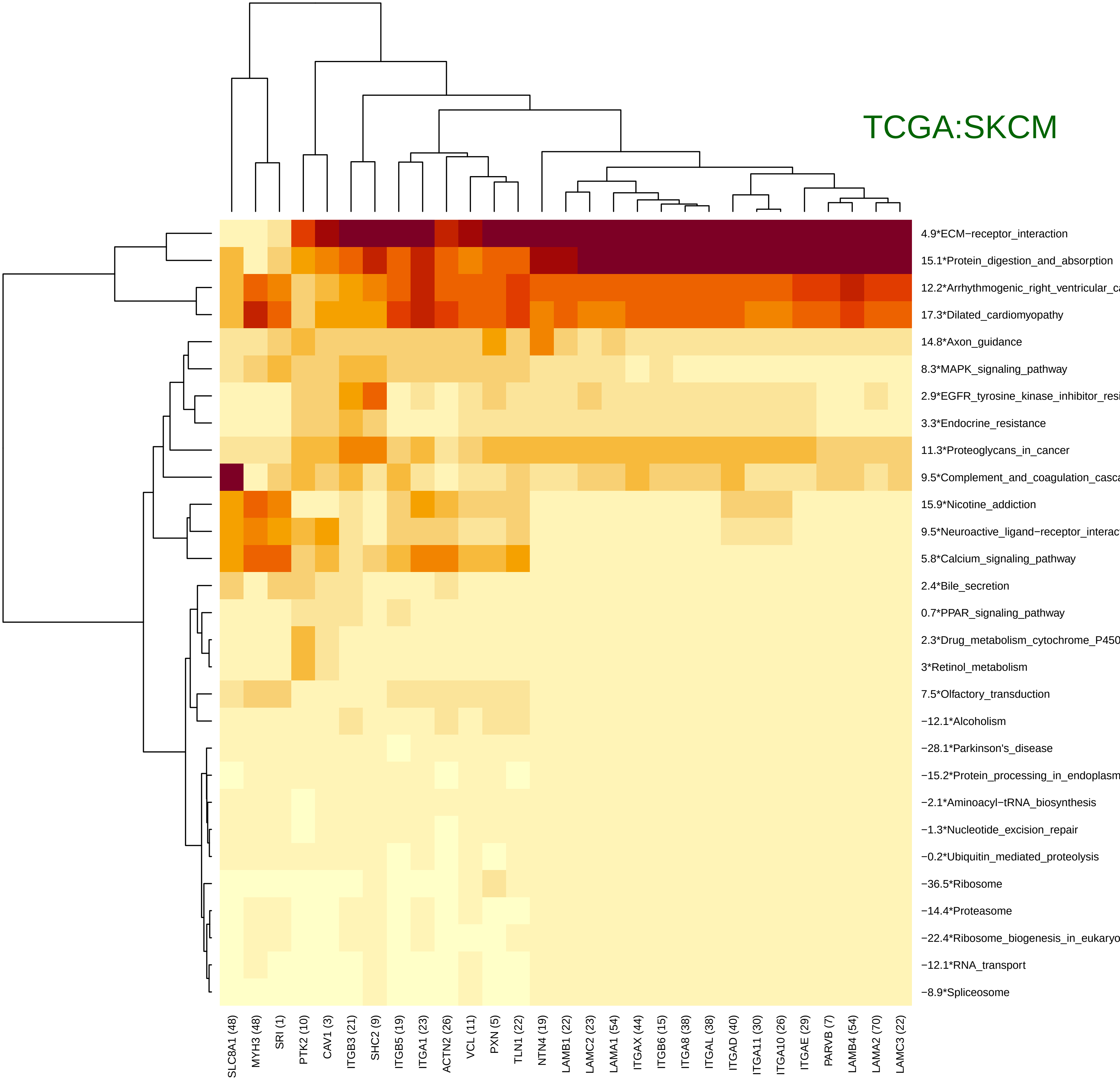


**Supplementary Figure 1R**

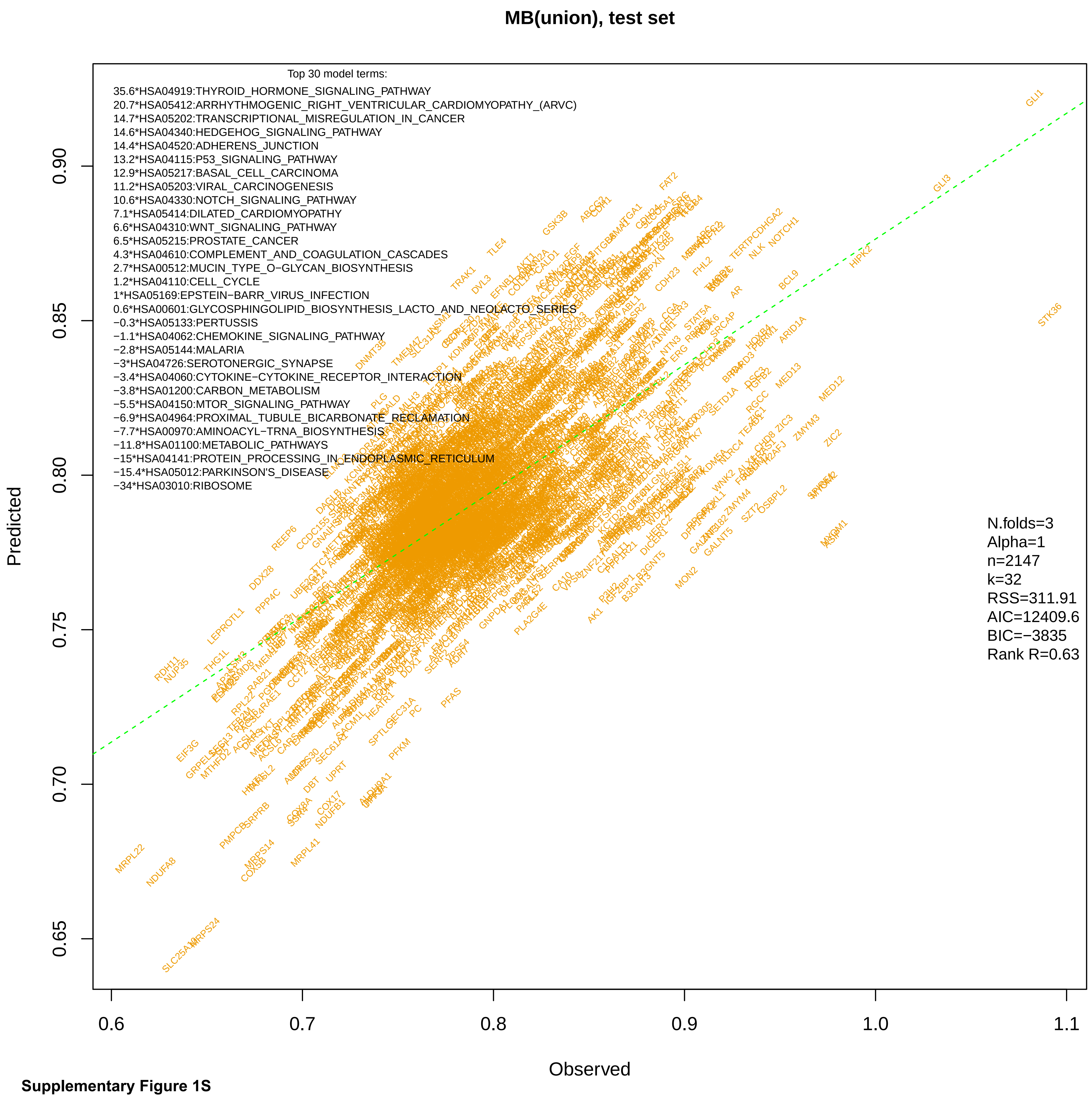


**Supplementary Figure 1S**

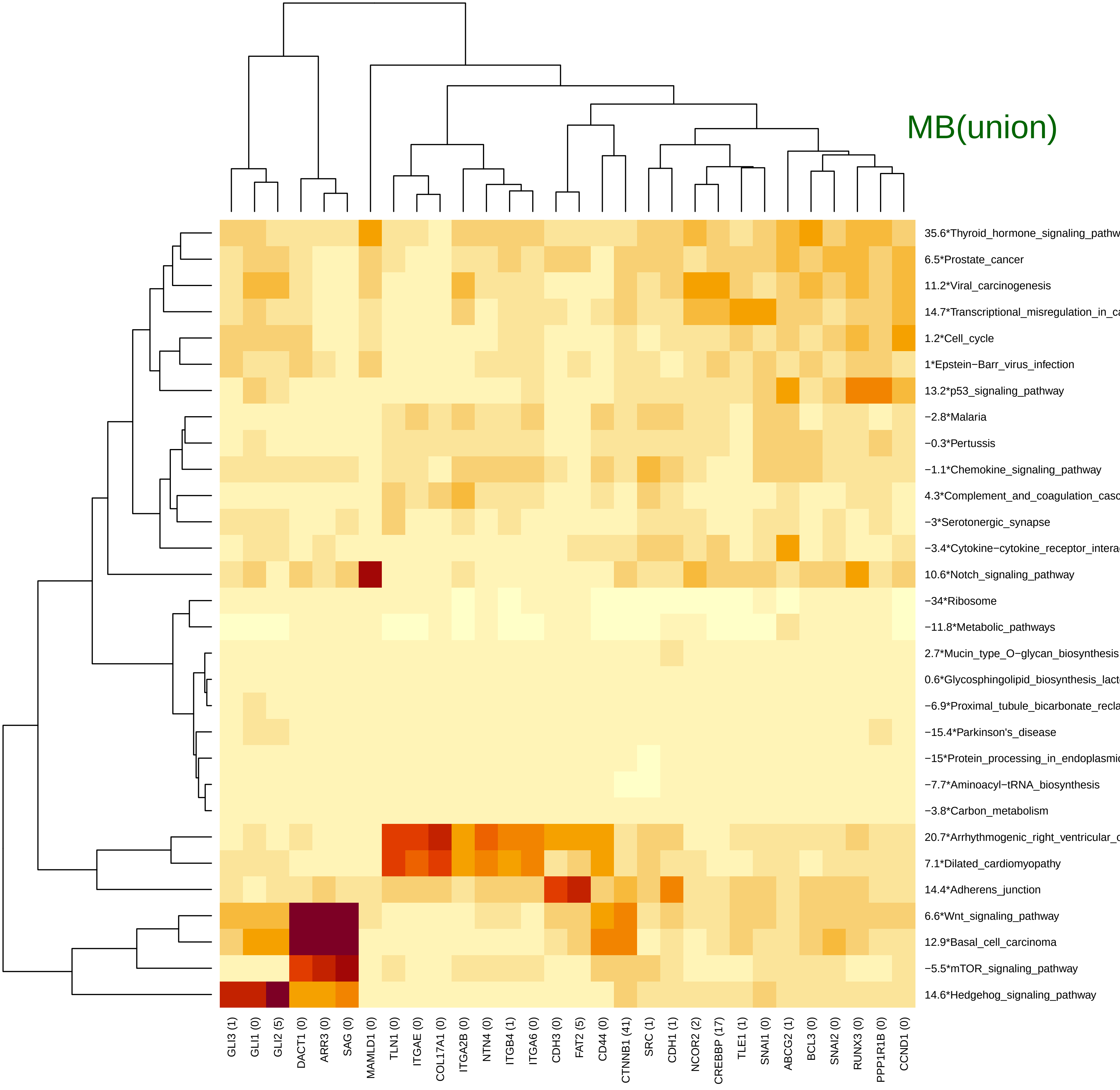


**Supplementary Figure 1T**
