# Supplementary figures and images for: Individualized discovery of rare cancer drivers in global network context (part 1 of 2)
Source: eLife. 2022 May 20;11:e74010. doi: 10.7554/eLife.74010 (PMC9159755; doi:10.7554/eLife.74010)

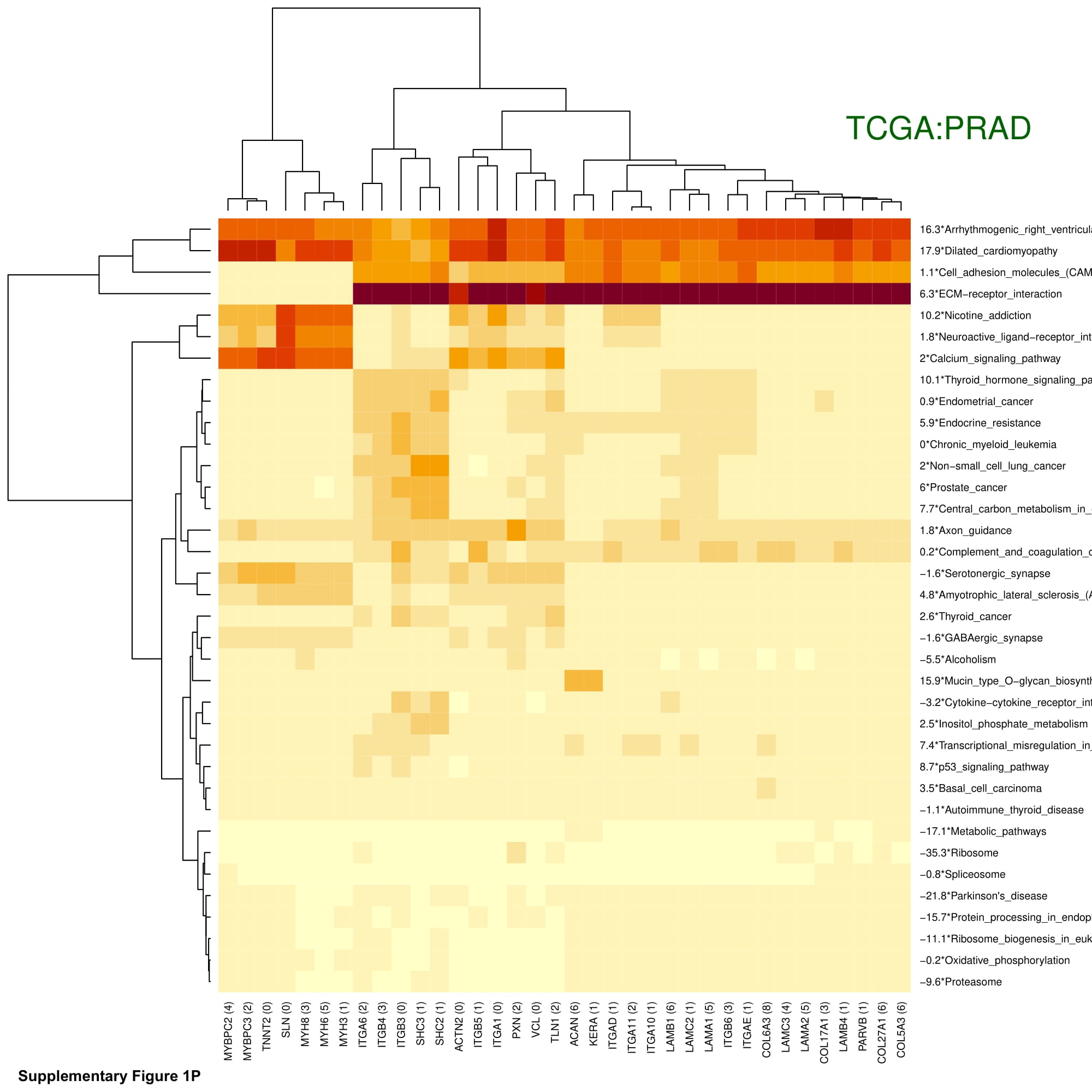

Supplement: Supplementary file 1. — Observed vs. predicted values of anchor.summary values represents performance of created models on continuous scale. In absence of a strict cut-off, performance was measured as a correlation between anchor.summary observed for each gene in the given cohort versus the a value predicted by the multiple regression model. In heatmaps, values next to gene names indicate number of samples with mutations in the given gene. [file elife-74010-supp1.zip › SupplementaryFigure1.Models/SupplementaryFigure1.Models-16.jpg]

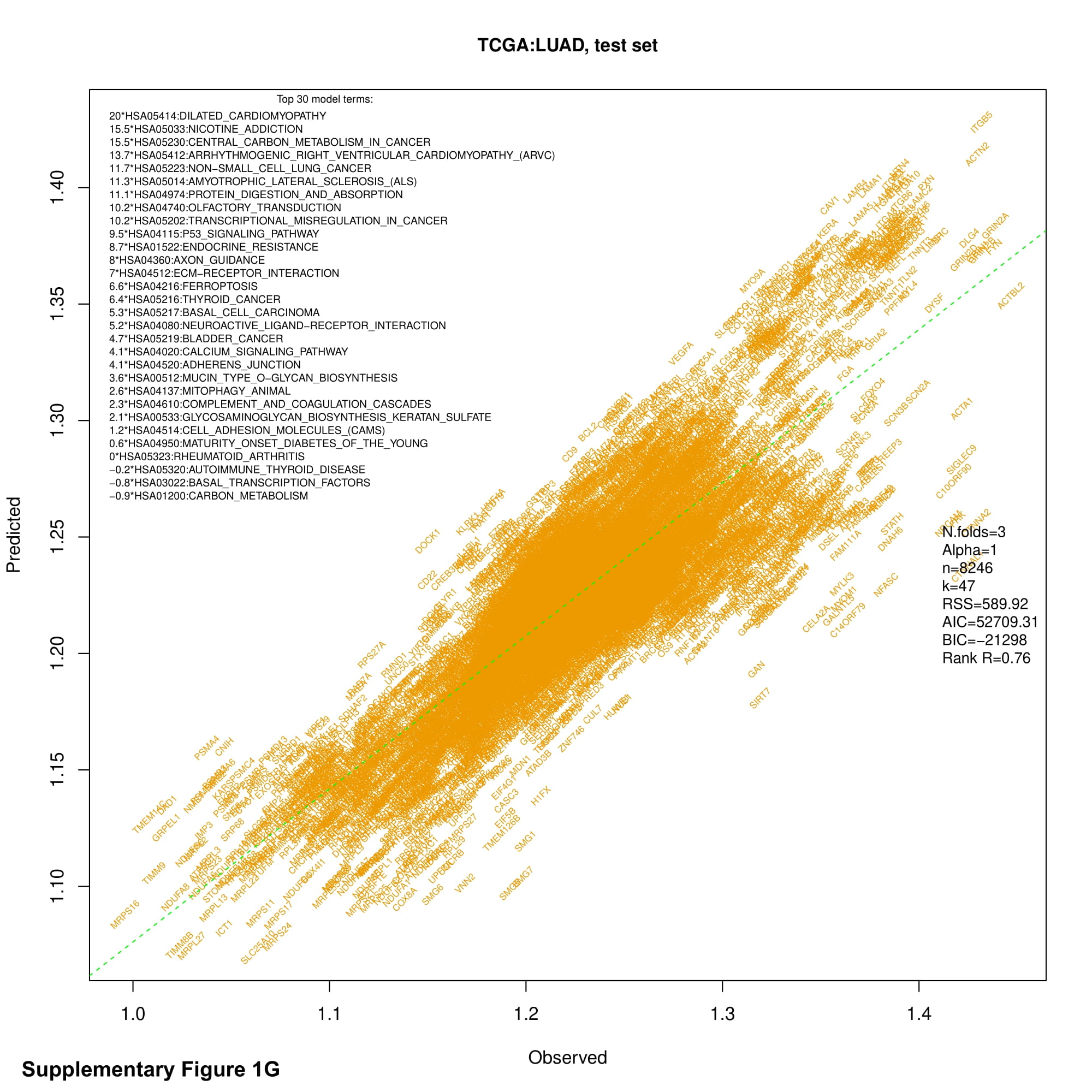

Supplement: Supplementary file 1. — Observed vs. predicted values of anchor.summary values represents performance of created models on continuous scale. In absence of a strict cut-off, performance was measured as a correlation between anchor.summary observed for each gene in the given cohort versus the a value predicted by the multiple regression model. In heatmaps, values next to gene names indicate number of samples with mutations in the given gene. [file elife-74010-supp1.zip › SupplementaryFigure1.Models/SupplementaryFigure1.Models-07.jpg]

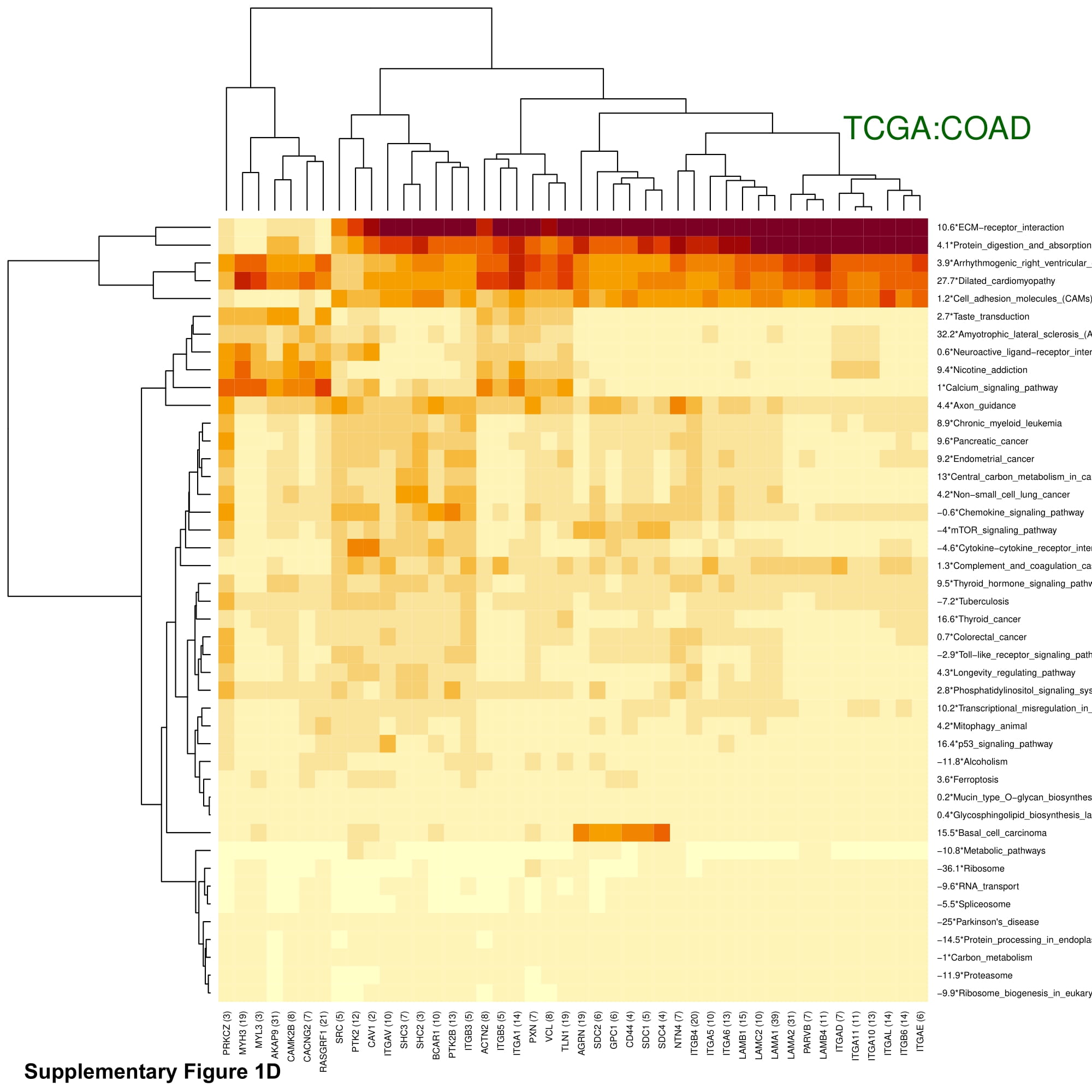

Supplement: Supplementary file 1. — Observed vs. predicted values of anchor.summary values represents performance of created models on continuous scale. In absence of a strict cut-off, performance was measured as a correlation between anchor.summary observed for each gene in the given cohort versus the a value predicted by the multiple regression model. In heatmaps, values next to gene names indicate number of samples with mutations in the given gene. [file elife-74010-supp1.zip › SupplementaryFigure1.Models/SupplementaryFigure1.Models-04.jpg]

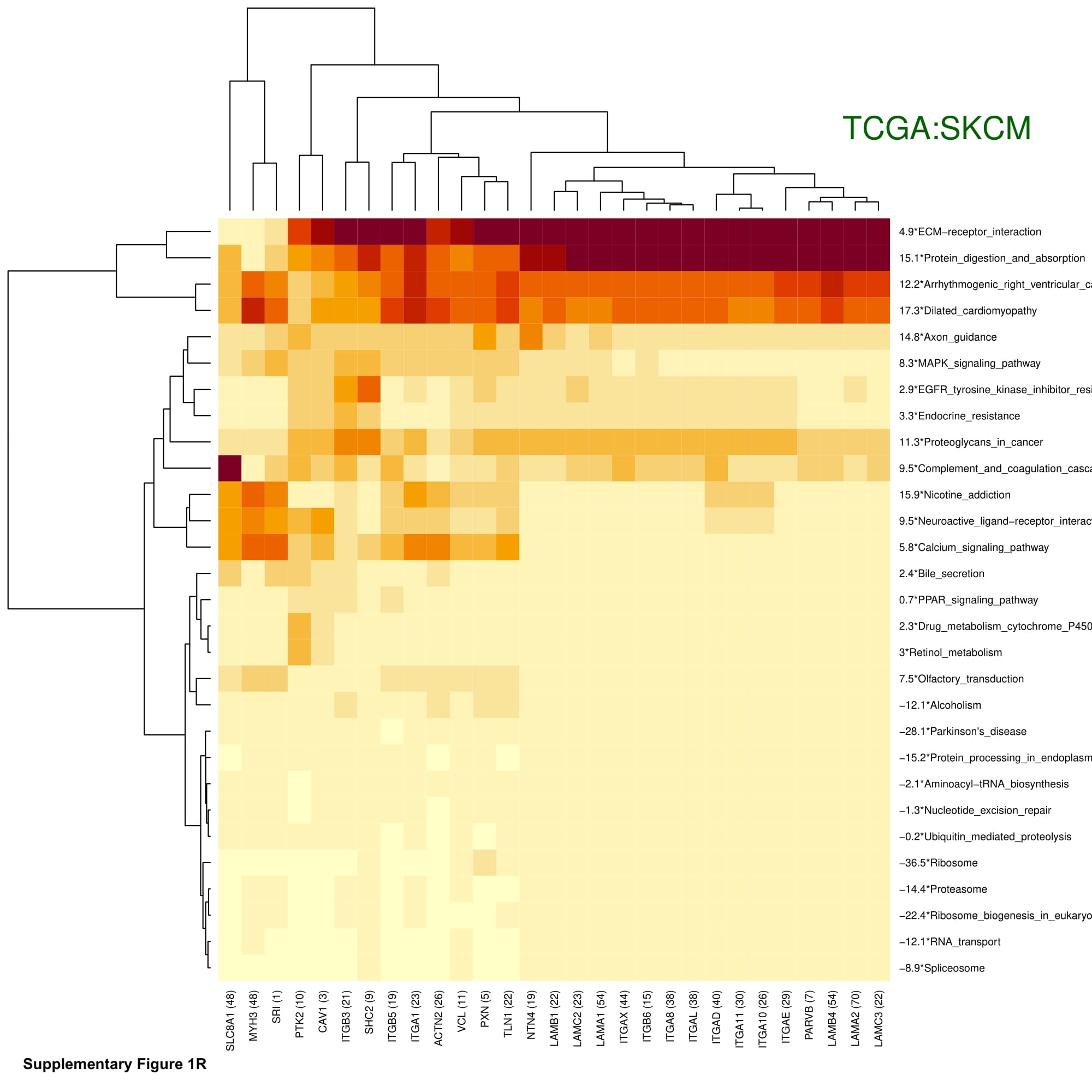

Supplement: Supplementary file 1. — Observed vs. predicted values of anchor.summary values represents performance of created models on continuous scale. In absence of a strict cut-off, performance was measured as a correlation between anchor.summary observed for each gene in the given cohort versus the a value predicted by the multiple regression model. In heatmaps, values next to gene names indicate number of samples with mutations in the given gene. [file elife-74010-supp1.zip › SupplementaryFigure1.Models/SupplementaryFigure1.Models-18.jpg]

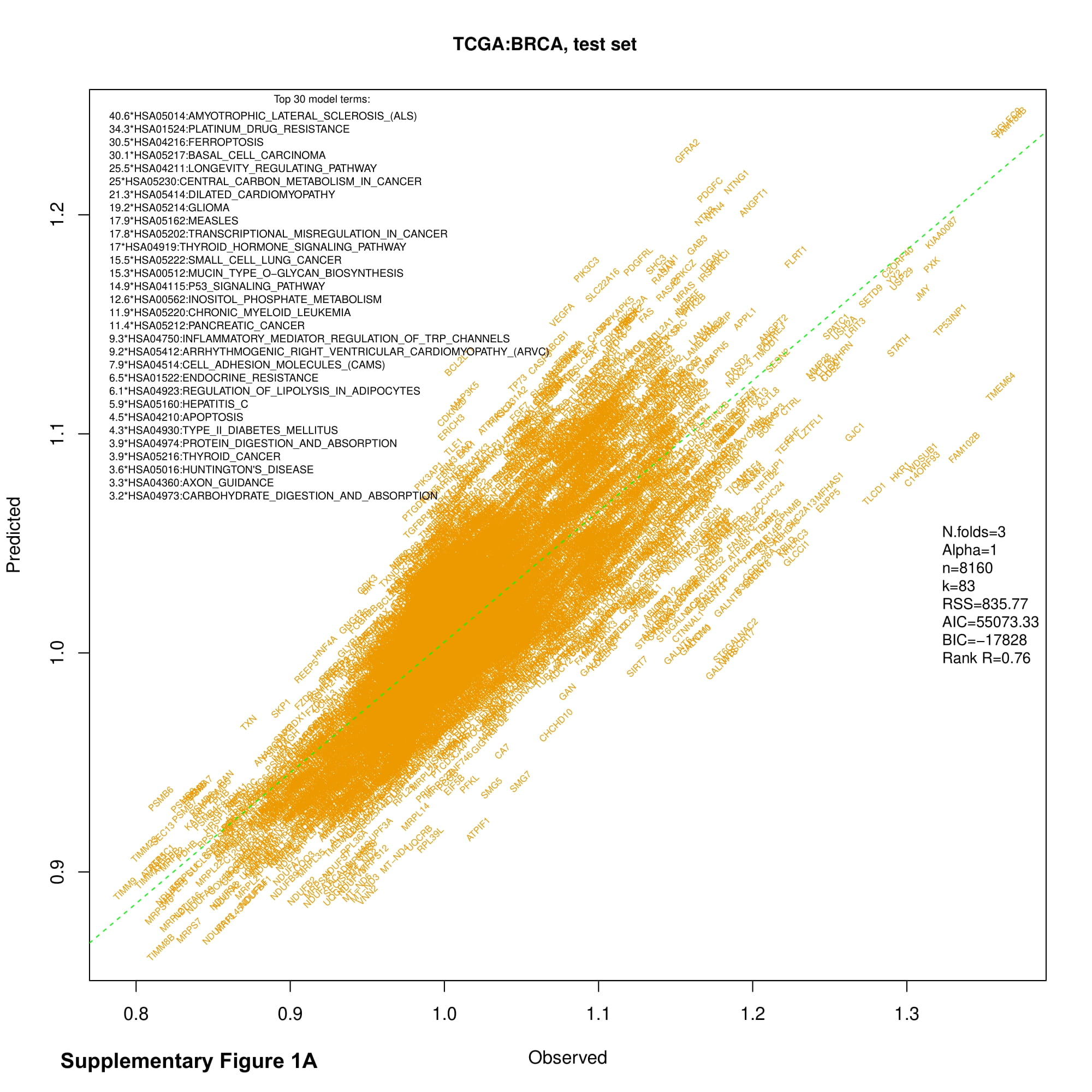

Supplement: Supplementary file 1. — Observed vs. predicted values of anchor.summary values represents performance of created models on continuous scale. In absence of a strict cut-off, performance was measured as a correlation between anchor.summary observed for each gene in the given cohort versus the a value predicted by the multiple regression model. In heatmaps, values next to gene names indicate number of samples with mutations in the given gene. [file elife-74010-supp1.zip › SupplementaryFigure1.Models/SupplementaryFigure1.Models-01.jpg]

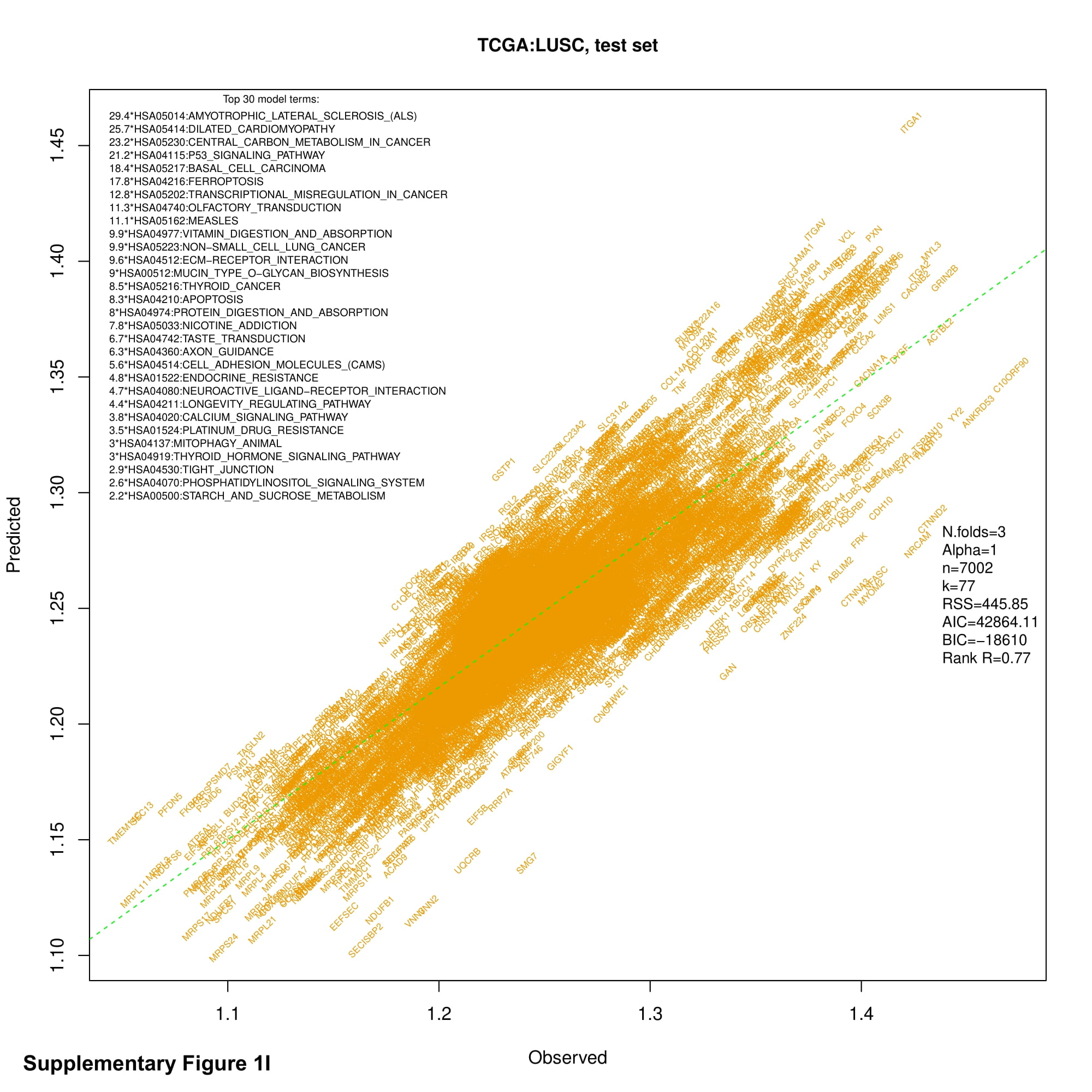

Supplement: Supplementary file 1. — Observed vs. predicted values of anchor.summary values represents performance of created models on continuous scale. In absence of a strict cut-off, performance was measured as a correlation between anchor.summary observed for each gene in the given cohort versus the a value predicted by the multiple regression model. In heatmaps, values next to gene names indicate number of samples with mutations in the given gene. [file elife-74010-supp1.zip › SupplementaryFigure1.Models/SupplementaryFigure1.Models-09.jpg]

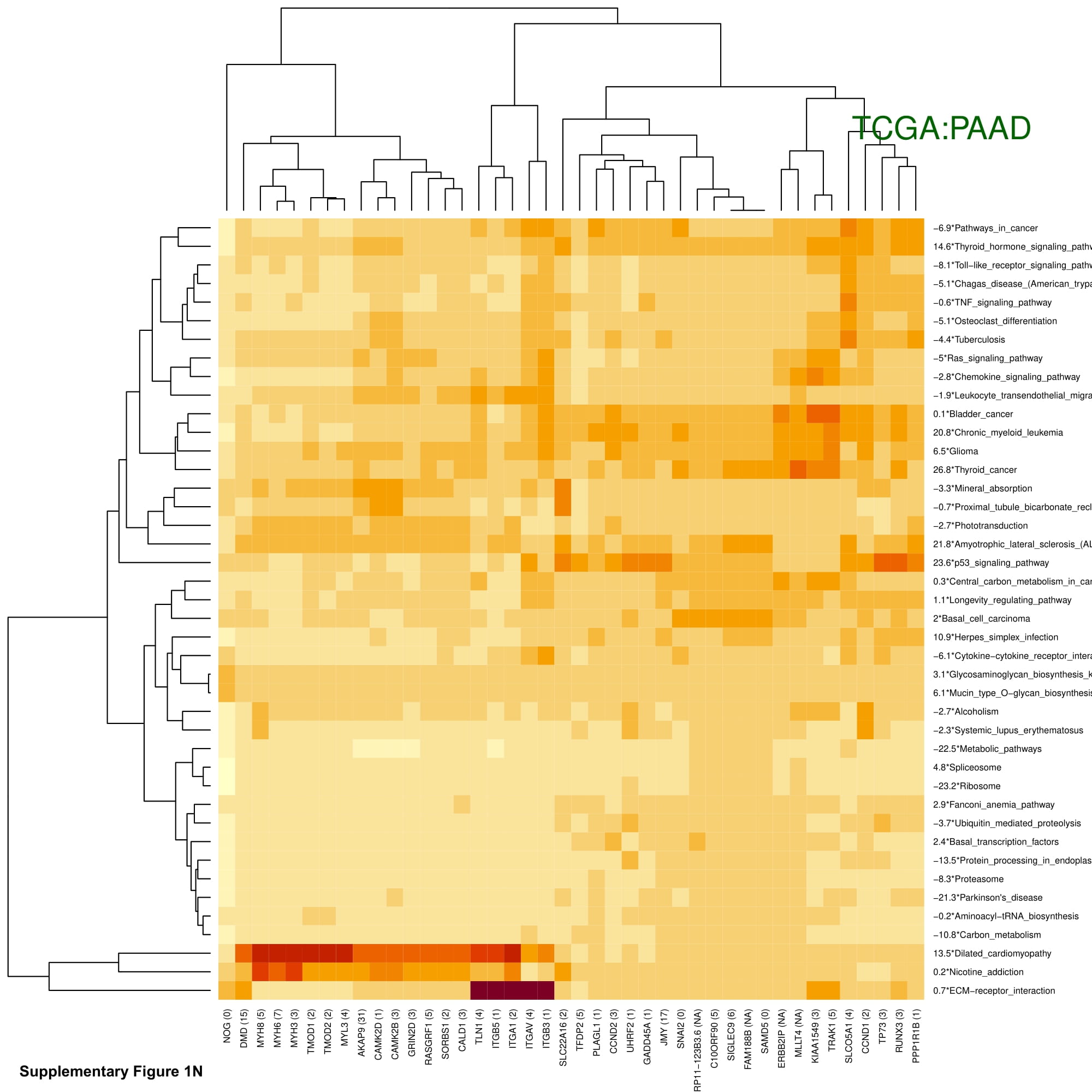

Supplement: Supplementary file 1. — Observed vs. predicted values of anchor.summary values represents performance of created models on continuous scale. In absence of a strict cut-off, performance was measured as a correlation between anchor.summary observed for each gene in the given cohort versus the a value predicted by the multiple regression model. In heatmaps, values next to gene names indicate number of samples with mutations in the given gene. [file elife-74010-supp1.zip › SupplementaryFigure1.Models/SupplementaryFigure1.Models-14.jpg]

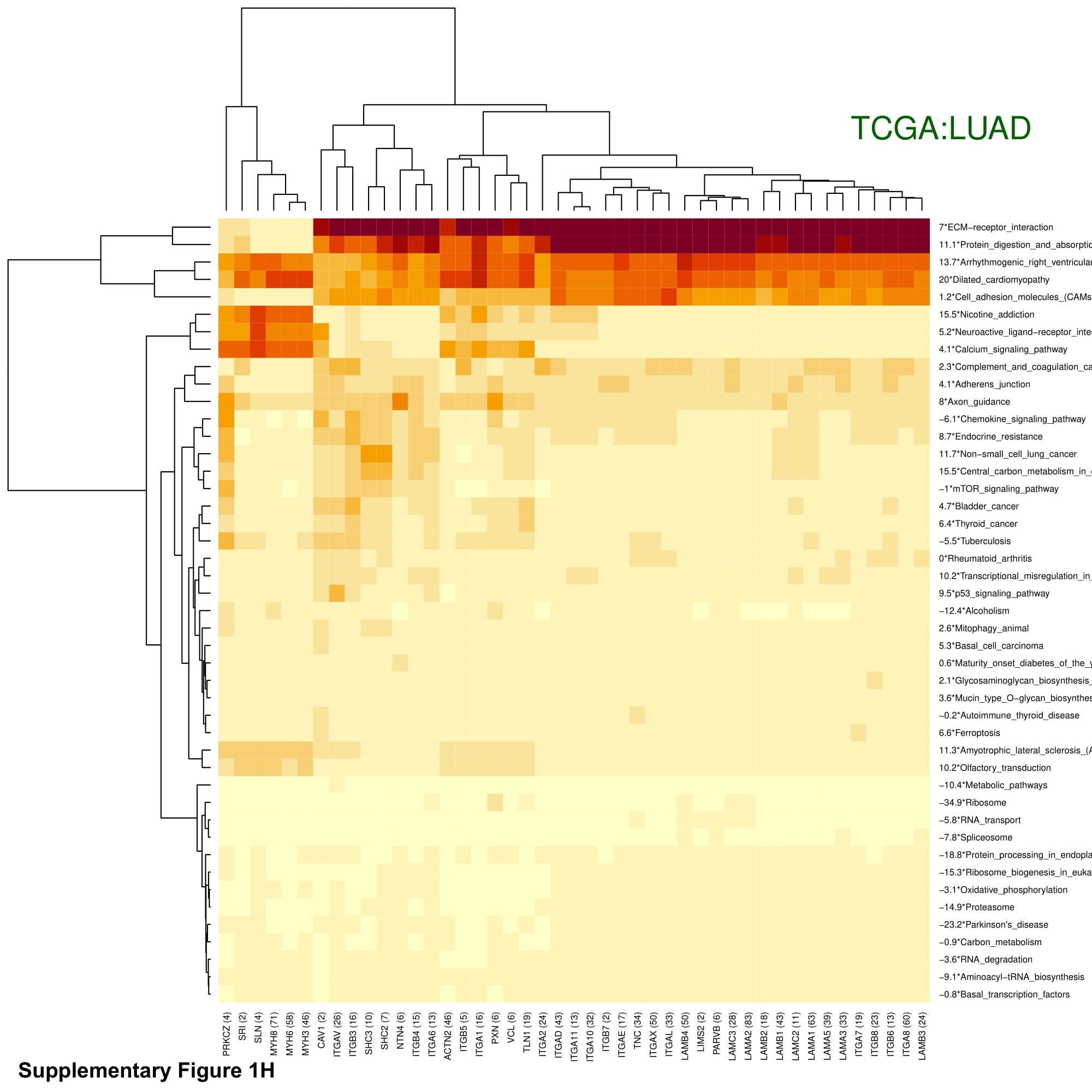

Supplement: Supplementary file 1. — Observed vs. predicted values of anchor.summary values represents performance of created models on continuous scale. In absence of a strict cut-off, performance was measured as a correlation between anchor.summary observed for each gene in the given cohort versus the a value predicted by the multiple regression model. In heatmaps, values next to gene names indicate number of samples with mutations in the given gene. [file elife-74010-supp1.zip › SupplementaryFigure1.Models/SupplementaryFigure1.Models-08.jpg]

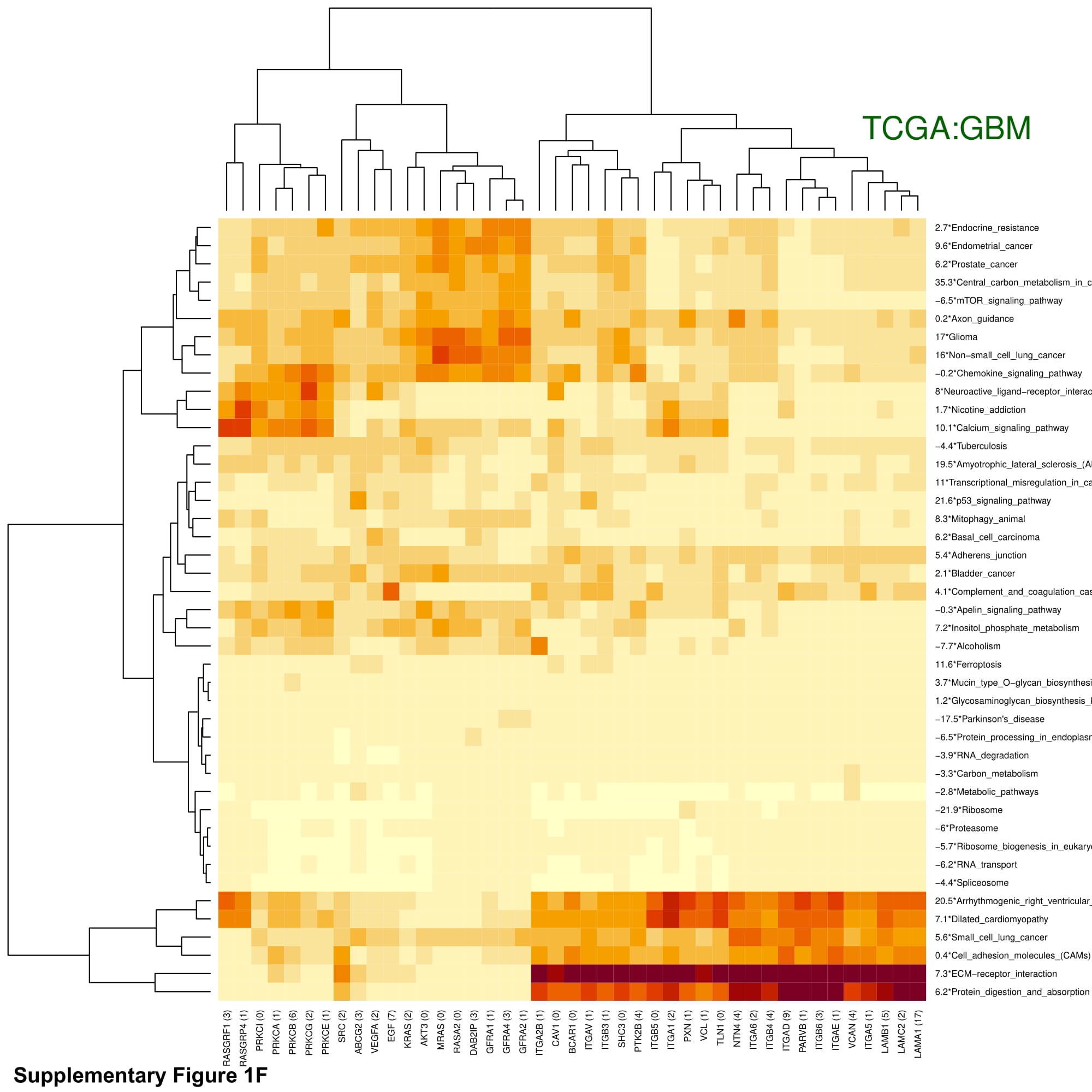

Supplement: Supplementary file 1. — Observed vs. predicted values of anchor.summary values represents performance of created models on continuous scale. In absence of a strict cut-off, performance was measured as a correlation between anchor.summary observed for each gene in the given cohort versus the a value predicted by the multiple regression model. In heatmaps, values next to gene names indicate number of samples with mutations in the given gene. [file elife-74010-supp1.zip › SupplementaryFigure1.Models/SupplementaryFigure1.Models-06.jpg]

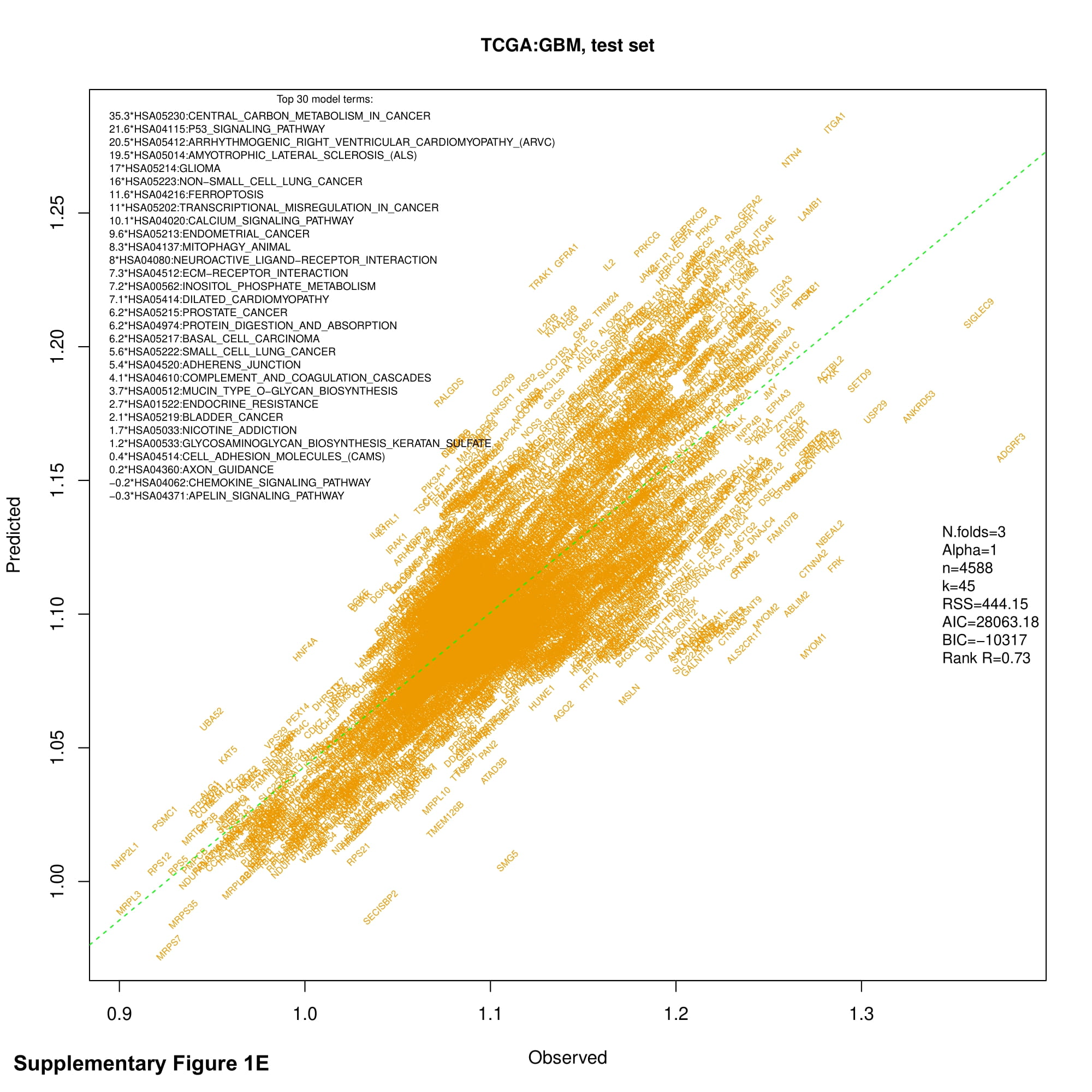

Supplement: Supplementary file 1. — Observed vs. predicted values of anchor.summary values represents performance of created models on continuous scale. In absence of a strict cut-off, performance was measured as a correlation between anchor.summary observed for each gene in the given cohort versus the a value predicted by the multiple regression model. In heatmaps, values next to gene names indicate number of samples with mutations in the given gene. [file elife-74010-supp1.zip › SupplementaryFigure1.Models/SupplementaryFigure1.Models-05.jpg]

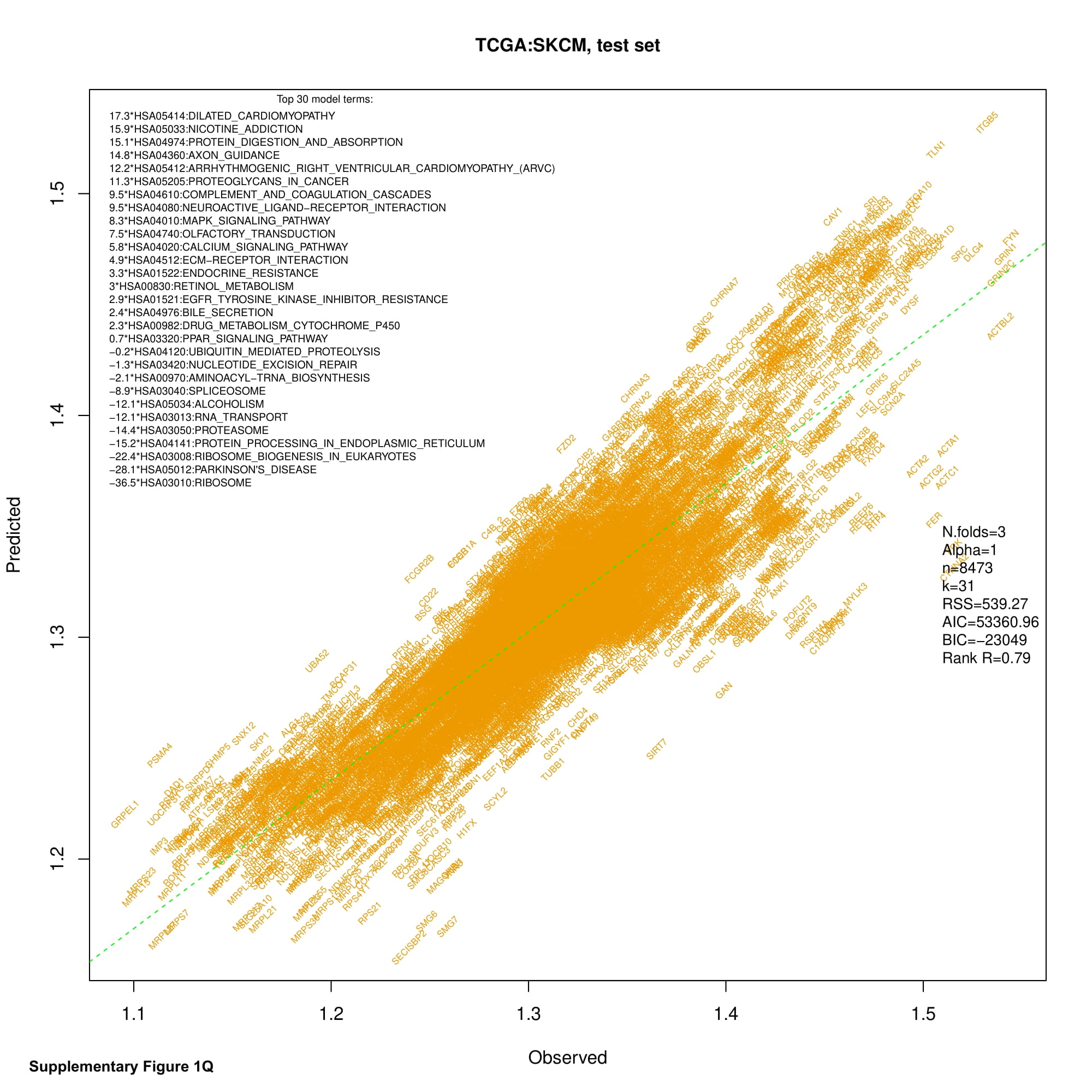

Supplement: Supplementary file 1. — Observed vs. predicted values of anchor.summary values represents performance of created models on continuous scale. In absence of a strict cut-off, performance was measured as a correlation between anchor.summary observed for each gene in the given cohort versus the a value predicted by the multiple regression model. In heatmaps, values next to gene names indicate number of samples with mutations in the given gene. [file elife-74010-supp1.zip › SupplementaryFigure1.Models/SupplementaryFigure1.Models-17.jpg]

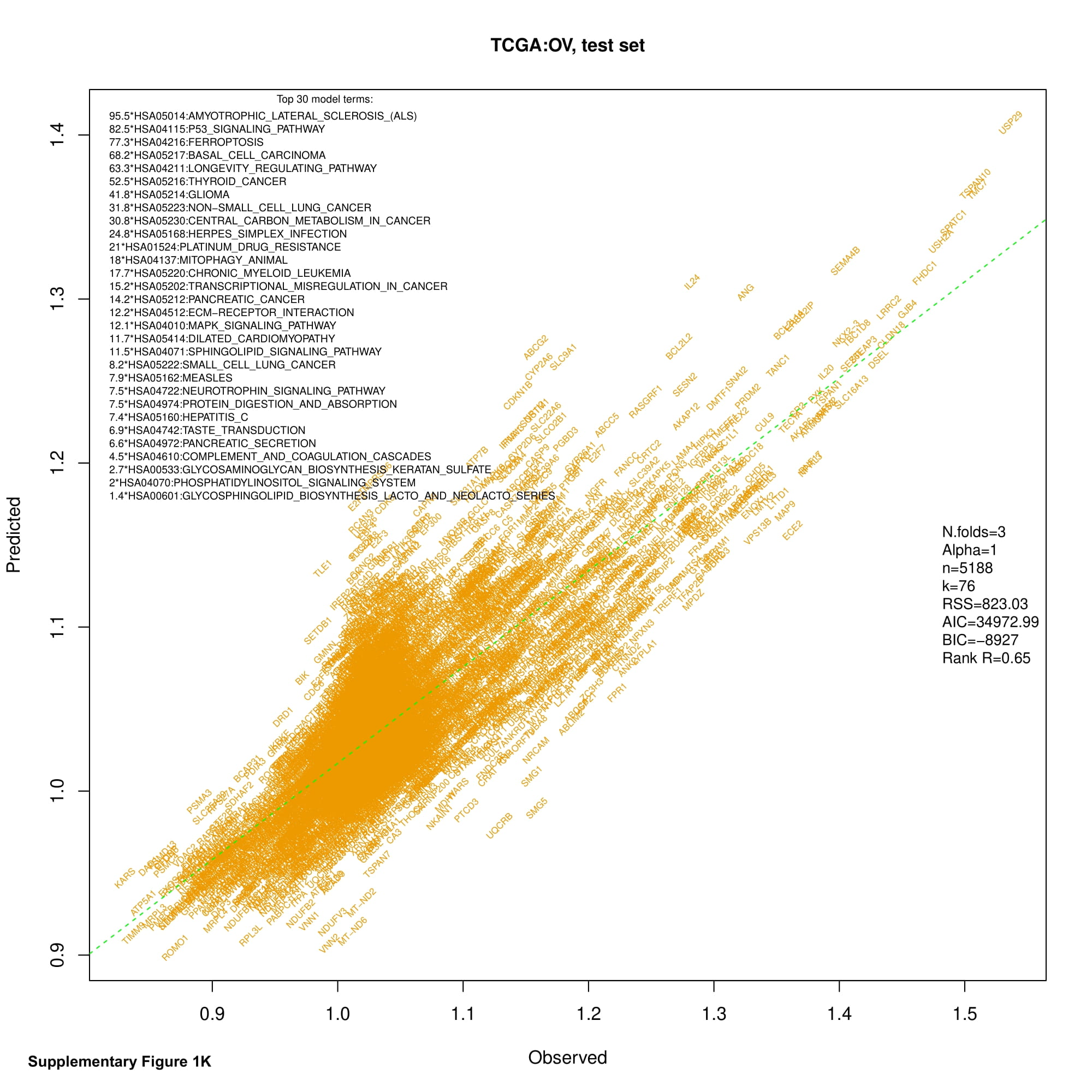

Supplement: Supplementary file 1. — Observed vs. predicted values of anchor.summary values represents performance of created models on continuous scale. In absence of a strict cut-off, performance was measured as a correlation between anchor.summary observed for each gene in the given cohort versus the a value predicted by the multiple regression model. In heatmaps, values next to gene names indicate number of samples with mutations in the given gene. [file elife-74010-supp1.zip › SupplementaryFigure1.Models/SupplementaryFigure1.Models-11.jpg]

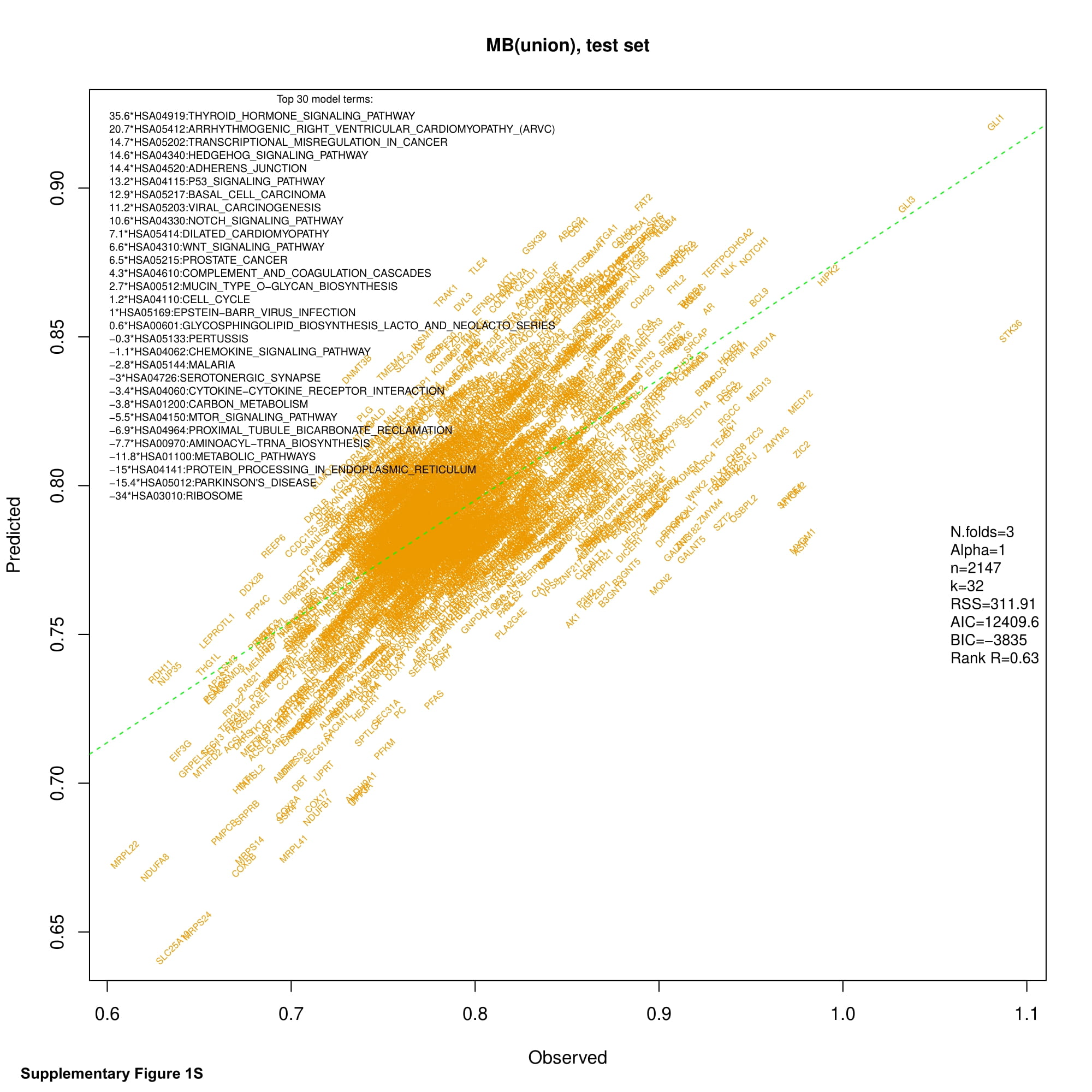

Supplement: Supplementary file 1. — Observed vs. predicted values of anchor.summary values represents performance of created models on continuous scale. In absence of a strict cut-off, performance was measured as a correlation between anchor.summary observed for each gene in the given cohort versus the a value predicted by the multiple regression model. In heatmaps, values next to gene names indicate number of samples with mutations in the given gene. [file elife-74010-supp1.zip › SupplementaryFigure1.Models/SupplementaryFigure1.Models-19.jpg]

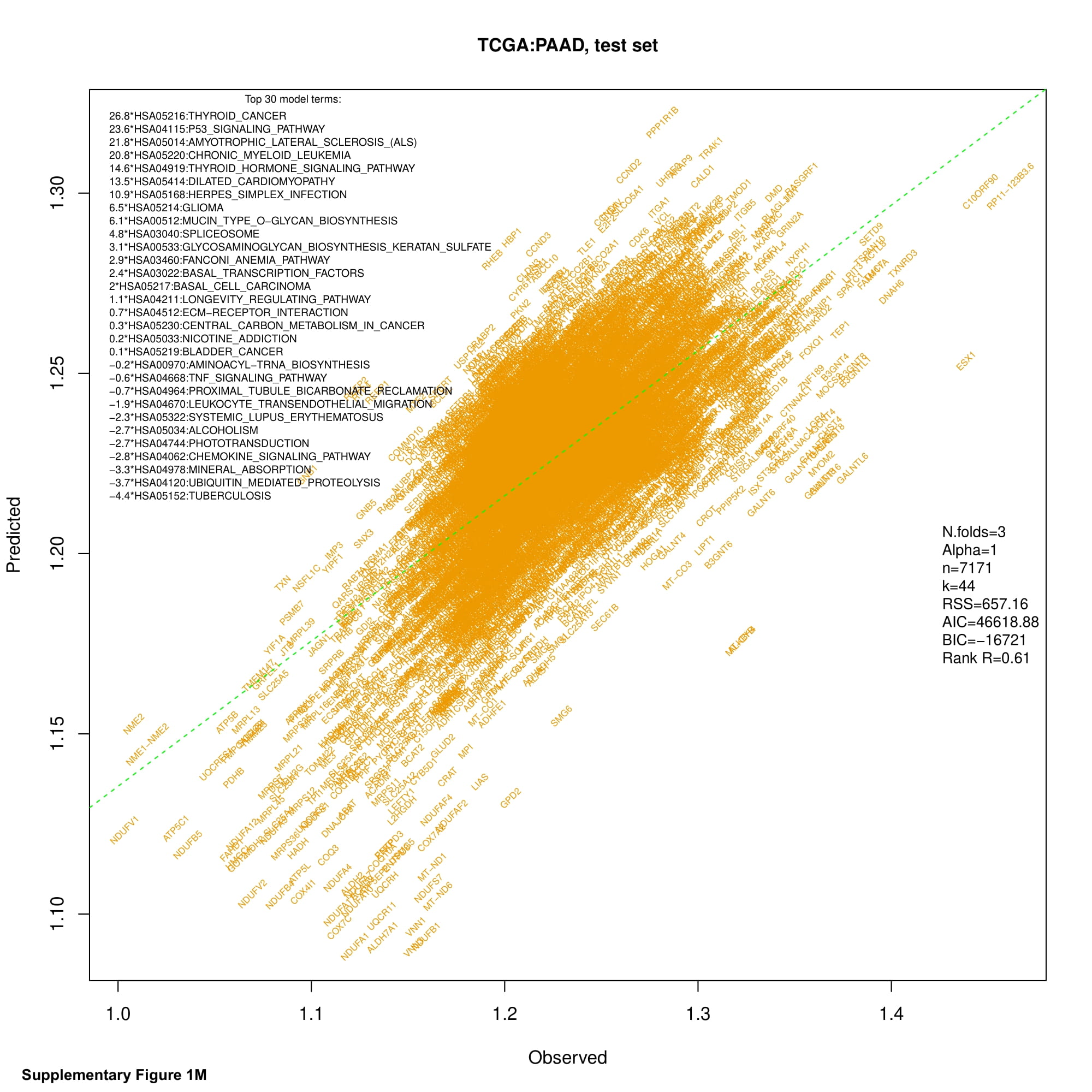

Supplement: Supplementary file 1. — Observed vs. predicted values of anchor.summary values represents performance of created models on continuous scale. In absence of a strict cut-off, performance was measured as a correlation between anchor.summary observed for each gene in the given cohort versus the a value predicted by the multiple regression model. In heatmaps, values next to gene names indicate number of samples with mutations in the given gene. [file elife-74010-supp1.zip › SupplementaryFigure1.Models/SupplementaryFigure1.Models-13.jpg]

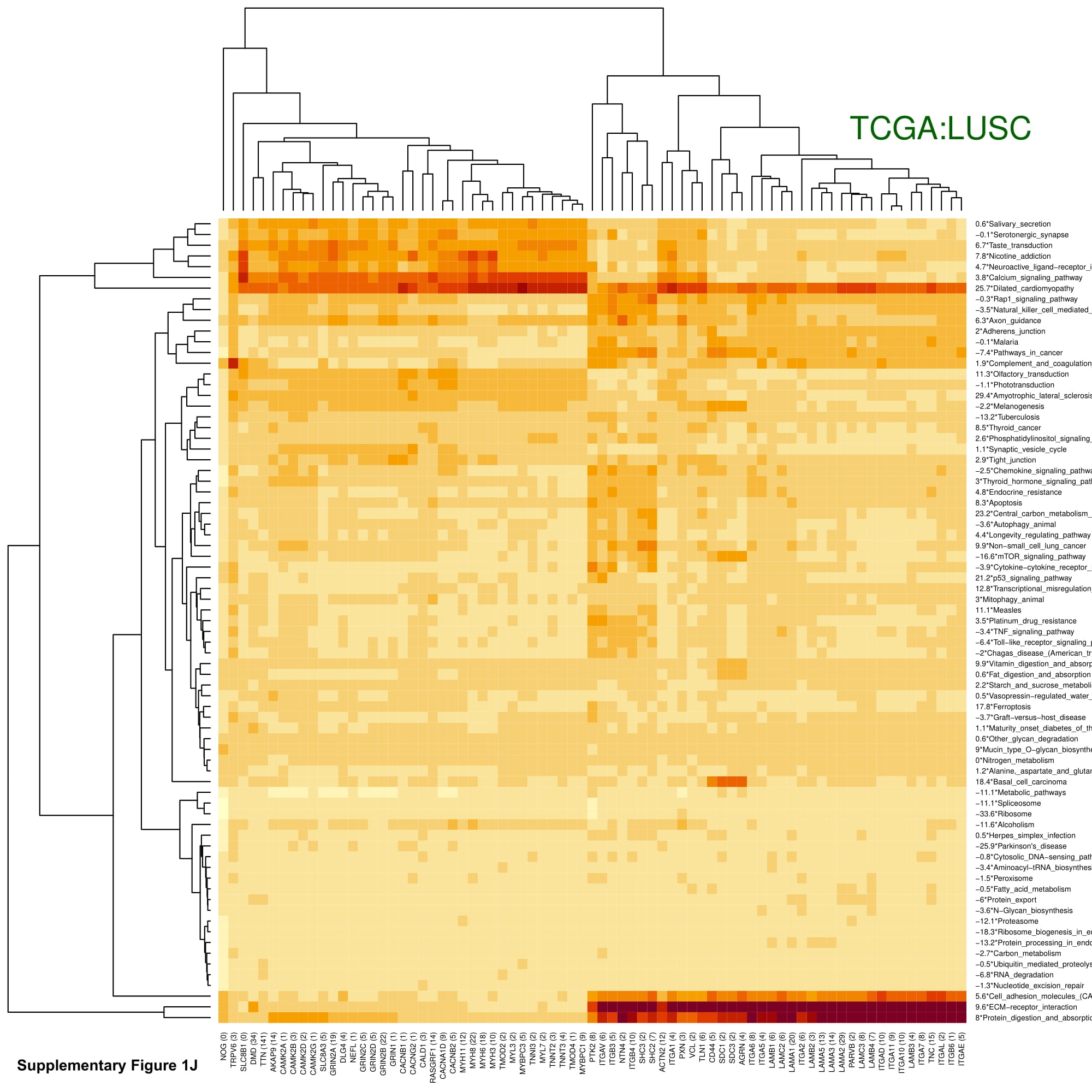

Supplement: Supplementary file 1. — Observed vs. predicted values of anchor.summary values represents performance of created models on continuous scale. In absence of a strict cut-off, performance was measured as a correlation between anchor.summary observed for each gene in the given cohort versus the a value predicted by the multiple regression model. In heatmaps, values next to gene names indicate number of samples with mutations in the given gene. [file elife-74010-supp1.zip › SupplementaryFigure1.Models/SupplementaryFigure1.Models-10.jpg]

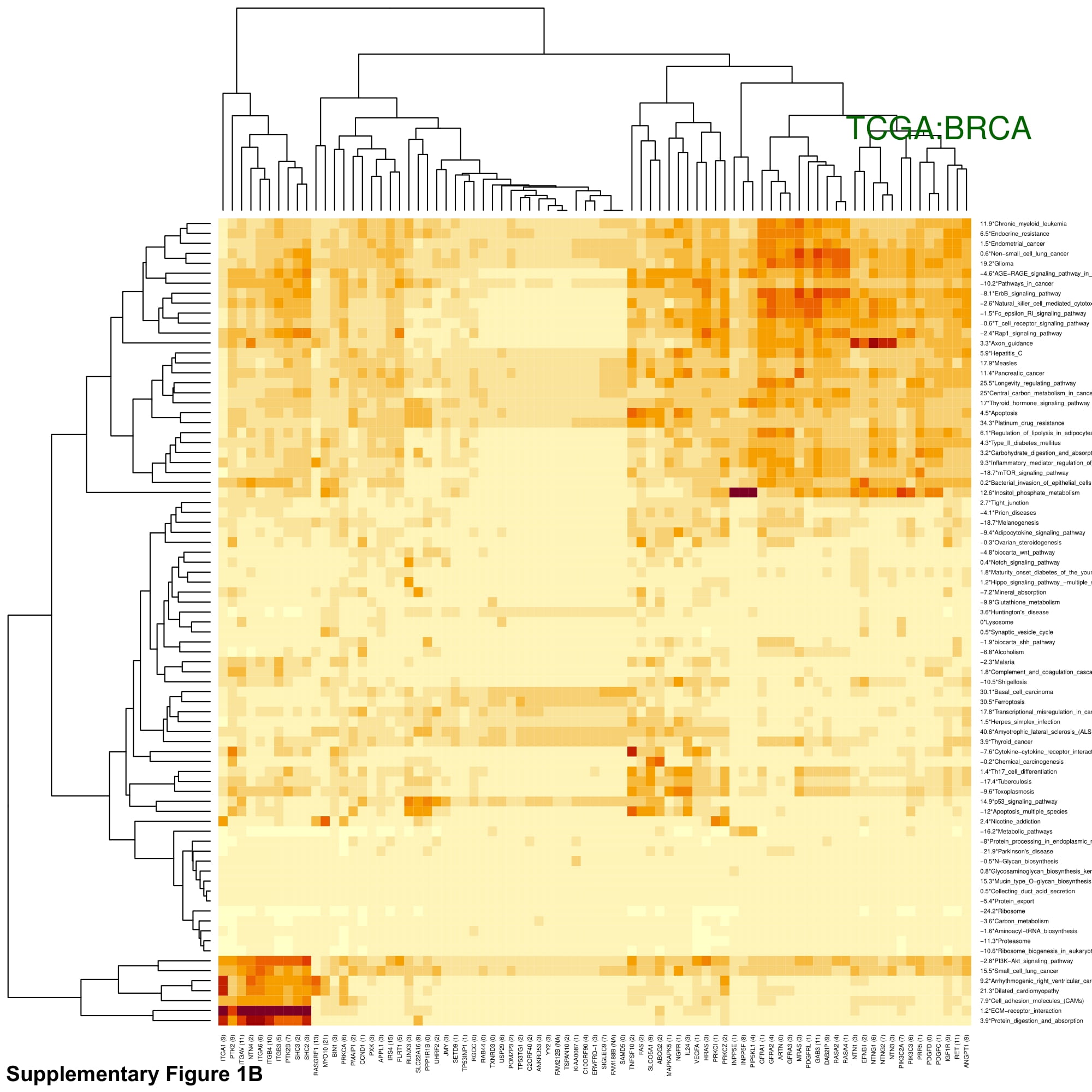

Supplement: Supplementary file 1. — Observed vs. predicted values of anchor.summary values represents performance of created models on continuous scale. In absence of a strict cut-off, performance was measured as a correlation between anchor.summary observed for each gene in the given cohort versus the a value predicted by the multiple regression model. In heatmaps, values next to gene names indicate number of samples with mutations in the given gene. [file elife-74010-supp1.zip › SupplementaryFigure1.Models/SupplementaryFigure1.Models-02.jpg]

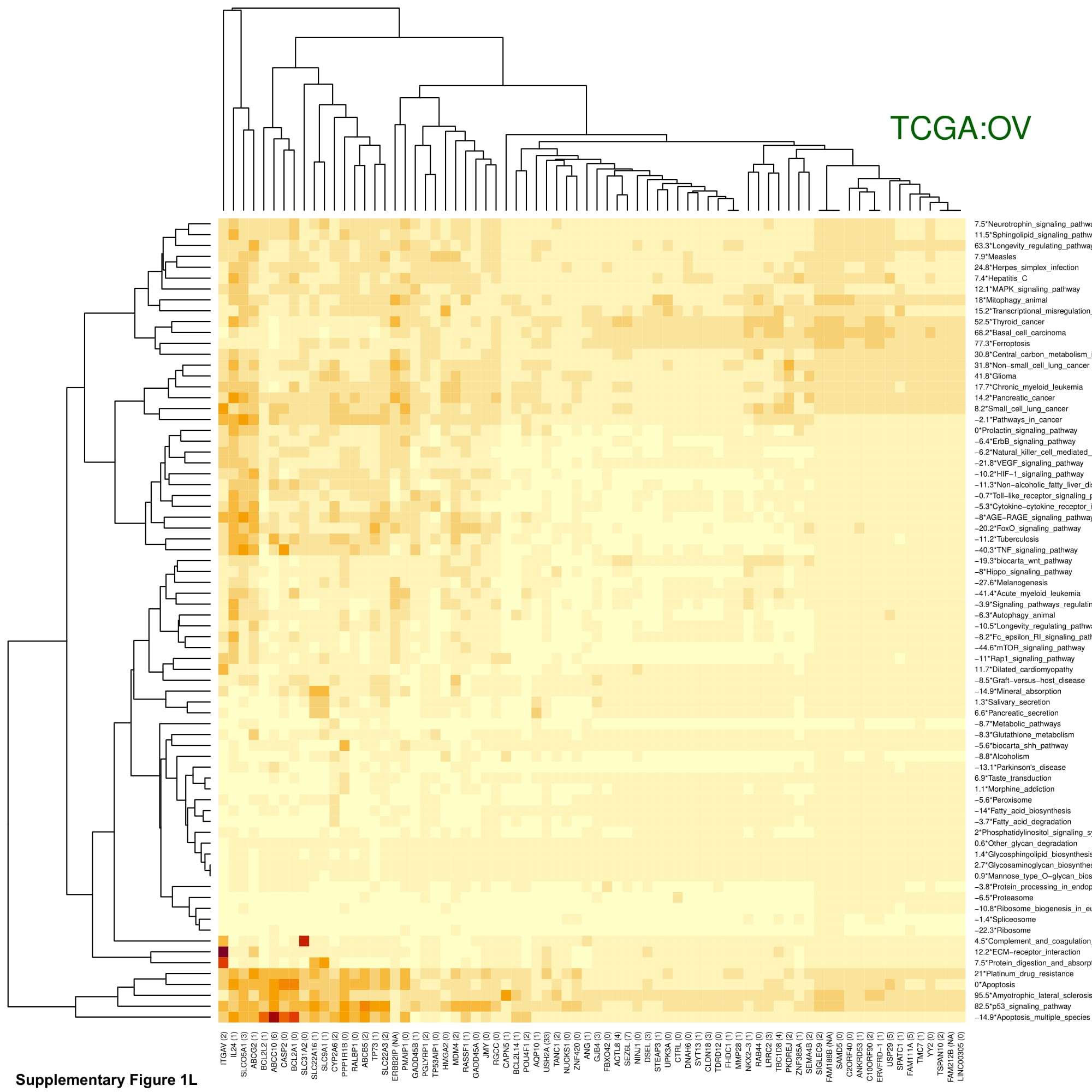

Supplement: Supplementary file 1. — Observed vs. predicted values of anchor.summary values represents performance of created models on continuous scale. In absence of a strict cut-off, performance was measured as a correlation between anchor.summary observed for each gene in the given cohort versus the a value predicted by the multiple regression model. In heatmaps, values next to gene names indicate number of samples with mutations in the given gene. [file elife-74010-supp1.zip › SupplementaryFigure1.Models/SupplementaryFigure1.Models-12.jpg]

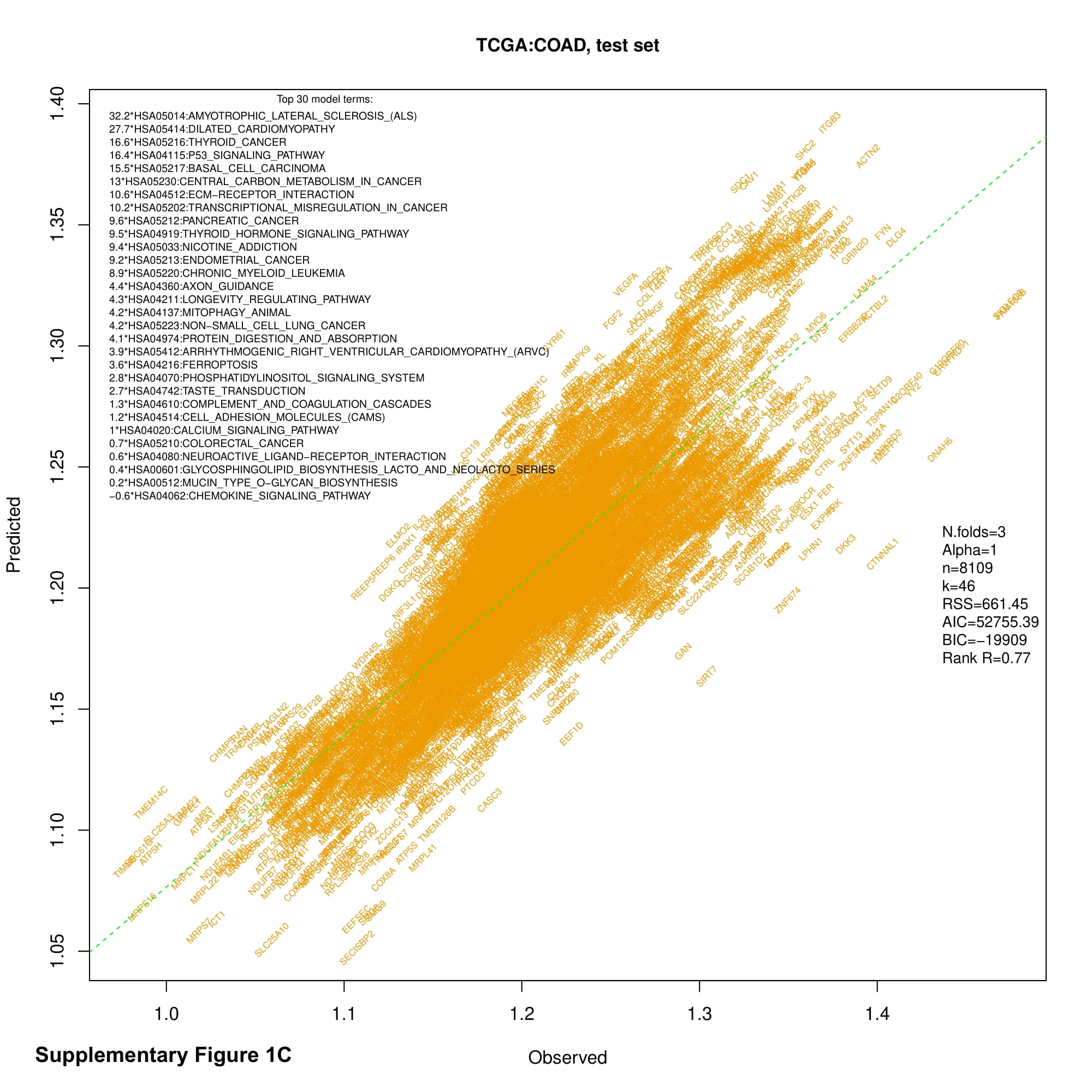

Supplement: Supplementary file 1. — Observed vs. predicted values of anchor.summary values represents performance of created models on continuous scale. In absence of a strict cut-off, performance was measured as a correlation between anchor.summary observed for each gene in the given cohort versus the a value predicted by the multiple regression model. In heatmaps, values next to gene names indicate number of samples with mutations in the given gene. [file elife-74010-supp1.zip › SupplementaryFigure1.Models/SupplementaryFigure1.Models-03.jpg]

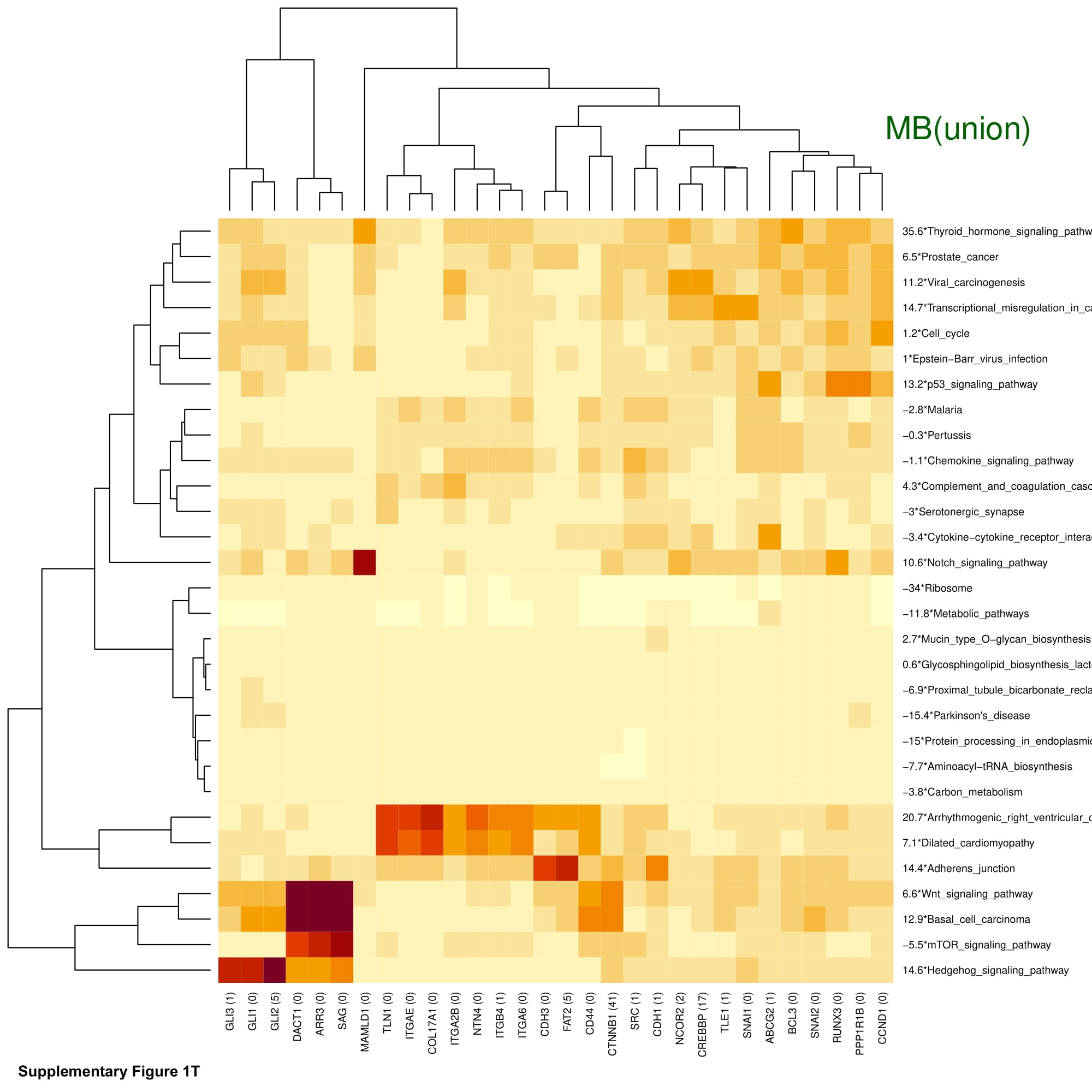

Supplement: Supplementary file 1. — Observed vs. predicted values of anchor.summary values represents performance of created models on continuous scale. In absence of a strict cut-off, performance was measured as a correlation between anchor.summary observed for each gene in the given cohort versus the a value predicted by the multiple regression model. In heatmaps, values next to gene names indicate number of samples with mutations in the given gene. [file elife-74010-supp1.zip › SupplementaryFigure1.Models/SupplementaryFigure1.Models-20.jpg]

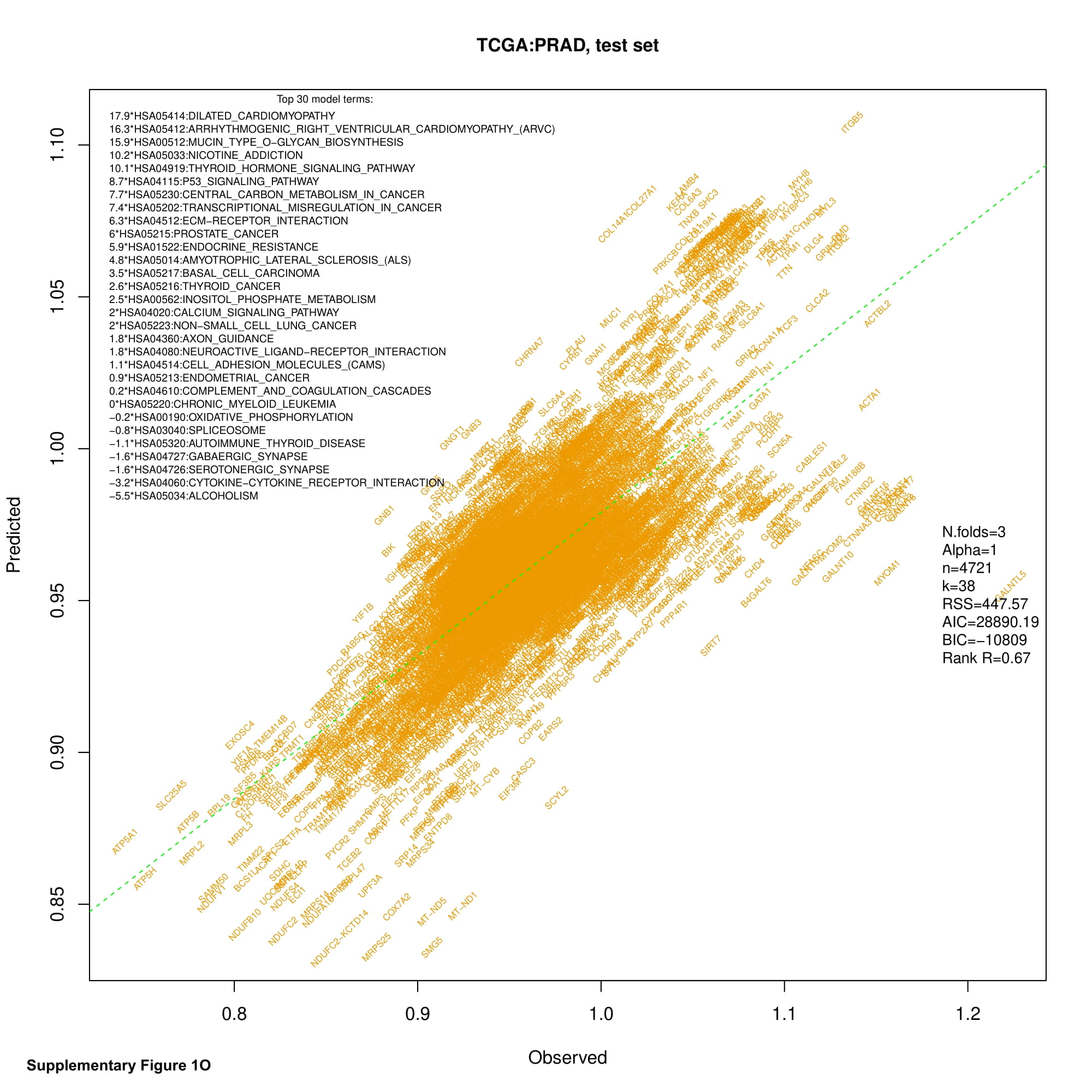

Supplement: Supplementary file 1. — Observed vs. predicted values of anchor.summary values represents performance of created models on continuous scale. In absence of a strict cut-off, performance was measured as a correlation between anchor.summary observed for each gene in the given cohort versus the a value predicted by the multiple regression model. In heatmaps, values next to gene names indicate number of samples with mutations in the given gene. [file elife-74010-supp1.zip › SupplementaryFigure1.Models/SupplementaryFigure1.Models-15.jpg]

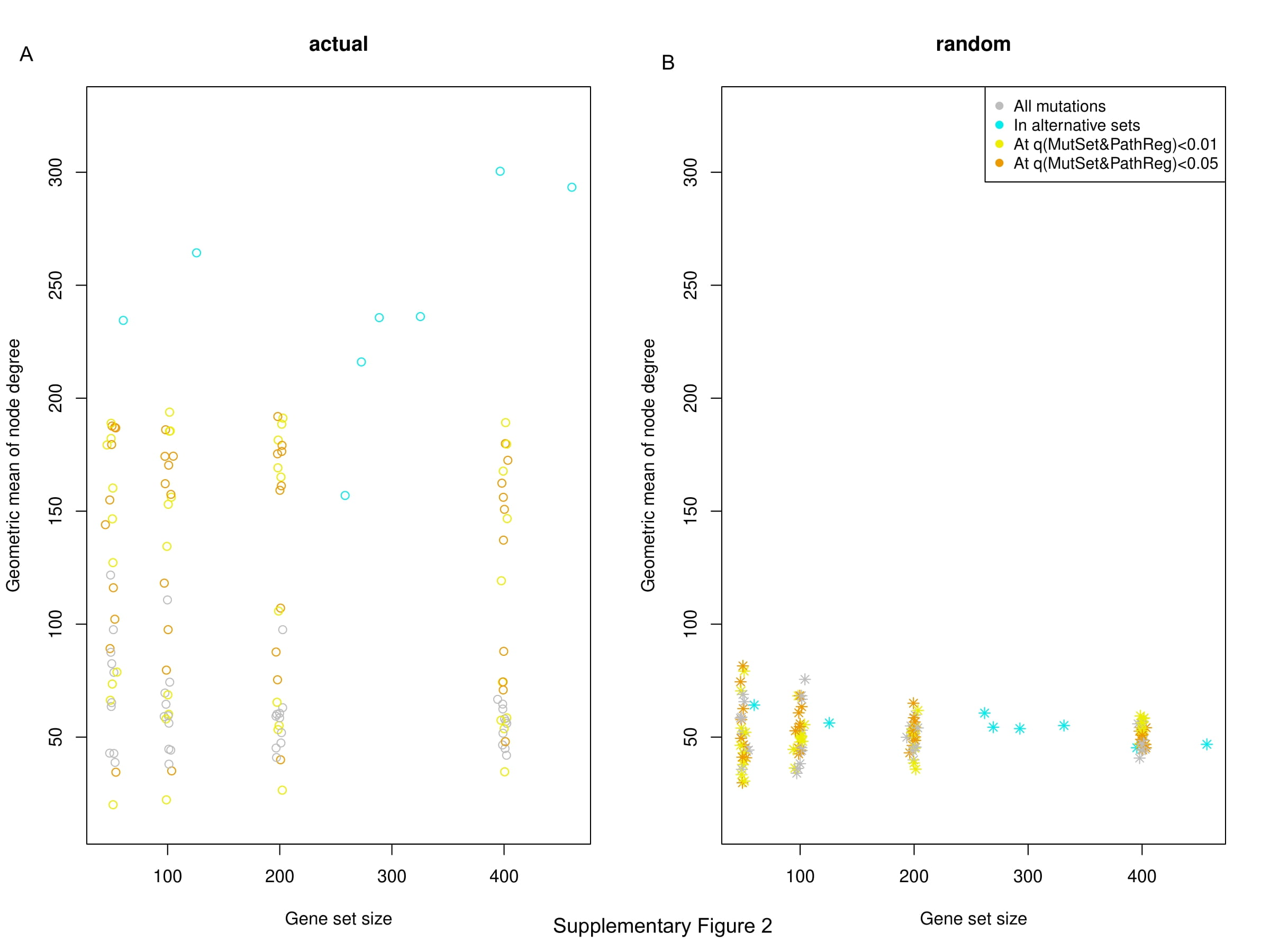

Supplement: Supplementary file 2. — X coordinates for the alternative sets represent their sizes. For NEAdriver (which in total, across all cohort samples typically predicted hundreds genes, most being very rare) the sets represent samples n=[50, 100, 200, 400] genes most frequently predicted in each cohort. [file elife-74010-supp2.zip › SupplementaryFile2.jpg]

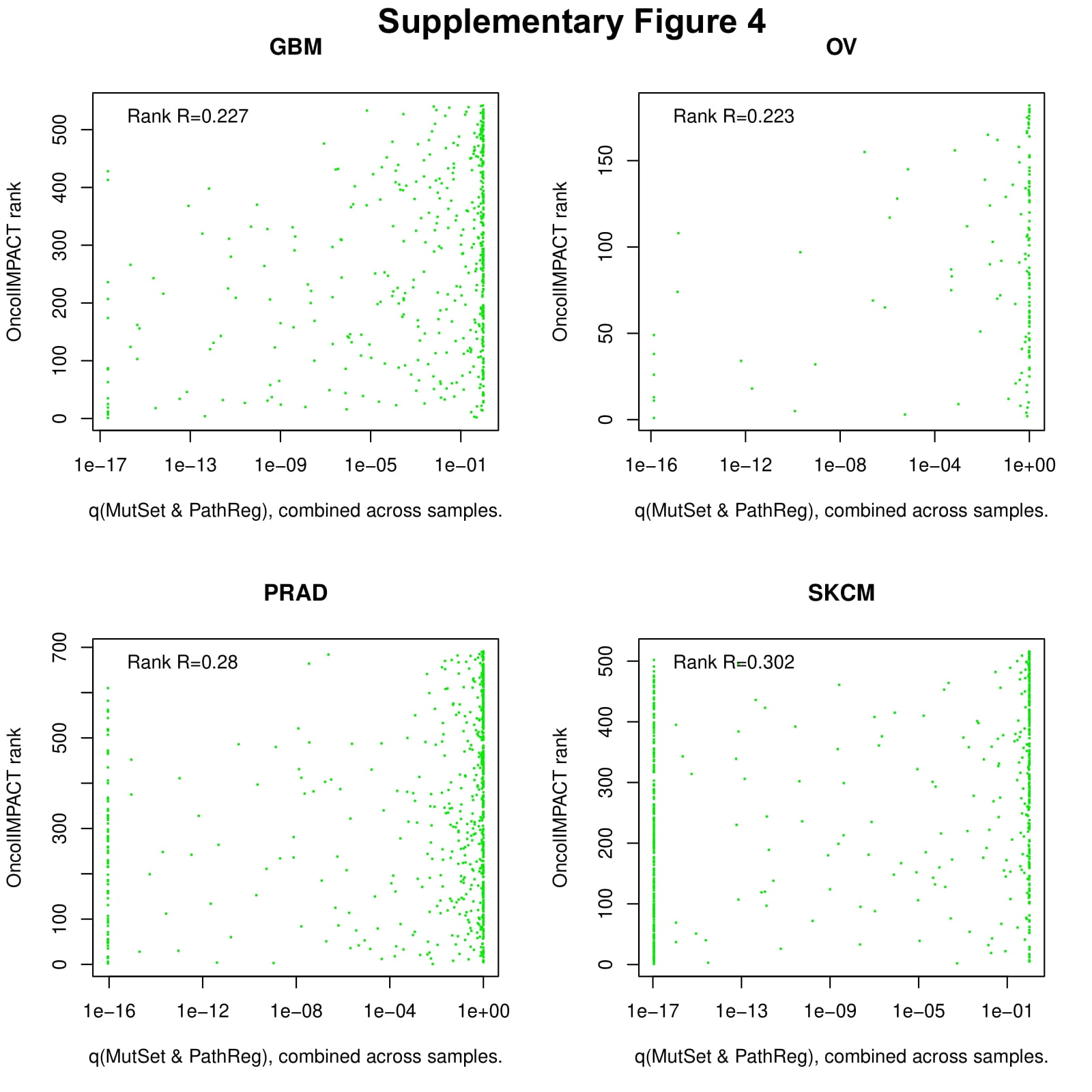

Supplement: Supplementary file 3. [file elife-74010-supp3.zip › SupplementaryFile3.jpg]

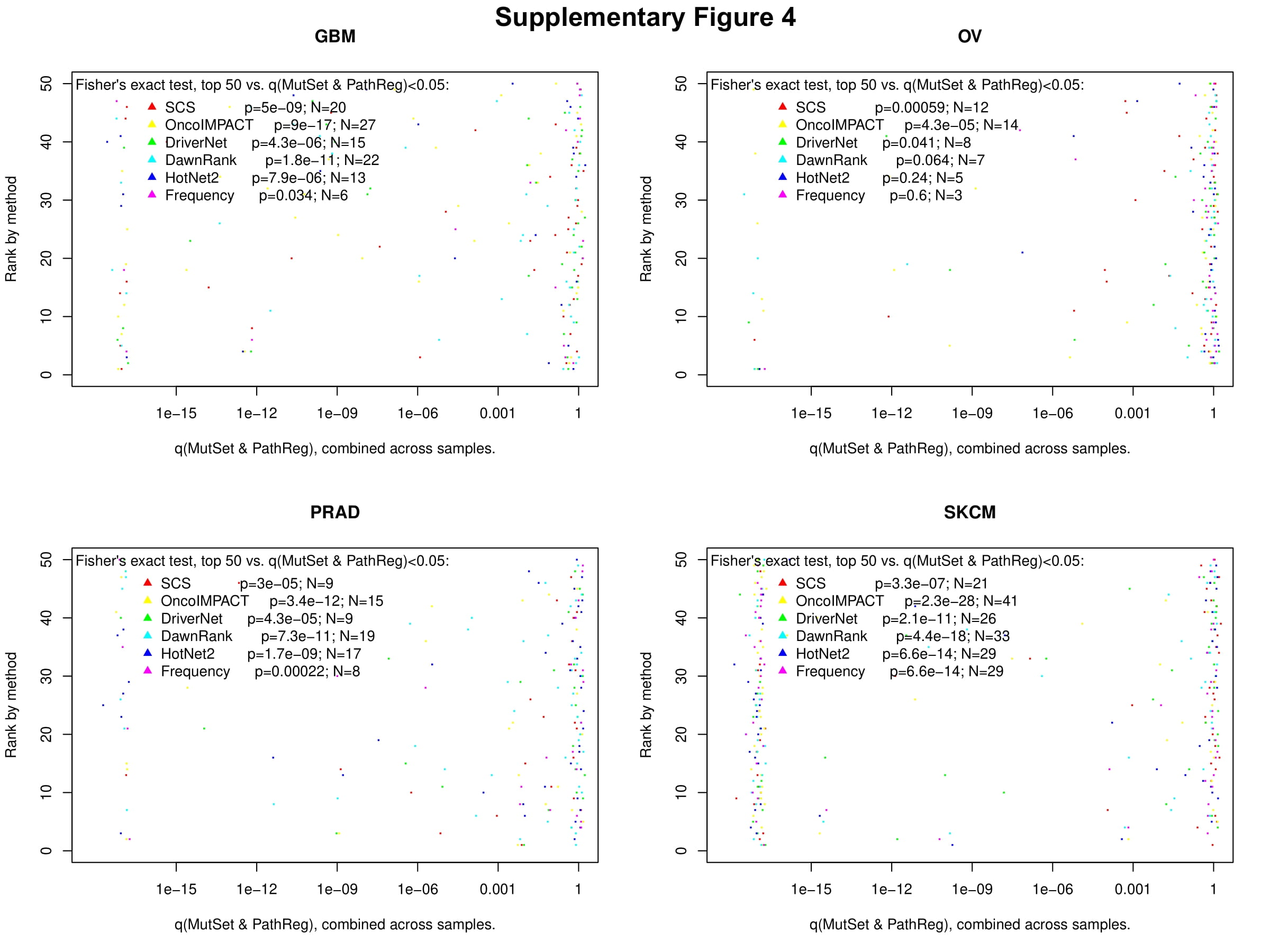

Supplement: Supplementary file 4. [file elife-74010-supp4.zip › SupplementaryFile4.jpg]

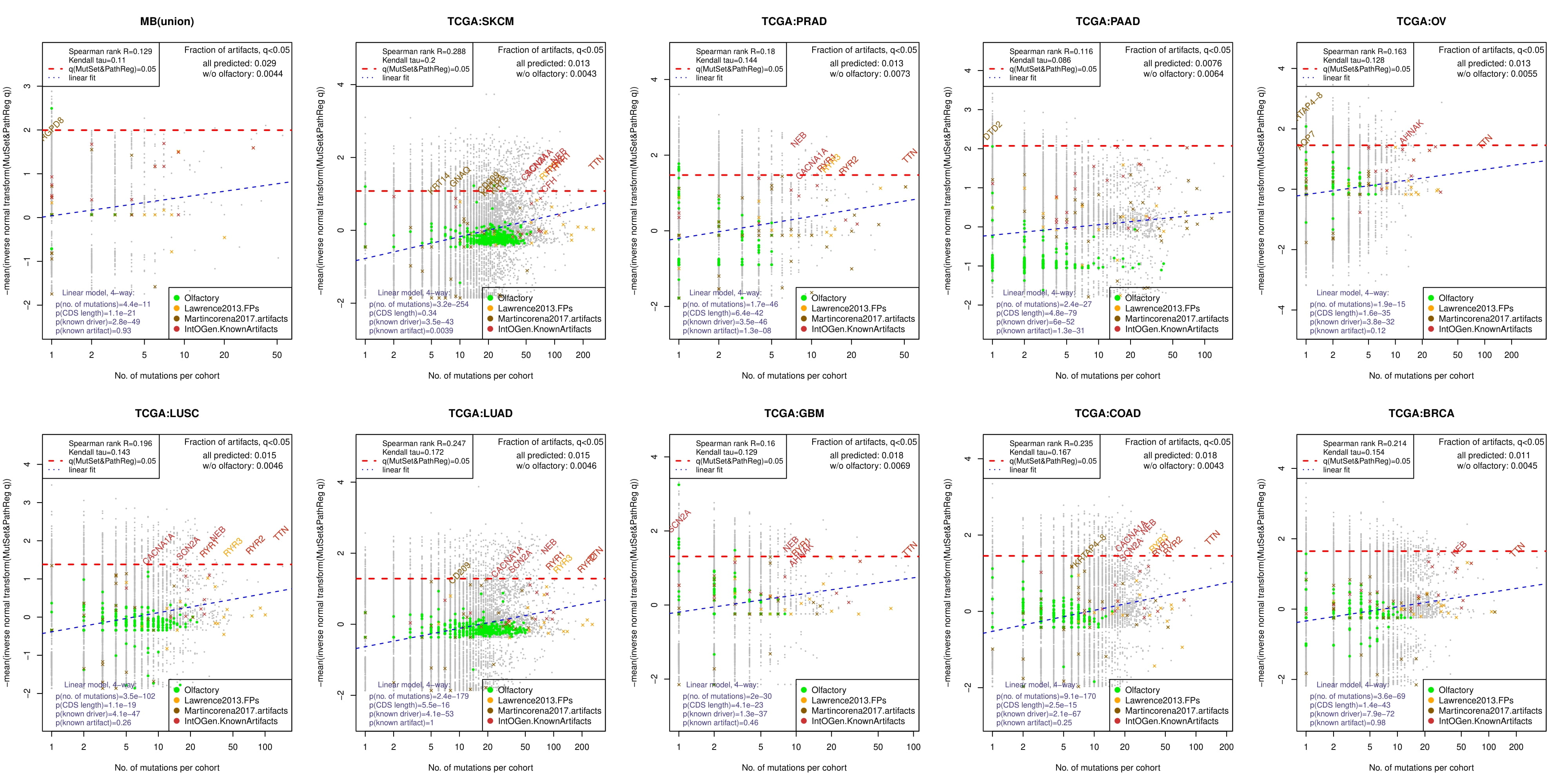

Supplement: Supplementary file 5. — The five pages present relations between MutSet&PathReg q (Y axis) and no. of mutations per cohort, gene length, and normalized mutation frequency, replication rate, and gene expression rates, respectively (X axis). Top left legend: Spearman rank R and Kendall tau represent overall correlations between X and Y coordinates regardless of other factors. Bottom left legend: terms’ significance in 4-way linear models. Colored points: genes suggested as potential artifacts in literature; those receiving q<0.05 are text-labeled. [file elife-74010-supp5.zip › SupplementaryFigure5-1.jpg]

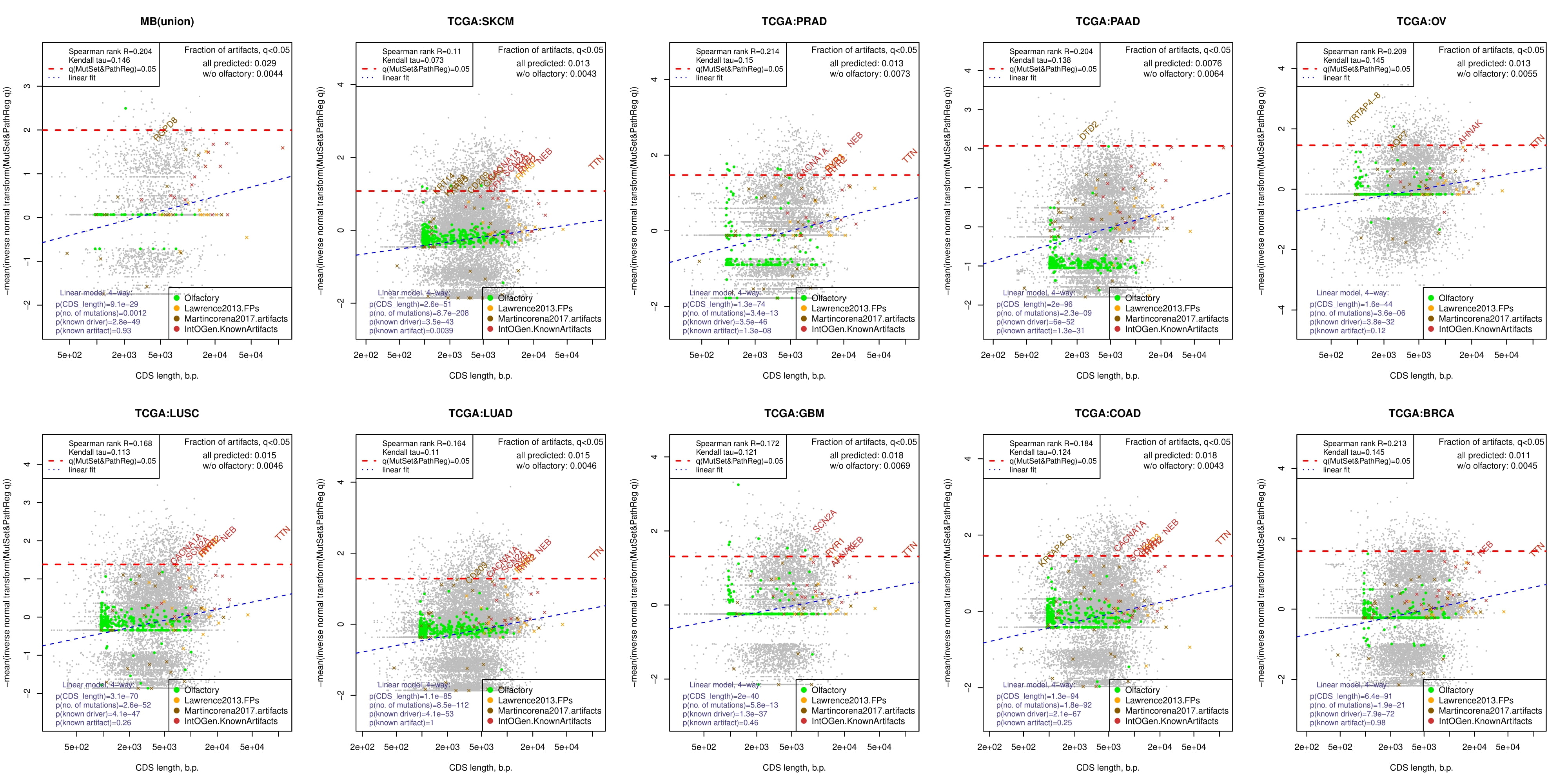

Supplement: Supplementary file 5. — The five pages present relations between MutSet&PathReg q (Y axis) and no. of mutations per cohort, gene length, and normalized mutation frequency, replication rate, and gene expression rates, respectively (X axis). Top left legend: Spearman rank R and Kendall tau represent overall correlations between X and Y coordinates regardless of other factors. Bottom left legend: terms’ significance in 4-way linear models. Colored points: genes suggested as potential artifacts in literature; those receiving q<0.05 are text-labeled. [file elife-74010-supp5.zip › SupplementaryFigure5-2.jpg]

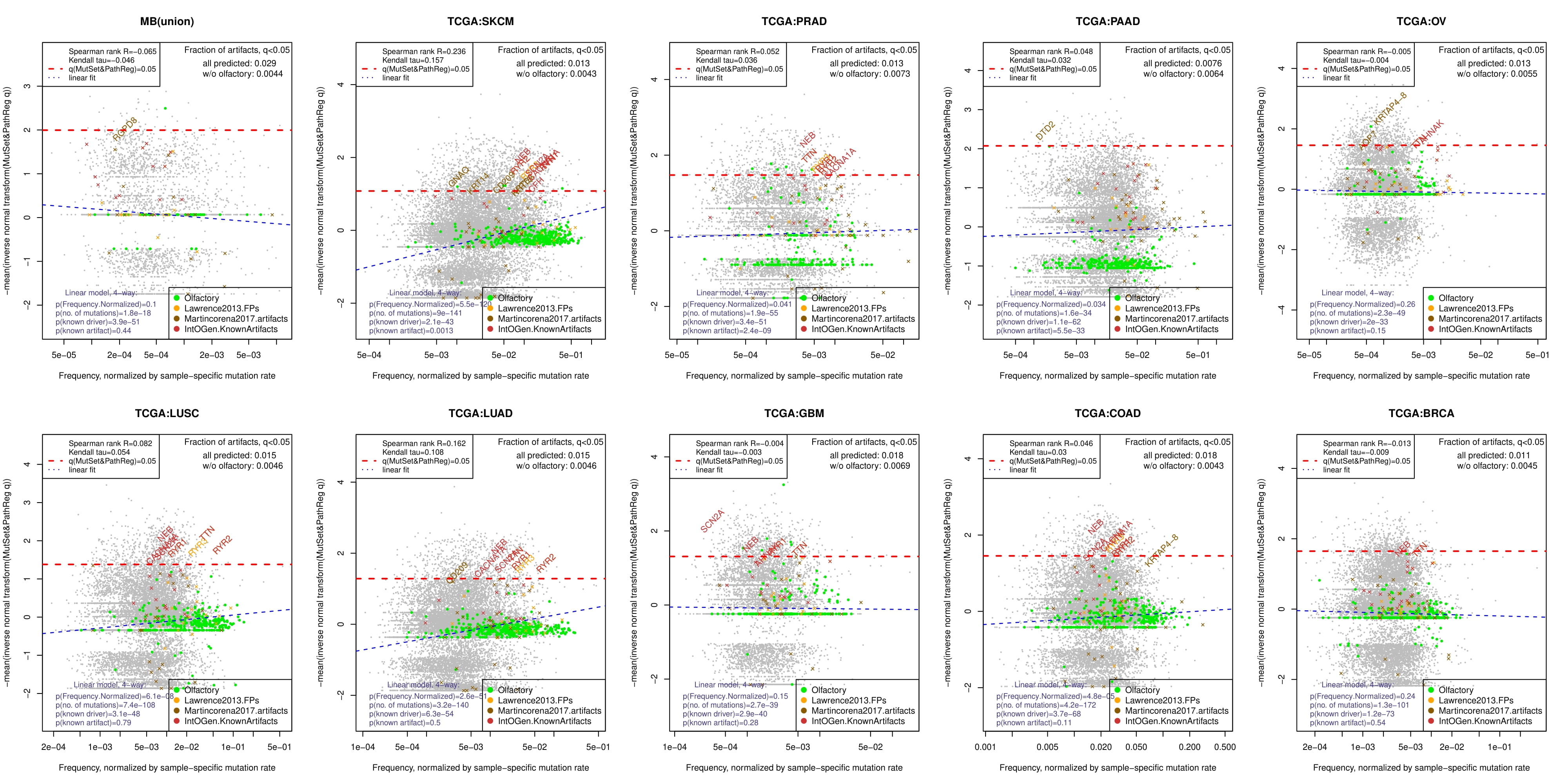

Supplement: Supplementary file 5. — The five pages present relations between MutSet&PathReg q (Y axis) and no. of mutations per cohort, gene length, and normalized mutation frequency, replication rate, and gene expression rates, respectively (X axis). Top left legend: Spearman rank R and Kendall tau represent overall correlations between X and Y coordinates regardless of other factors. Bottom left legend: terms’ significance in 4-way linear models. Colored points: genes suggested as potential artifacts in literature; those receiving q<0.05 are text-labeled. [file elife-74010-supp5.zip › SupplementaryFigure5-3.jpg]

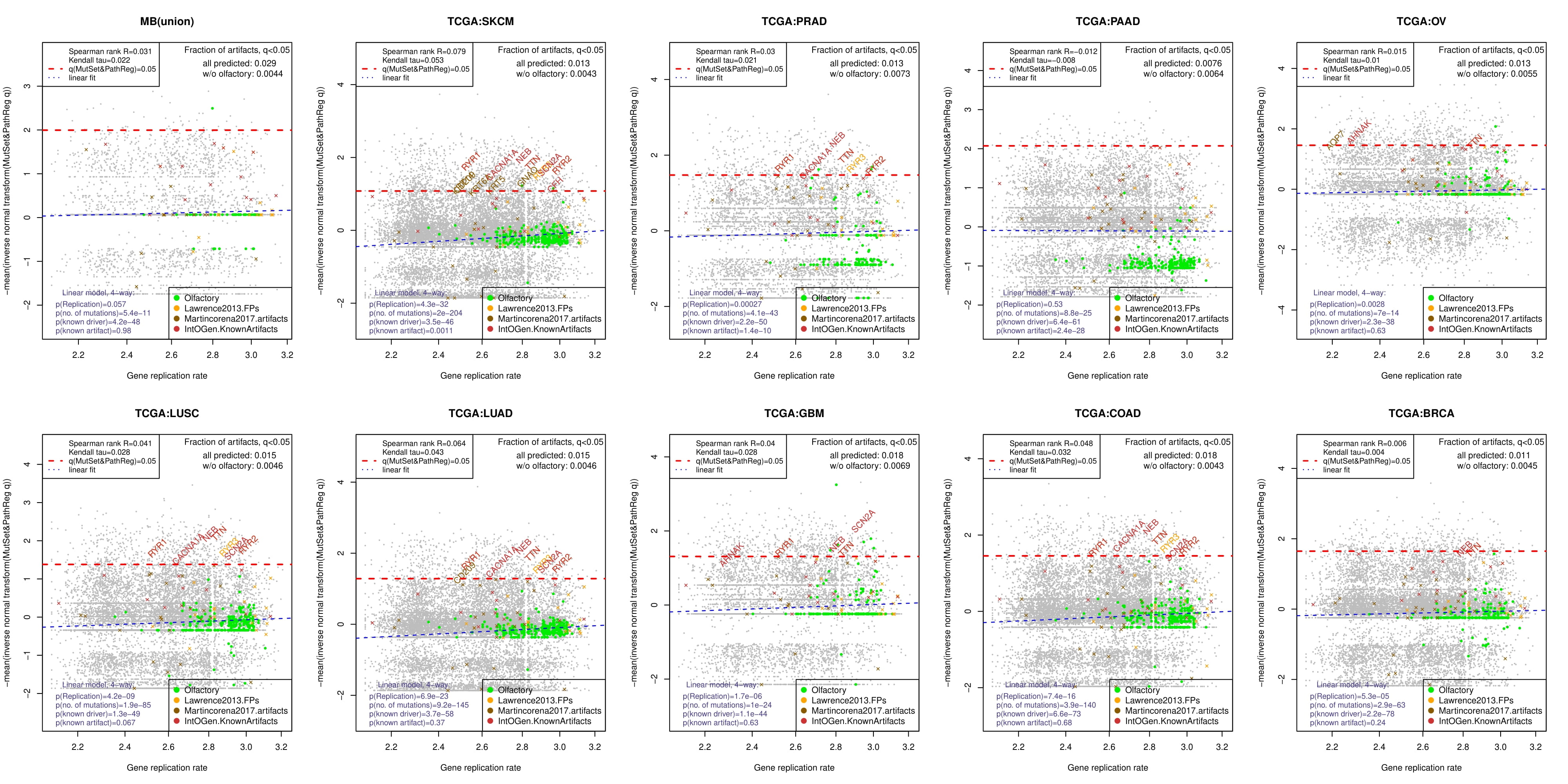

Supplement: Supplementary file 5. — The five pages present relations between MutSet&PathReg q (Y axis) and no. of mutations per cohort, gene length, and normalized mutation frequency, replication rate, and gene expression rates, respectively (X axis). Top left legend: Spearman rank R and Kendall tau represent overall correlations between X and Y coordinates regardless of other factors. Bottom left legend: terms’ significance in 4-way linear models. Colored points: genes suggested as potential artifacts in literature; those receiving q<0.05 are text-labeled. [file elife-74010-supp5.zip › SupplementaryFigure5-4.jpg]

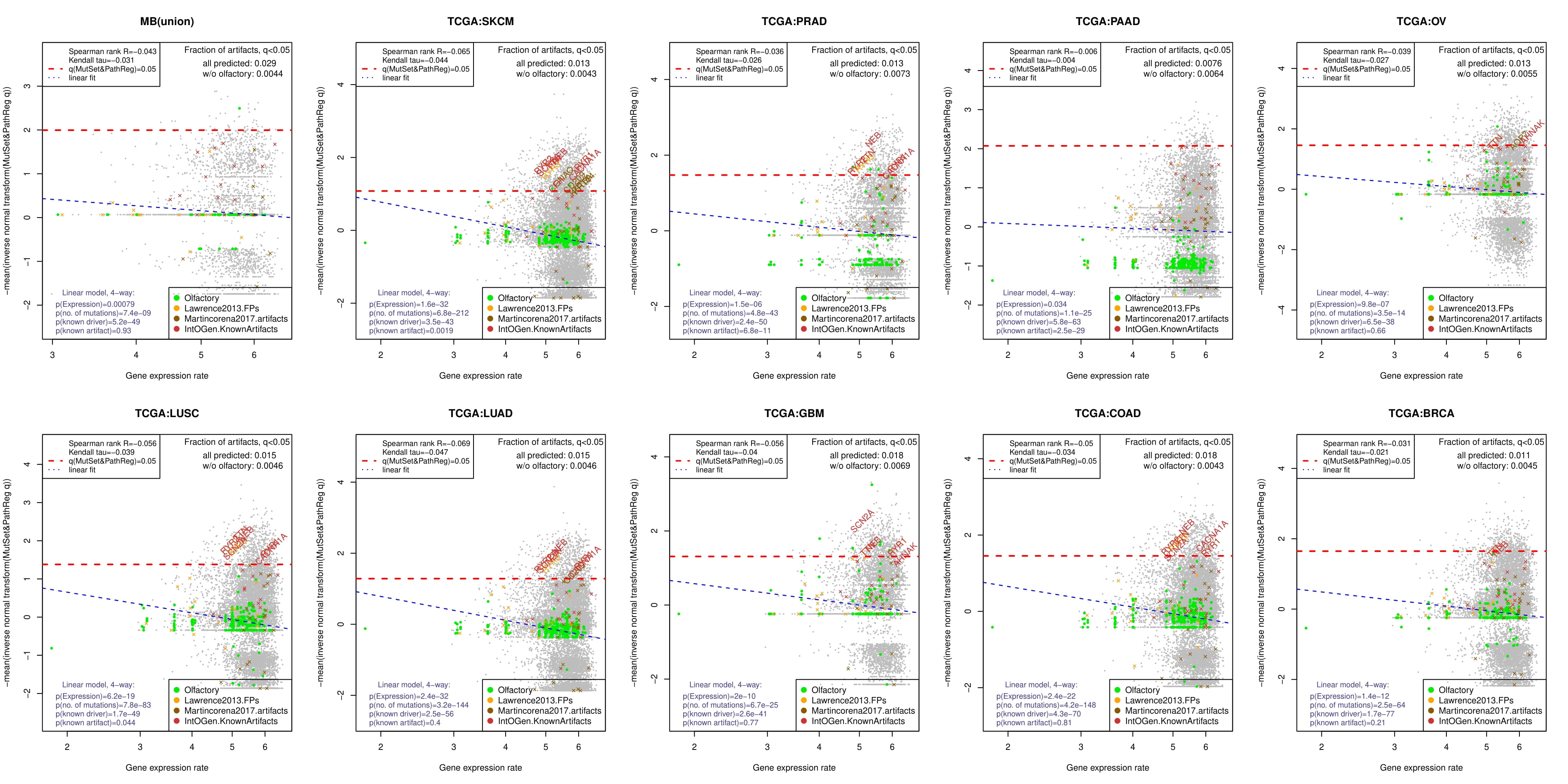

Supplement: Supplementary file 5. — The five pages present relations between MutSet&PathReg q (Y axis) and no. of mutations per cohort, gene length, and normalized mutation frequency, replication rate, and gene expression rates, respectively (X axis). Top left legend: Spearman rank R and Kendall tau represent overall correlations between X and Y coordinates regardless of other factors. Bottom left legend: terms’ significance in 4-way linear models. Colored points: genes suggested as potential artifacts in literature; those receiving q<0.05 are text-labeled. [file elife-74010-supp5.zip › SupplementaryFigure5-5.jpg]

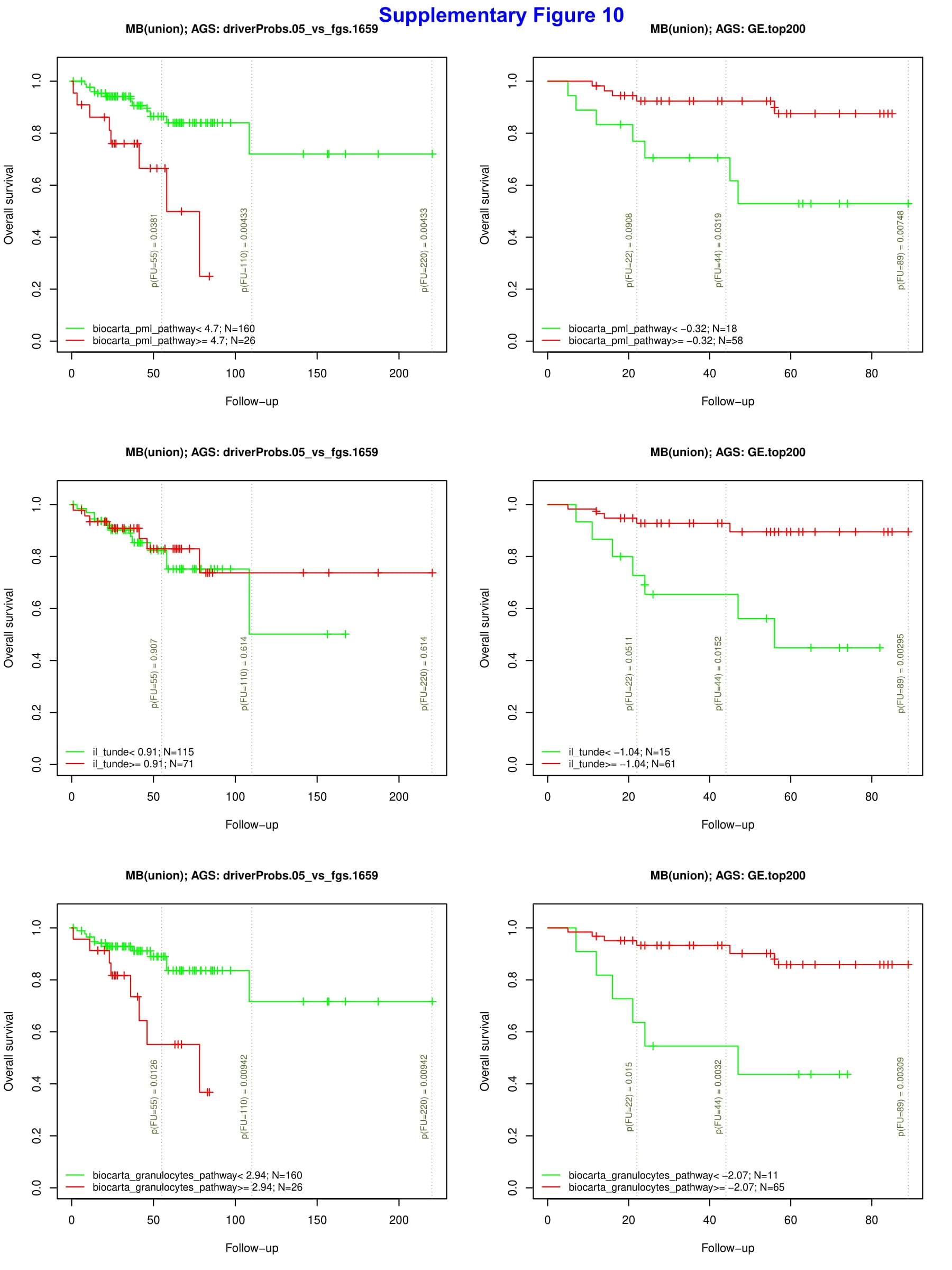

Supplement: Supplementary file 6. [file elife-74010-supp6.zip › SupplementaryFile6-01.jpg]

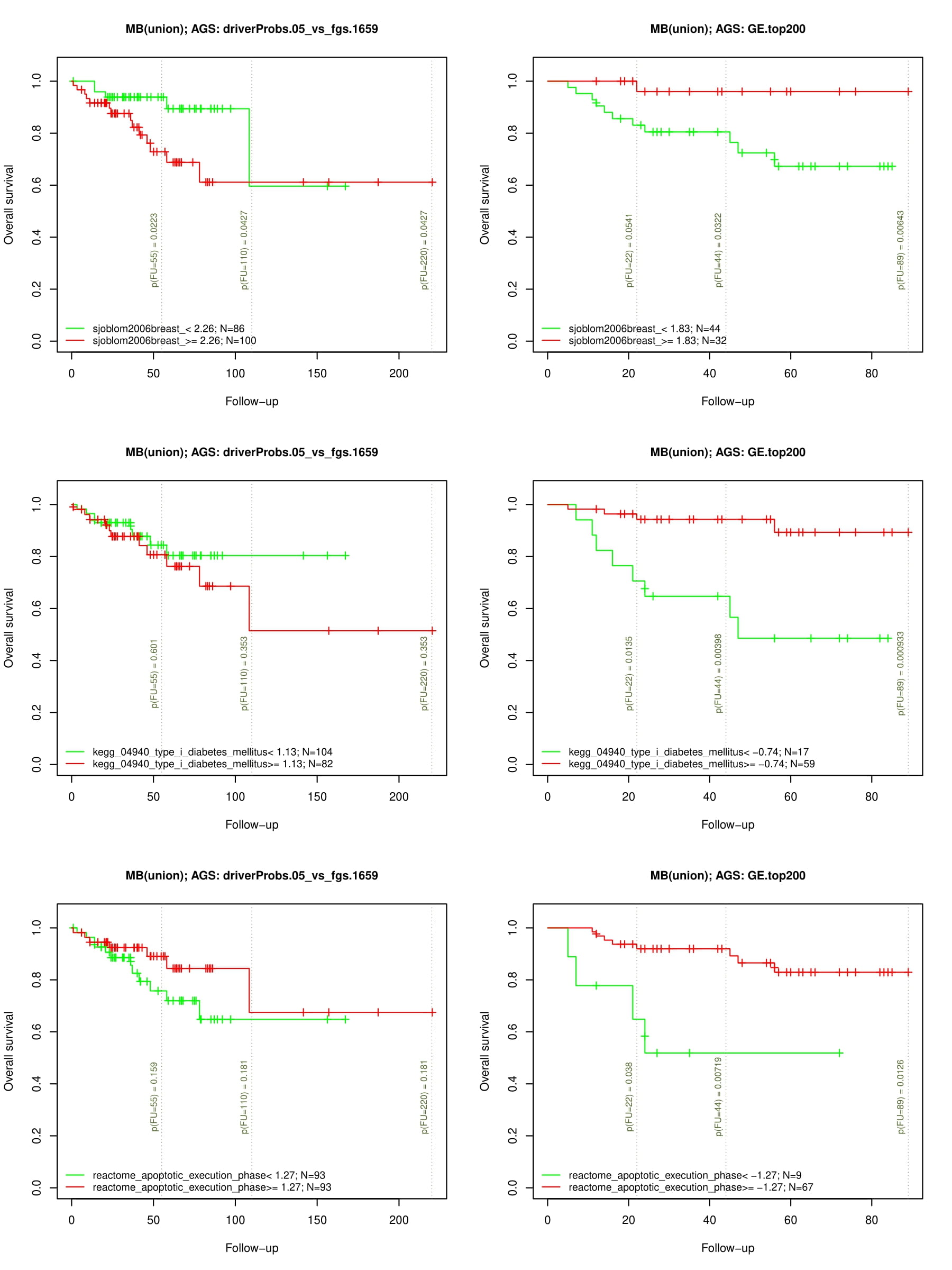

Supplement: Supplementary file 6. [file elife-74010-supp6.zip › SupplementaryFile6-02.jpg]

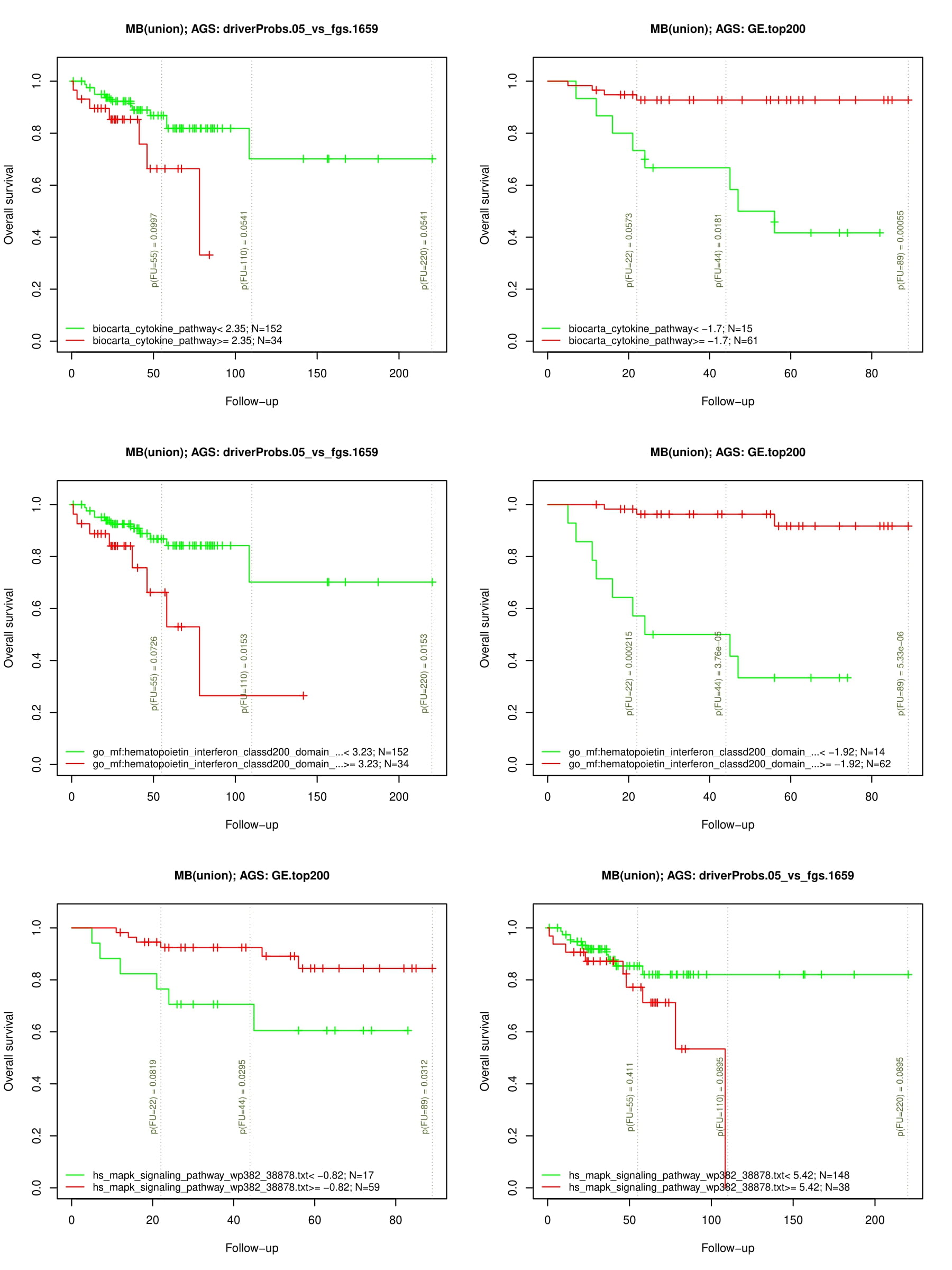

Supplement: Supplementary file 6. [file elife-74010-supp6.zip › SupplementaryFile6-03.jpg]

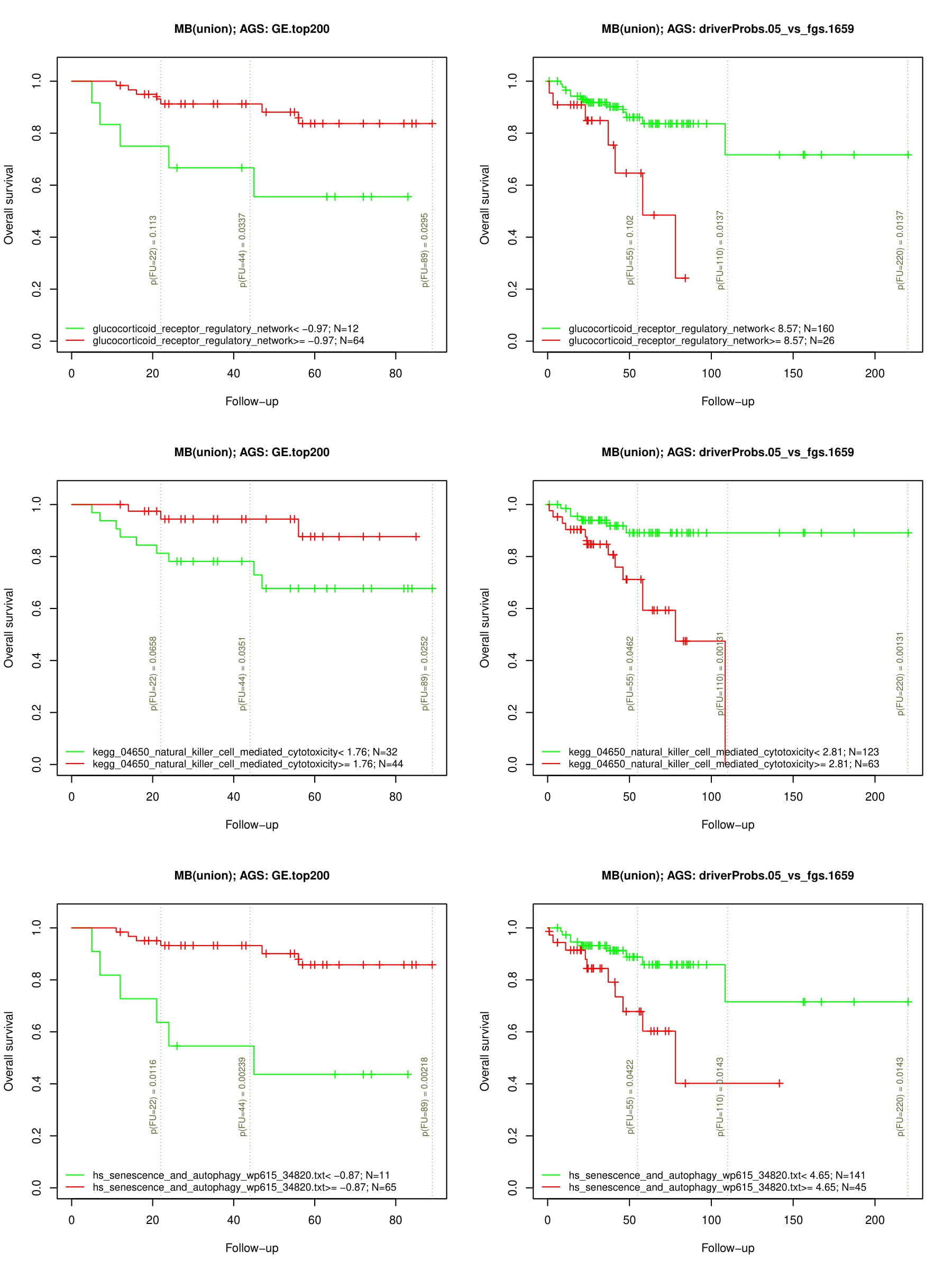

Supplement: Supplementary file 6. [file elife-74010-supp6.zip › SupplementaryFile6-04.jpg]

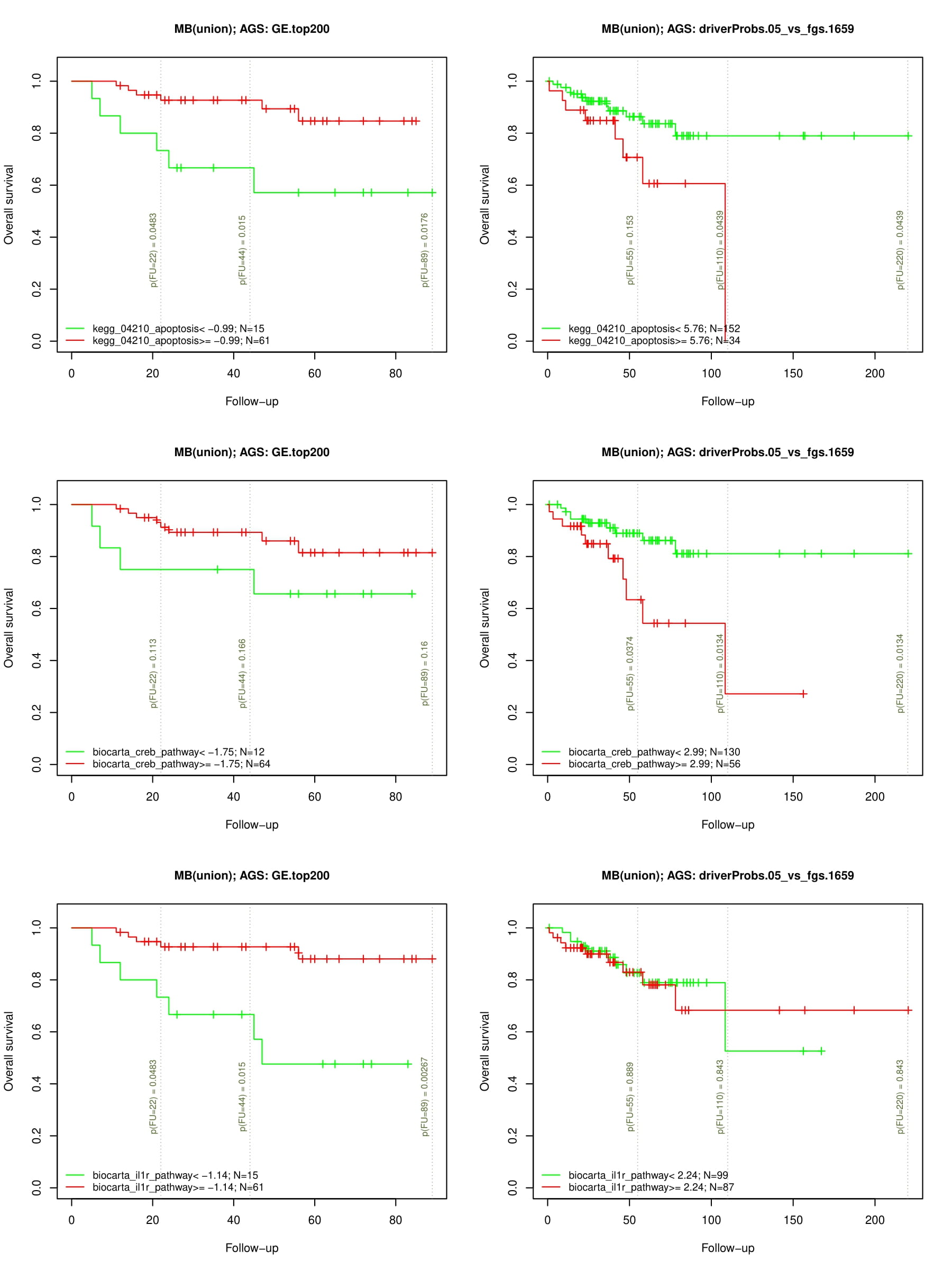

Supplement: Supplementary file 6. [file elife-74010-supp6.zip › SupplementaryFile6-05.jpg]

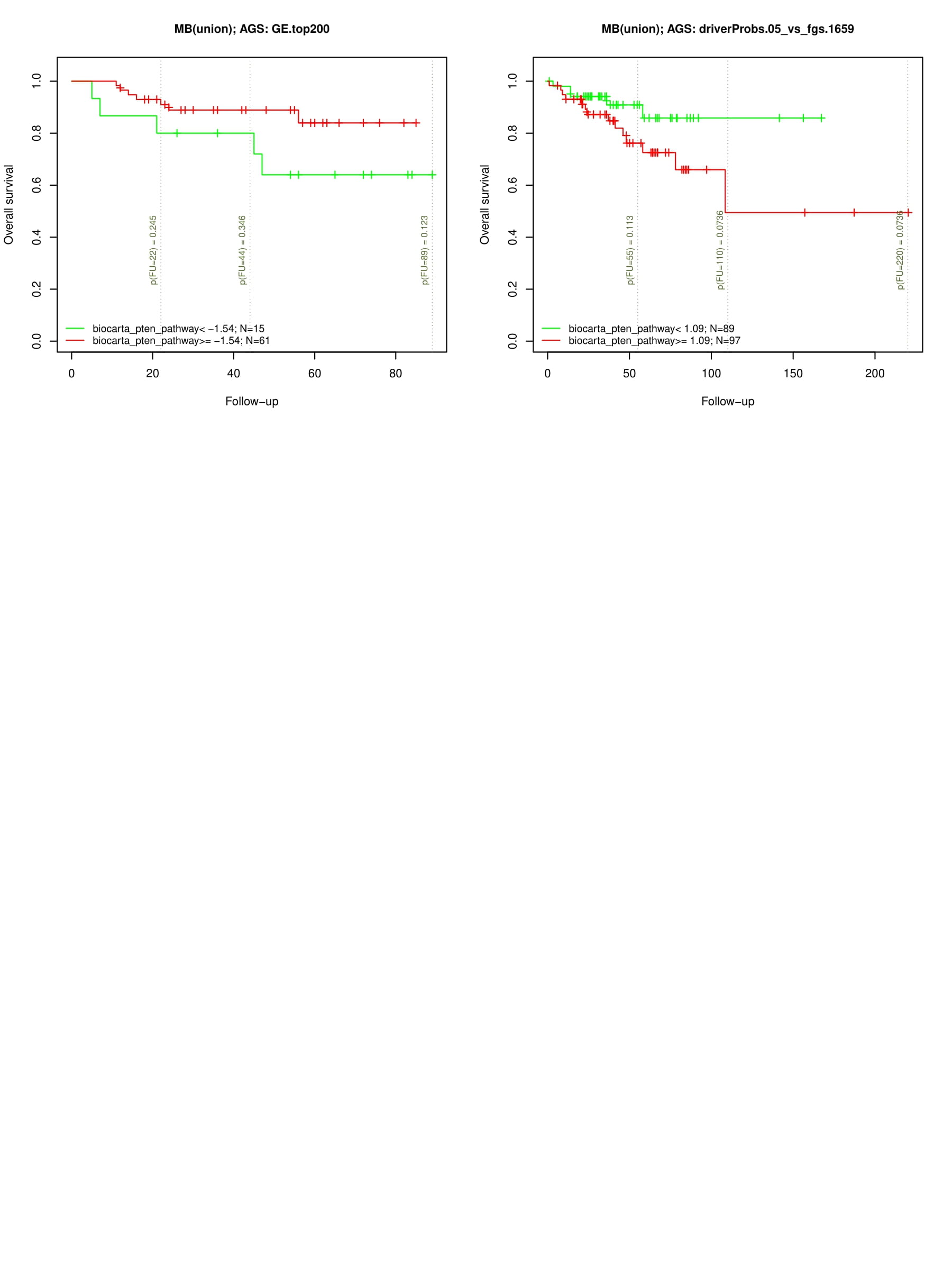

Supplement: Supplementary file 6. [file elife-74010-supp6.zip › SupplementaryFile6-06.jpg]

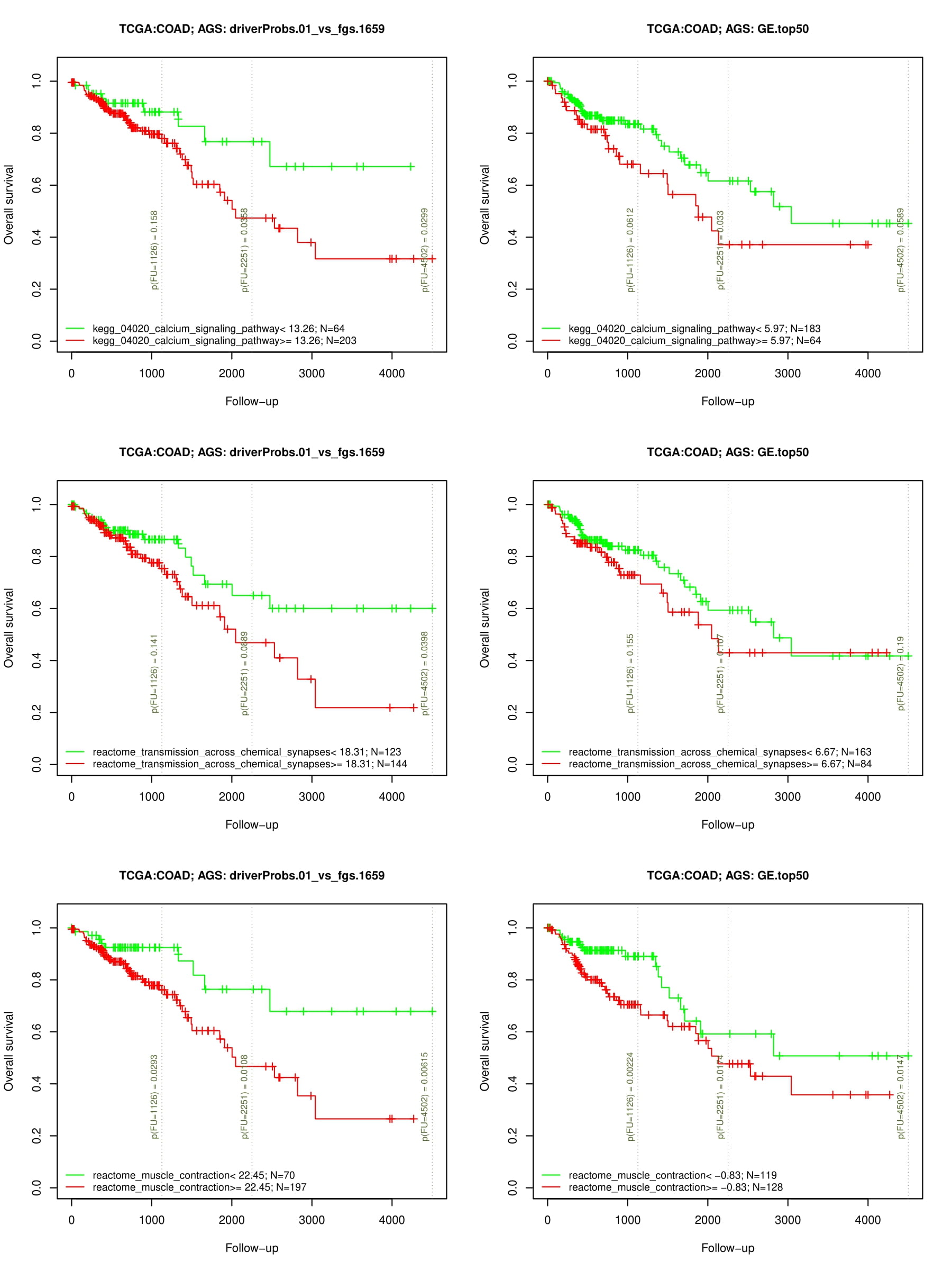

Supplement: Supplementary file 6. [file elife-74010-supp6.zip › SupplementaryFile6-07.jpg]

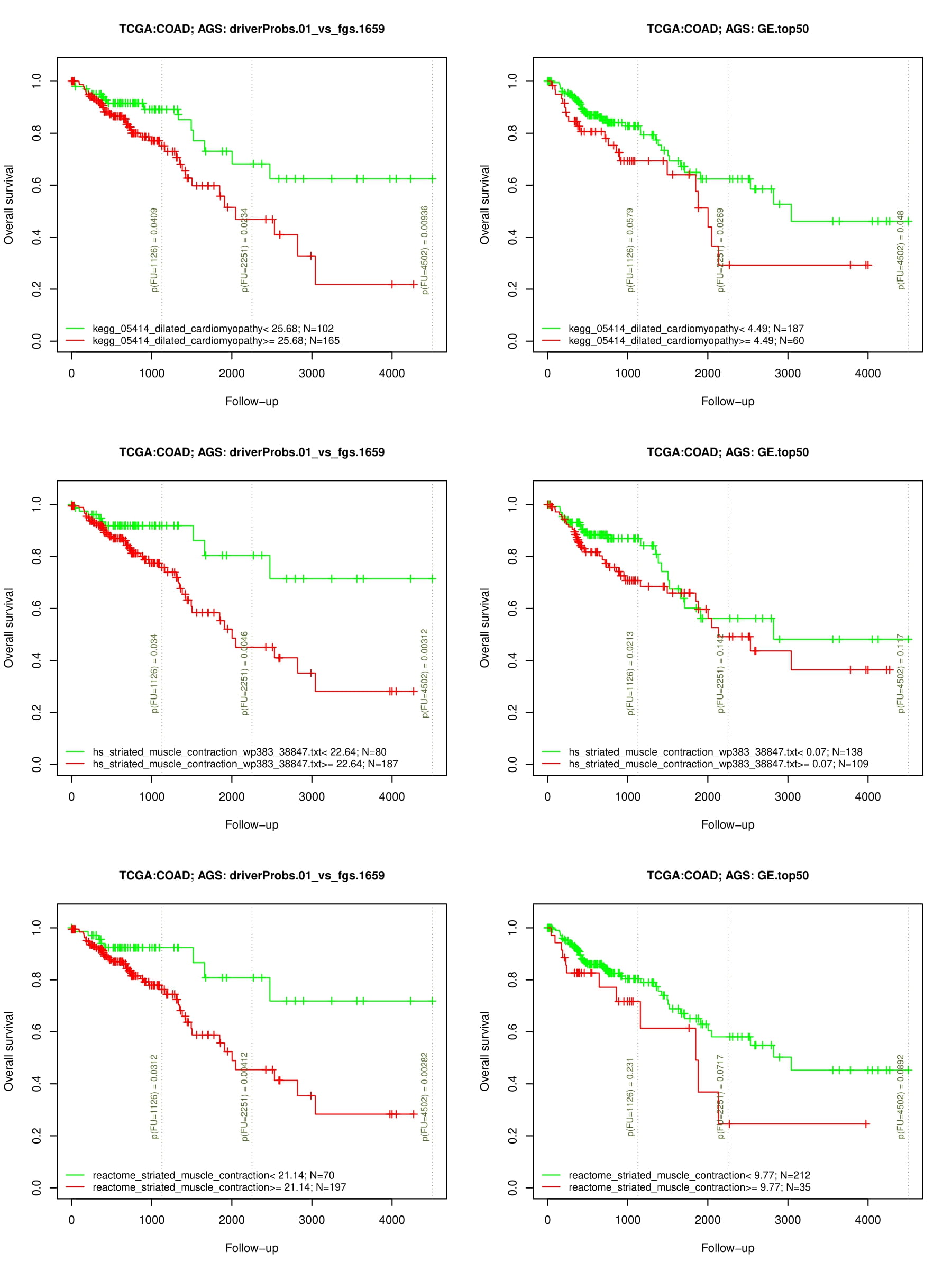

Supplement: Supplementary file 6. [file elife-74010-supp6.zip › SupplementaryFile6-08.jpg]

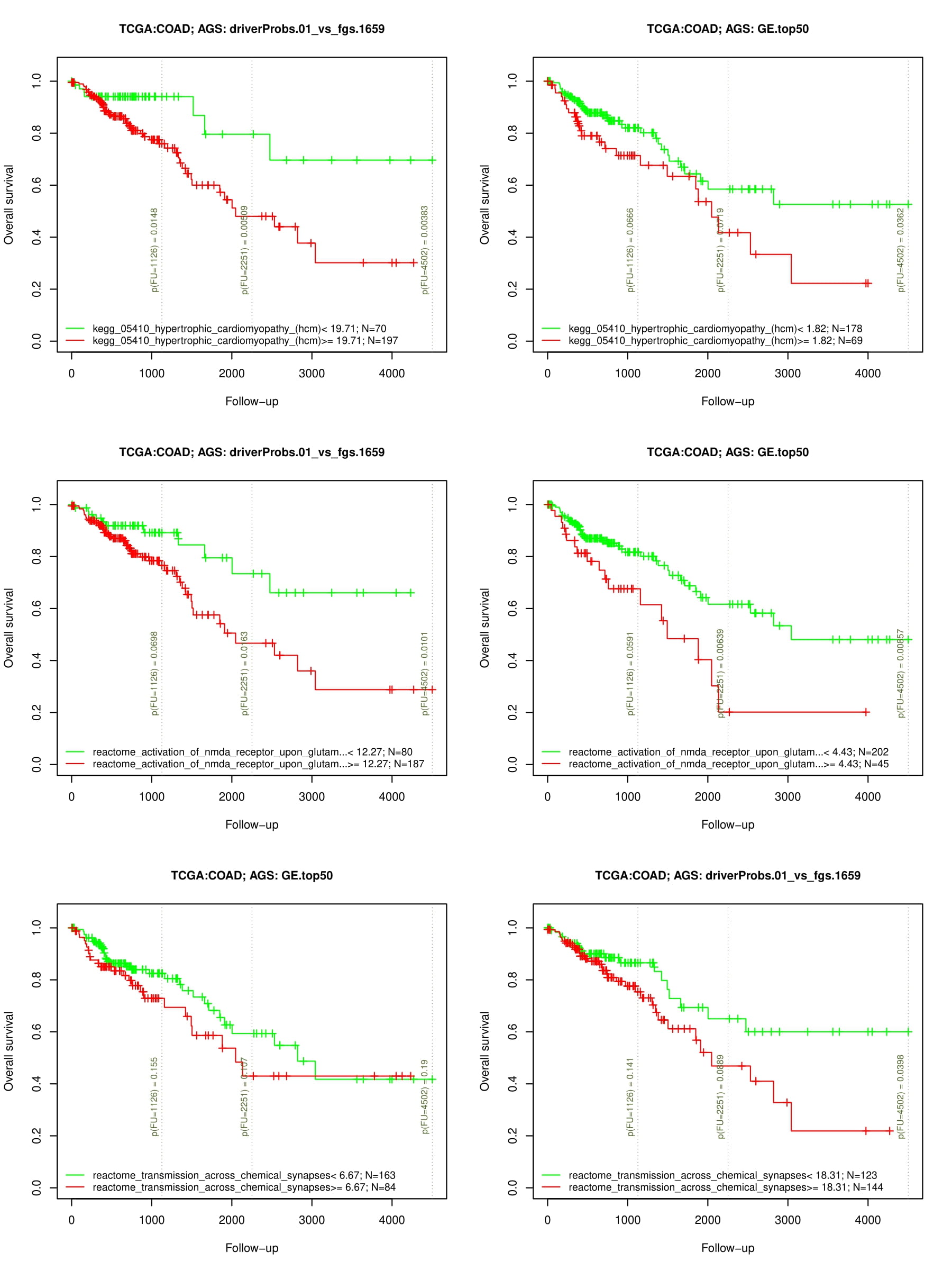

Supplement: Supplementary file 6. [file elife-74010-supp6.zip › SupplementaryFile6-09.jpg]

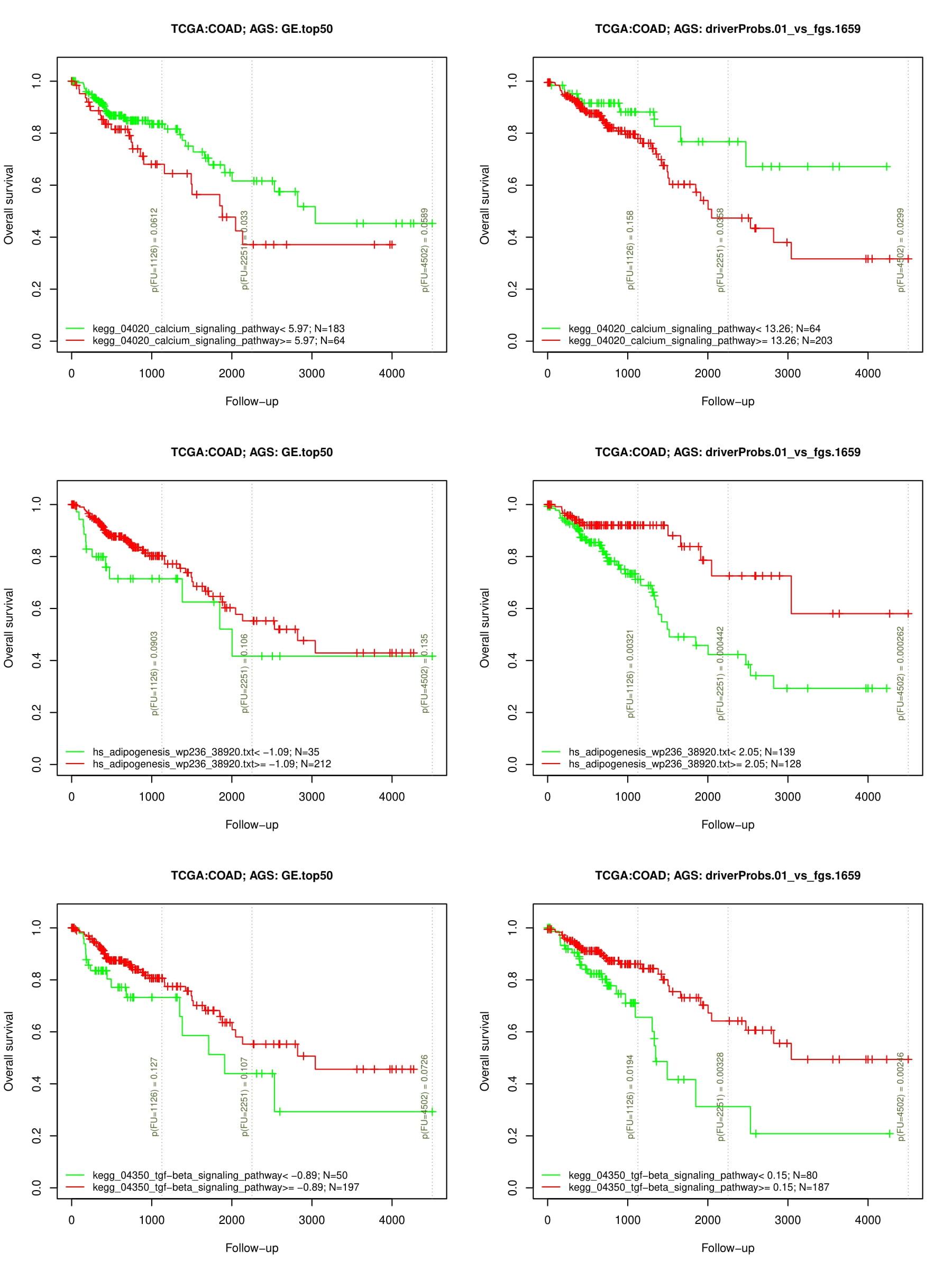

Supplement: Supplementary file 6. [file elife-74010-supp6.zip › SupplementaryFile6-10.jpg]

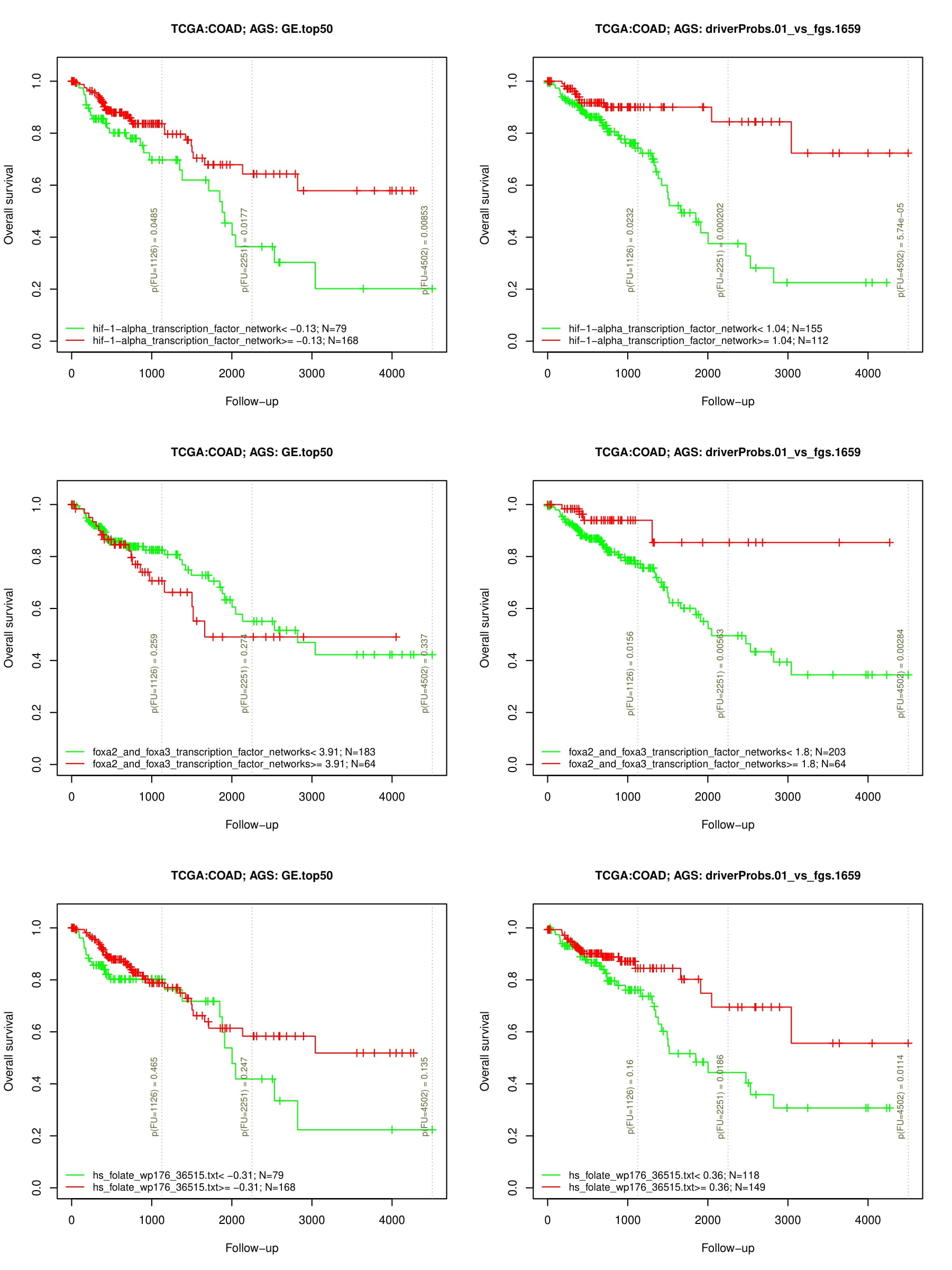

Supplement: Supplementary file 6. [file elife-74010-supp6.zip › SupplementaryFile6-11.jpg]

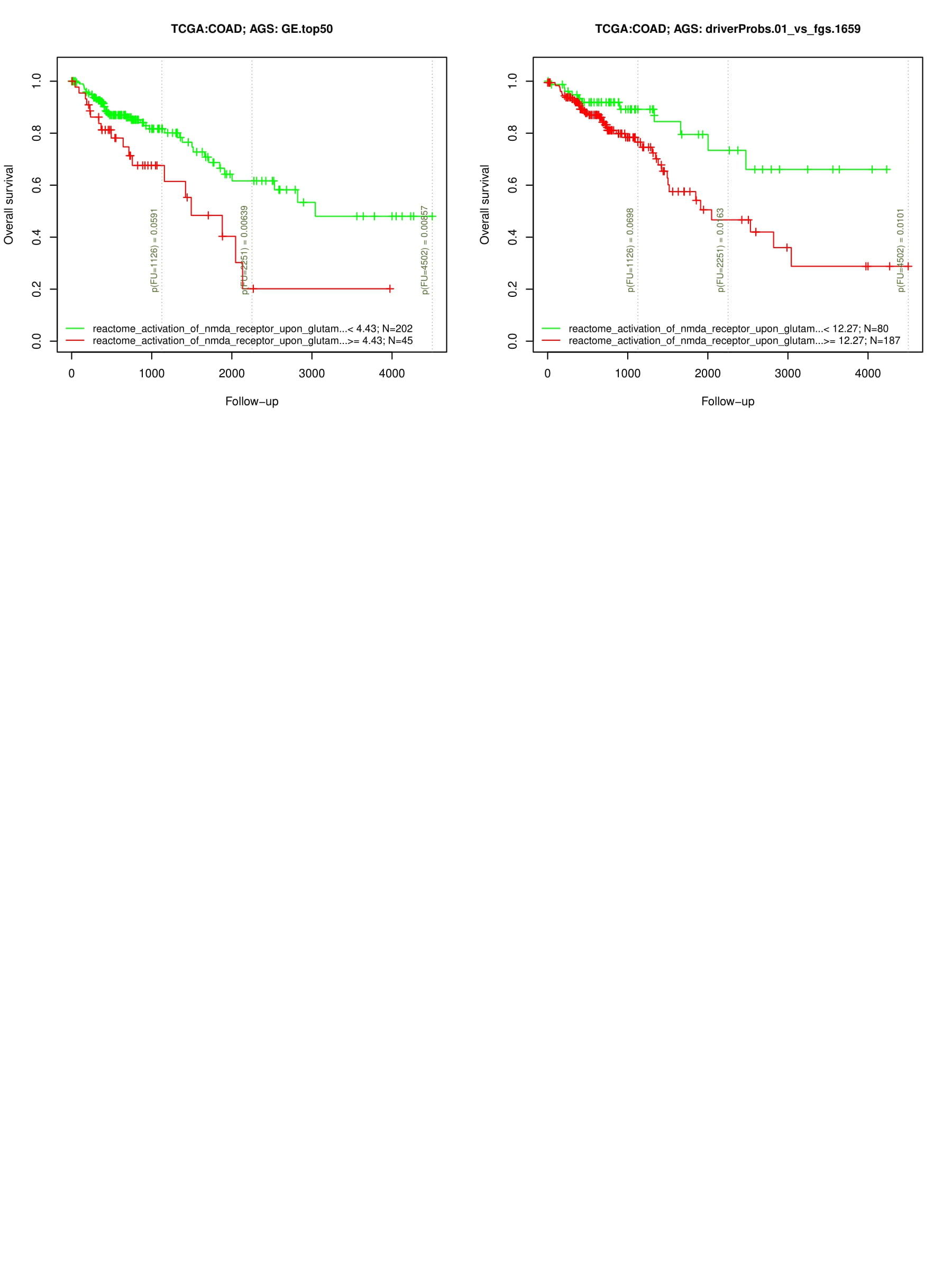

Supplement: Supplementary file 6. [file elife-74010-supp6.zip › SupplementaryFile6-12.jpg]

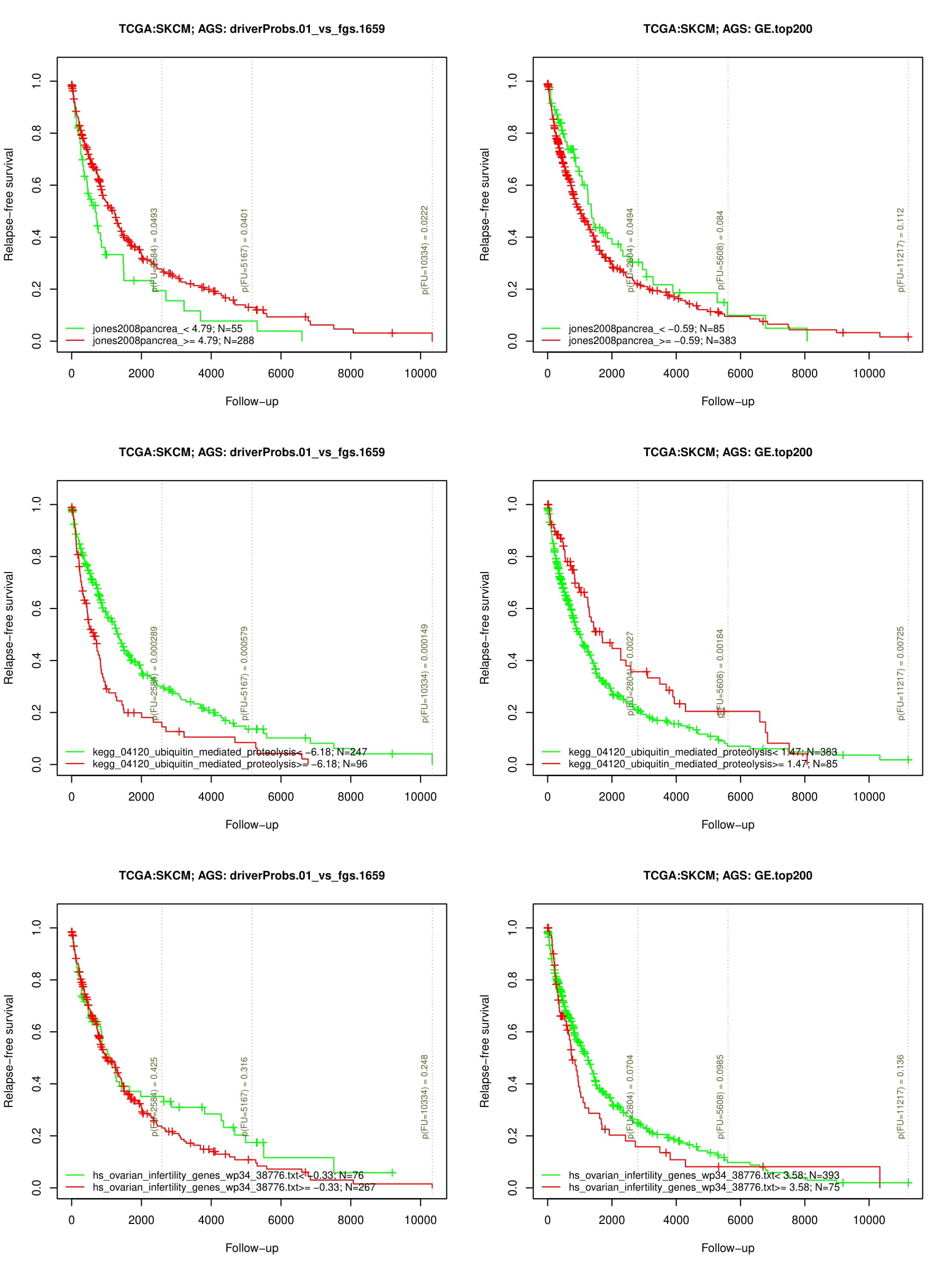

Supplement: Supplementary file 6. [file elife-74010-supp6.zip › SupplementaryFile6-13.jpg]

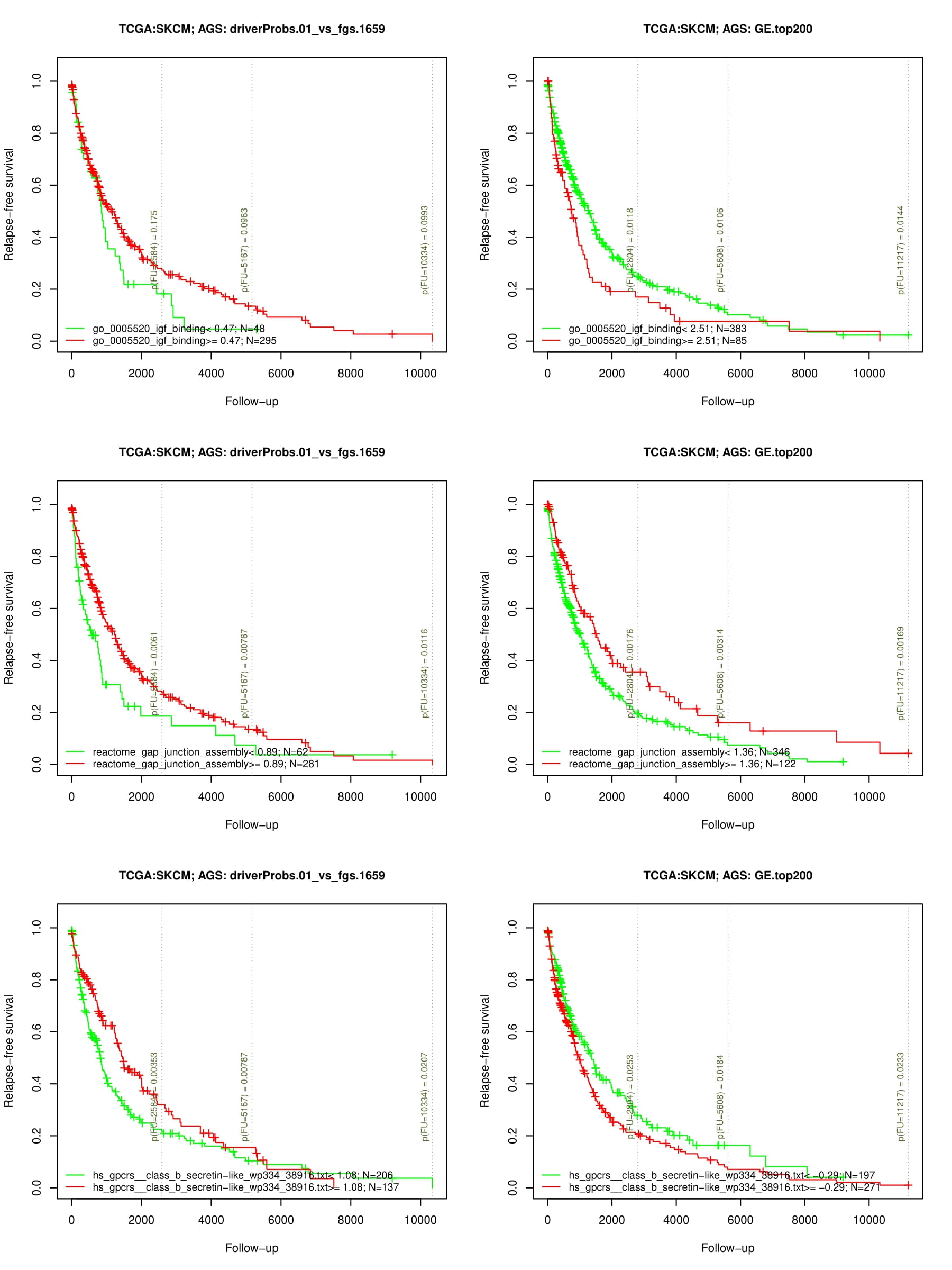

Supplement: Supplementary file 6. [file elife-74010-supp6.zip › SupplementaryFile6-14.jpg]

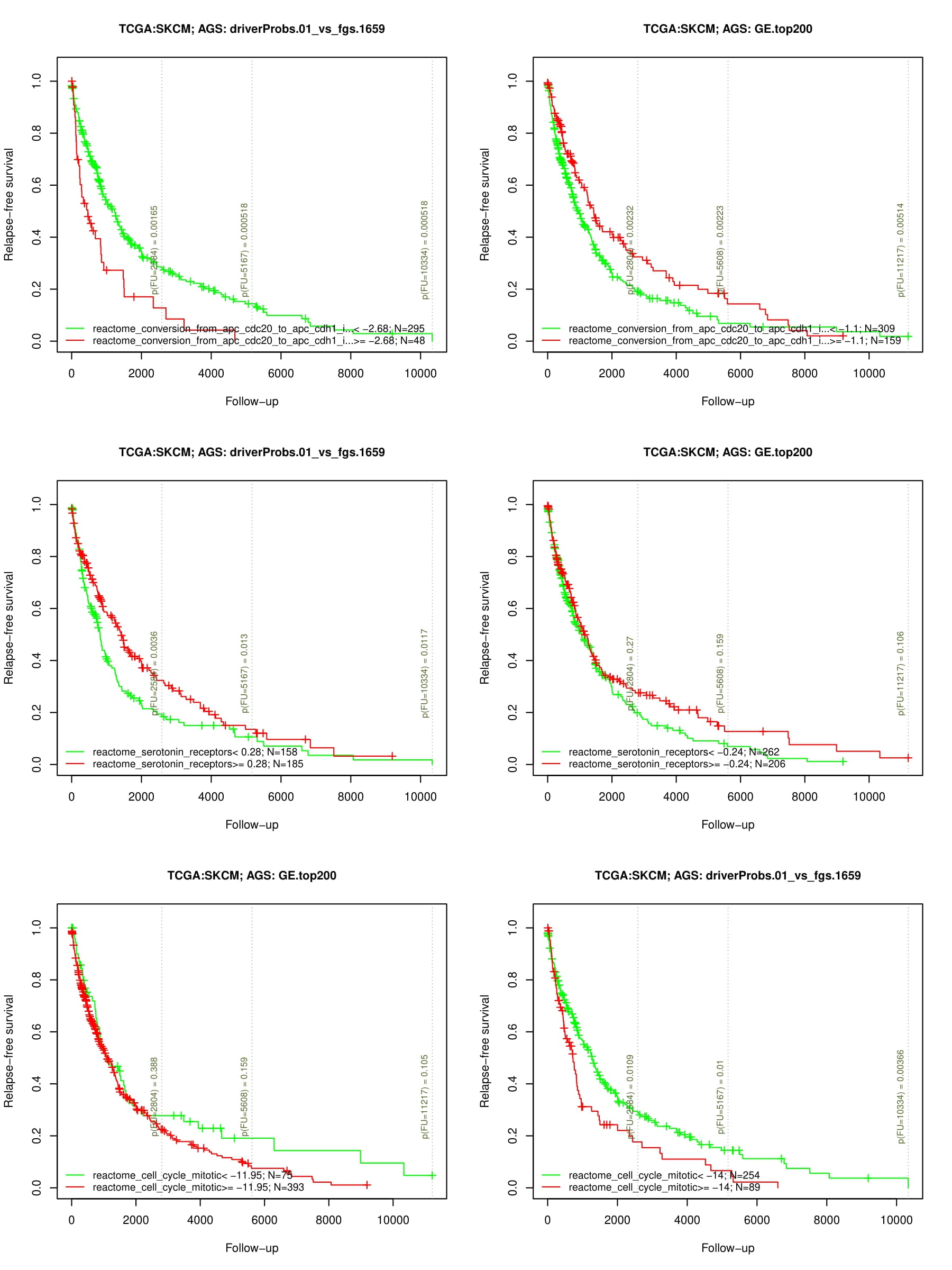

Supplement: Supplementary file 6. [file elife-74010-supp6.zip › SupplementaryFile6-15.jpg]

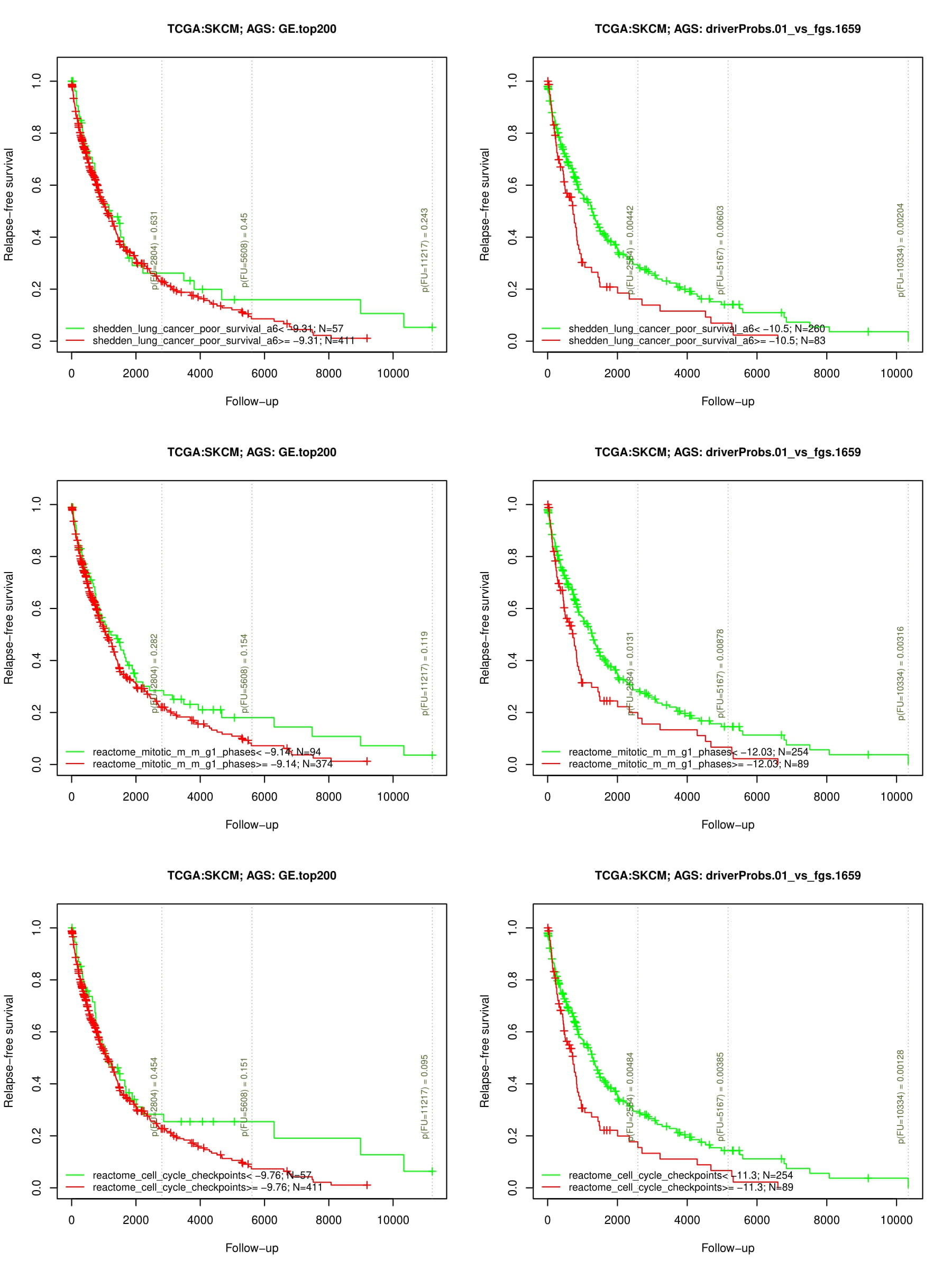

Supplement: Supplementary file 6. [file elife-74010-supp6.zip › SupplementaryFile6-16.jpg]

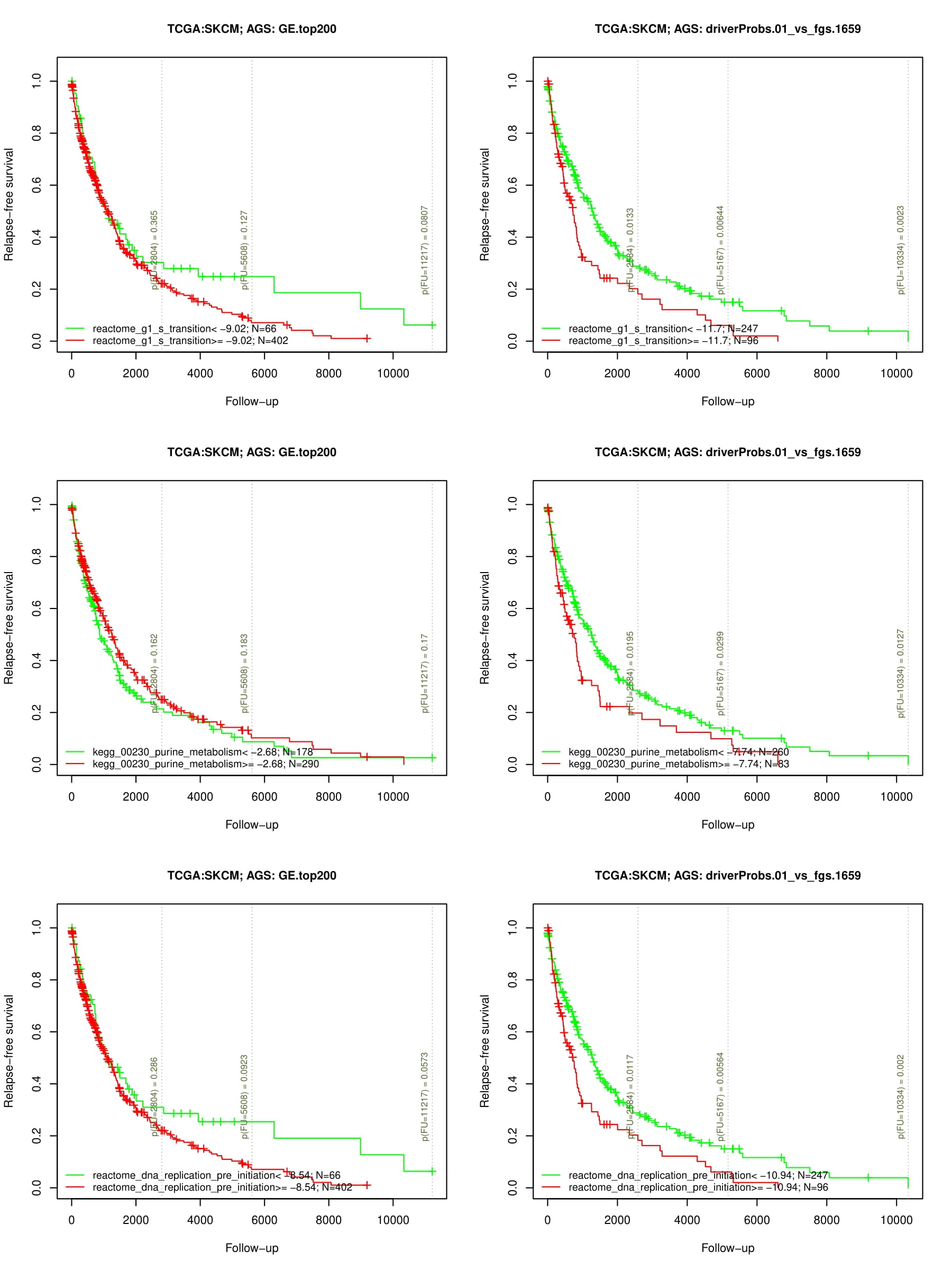

Supplement: Supplementary file 6. [file elife-74010-supp6.zip › SupplementaryFile6-17.jpg]

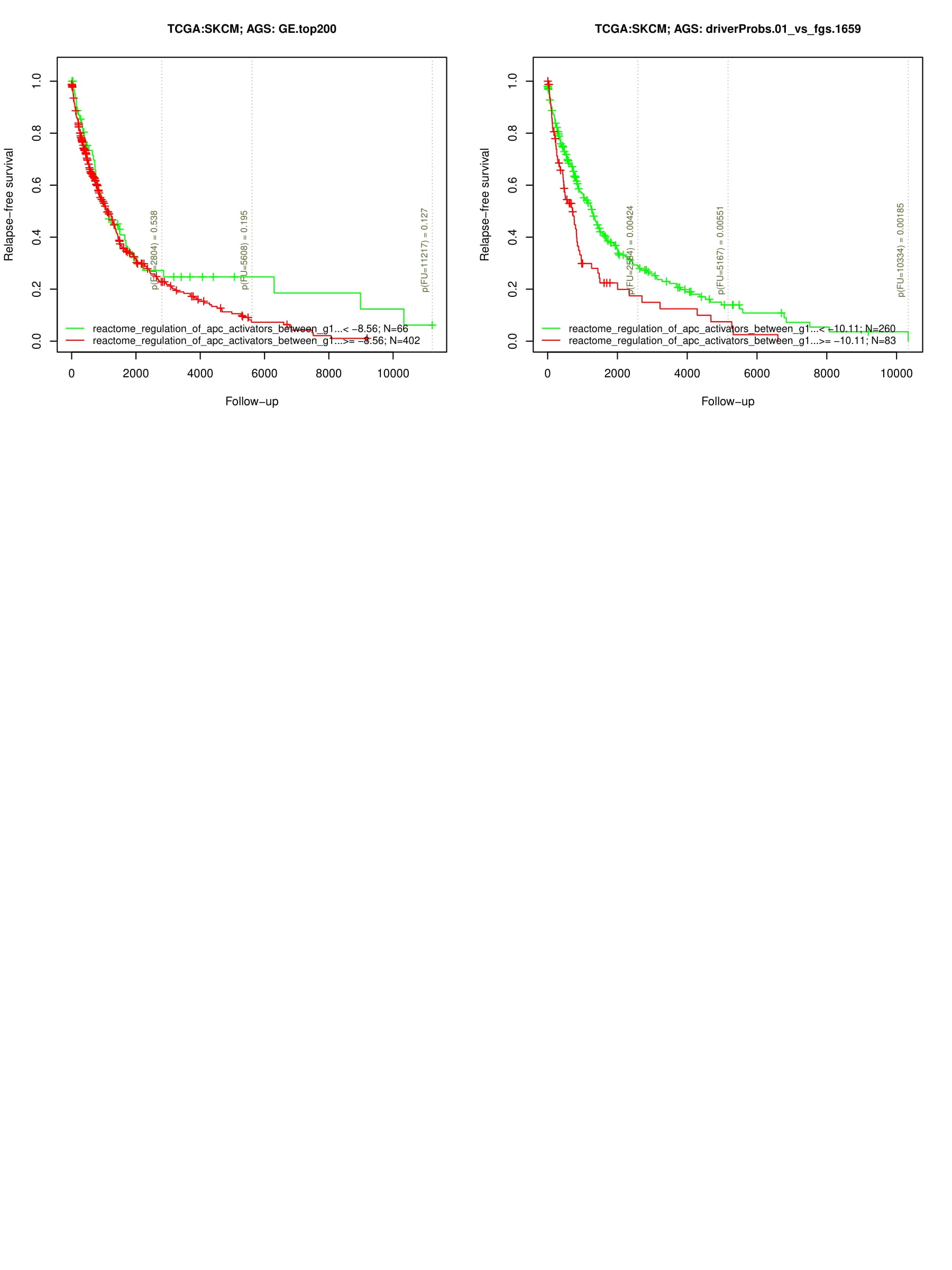

Supplement: Supplementary file 6. [file elife-74010-supp6.zip › SupplementaryFile6-18.jpg]

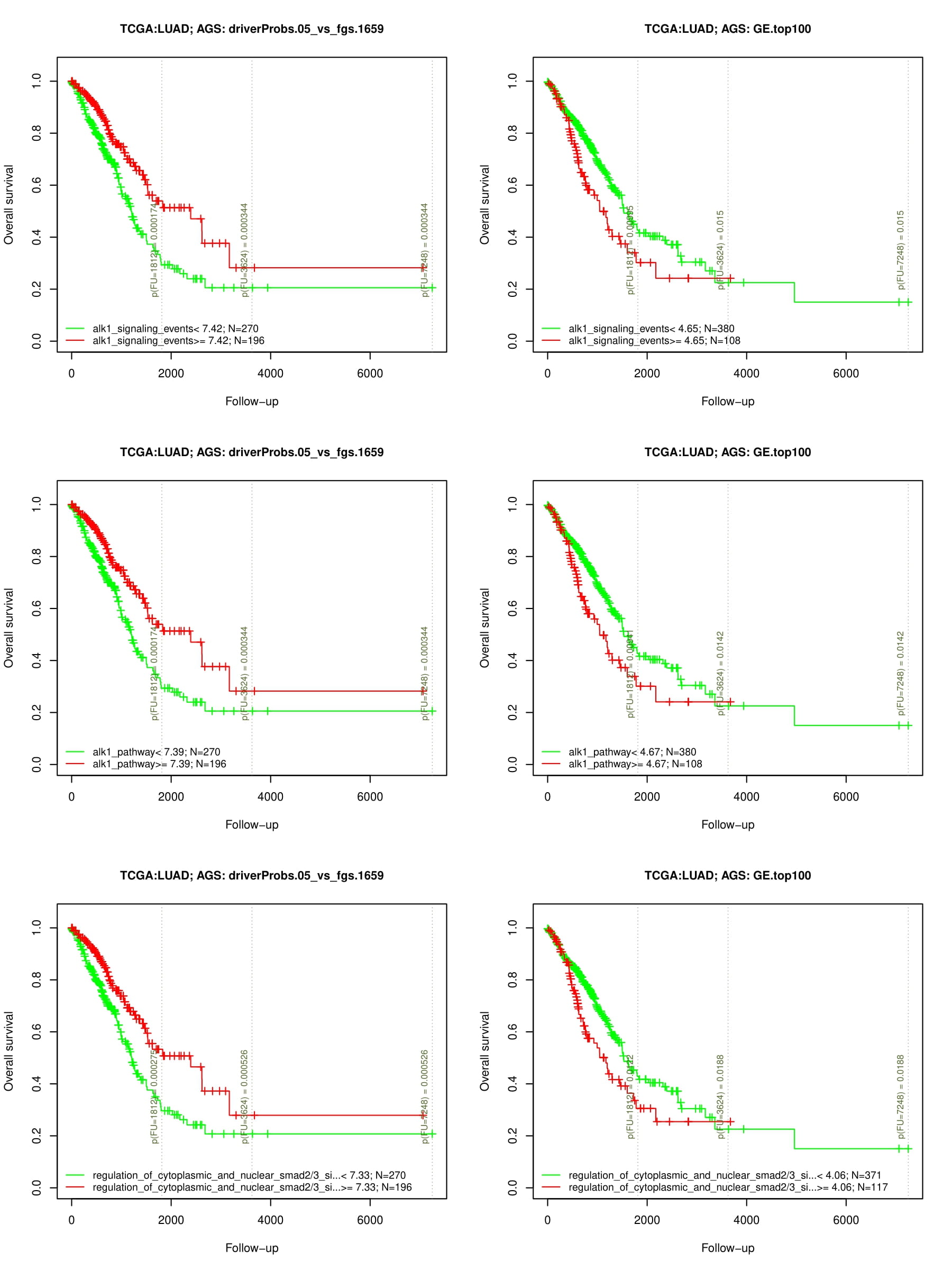

Supplement: Supplementary file 6. [file elife-74010-supp6.zip › SupplementaryFile6-19.jpg]

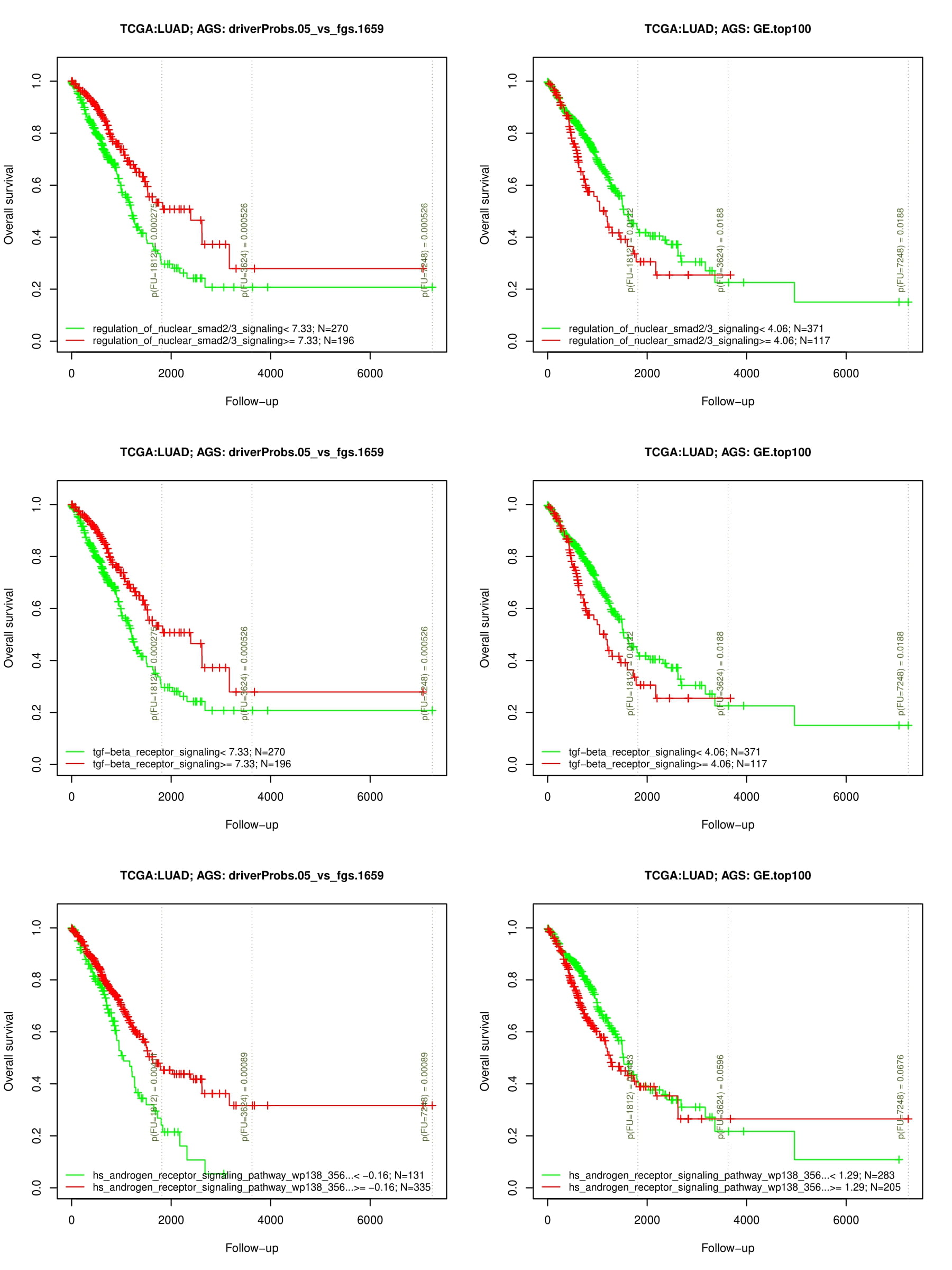

Supplement: Supplementary file 6. [file elife-74010-supp6.zip › SupplementaryFile6-20.jpg]

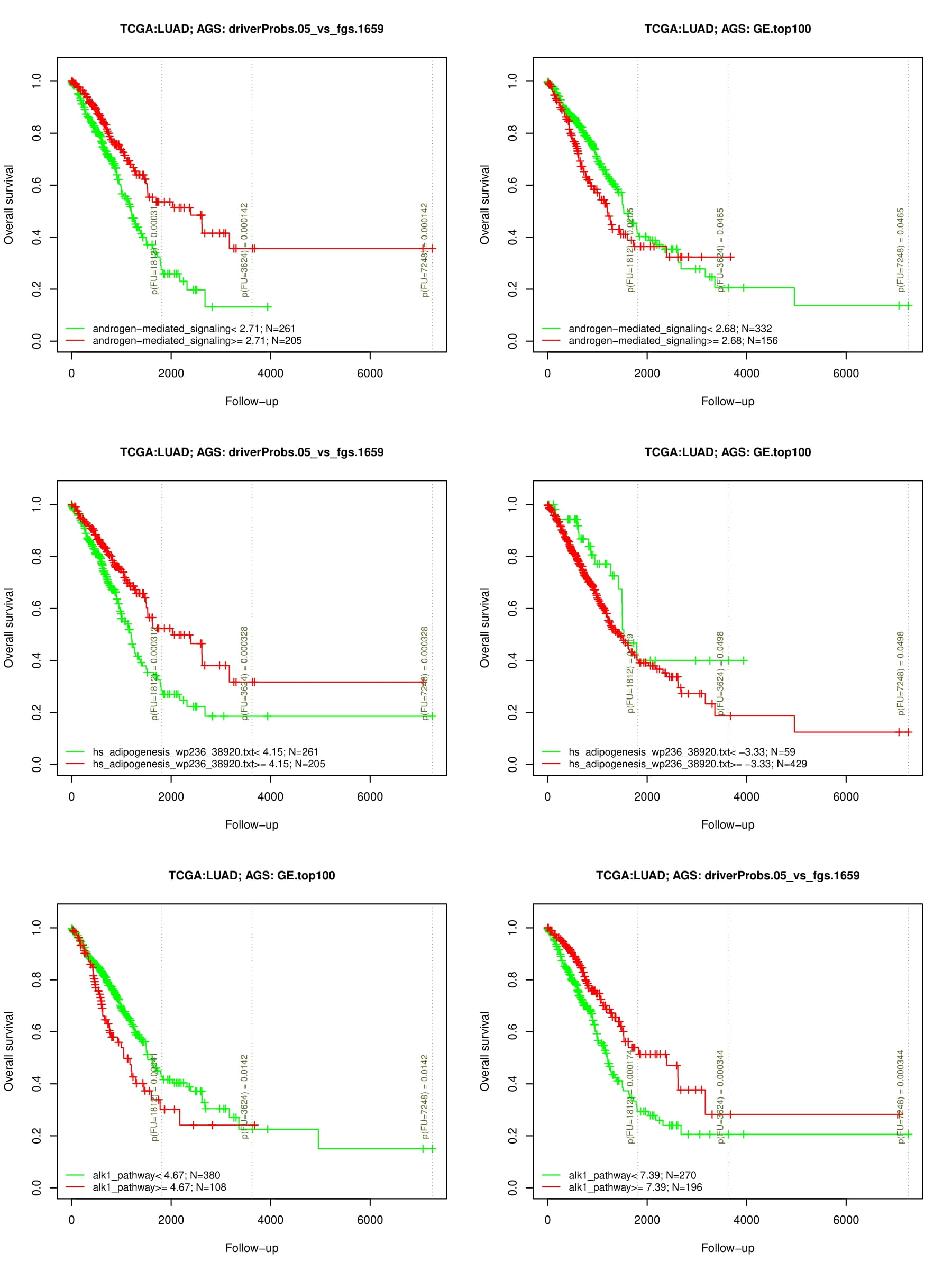

Supplement: Supplementary file 6. [file elife-74010-supp6.zip › SupplementaryFile6-21.jpg]

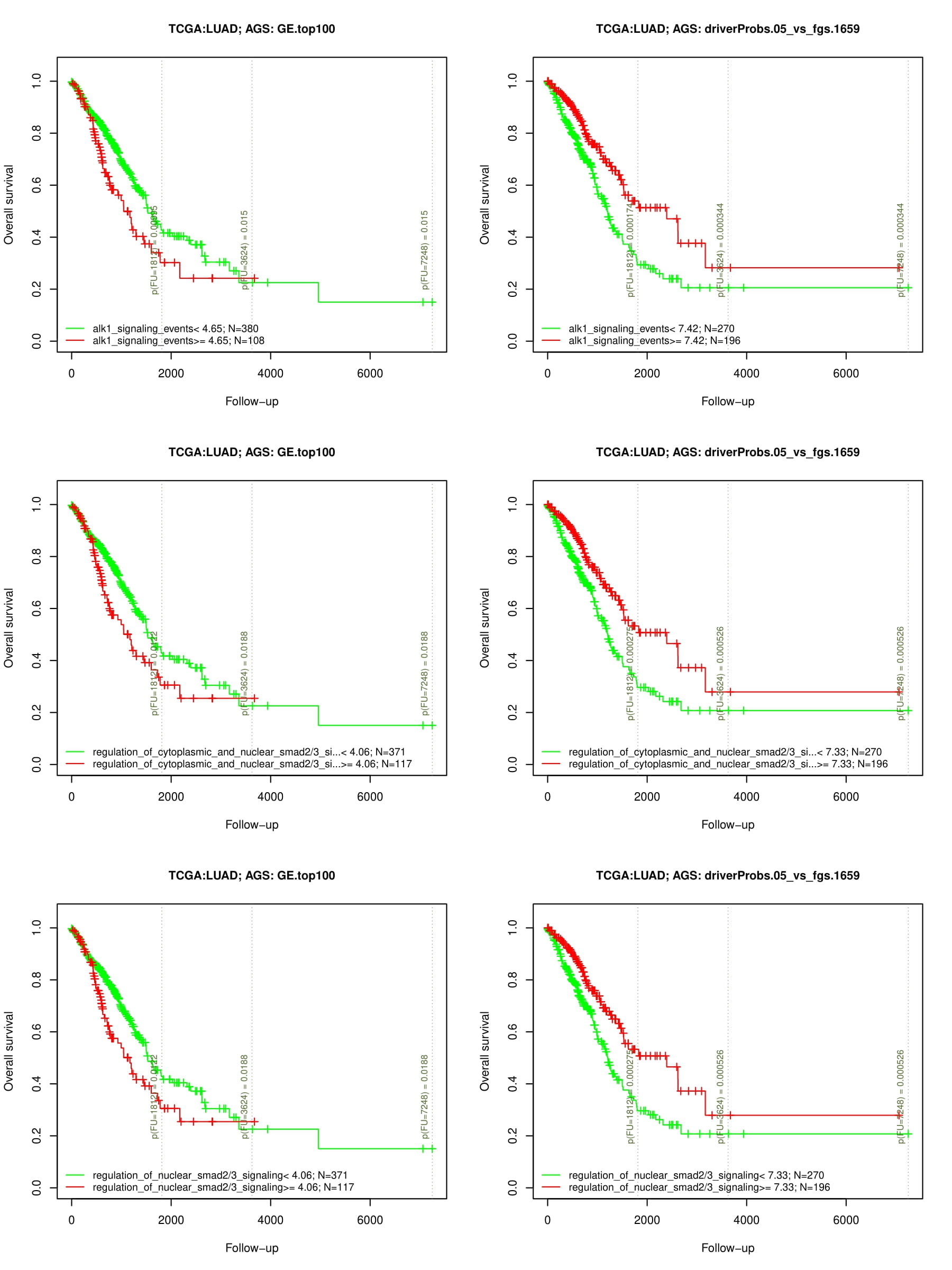

Supplement: Supplementary file 6. [file elife-74010-supp6.zip › SupplementaryFile6-22.jpg]

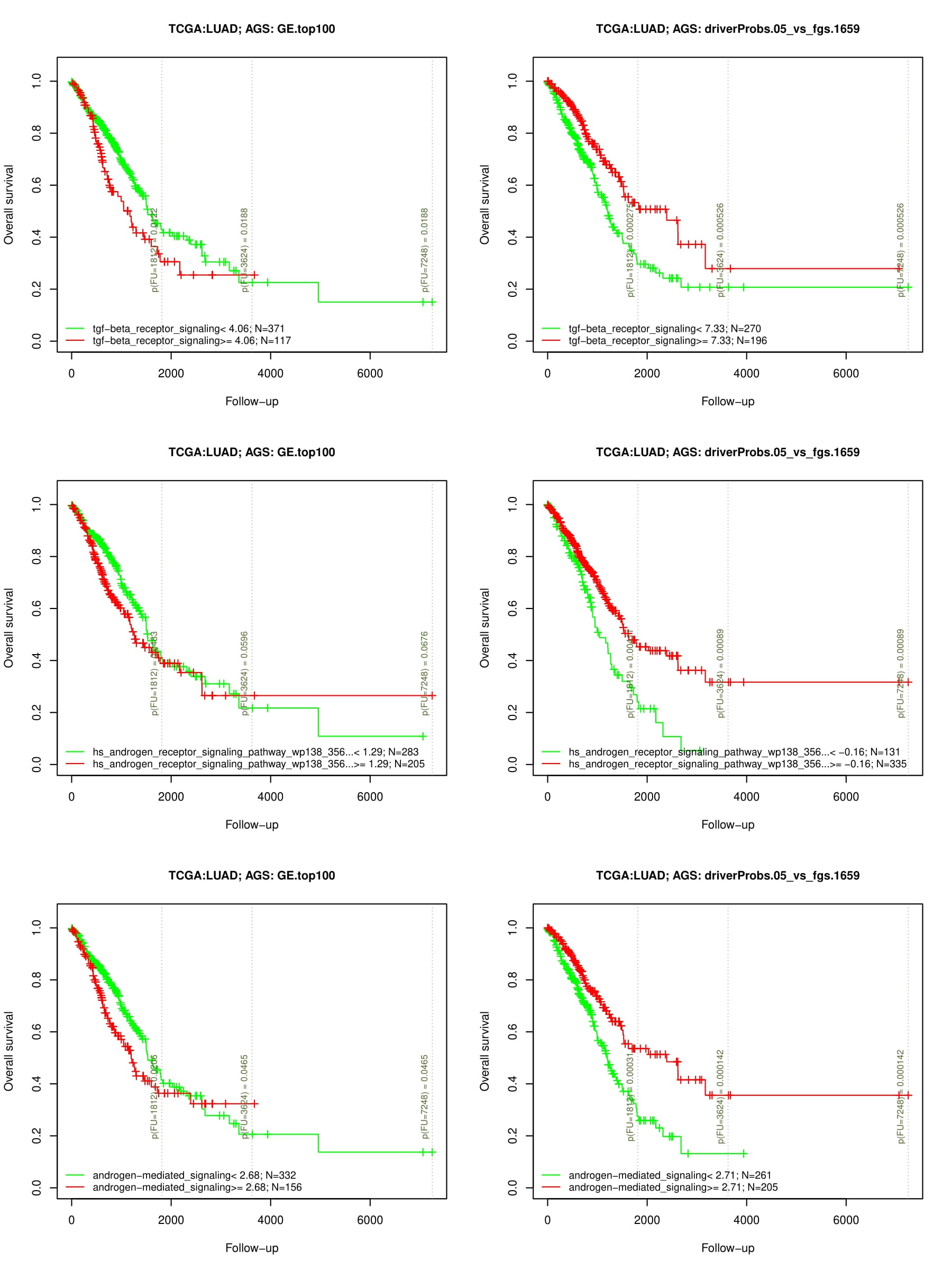

Supplement: Supplementary file 6. [file elife-74010-supp6.zip › SupplementaryFile6-23.jpg]

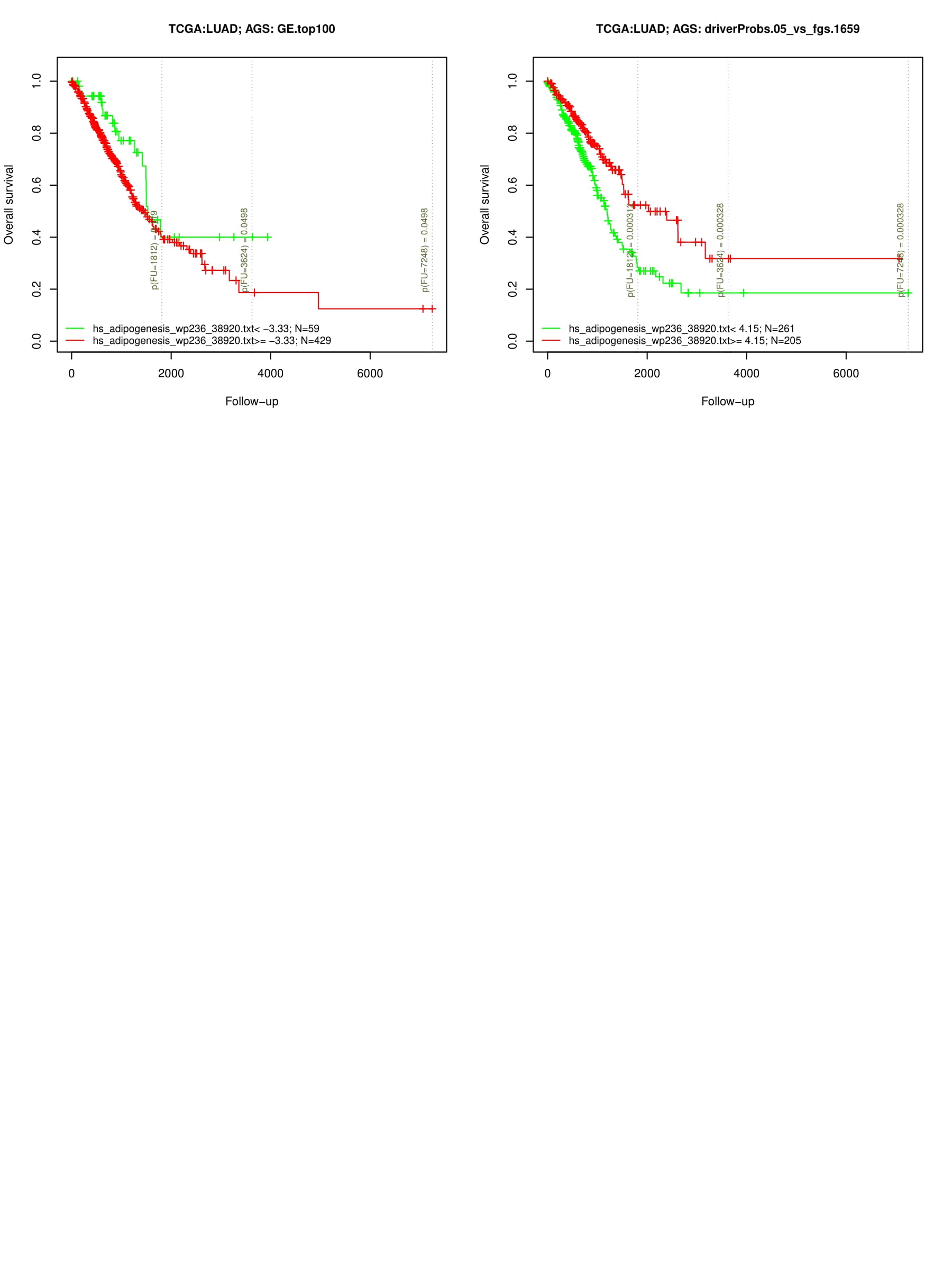

Supplement: Supplementary file 6. [file elife-74010-supp6.zip › SupplementaryFile6-24.jpg]

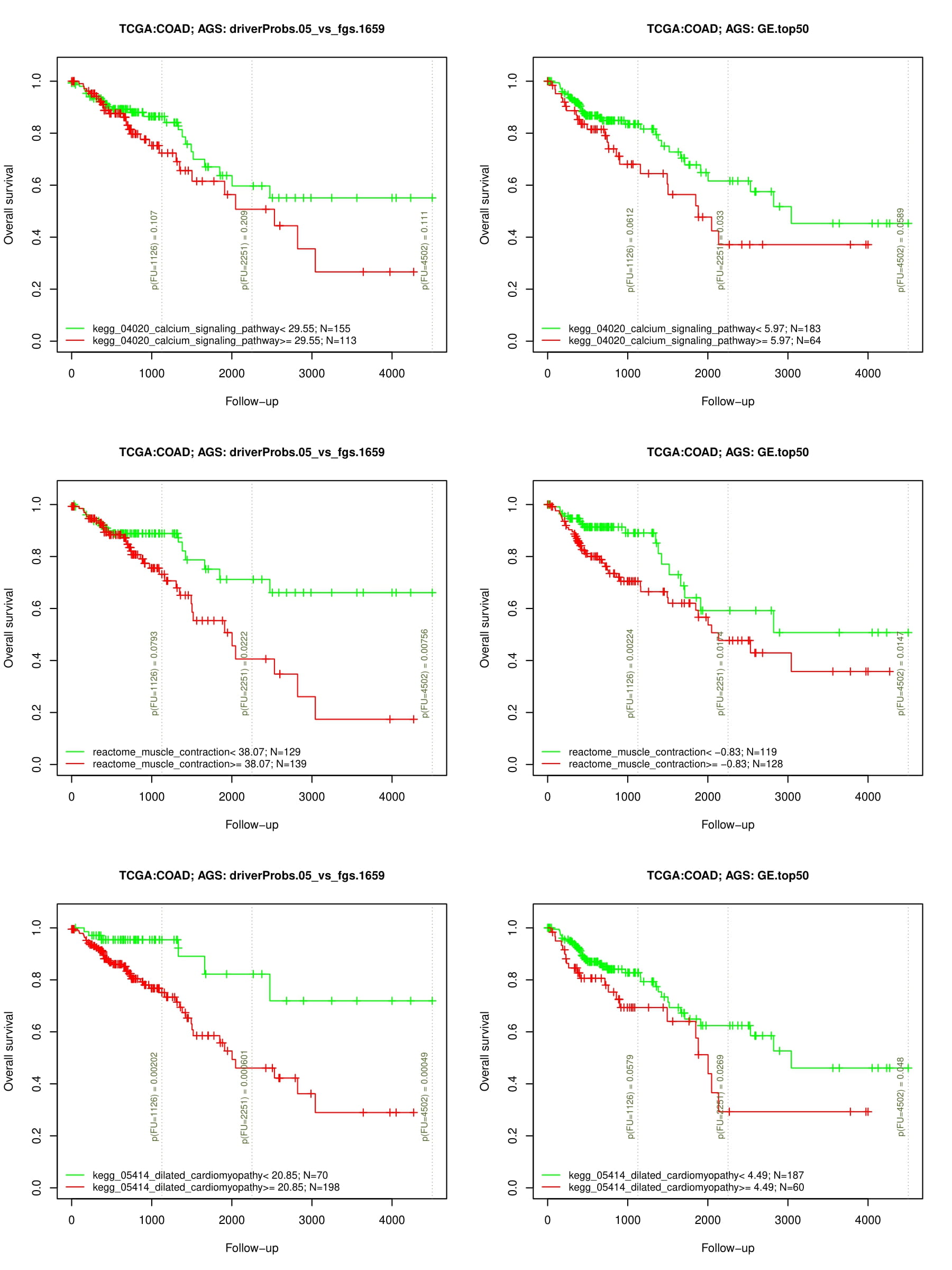

Supplement: Supplementary file 6. [file elife-74010-supp6.zip › SupplementaryFile6-25.jpg]

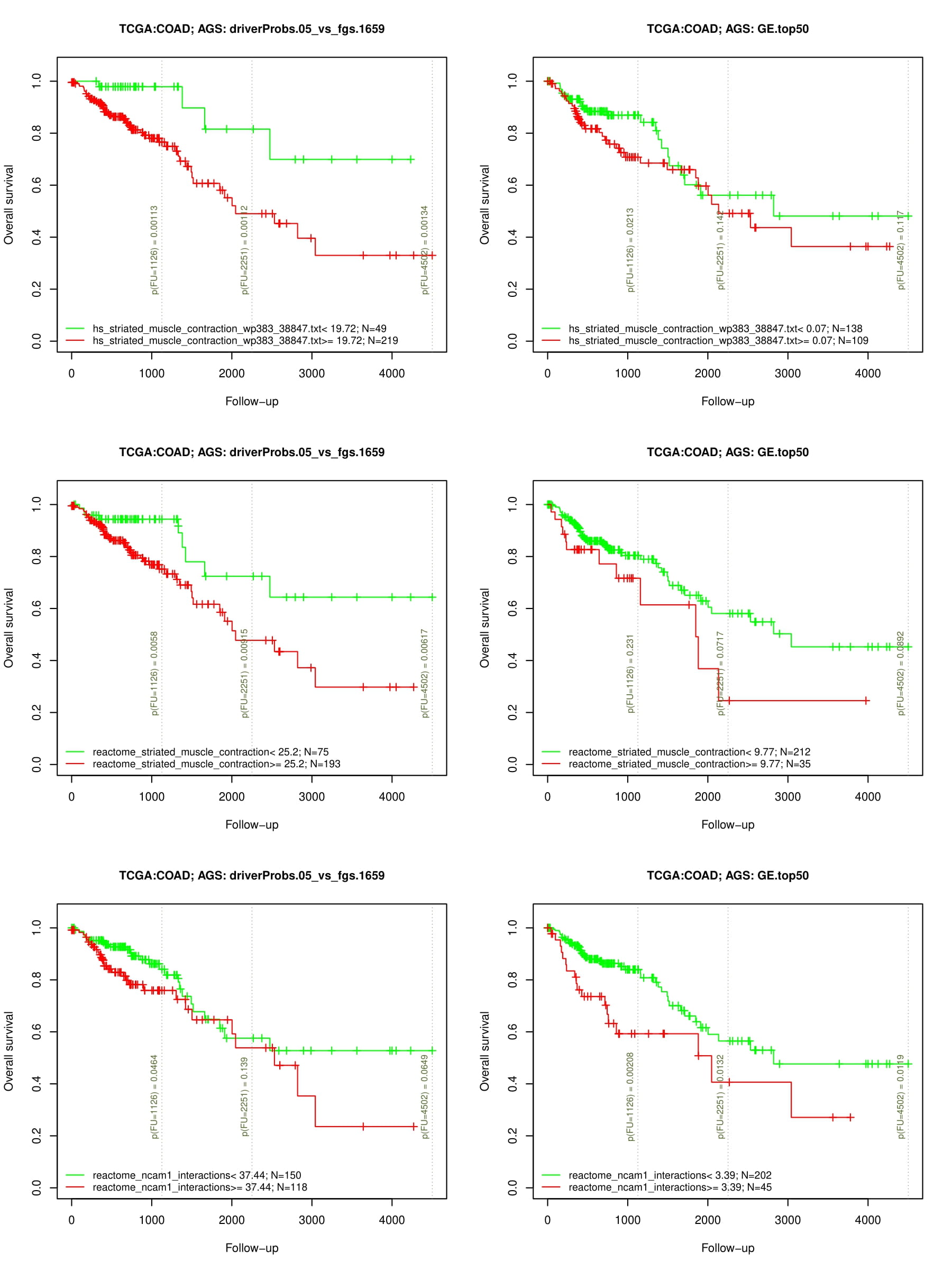

Supplement: Supplementary file 6. [file elife-74010-supp6.zip › SupplementaryFile6-26.jpg]

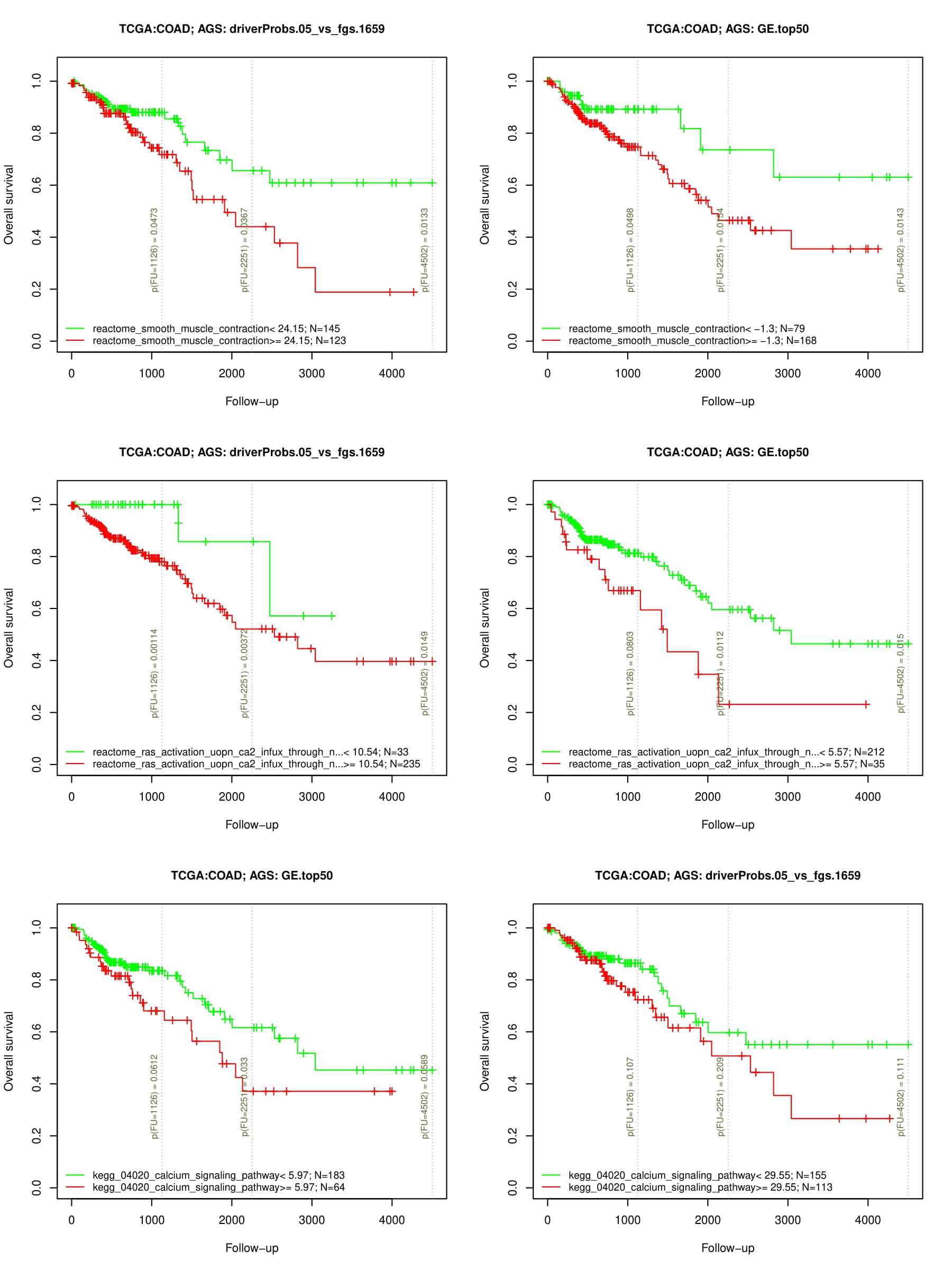

Supplement: Supplementary file 6. [file elife-74010-supp6.zip › SupplementaryFile6-27.jpg]

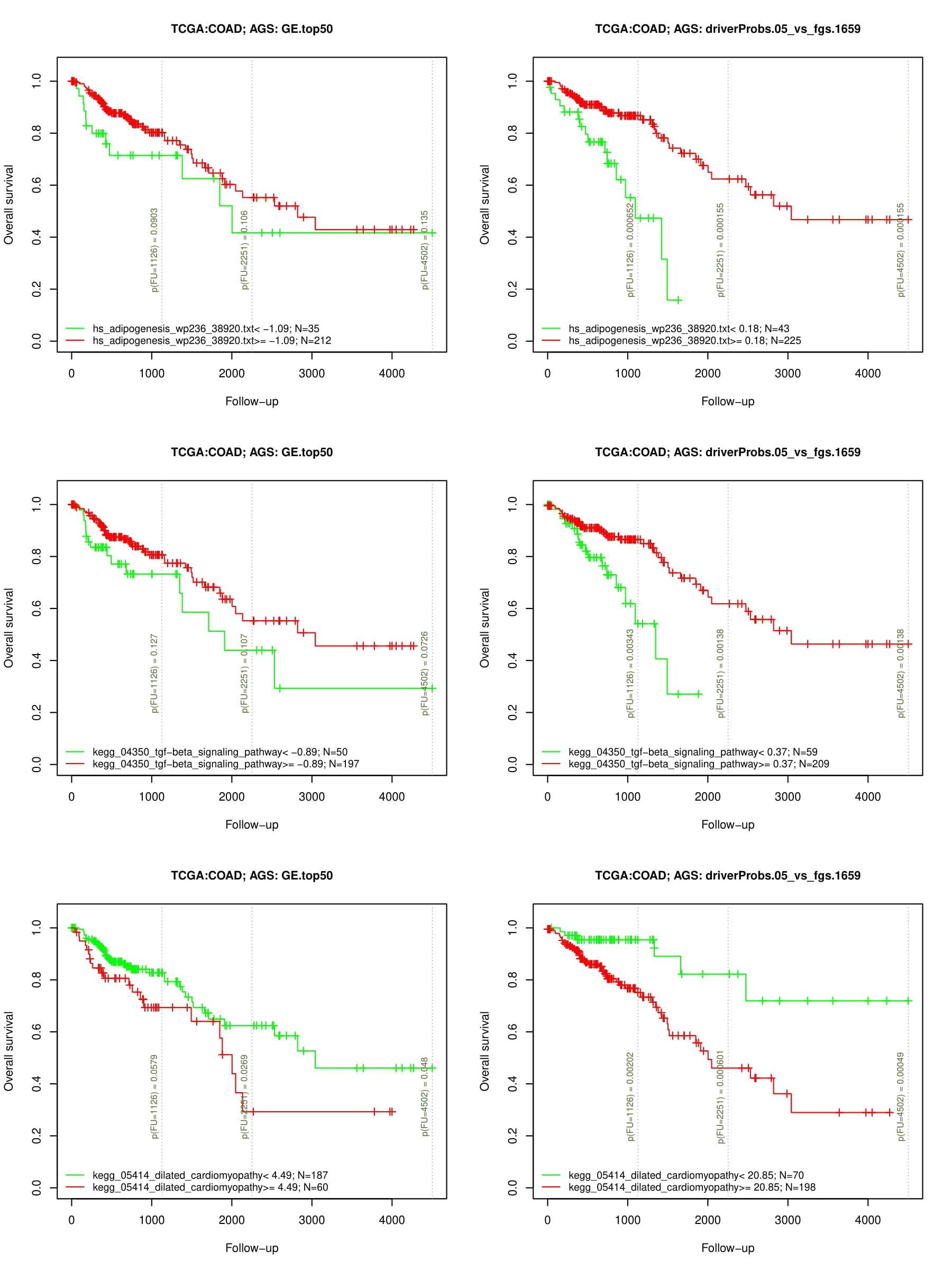

Supplement: Supplementary file 6. [file elife-74010-supp6.zip › SupplementaryFile6-28.jpg]

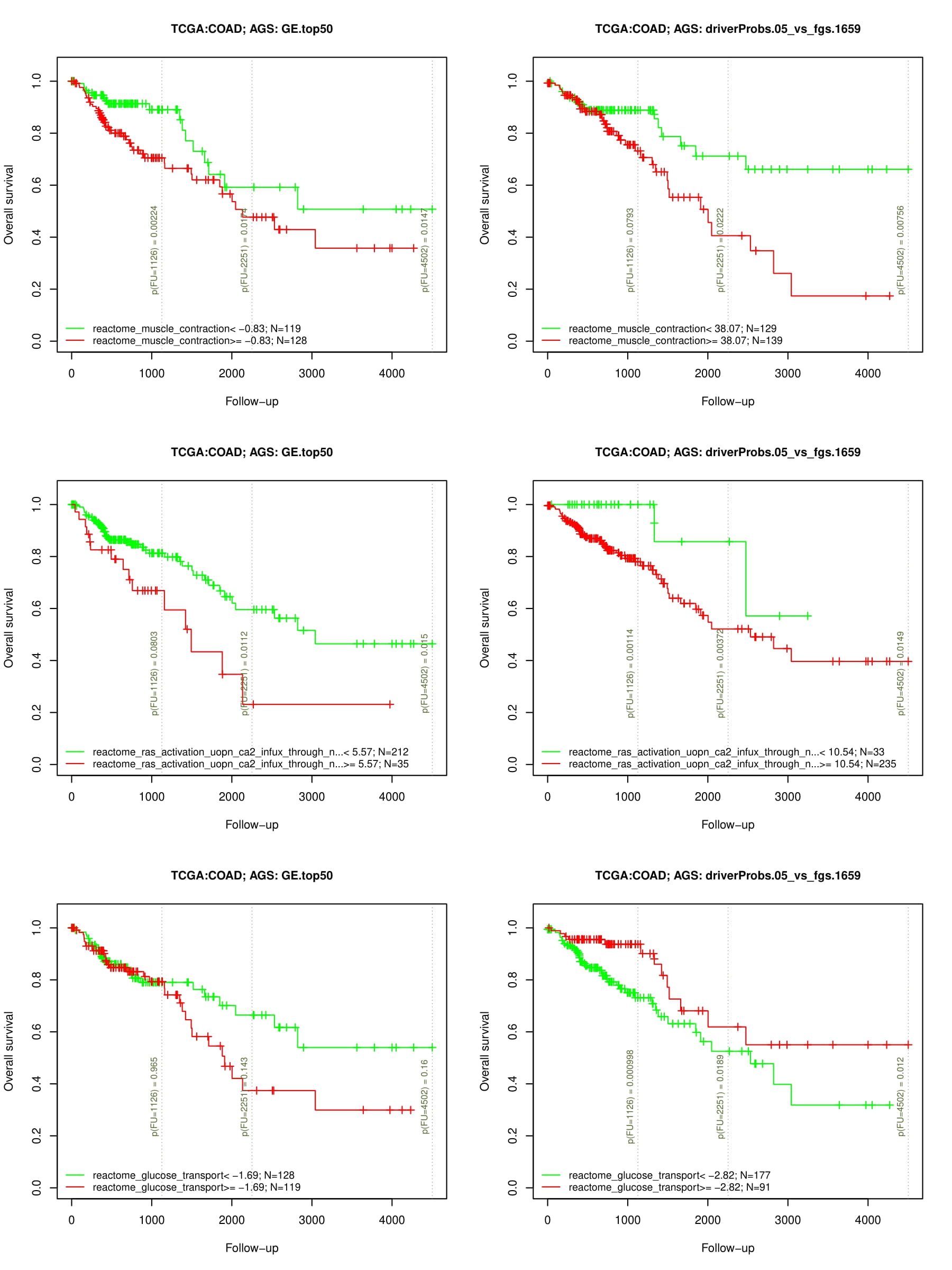

Supplement: Supplementary file 6. [file elife-74010-supp6.zip › SupplementaryFile6-29.jpg]

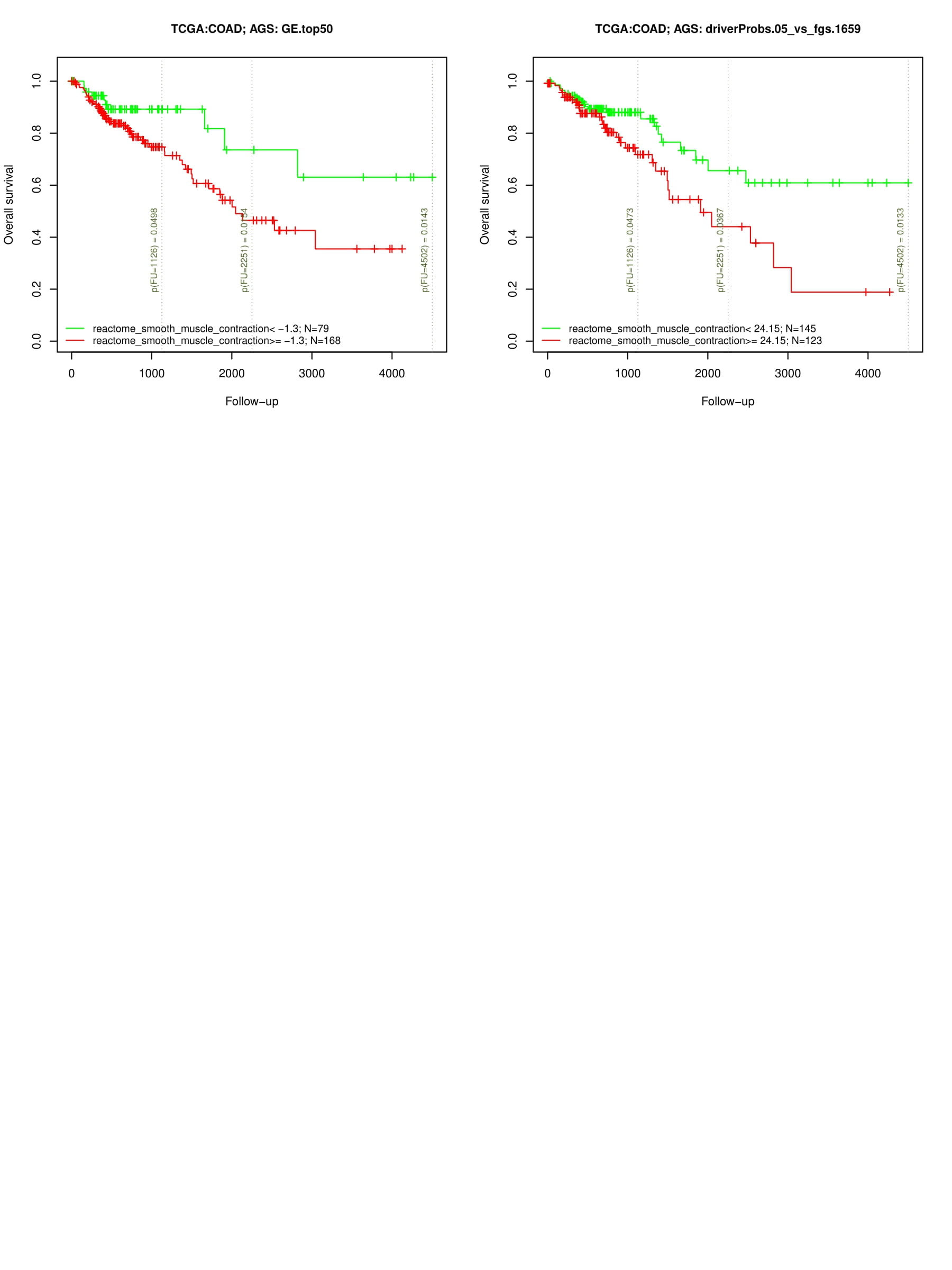

Supplement: Supplementary file 6. [file elife-74010-supp6.zip › SupplementaryFile6-30.jpg]

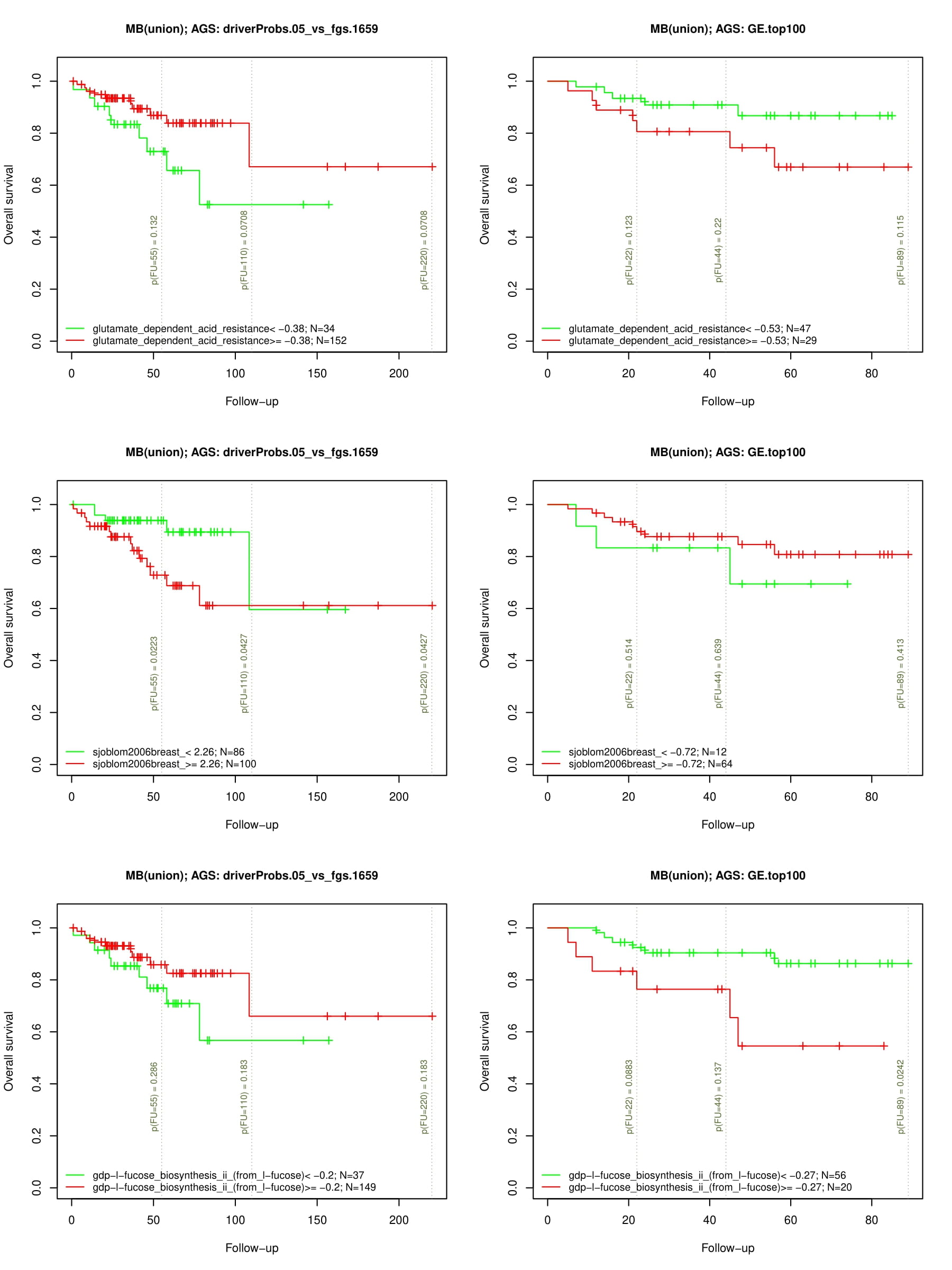

Supplement: Supplementary file 6. [file elife-74010-supp6.zip › SupplementaryFile6-31.jpg]

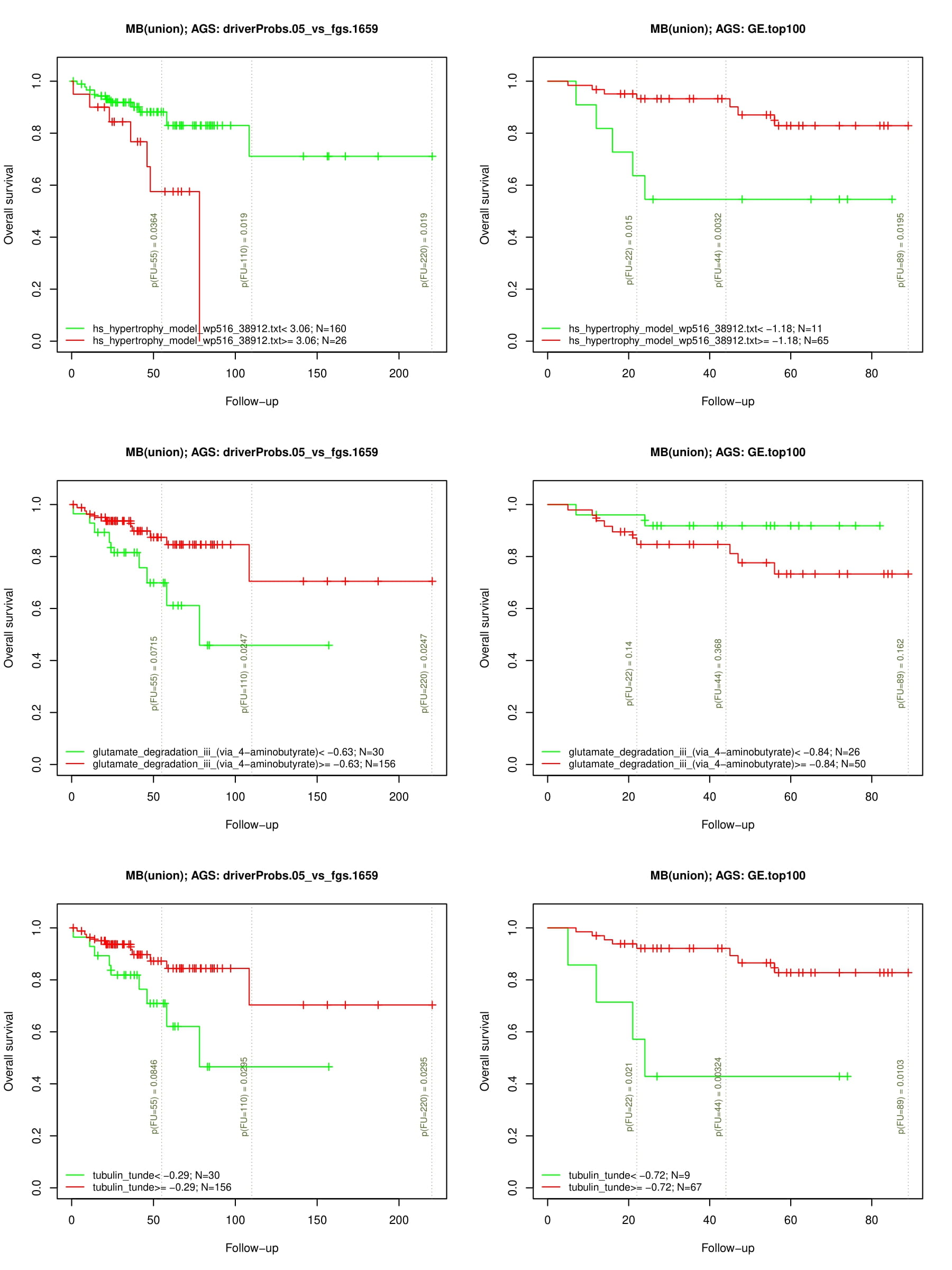

Supplement: Supplementary file 6. [file elife-74010-supp6.zip › SupplementaryFile6-32.jpg]

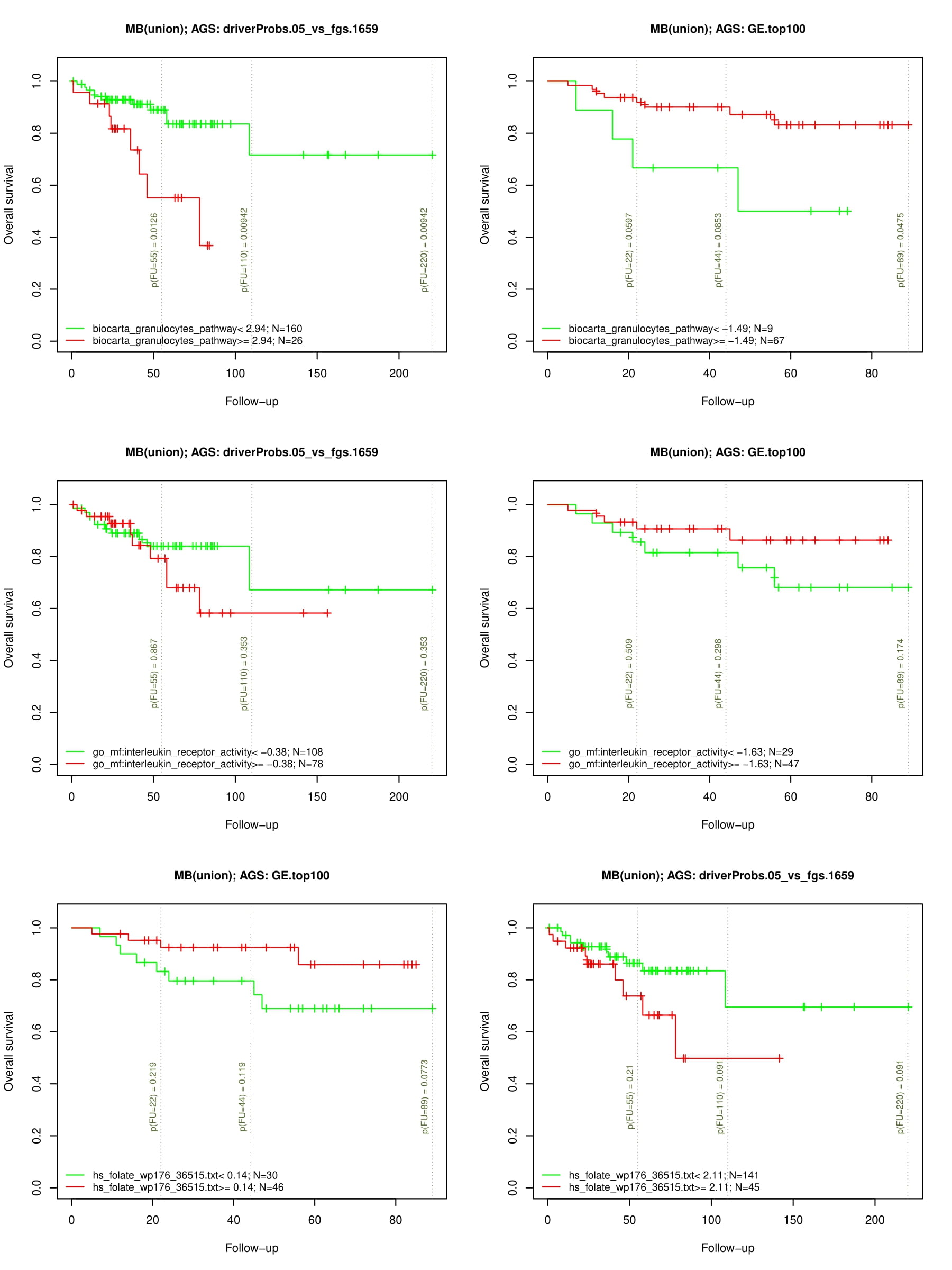

Supplement: Supplementary file 6. [file elife-74010-supp6.zip › SupplementaryFile6-33.jpg]

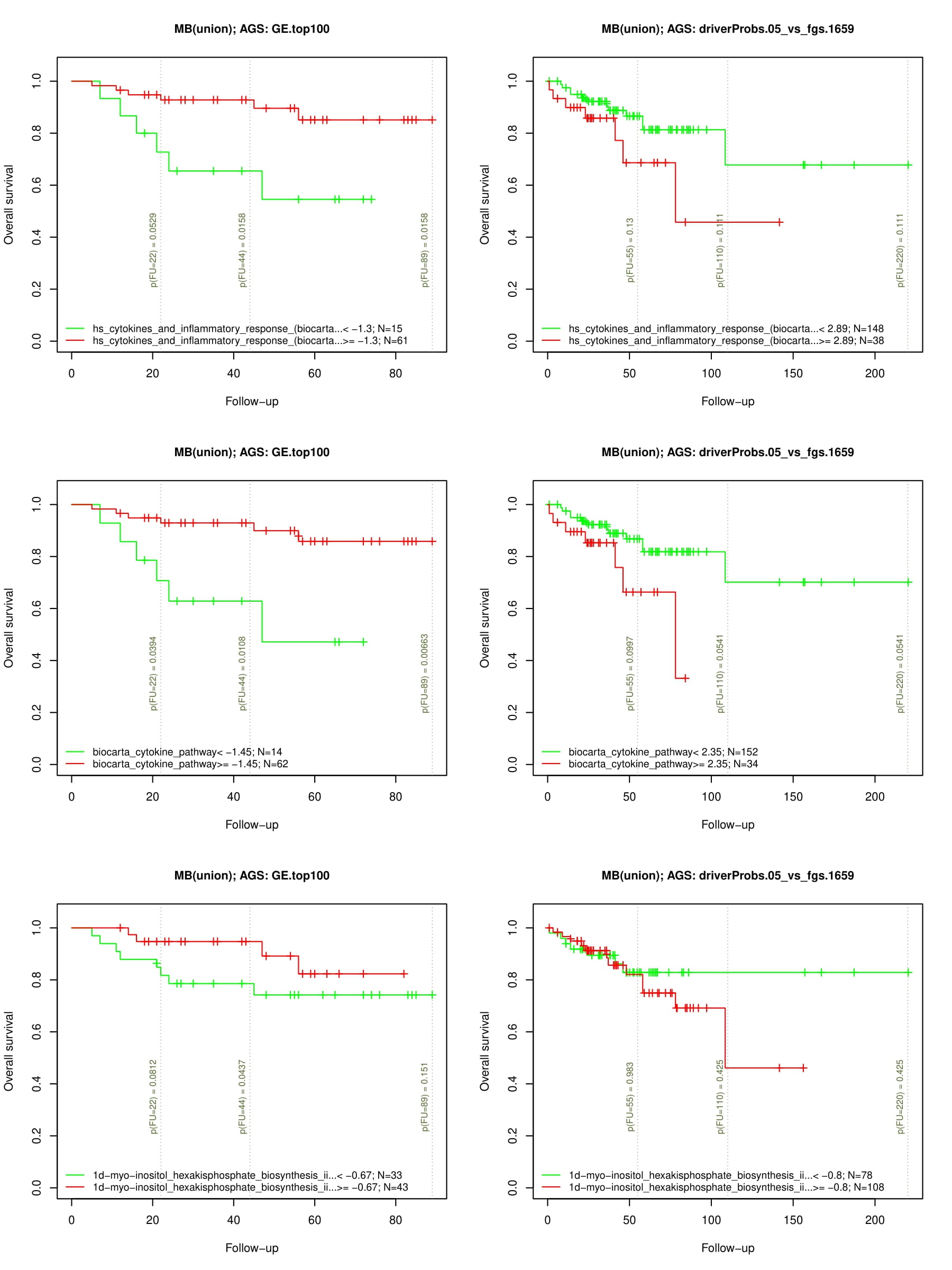

Supplement: Supplementary file 6. [file elife-74010-supp6.zip › SupplementaryFile6-34.jpg]

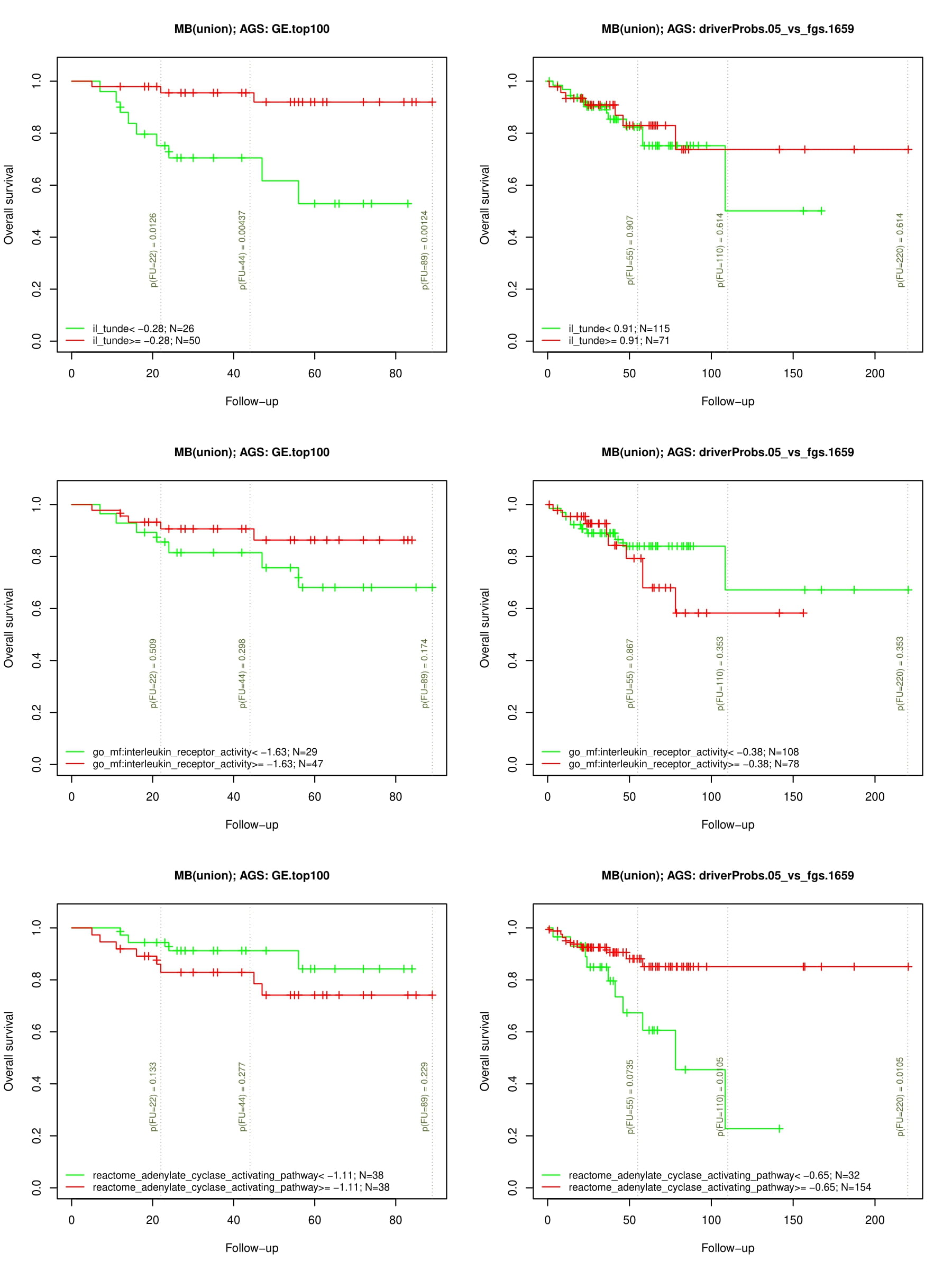

Supplement: Supplementary file 6. [file elife-74010-supp6.zip › SupplementaryFile6-35.jpg]

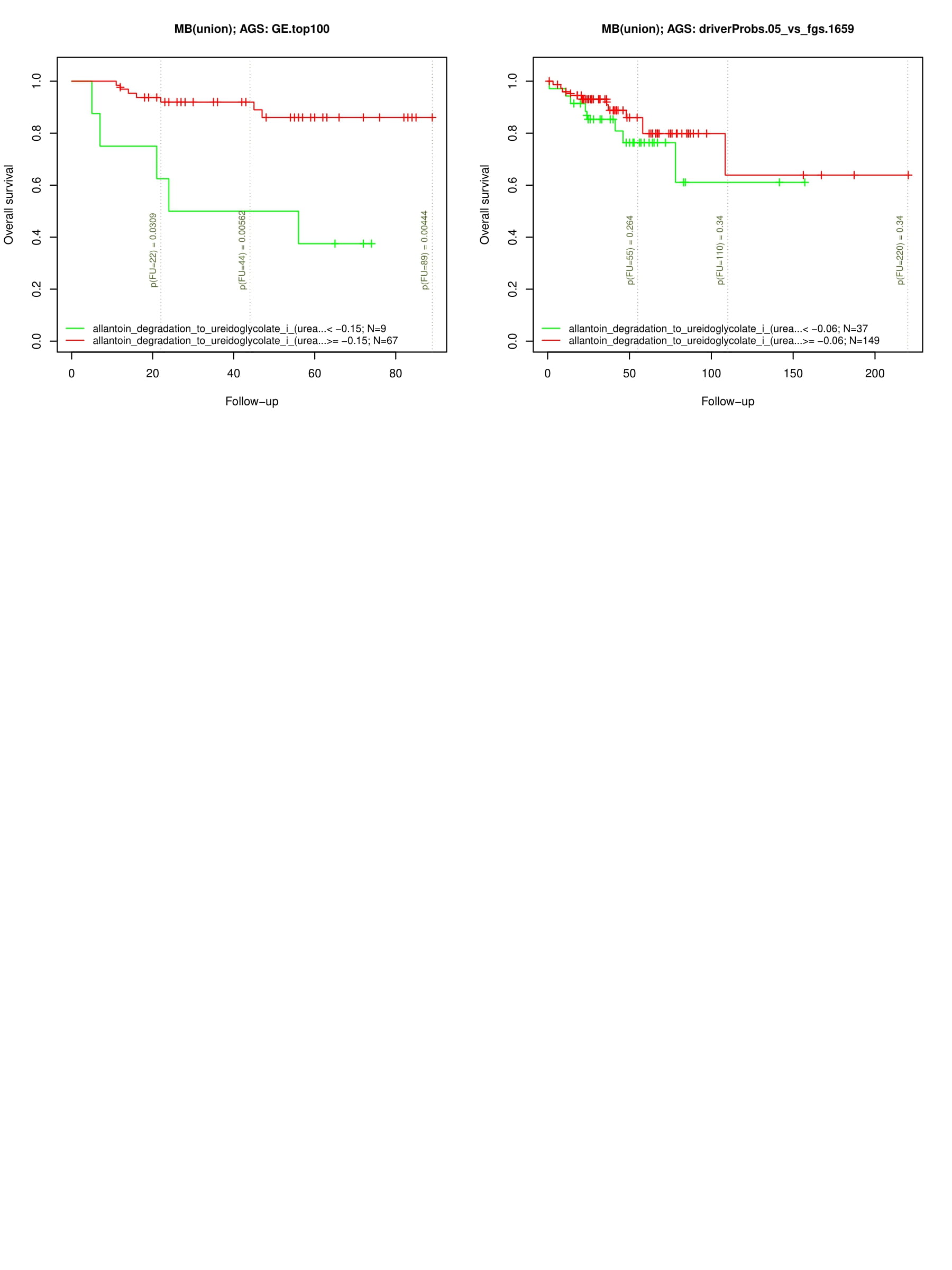

Supplement: Supplementary file 6. [file elife-74010-supp6.zip › SupplementaryFile6-36.jpg]

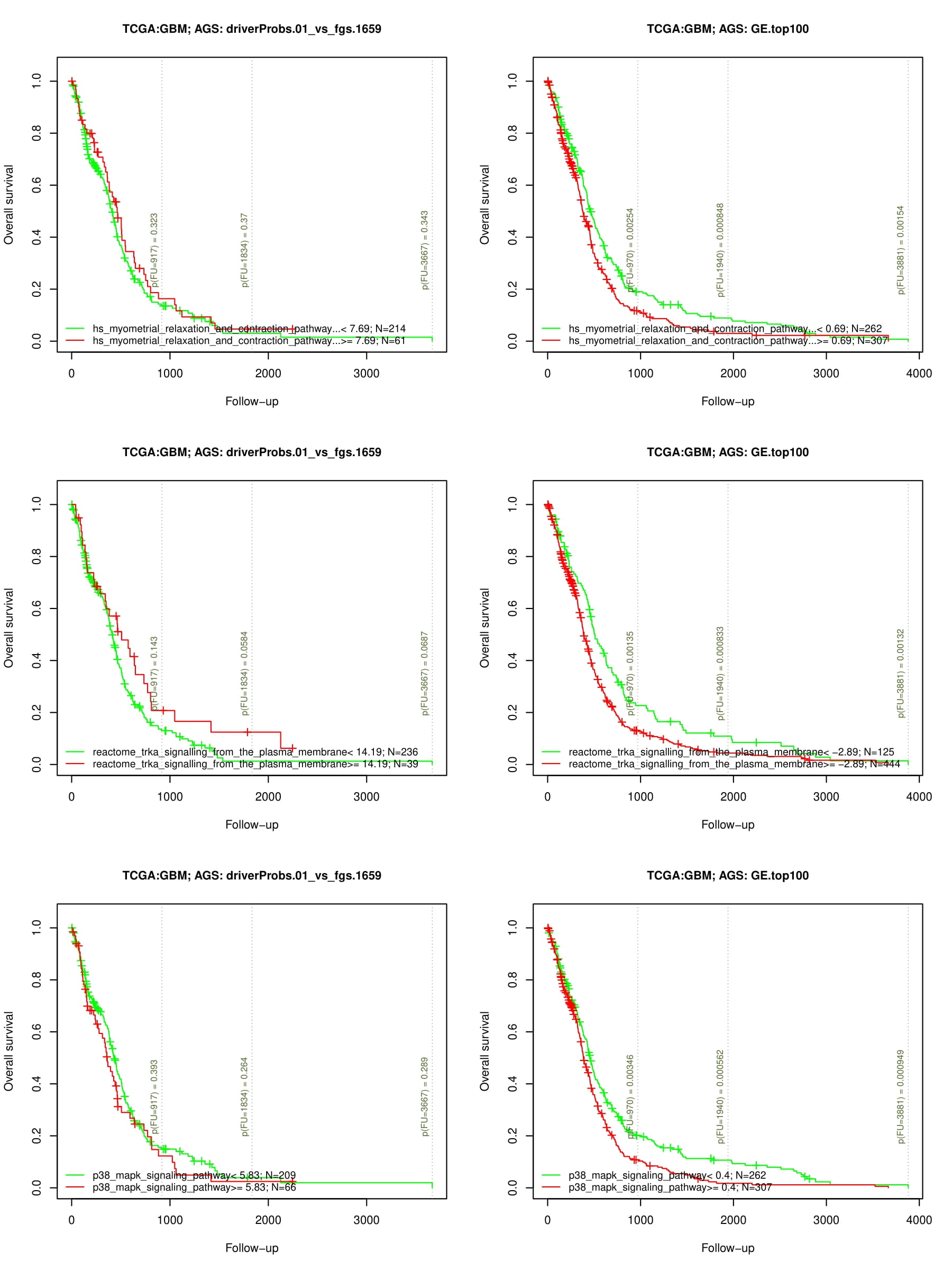

Supplement: Supplementary file 6. [file elife-74010-supp6.zip › SupplementaryFile6-37.jpg]

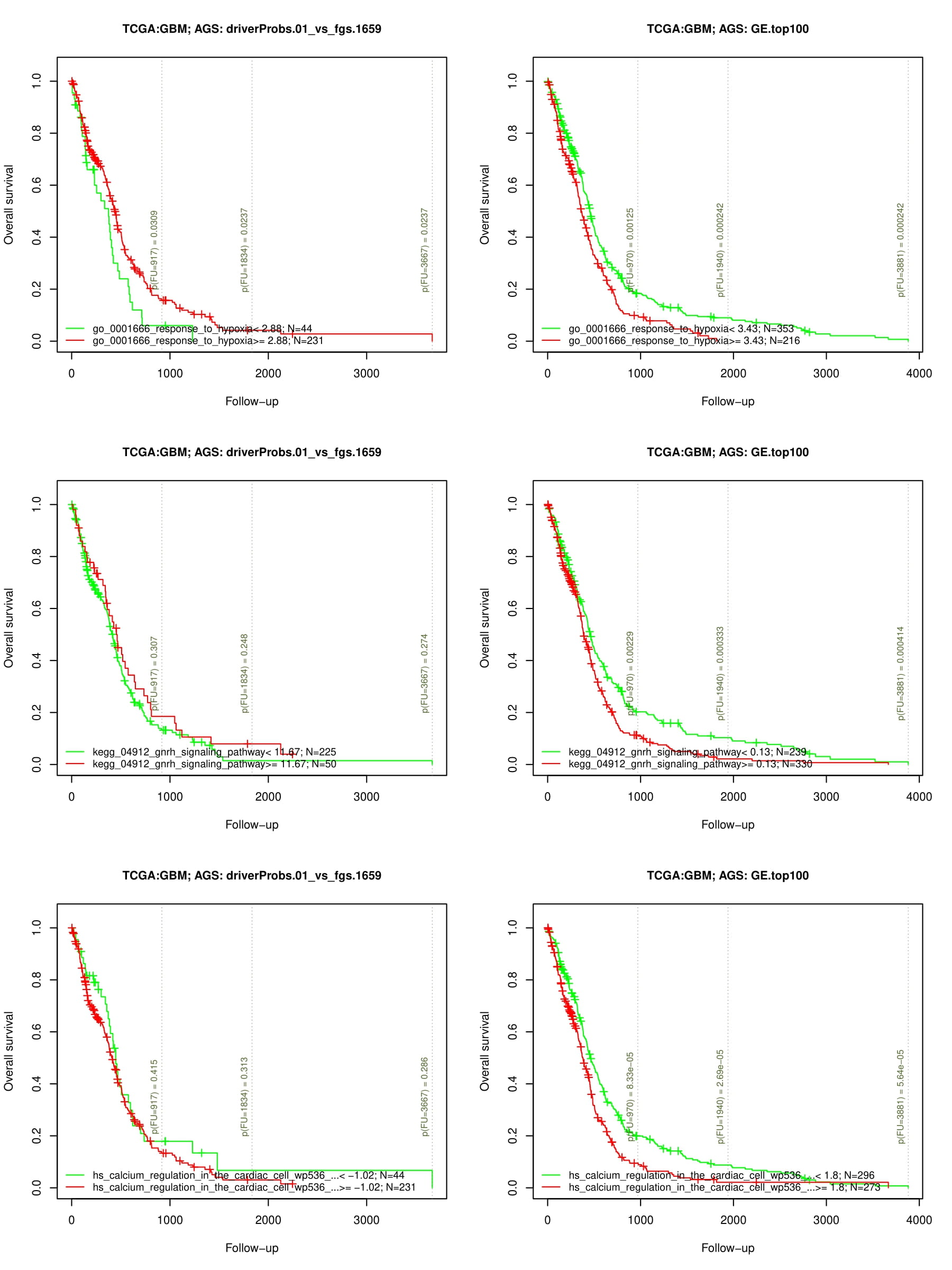

Supplement: Supplementary file 6. [file elife-74010-supp6.zip › SupplementaryFile6-38.jpg]

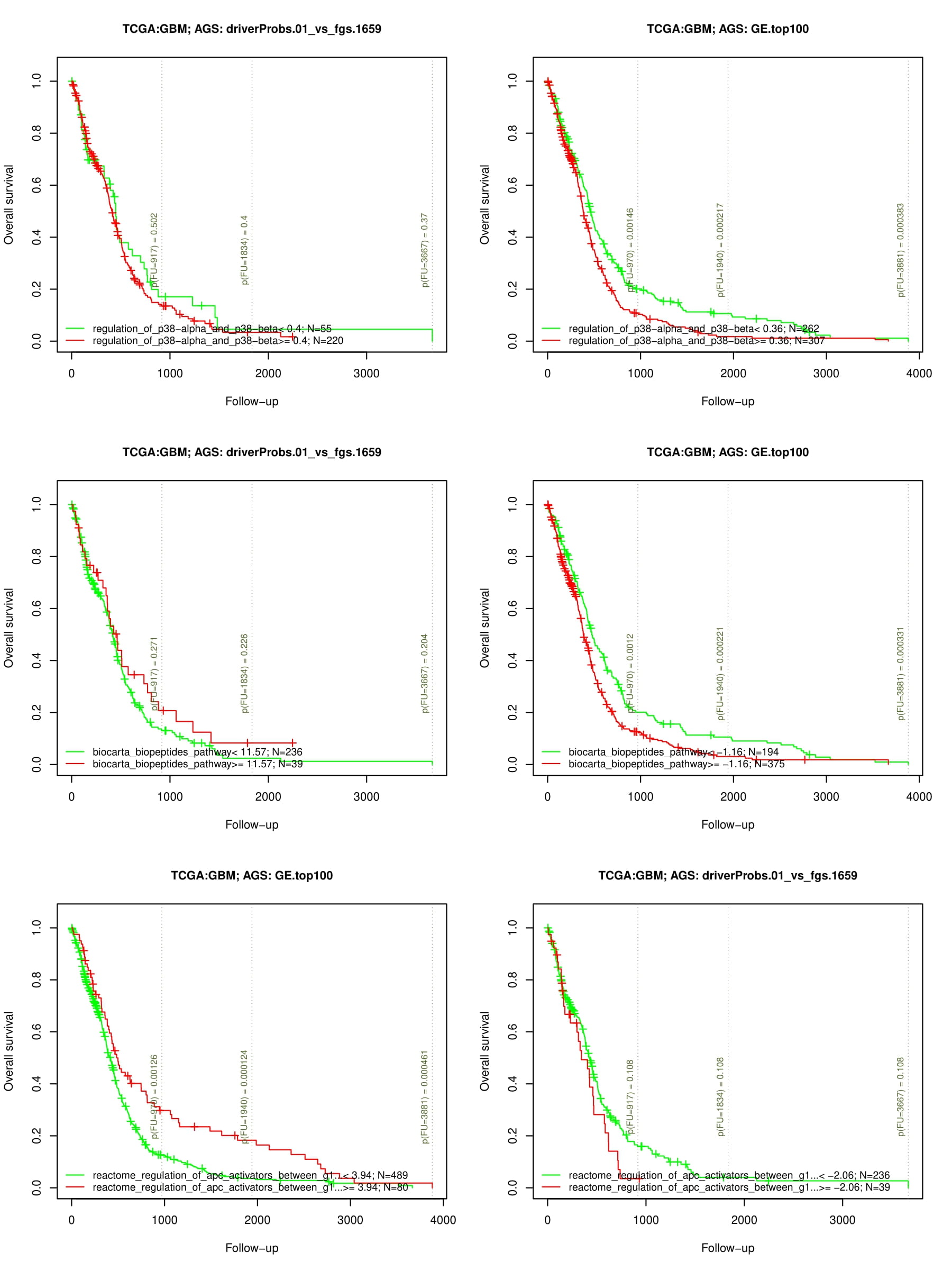

Supplement: Supplementary file 6. [file elife-74010-supp6.zip › SupplementaryFile6-39.jpg]

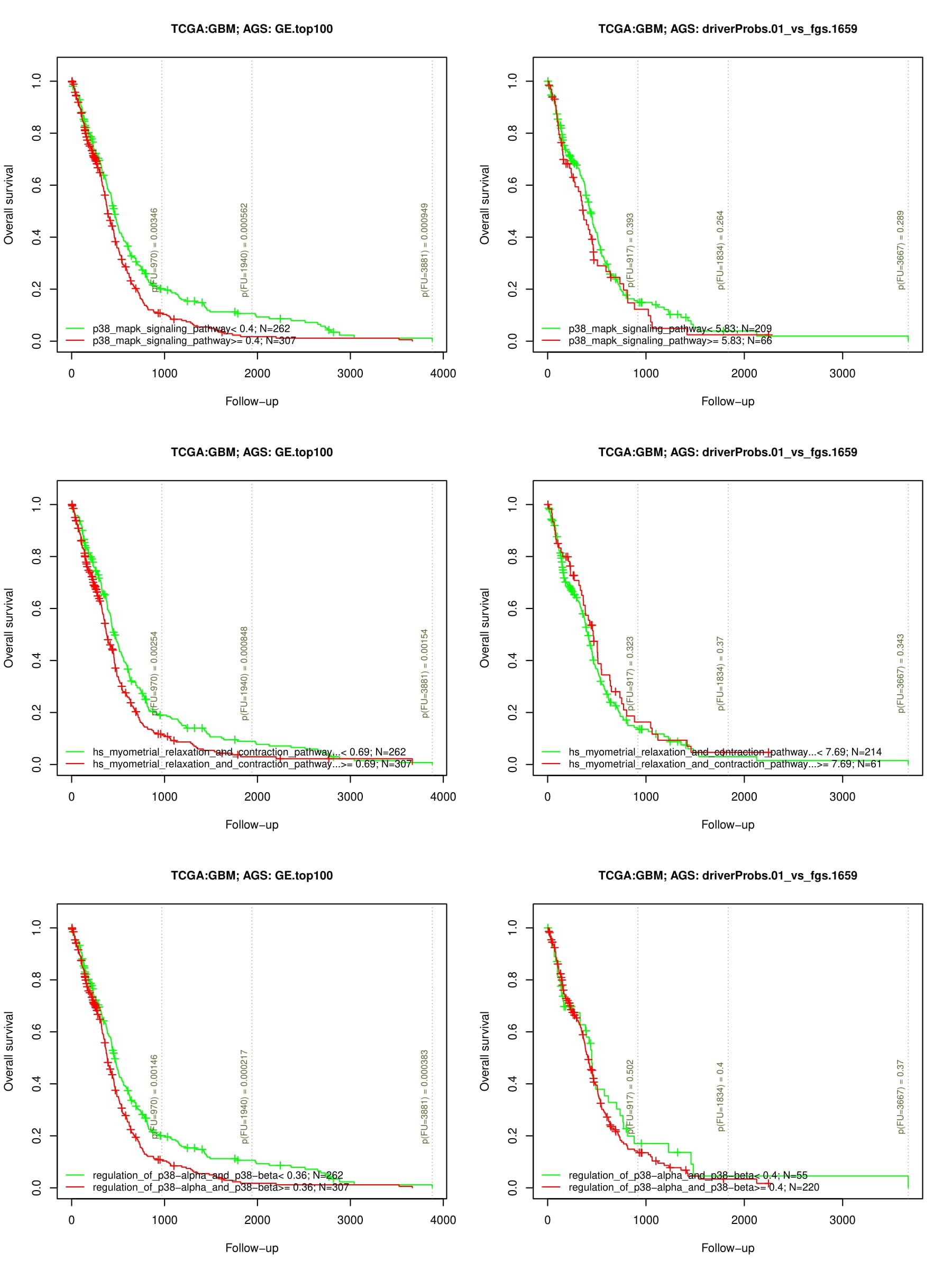

Supplement: Supplementary file 6. [file elife-74010-supp6.zip › SupplementaryFile6-40.jpg]

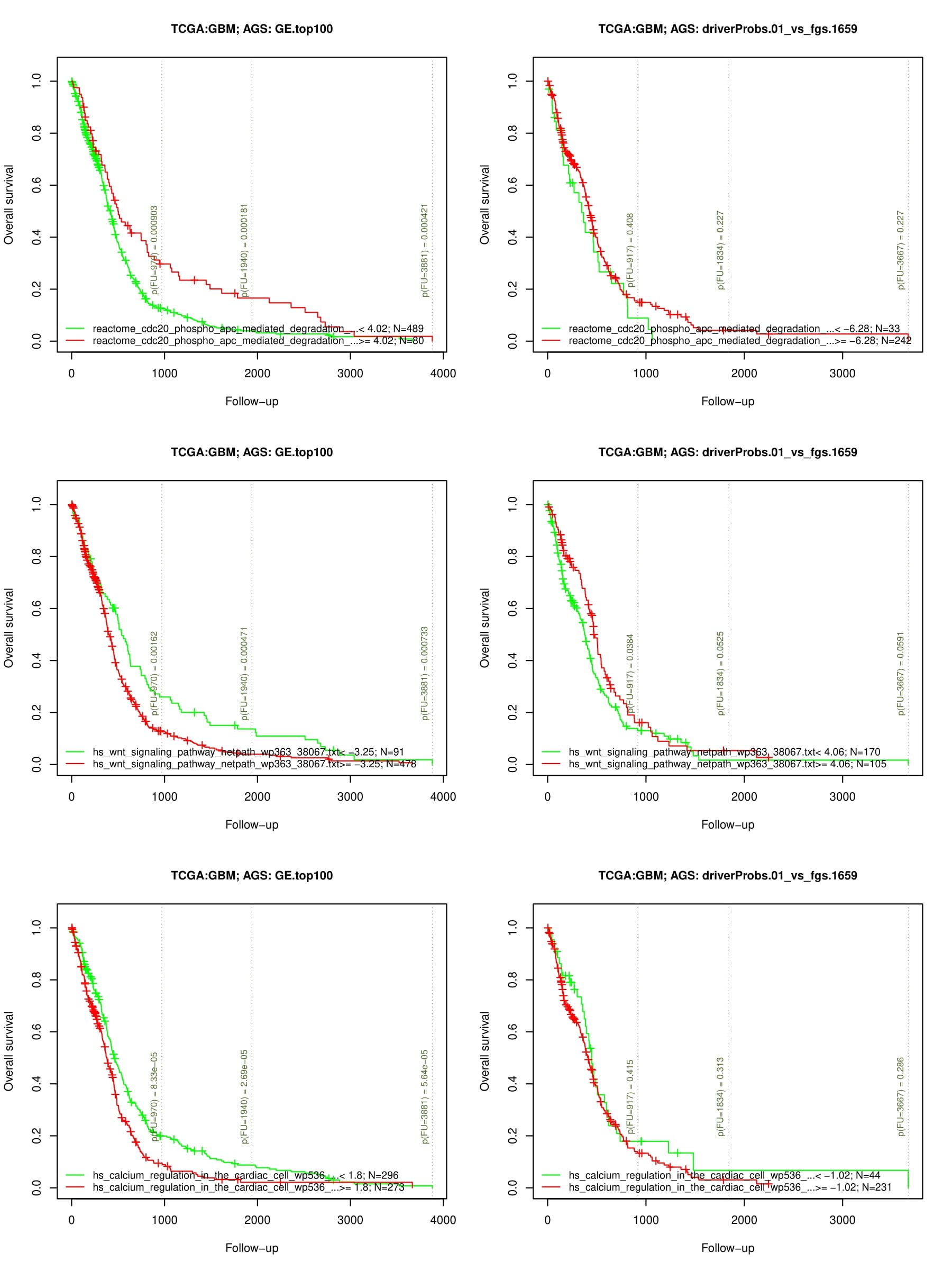

Supplement: Supplementary file 6. [file elife-74010-supp6.zip › SupplementaryFile6-41.jpg]

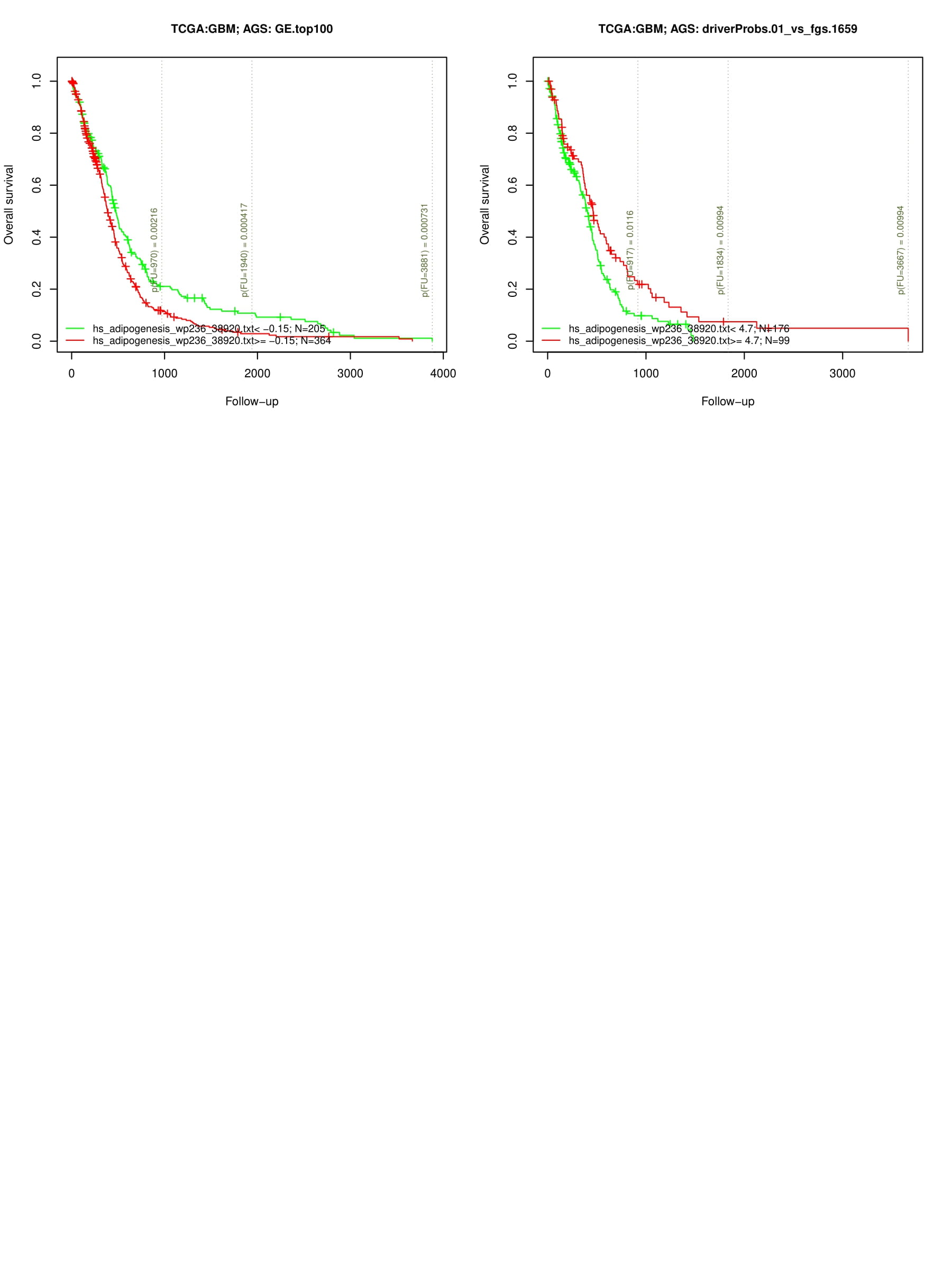

Supplement: Supplementary file 6. [file elife-74010-supp6.zip › SupplementaryFile6-42.jpg]

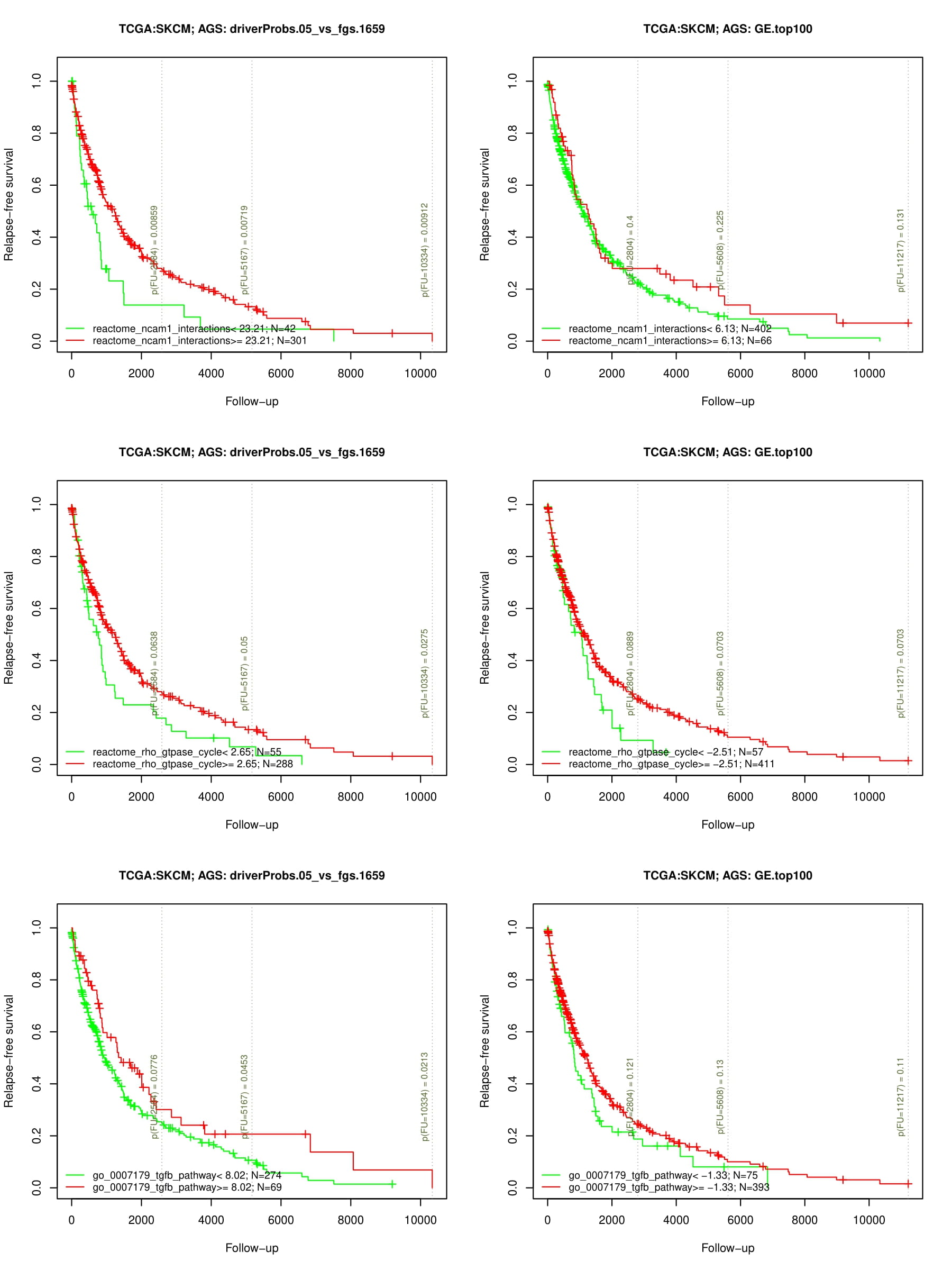

Supplement: Supplementary file 6. [file elife-74010-supp6.zip › SupplementaryFile6-43.jpg]

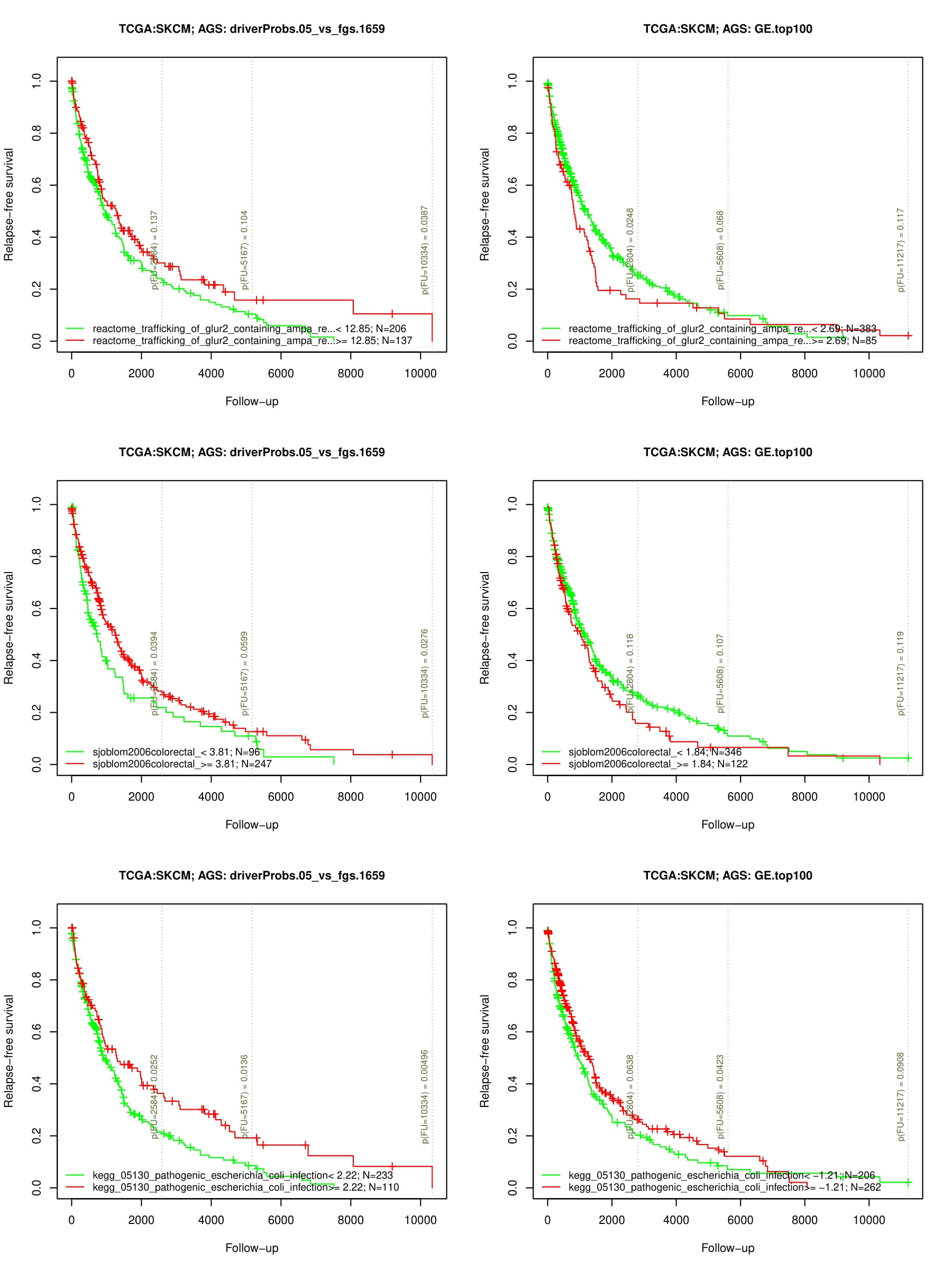

Supplement: Supplementary file 6. [file elife-74010-supp6.zip › SupplementaryFile6-44.jpg]

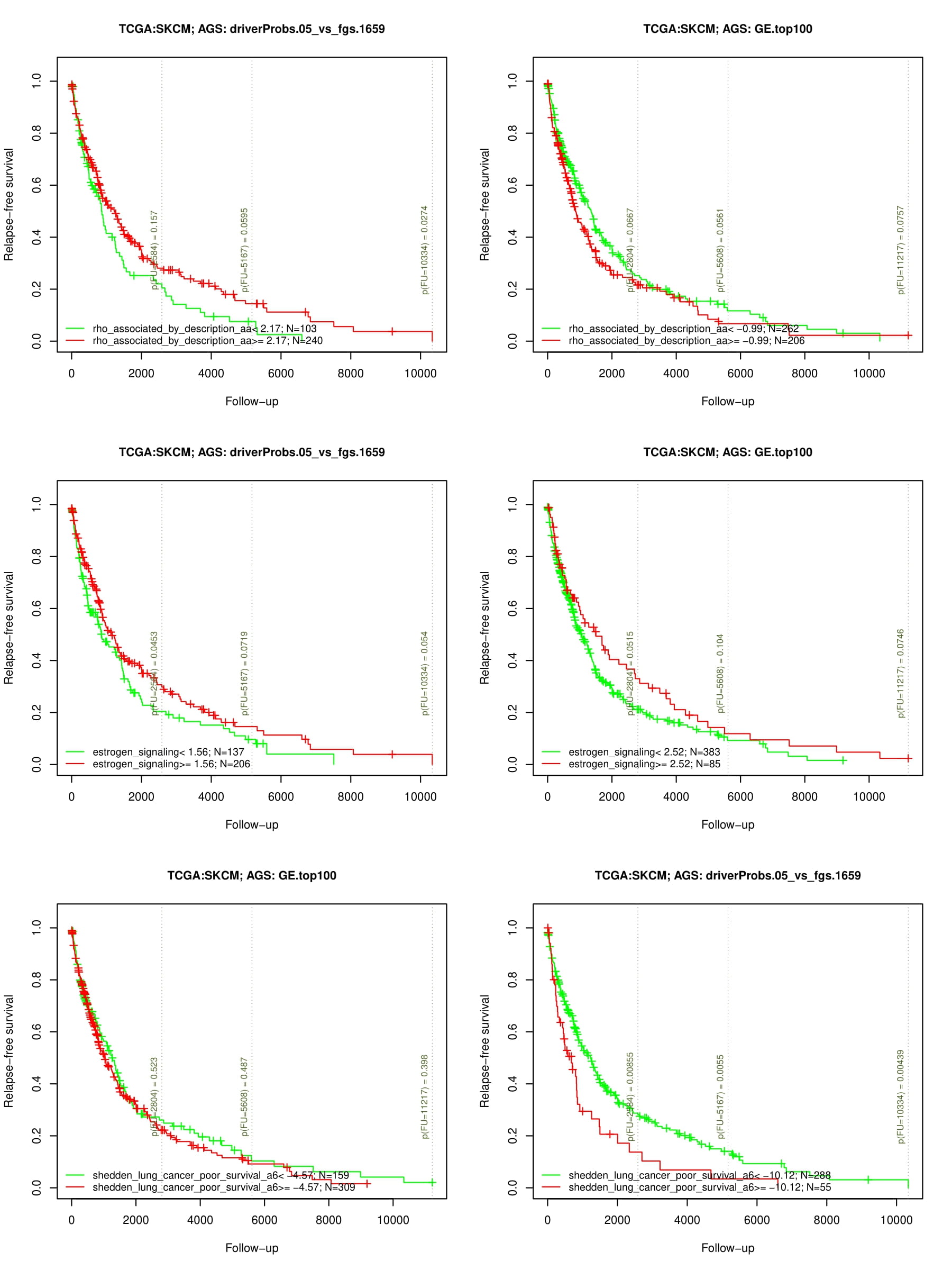

Supplement: Supplementary file 6. [file elife-74010-supp6.zip › SupplementaryFile6-45.jpg]

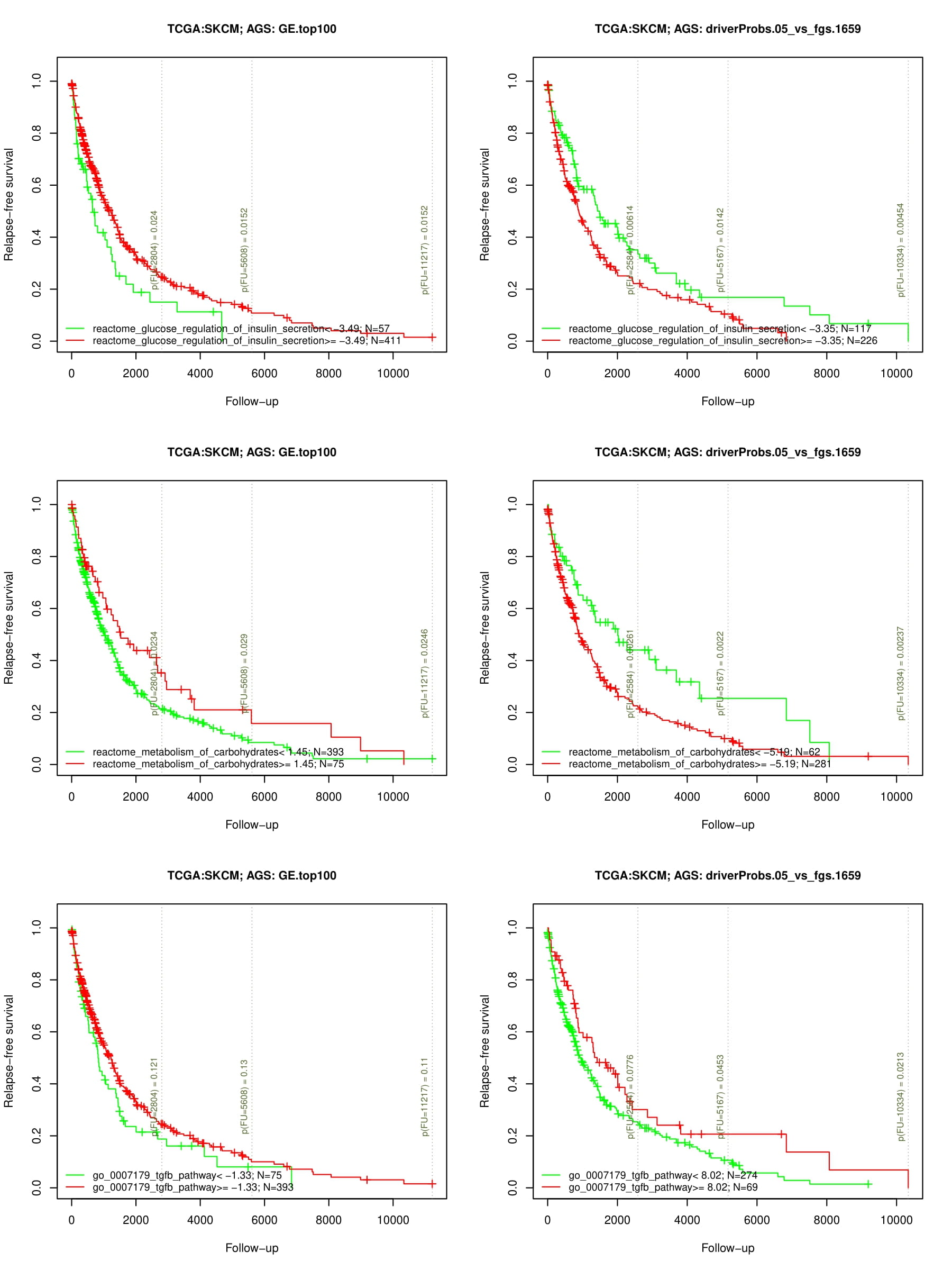

Supplement: Supplementary file 6. [file elife-74010-supp6.zip › SupplementaryFile6-46.jpg]

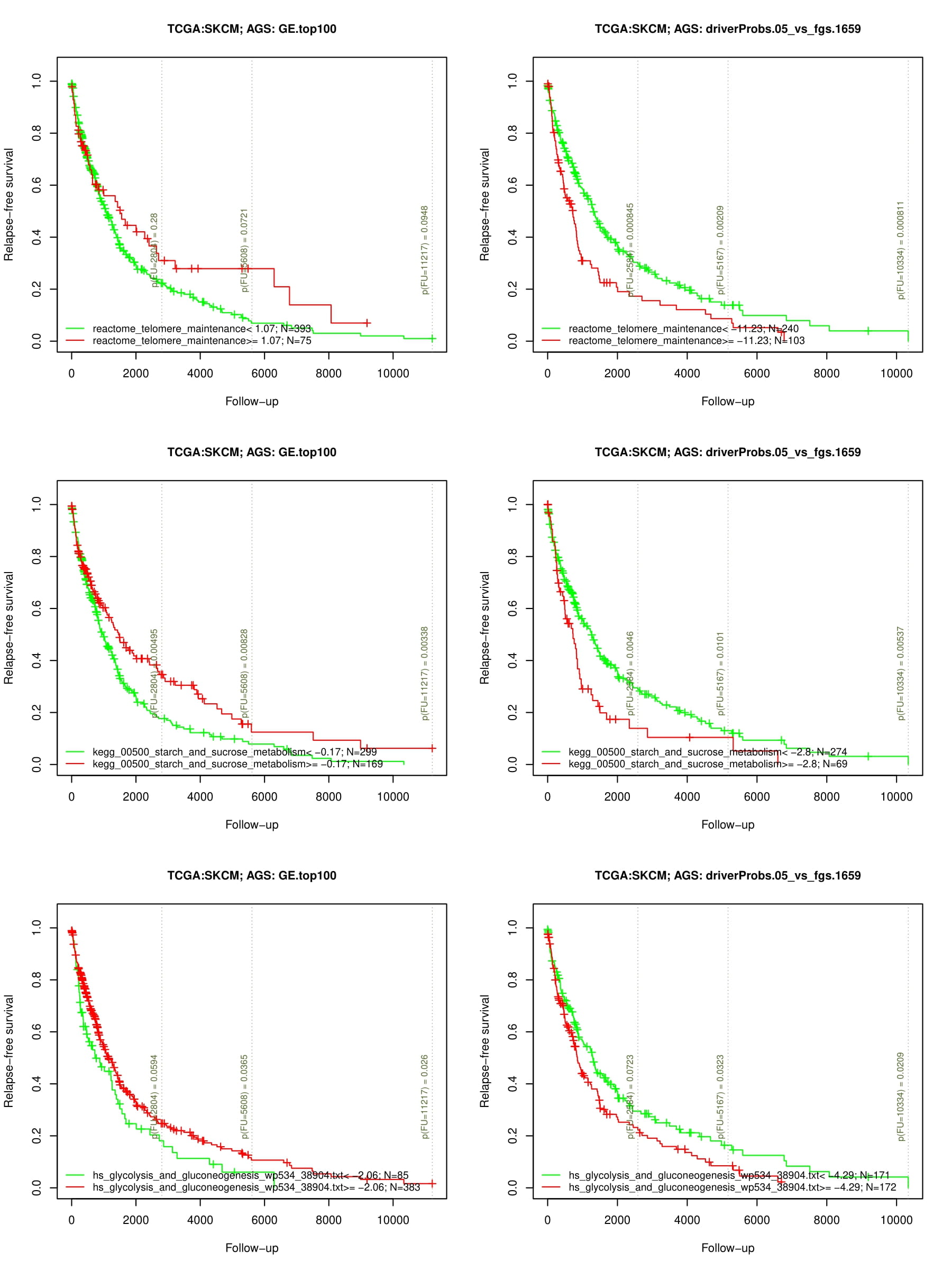

Supplement: Supplementary file 6. [file elife-74010-supp6.zip › SupplementaryFile6-47.jpg]

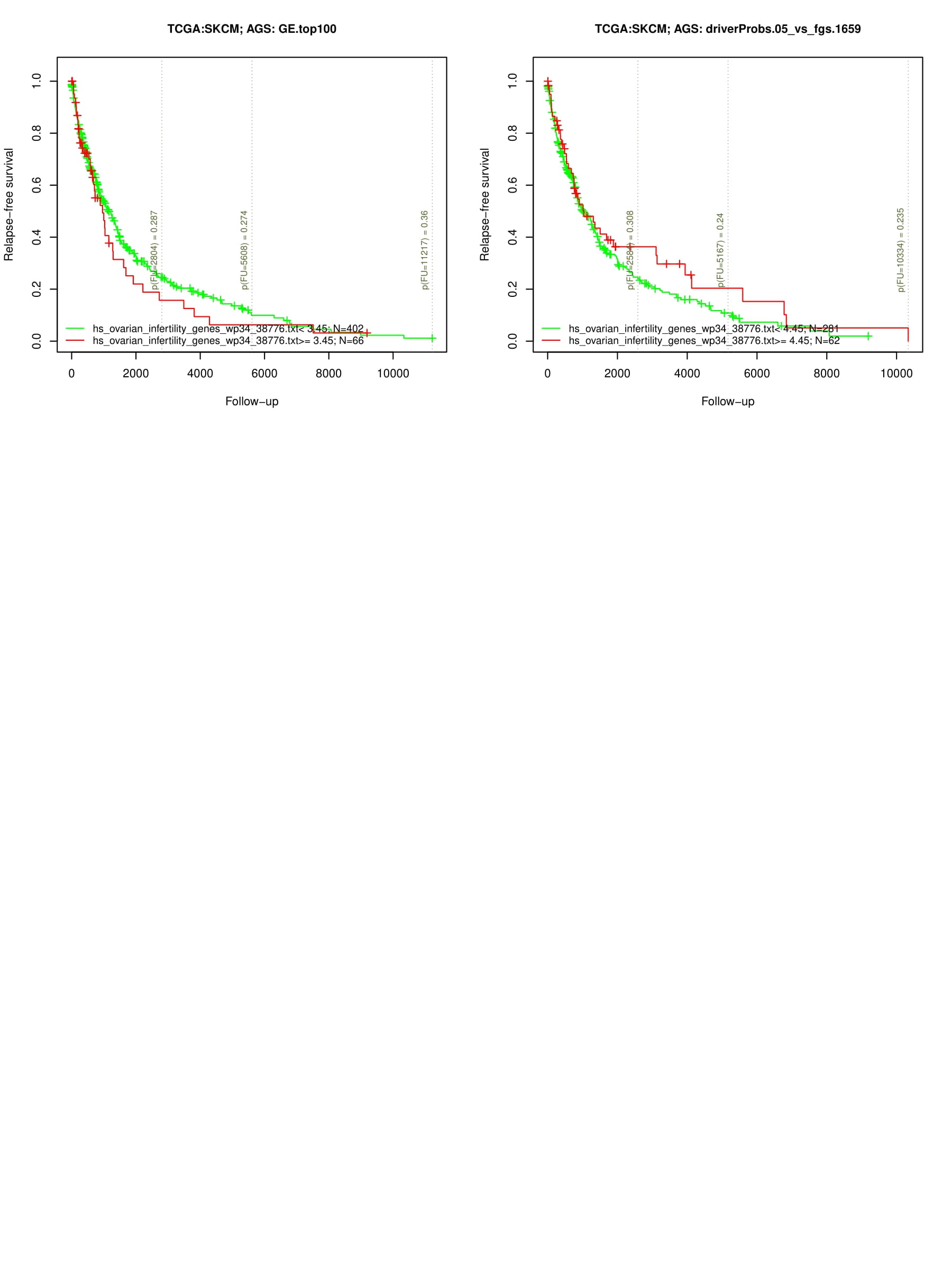

Supplement: Supplementary file 6. [file elife-74010-supp6.zip › SupplementaryFile6-48.jpg]

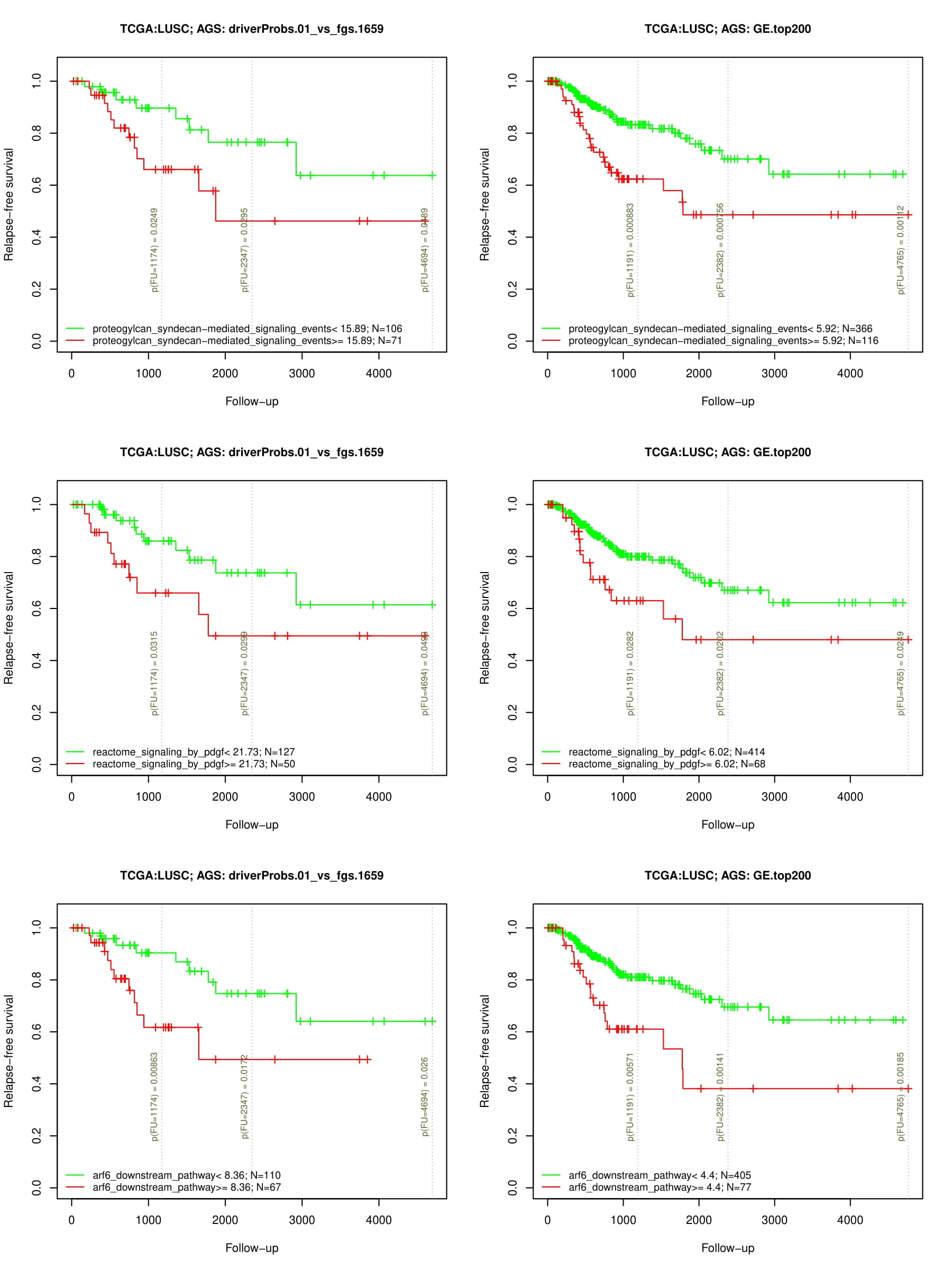

Supplement: Supplementary file 6. [file elife-74010-supp6.zip › SupplementaryFile6-49.jpg]

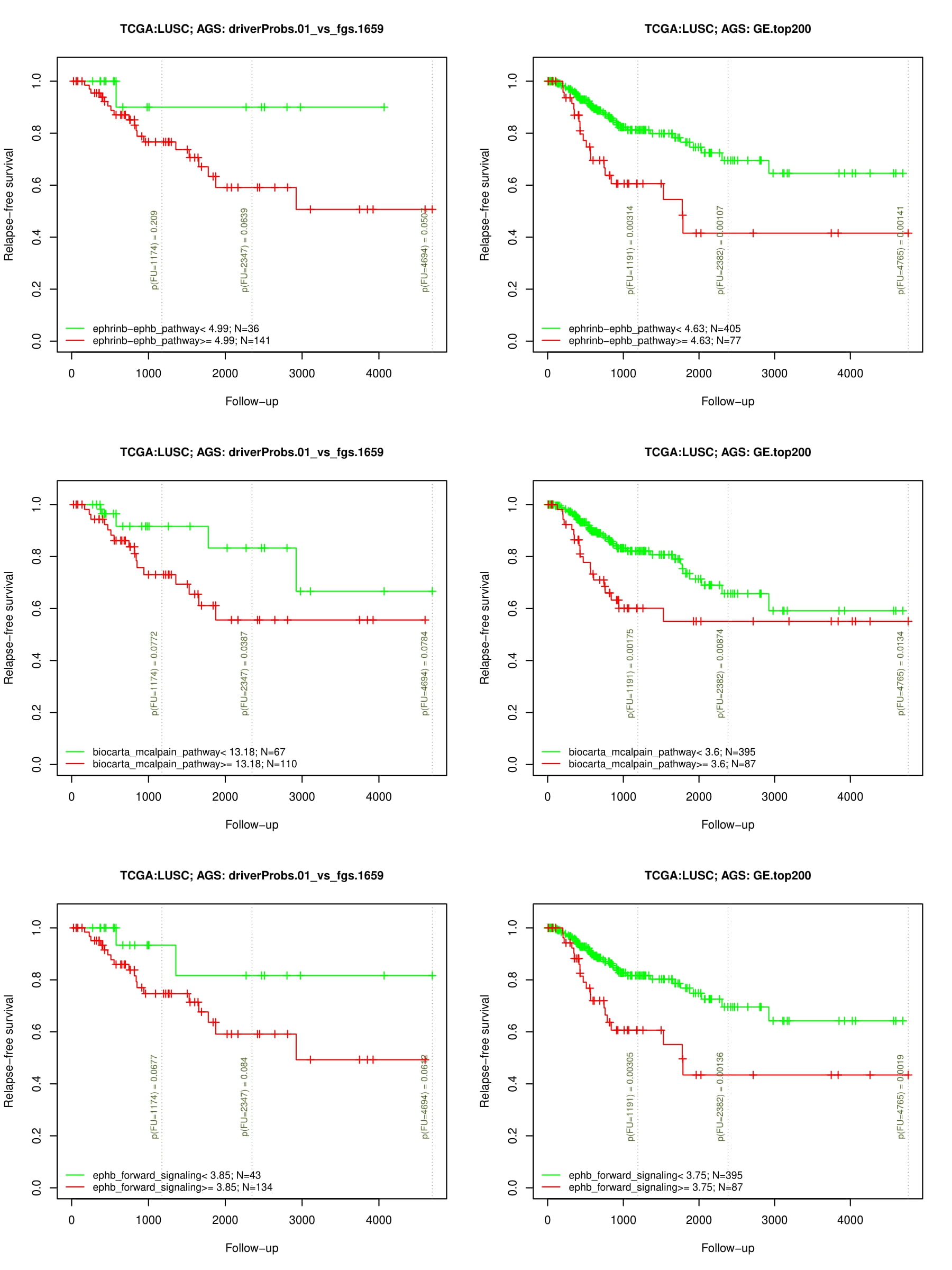

Supplement: Supplementary file 6. [file elife-74010-supp6.zip › SupplementaryFile6-50.jpg]

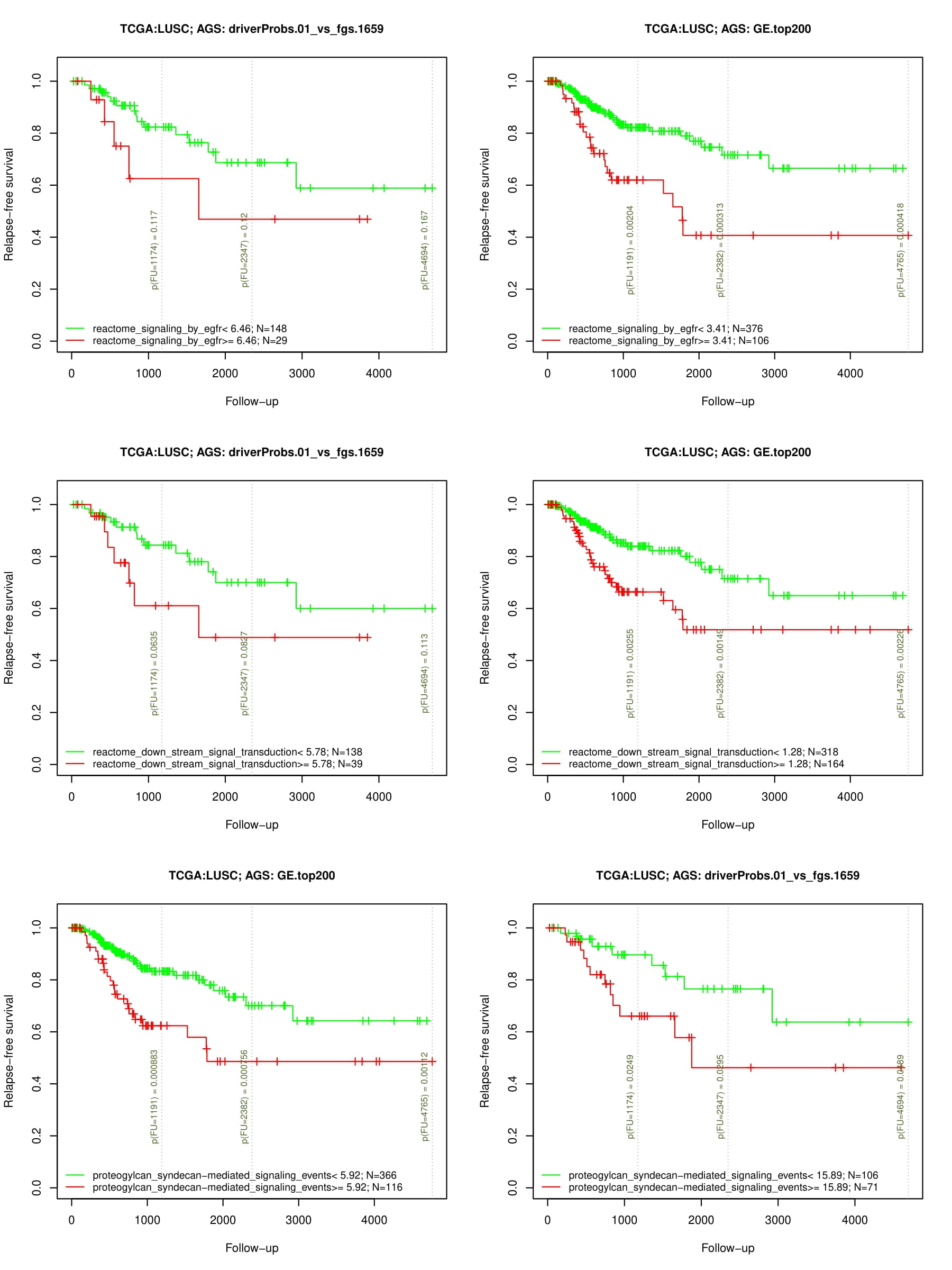

Supplement: Supplementary file 6. [file elife-74010-supp6.zip › SupplementaryFile6-51.jpg]

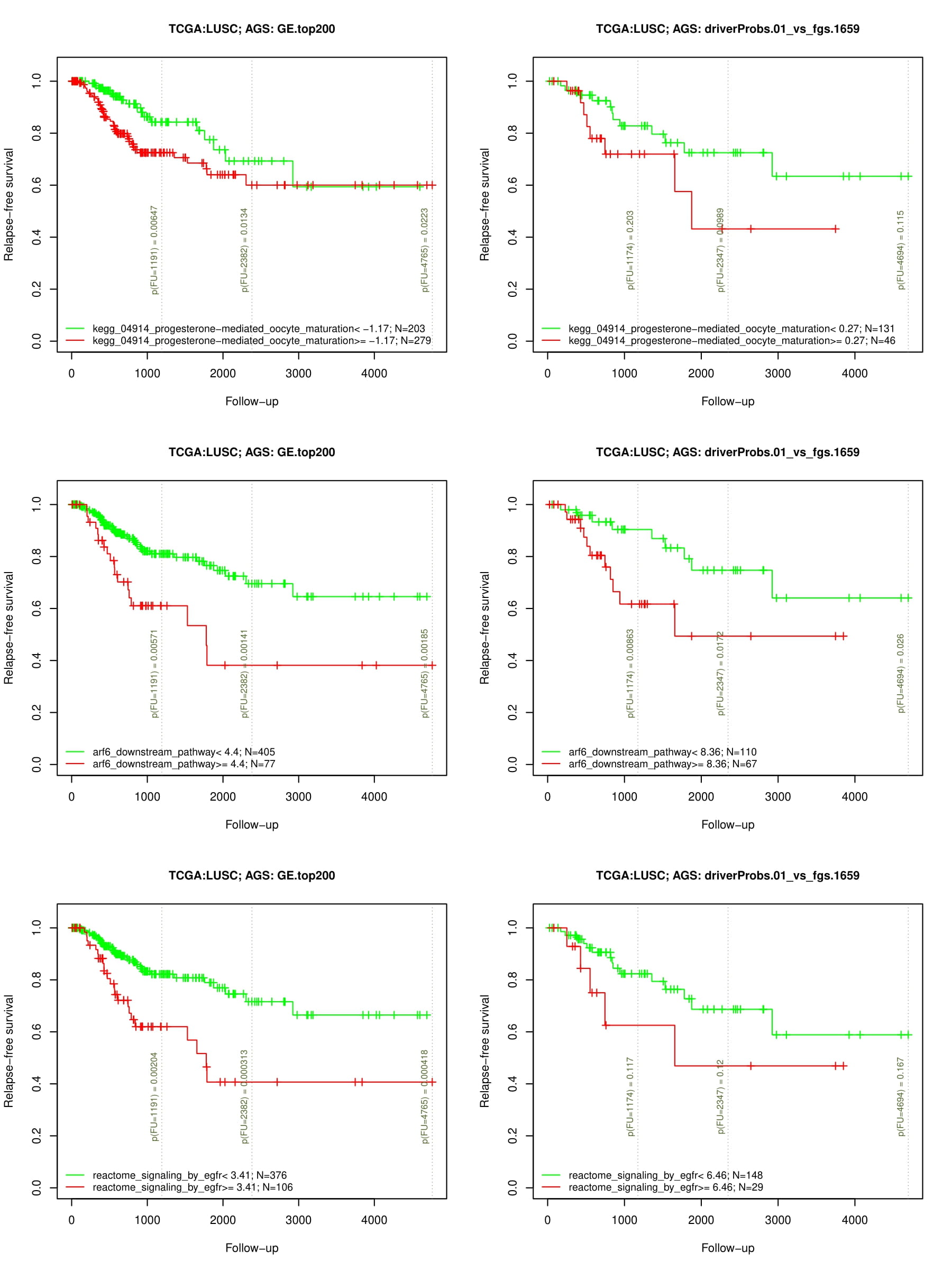

Supplement: Supplementary file 6. [file elife-74010-supp6.zip › SupplementaryFile6-52.jpg]

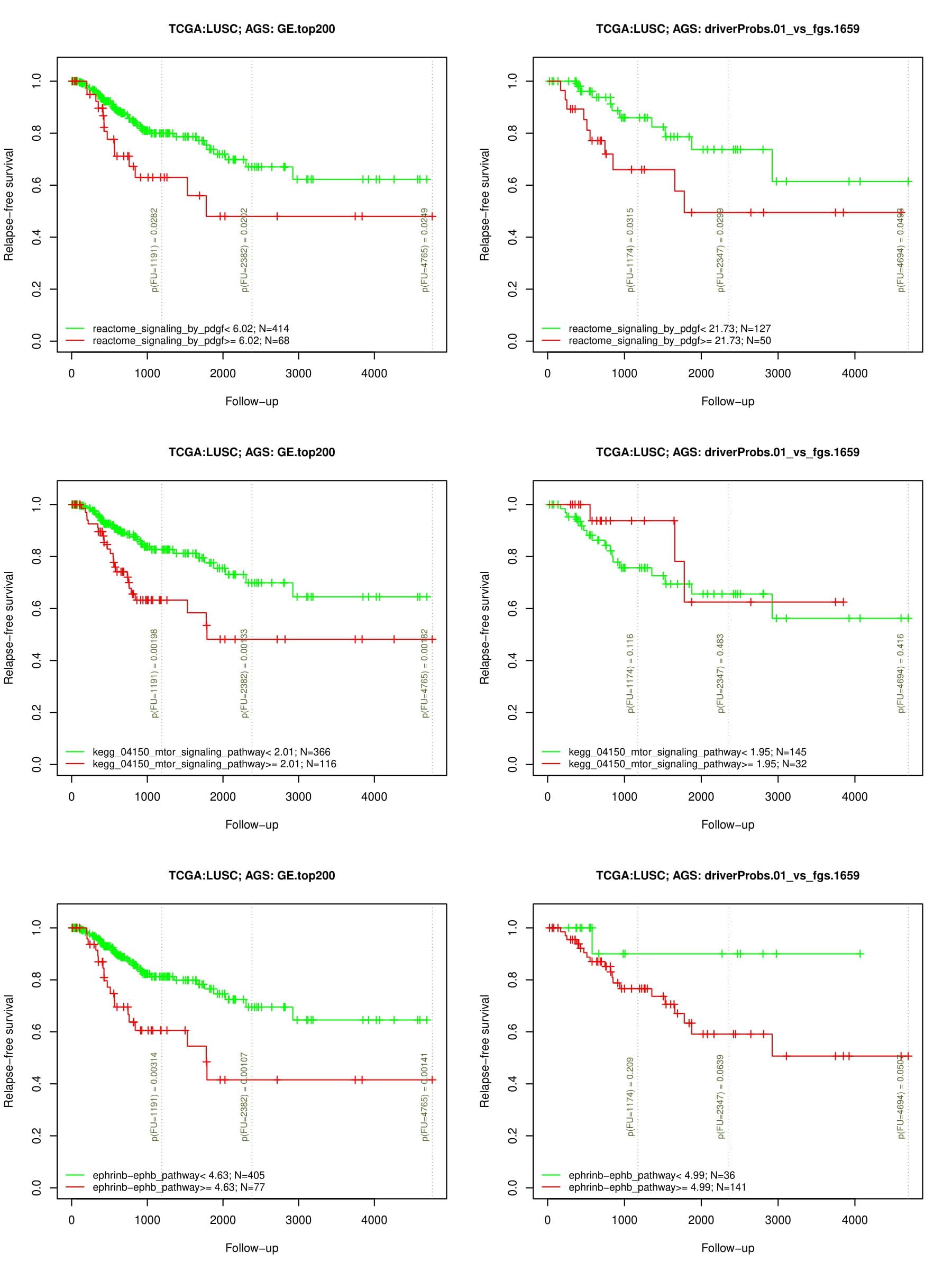

Supplement: Supplementary file 6. [file elife-74010-supp6.zip › SupplementaryFile6-53.jpg]

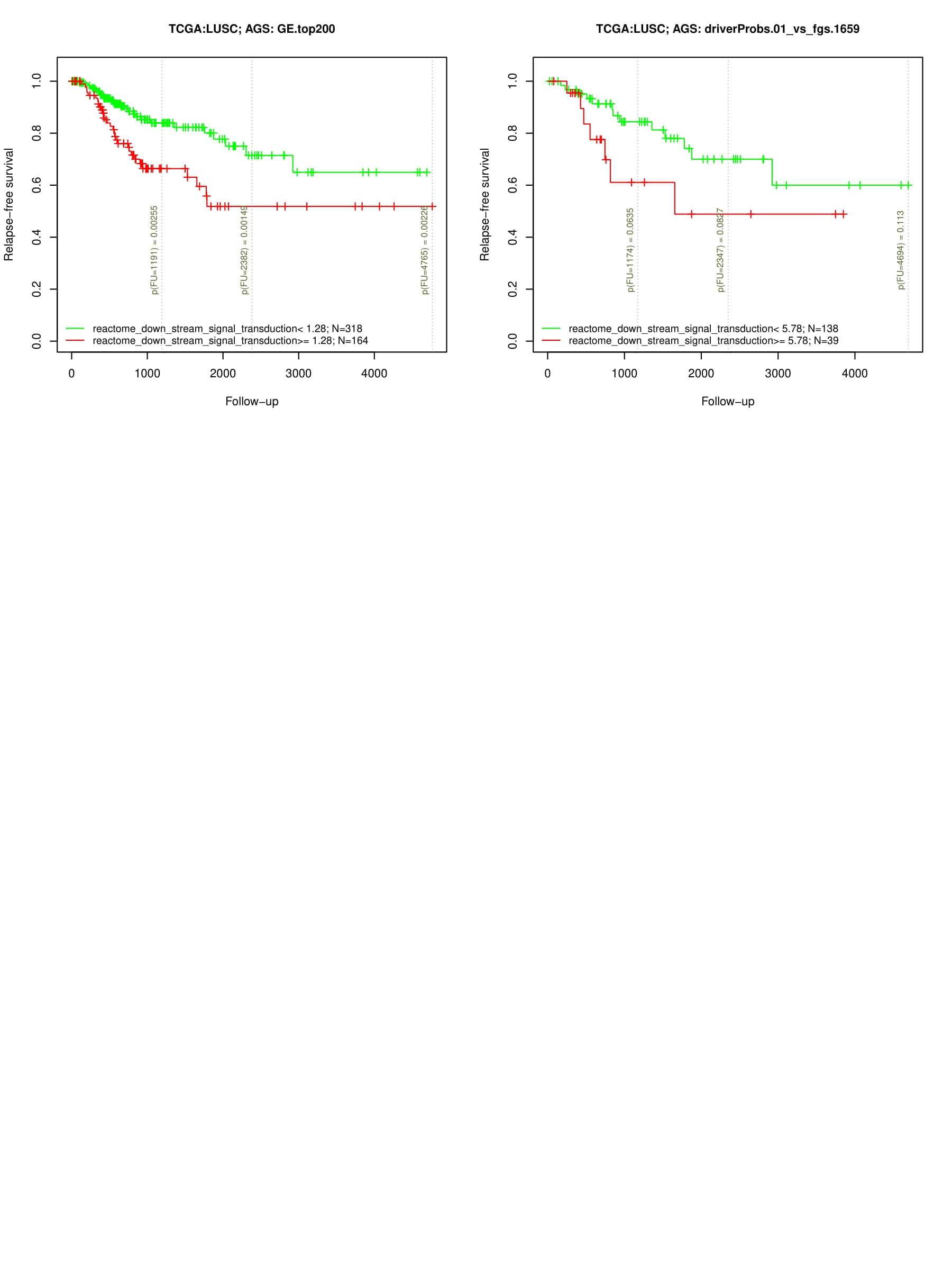

Supplement: Supplementary file 6. [file elife-74010-supp6.zip › SupplementaryFile6-54.jpg]

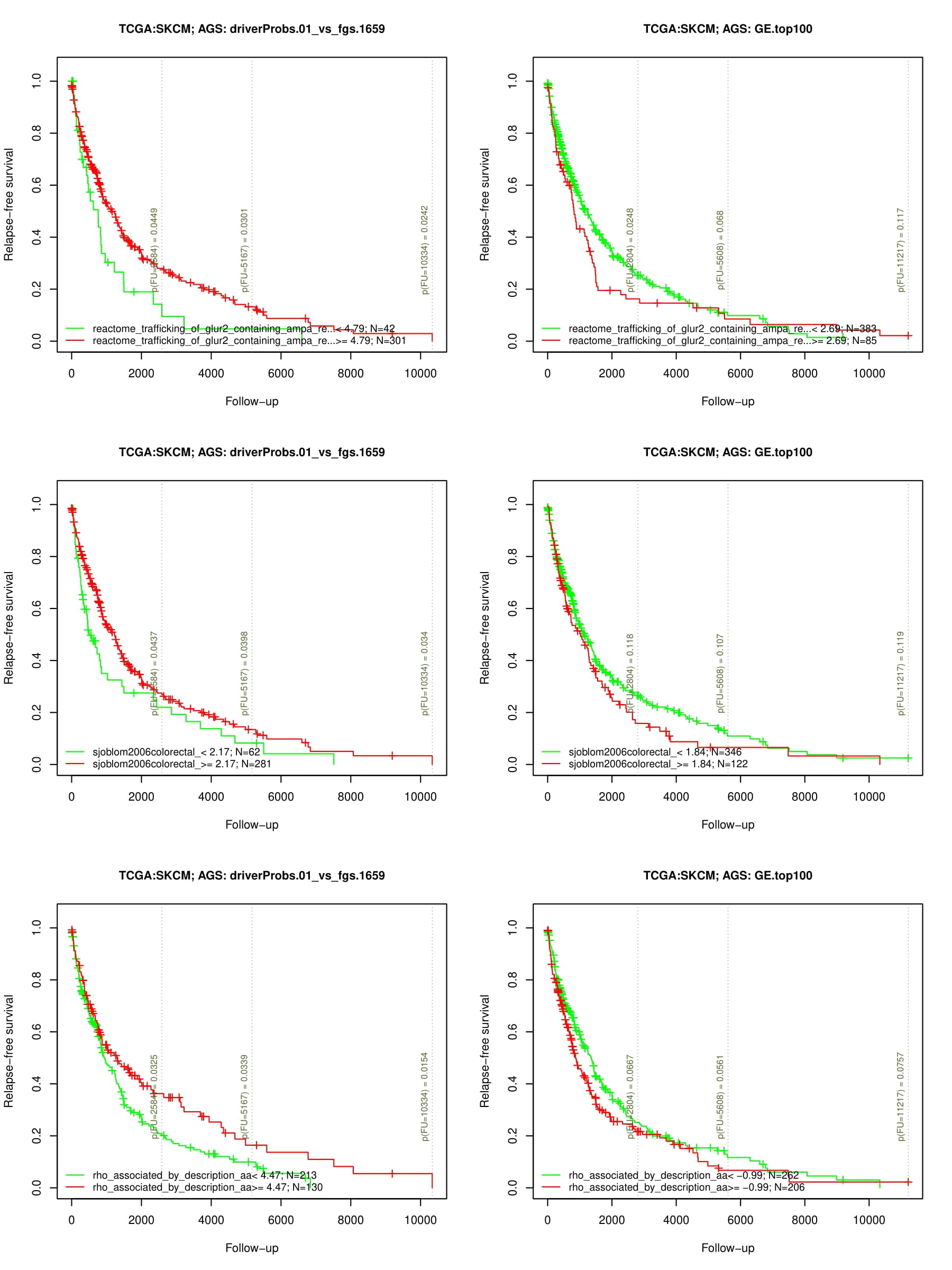

Supplement: Supplementary file 6. [file elife-74010-supp6.zip › SupplementaryFile6-55.jpg]

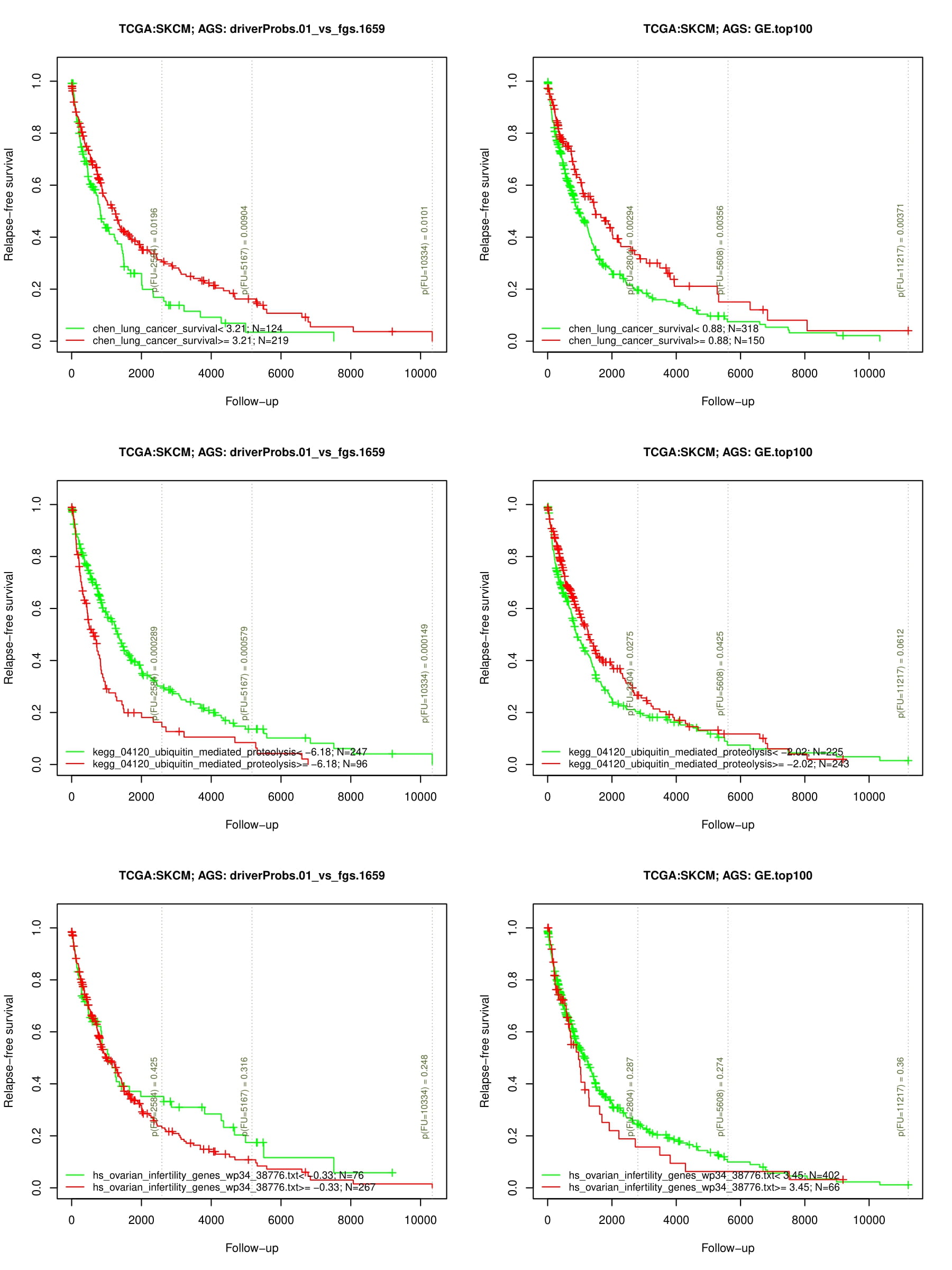

Supplement: Supplementary file 6. [file elife-74010-supp6.zip › SupplementaryFile6-56.jpg]

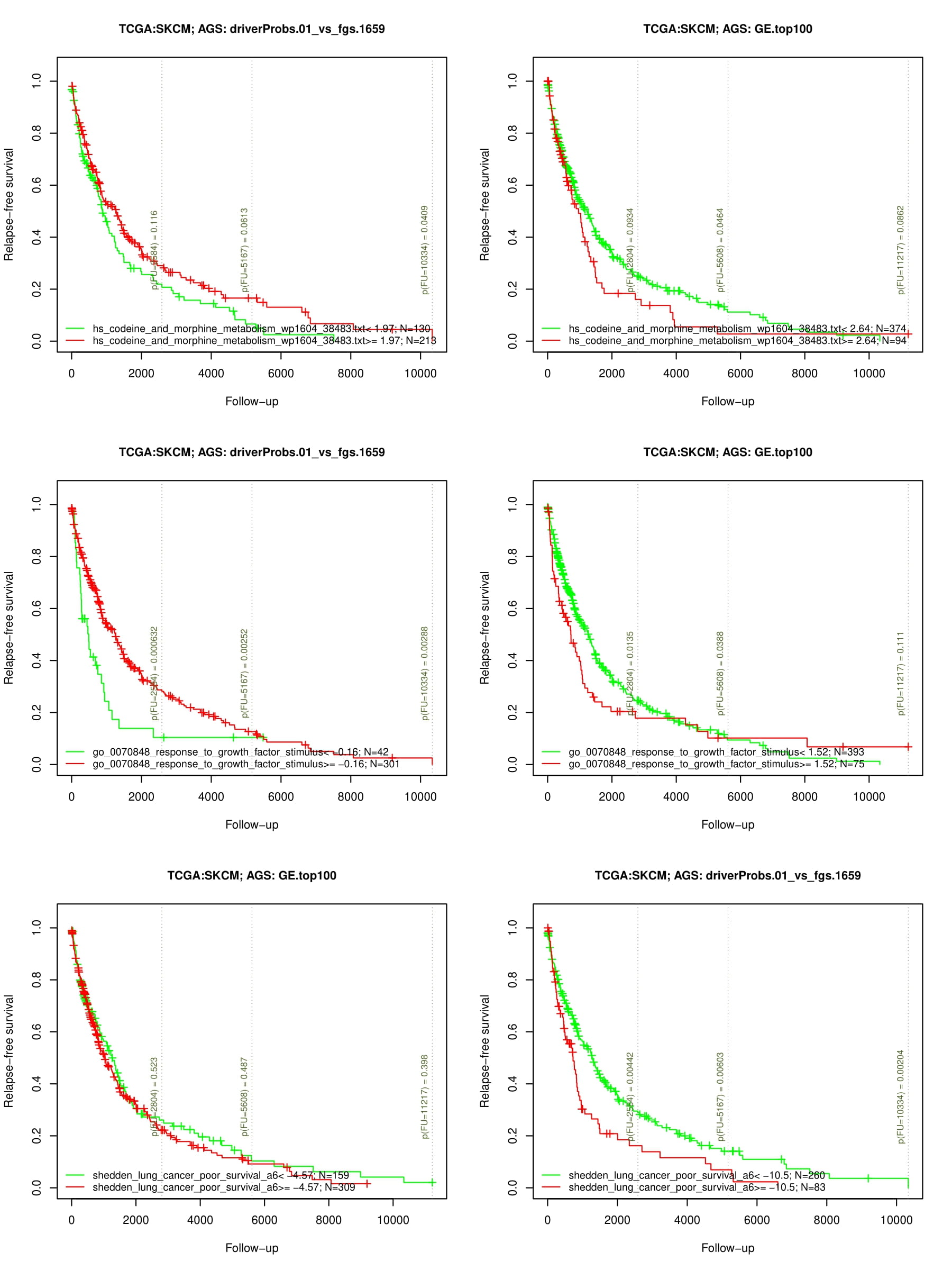

Supplement: Supplementary file 6. [file elife-74010-supp6.zip › SupplementaryFile6-57.jpg]

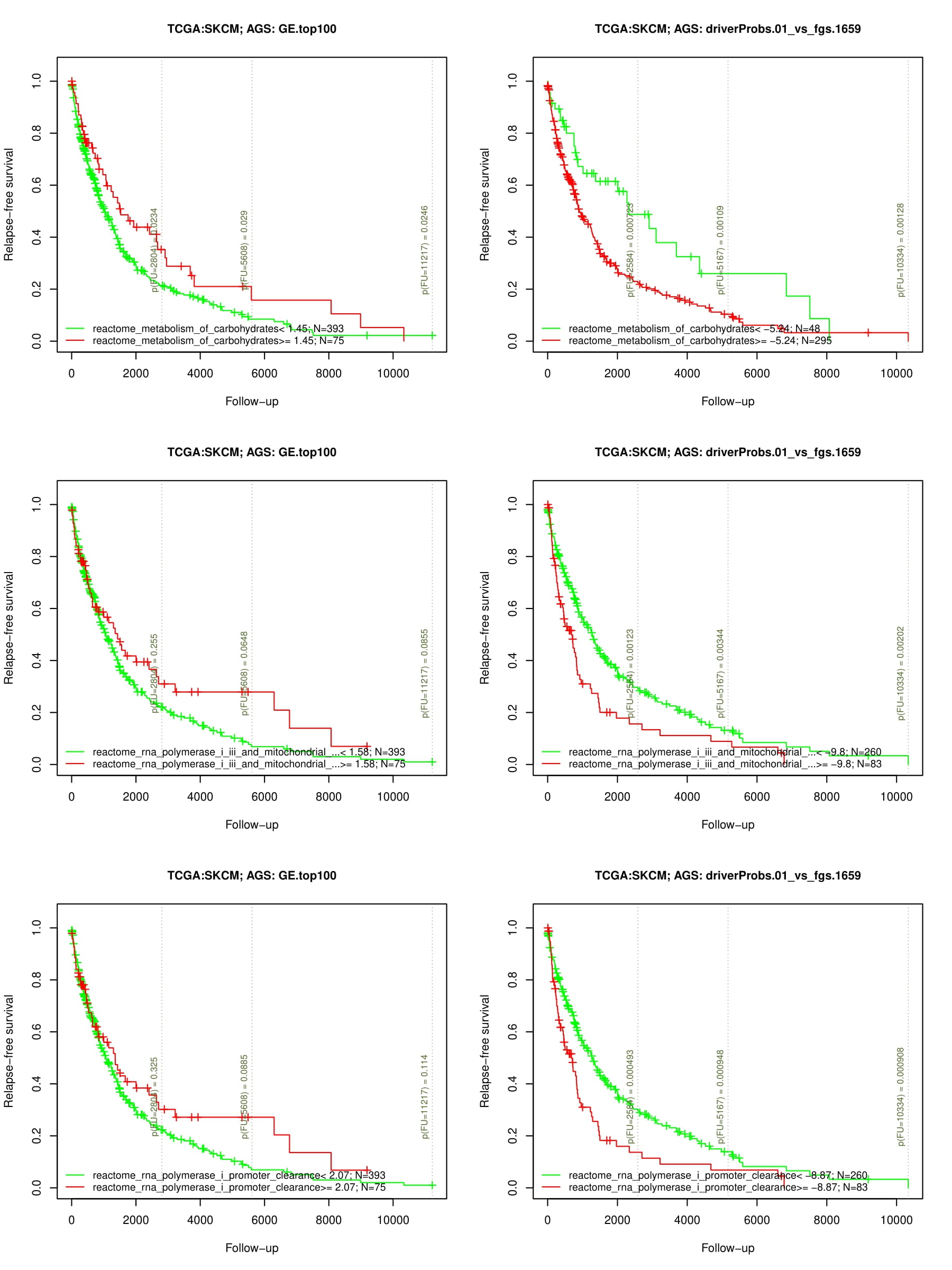

Supplement: Supplementary file 6. [file elife-74010-supp6.zip › SupplementaryFile6-58.jpg]

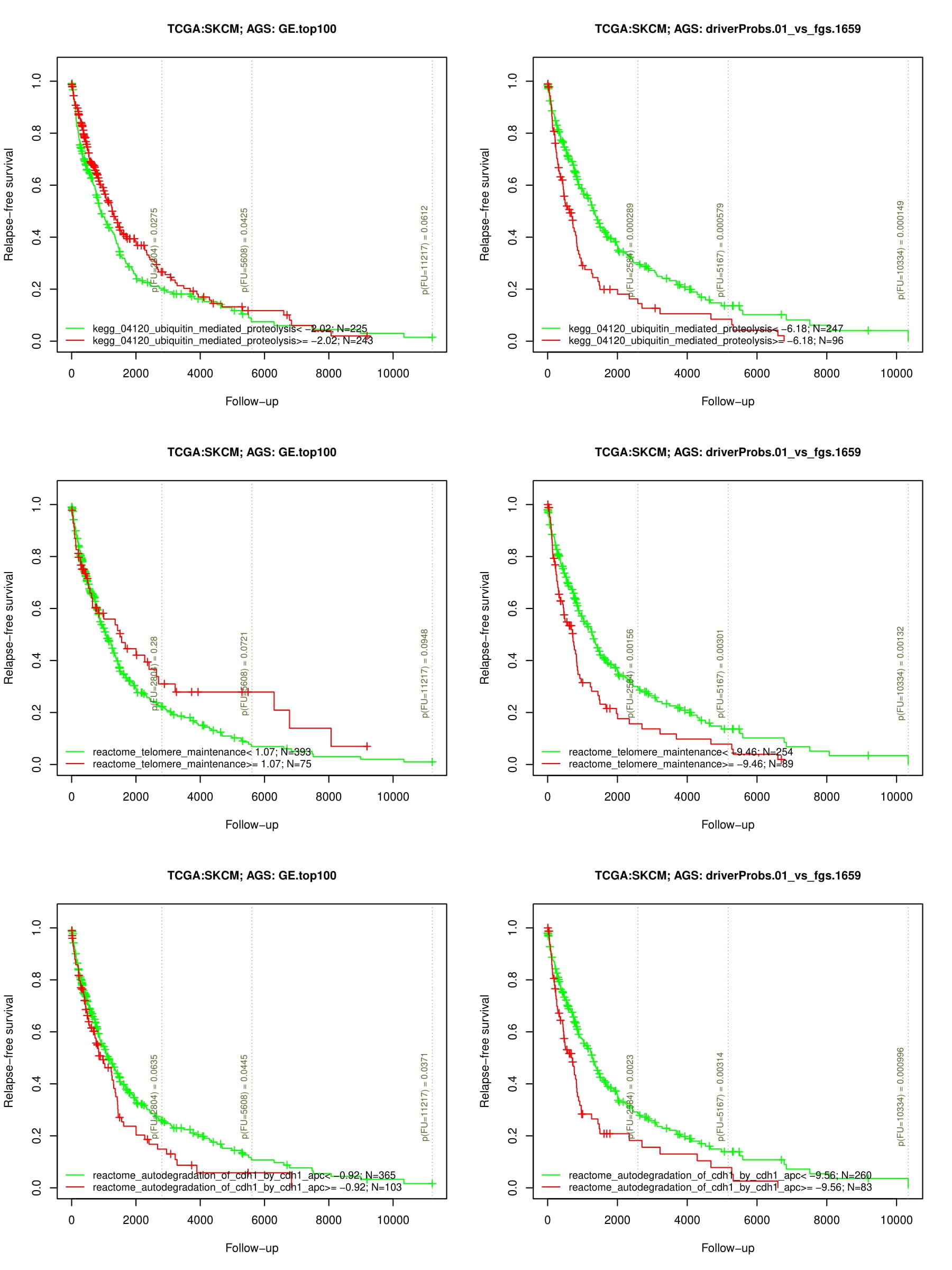

Supplement: Supplementary file 6. [file elife-74010-supp6.zip › SupplementaryFile6-59.jpg]

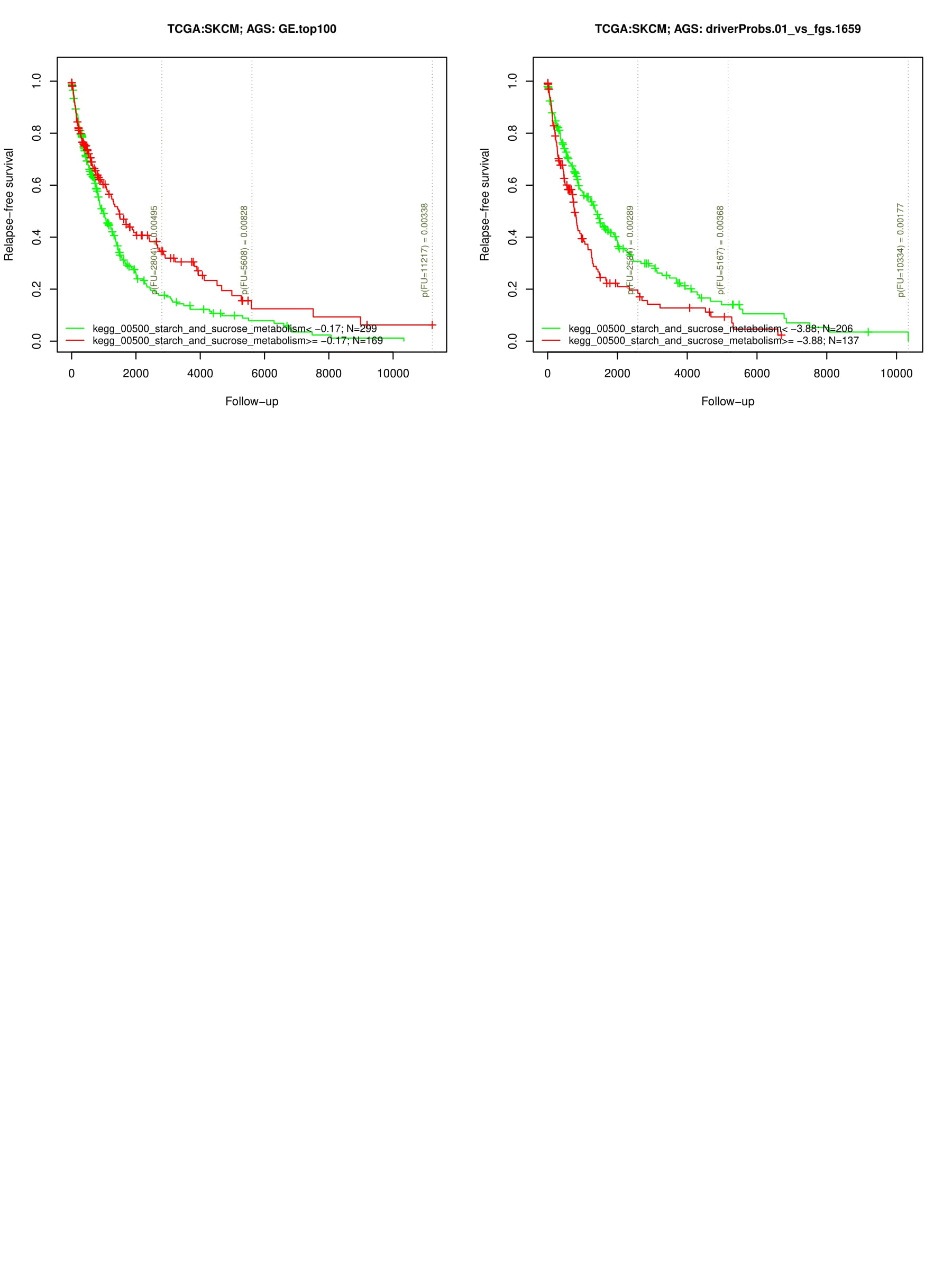

Supplement: Supplementary file 6. [file elife-74010-supp6.zip › SupplementaryFile6-60.jpg]

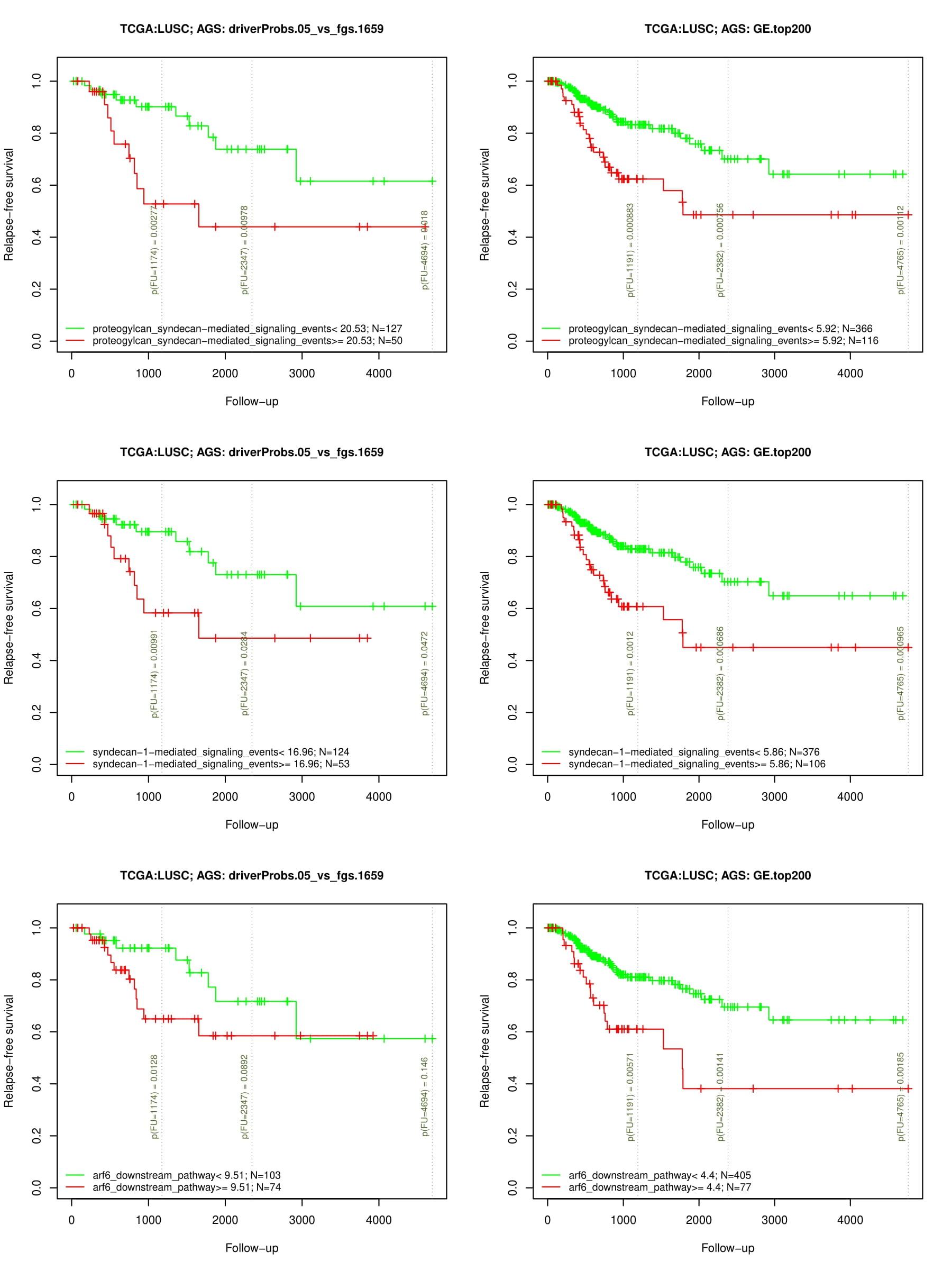

Supplement: Supplementary file 6. [file elife-74010-supp6.zip › SupplementaryFile6-61.jpg]

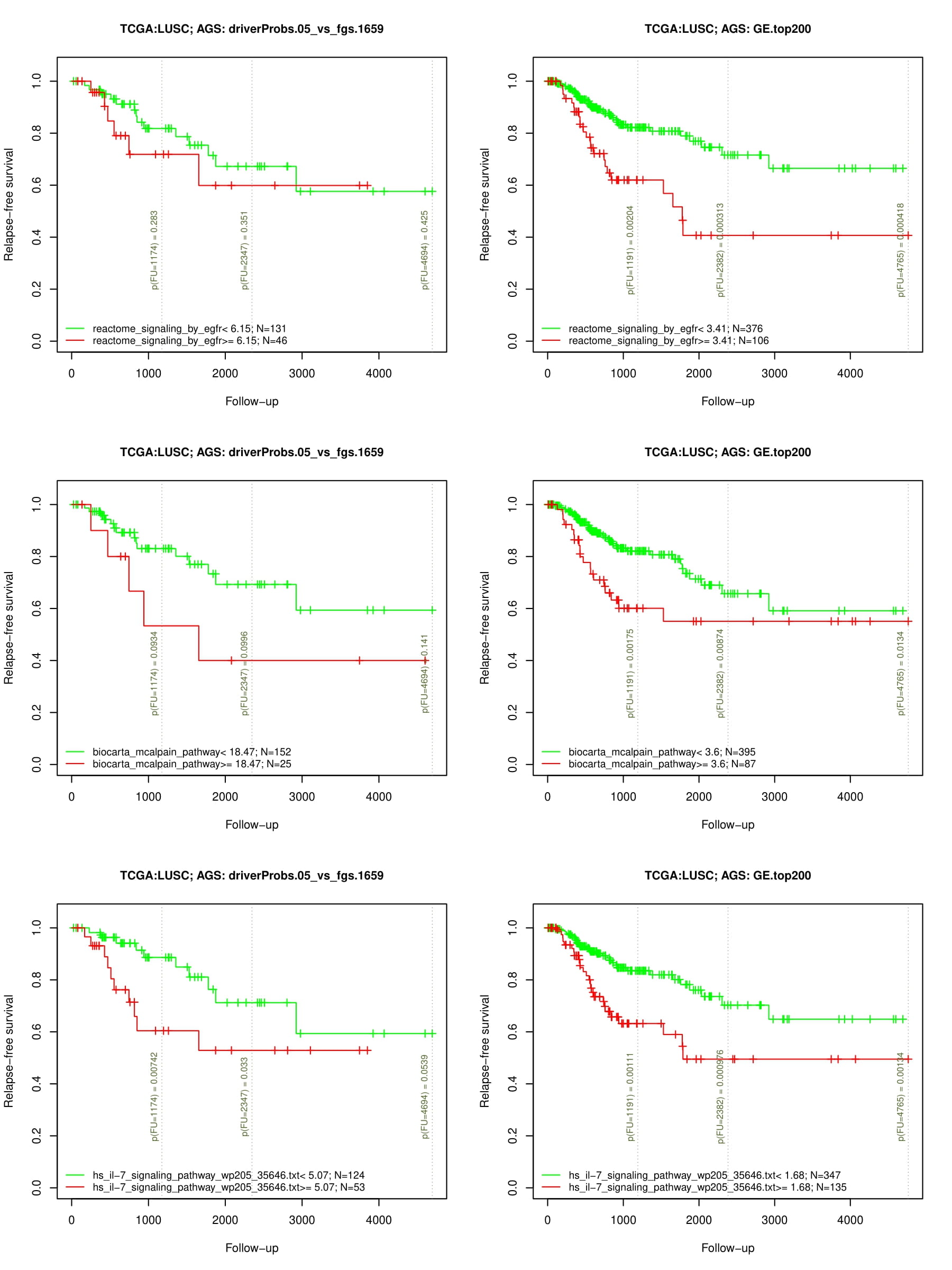

Supplement: Supplementary file 6. [file elife-74010-supp6.zip › SupplementaryFile6-62.jpg]

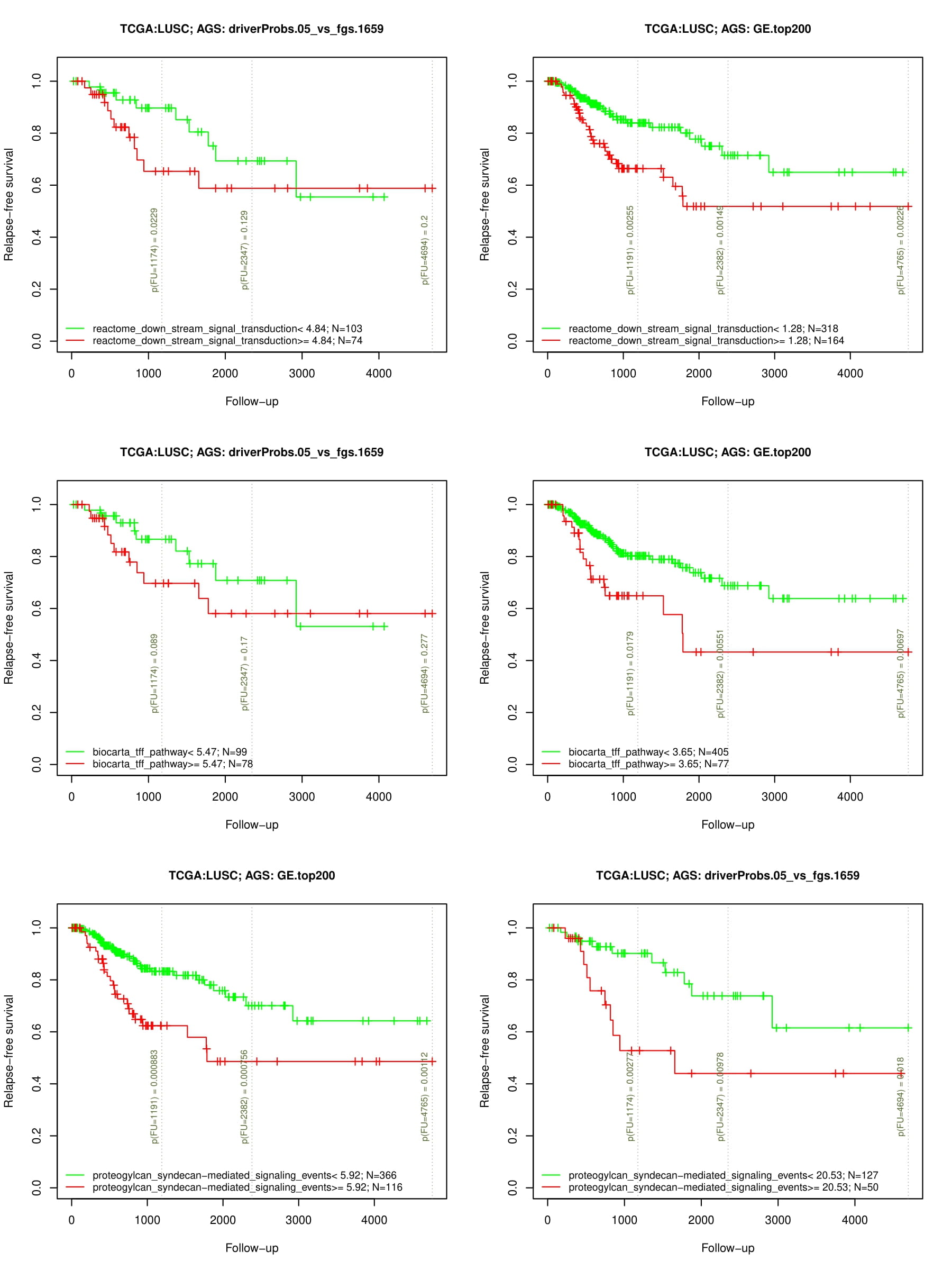

Supplement: Supplementary file 6. [file elife-74010-supp6.zip › SupplementaryFile6-63.jpg]

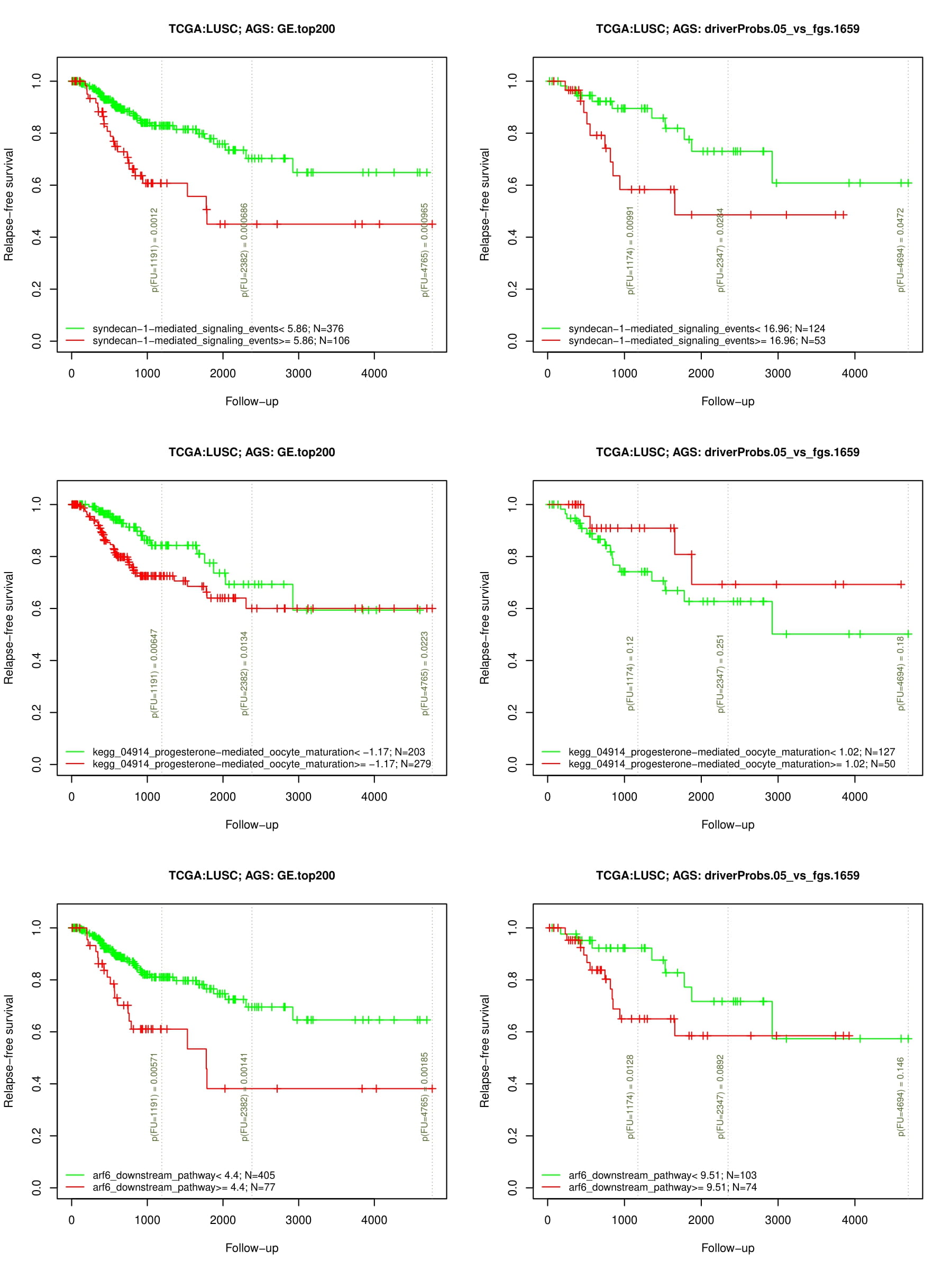

Supplement: Supplementary file 6. [file elife-74010-supp6.zip › SupplementaryFile6-64.jpg]

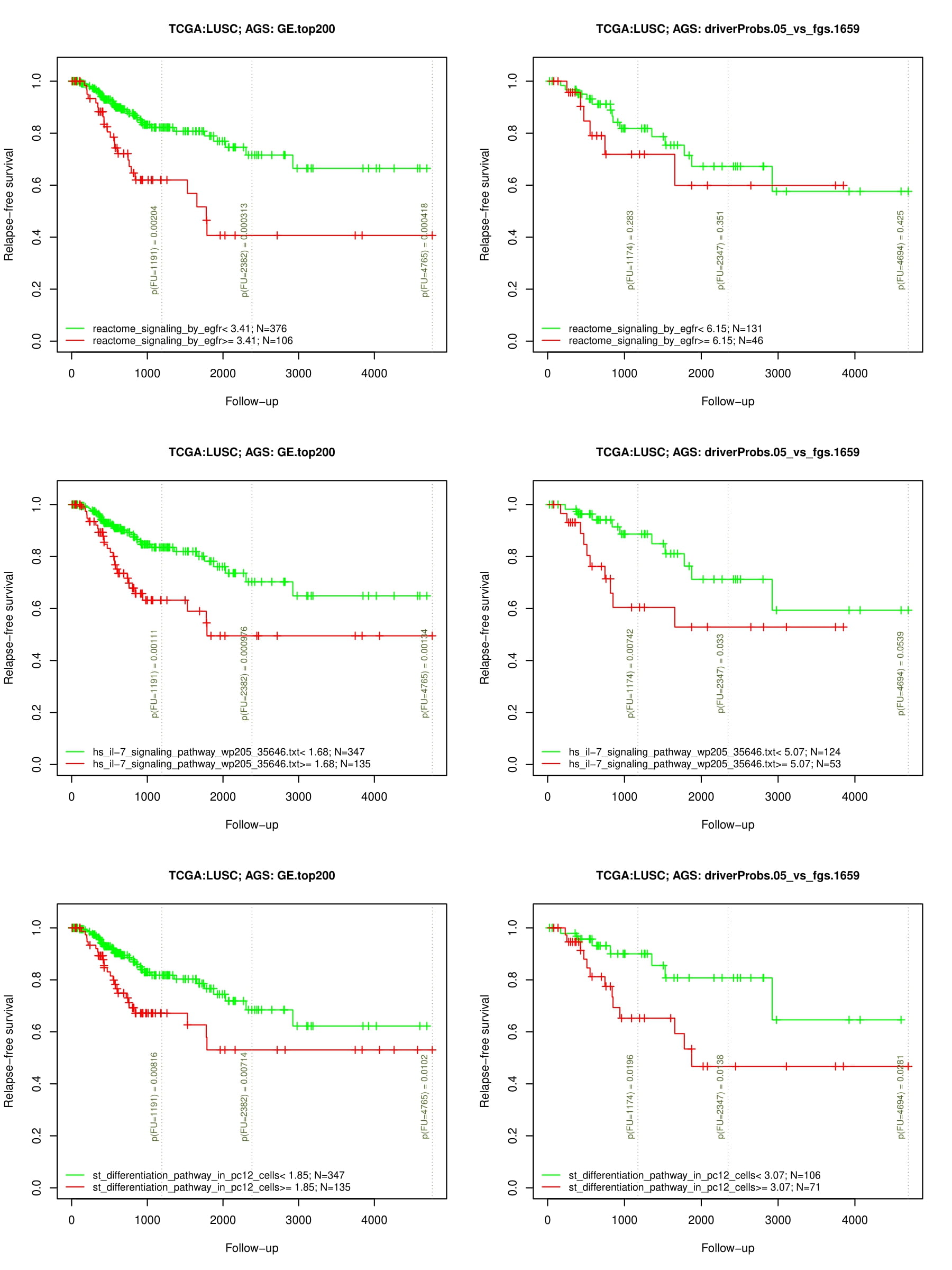

Supplement: Supplementary file 6. [file elife-74010-supp6.zip › SupplementaryFile6-65.jpg]

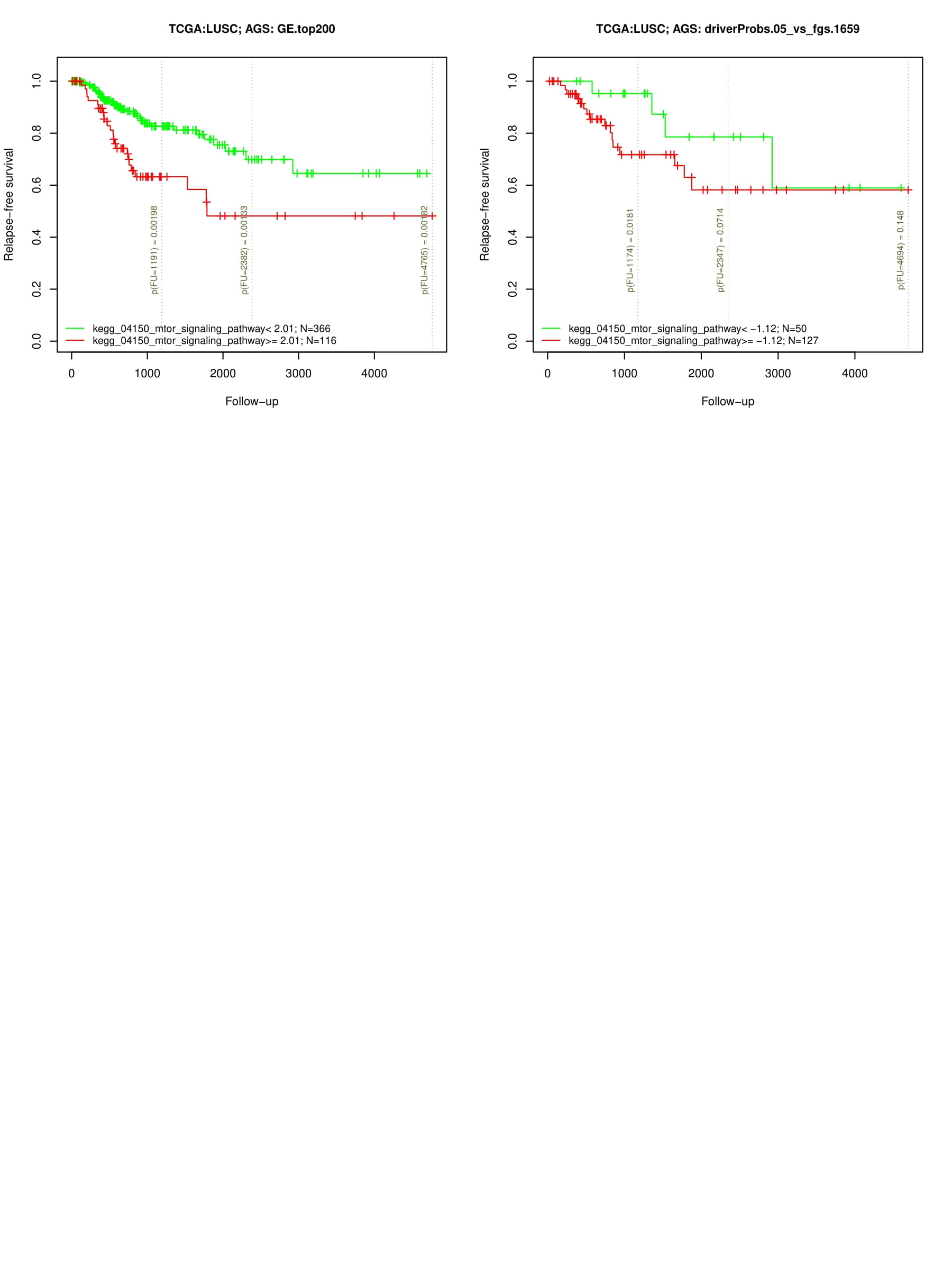

Supplement: Supplementary file 6. [file elife-74010-supp6.zip › SupplementaryFile6-66.jpg]

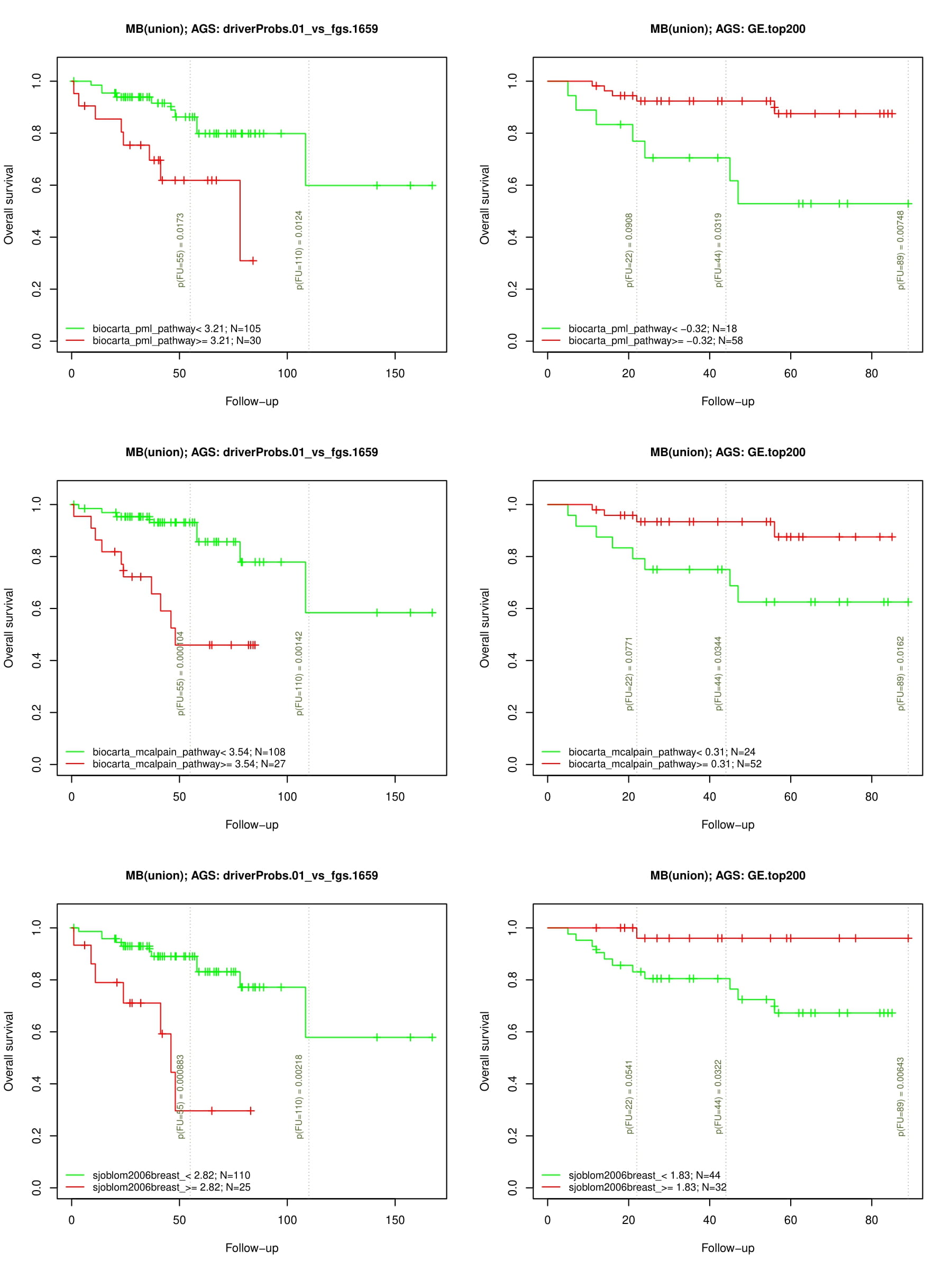

Supplement: Supplementary file 6. [file elife-74010-supp6.zip › SupplementaryFile6-67.jpg]

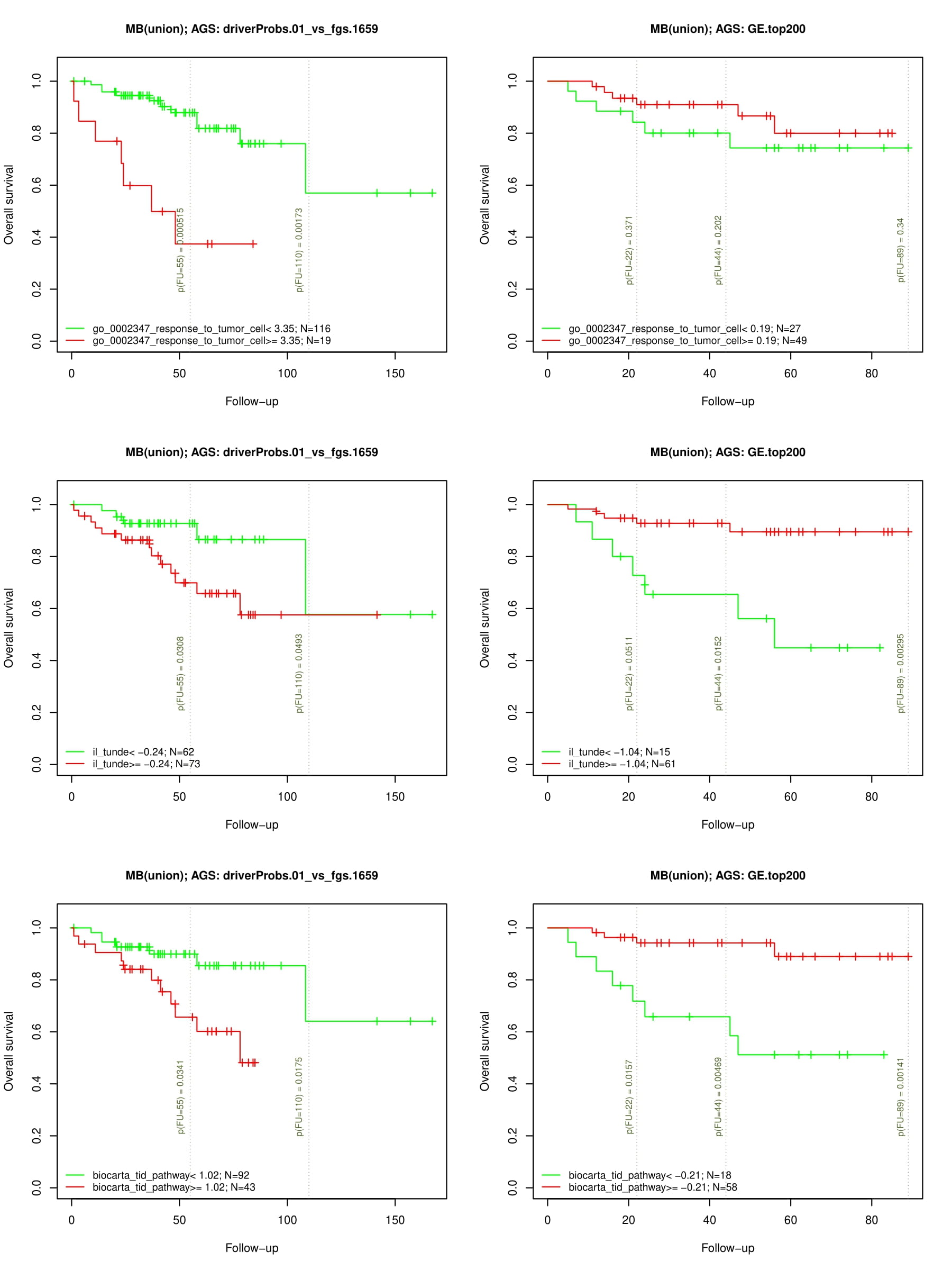

Supplement: Supplementary file 6. [file elife-74010-supp6.zip › SupplementaryFile6-68.jpg]

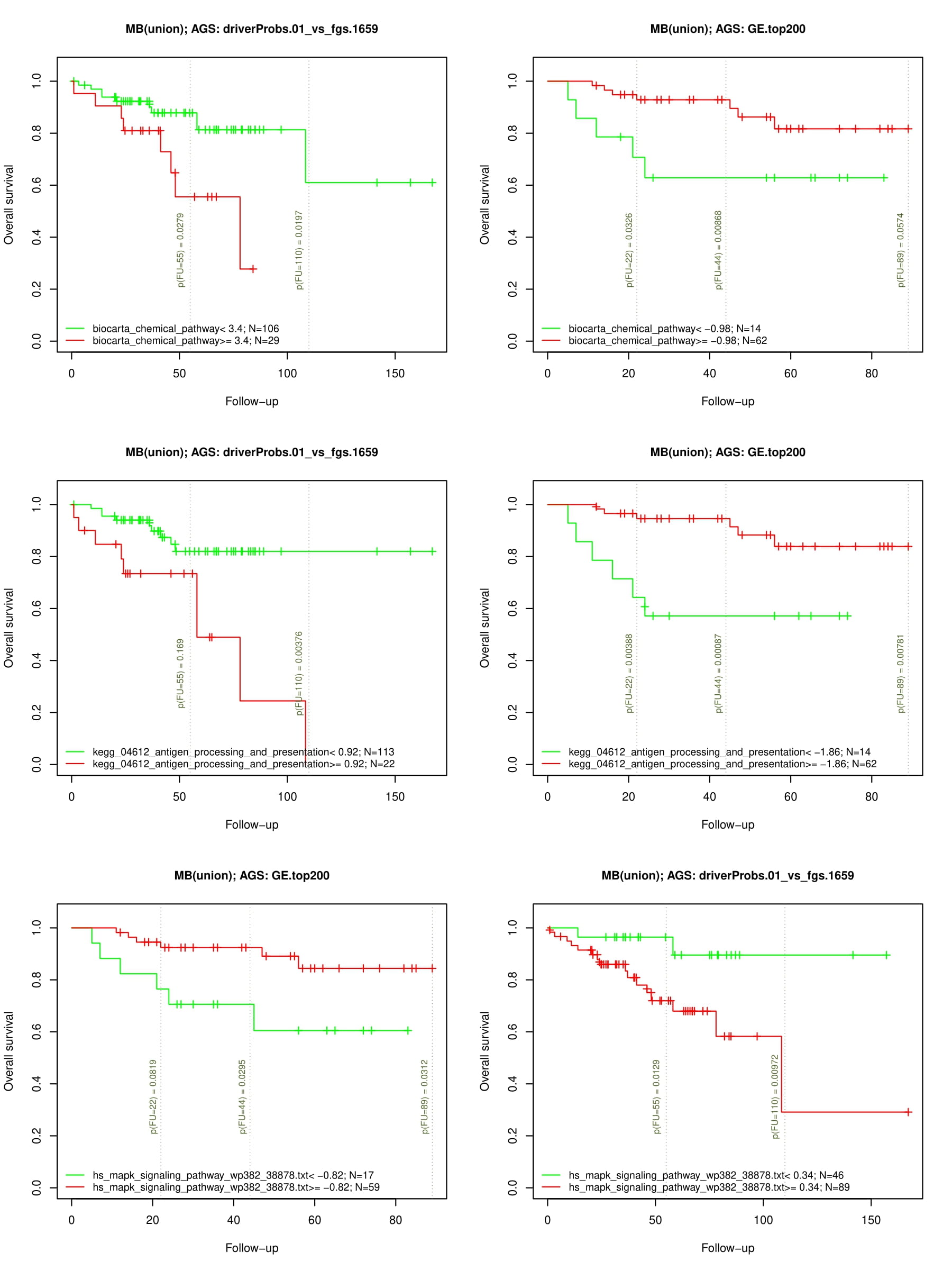

Supplement: Supplementary file 6. [file elife-74010-supp6.zip › SupplementaryFile6-69.jpg]

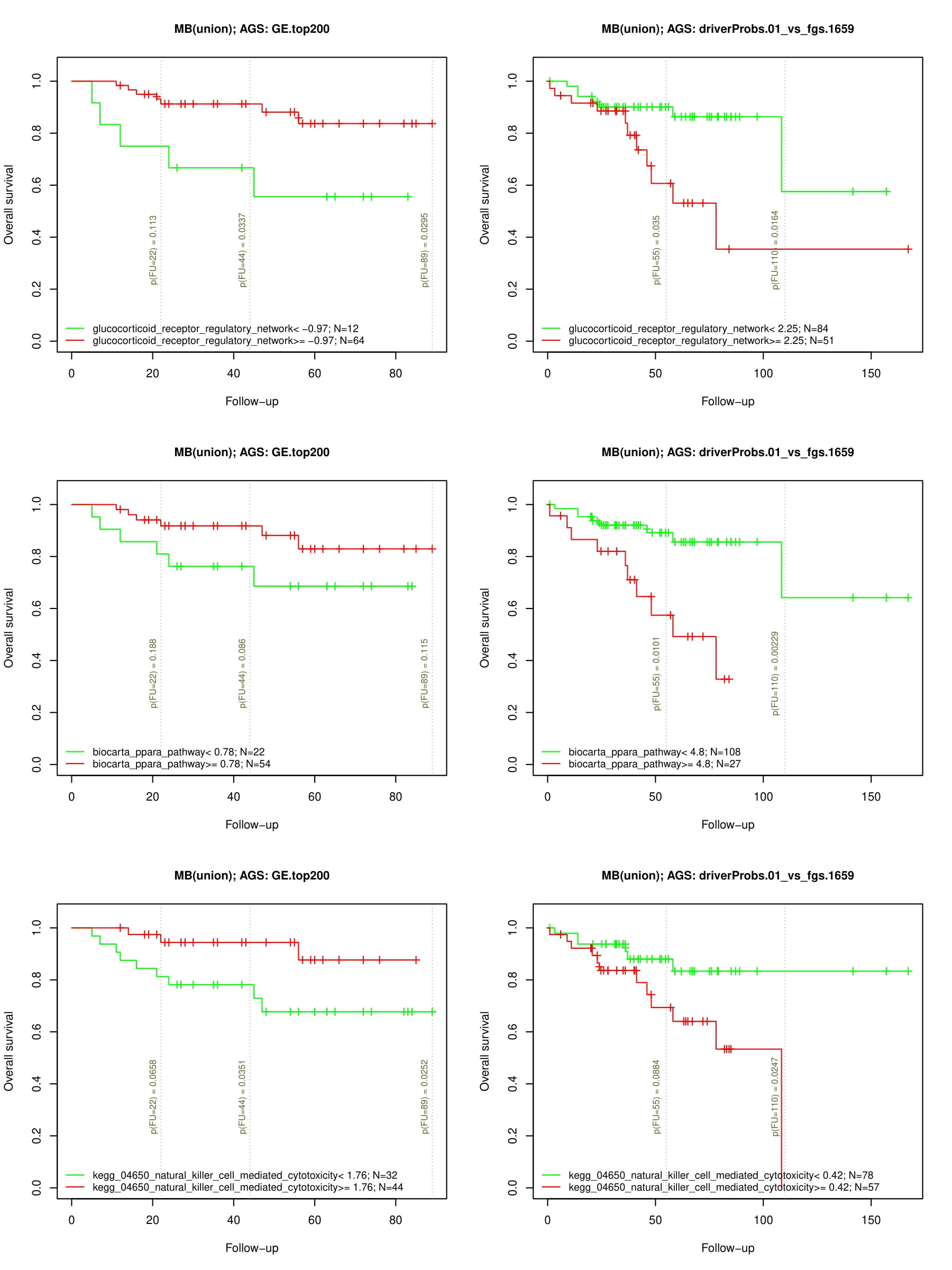

Supplement: Supplementary file 6. [file elife-74010-supp6.zip › SupplementaryFile6-70.jpg]

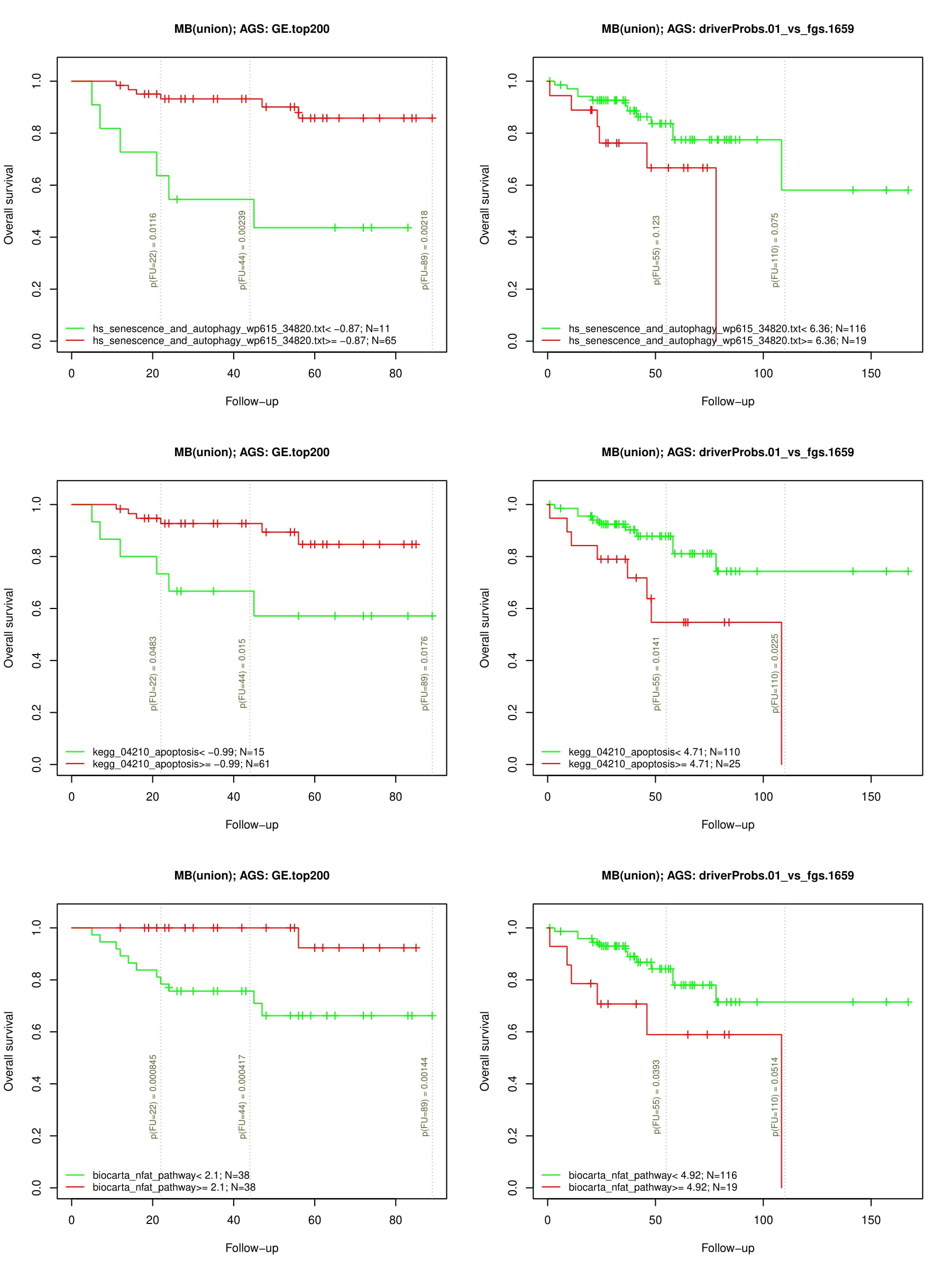

Supplement: Supplementary file 6. [file elife-74010-supp6.zip › SupplementaryFile6-71.jpg]

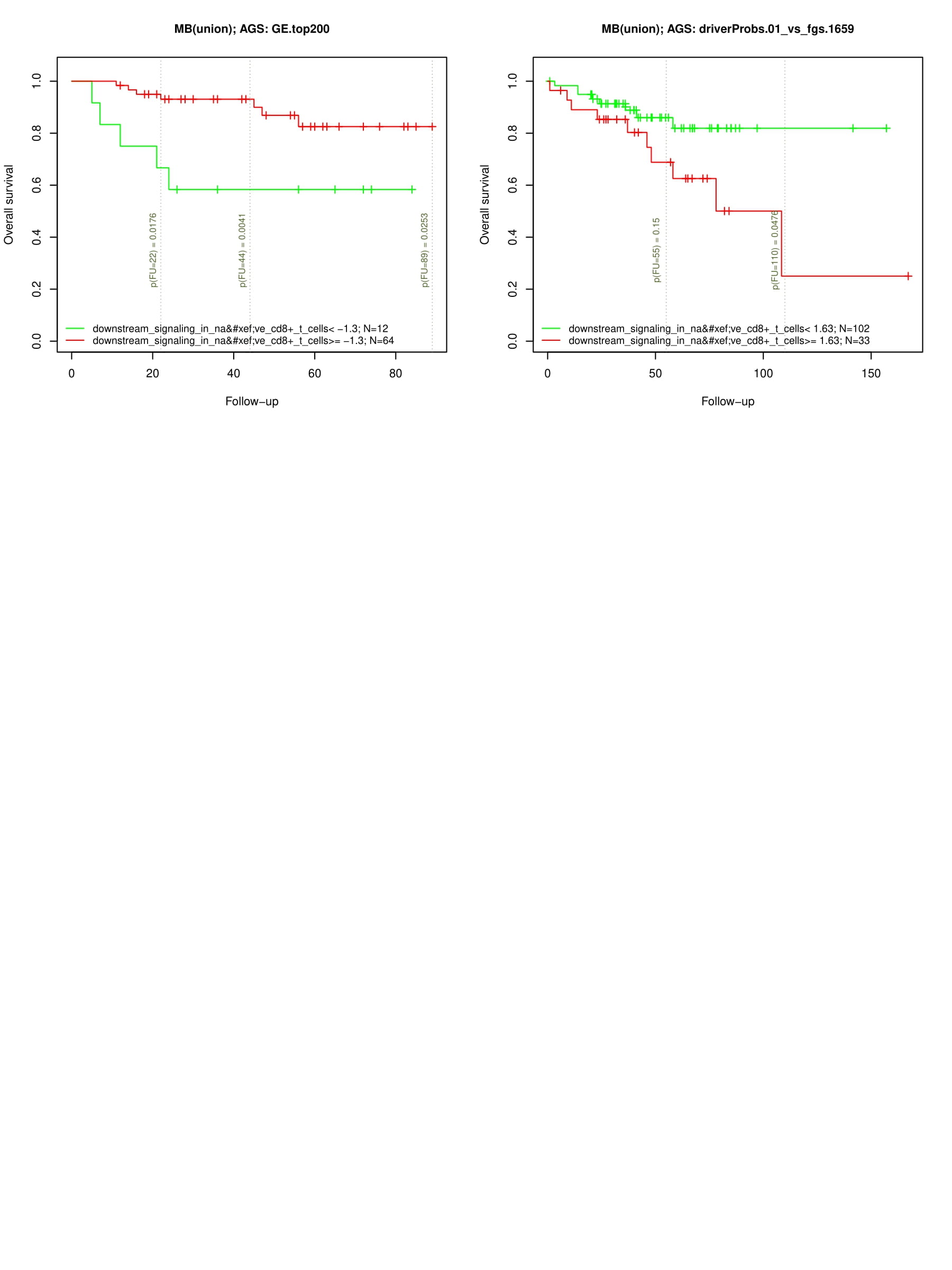

Supplement: Supplementary file 6. [file elife-74010-supp6.zip › SupplementaryFile6-72.jpg]
