# Supplementary material for: Individualized discovery of rare cancer drivers in global network context
Source: eLife. 2022 May 20;11:e74010. doi: 10.7554/eLife.74010 (PMC9159755; doi:10.7554/eLife.74010)
Supplement: Supplementary file 5. — The five pages present relations between MutSet&PathReg q (Y axis) and no. of mutations per cohort, gene length, and normalized mutation frequency, replication rate, and gene expression rates, respectively (X axis). Top left legend: Spearman rank R and Kendall tau represent overall correlations between X and Y coordinates regardless of other factors. Bottom left legend: terms’ significance in 4-way linear models. Colored points: genes suggested as potential artifacts in literature; those receiving q<0.05 are text-labeled. [file elife-74010-supp5.zip › SupplementaryFigure5.pdf]

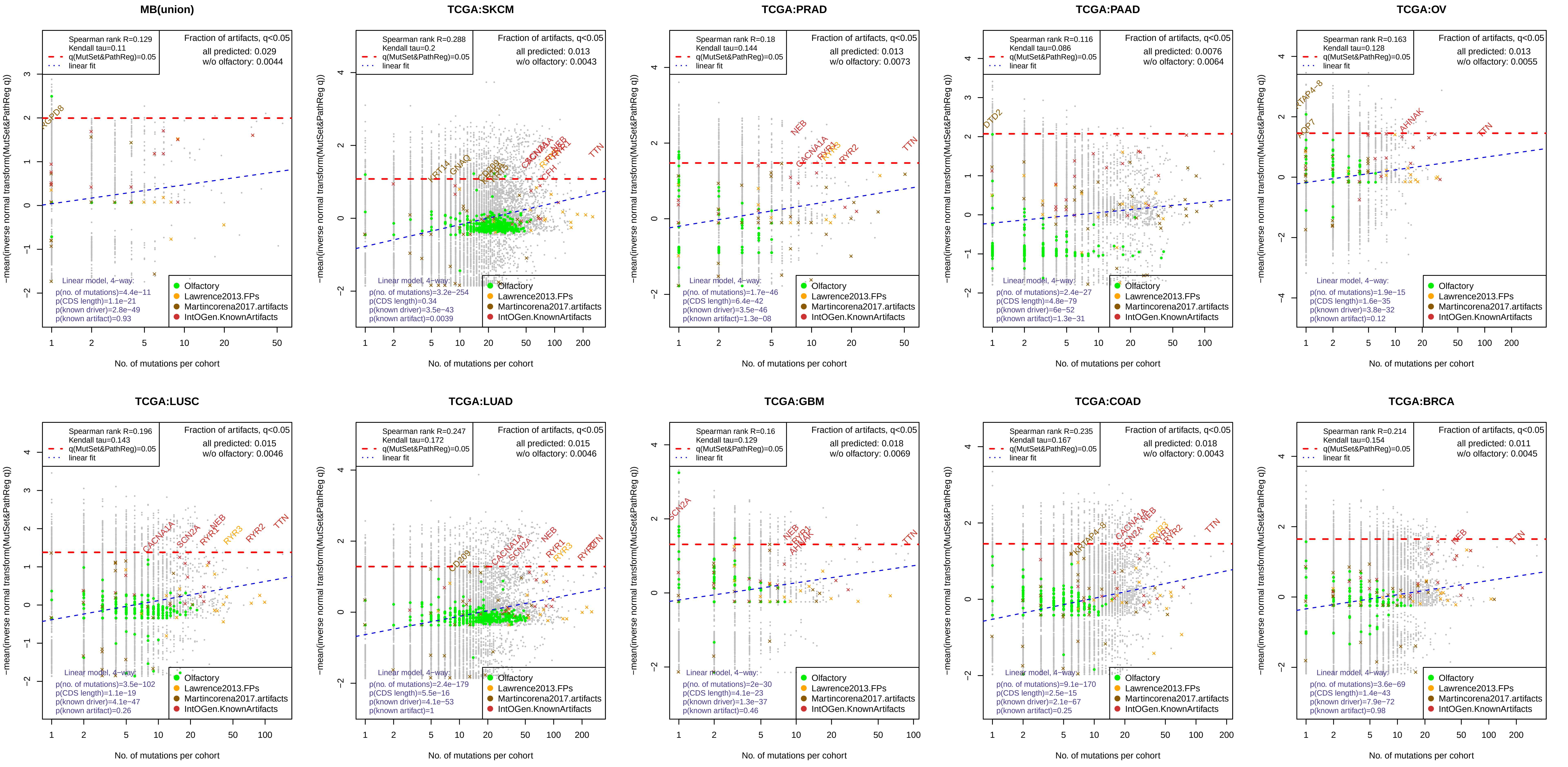
MB(union)
Spearman rank R=0.129
Kendall tau=0.11
q(MutSet&PathReg)=0.05
linear fit
Fraction of artifacts, q<0.05
all predicted: 0.029
w/o olfactory: 0.0044
Linear model, 4−way:
p(no. of mutations)=4.4e−11
p(CDS length)=1.1e−21
p(known driver)=2.8e−49
p(known artifact)=0.93
TCGA:SKCM
Spearman rank R=0.288
Kendall tau=0.2
Fraction of artifacts, q<0.05
all predicted: 0.013
w/o olfactory: 0.0043
Linear model, 4−way:
p(no. of mutations)=3.2e−254
p(CDS length)=0.34
p(known driver)=3.5e−43
p(known artifact)=0.0039
TCGA:PRAD
Spearman rank R=0.18
Kendall tau=0.144
Fraction of artifacts, q<0.05
all predicted: 0.013
w/o olfactory: 0.0073
Linear model, 4−way:
p(no. of mutations)=1.7e−46
p(CDS length)=6.4e−42
p(known driver)=3.5e−46
p(known artifact)=1.3e−08
TCGA:PAAD
Spearman rank R=0.116
Kendall tau=0.086
Fraction of artifacts, q<0.05
all predicted: 0.0076
w/o olfactory: 0.0064
Linear model, 4−way:
p(no. of mutations)=2.4e−27
p(CDS length)=4.8e−79
p(known driver)=6e−52
p(known artifact)=1.3e−31
TCGA:OV
Spearman rank R=0.163
Kendall tau=0.128
Fraction of artifacts, q<0.05
all predicted: 0.013
w/o olfactory: 0.0055
Linear model, 4−way:
p(no. of mutations)=1.9e−15
p(CDS length)=1.6e−35
p(known driver)=3.8e−32
p(known artifact)=0.12
TCGA:LUSC
Spearman rank R=0.196
Kendall tau=0.143
Fraction of artifacts, q<0.05
all predicted: 0.015
w/o olfactory: 0.0046
Linear model, 4−way:
p(no. of mutations)=3.5e−102
p(CDS length)=1.1e−19
p(known driver)=4.1e−47
p(known artifact)=0.26
TCGA:LUAD
Spearman rank R=0.247
Kendall tau=0.172
Fraction of artifacts, q<0.05
all predicted: 0.015
w/o olfactory: 0.0046
Linear model, 4−way:
p(no. of mutations)=2.4e−179
p(CDS length)=5.5e−16
p(known driver)=4.1e−53
p(known artifact)=1
TCGA:GBM
Spearman rank R=0.16
Kendall tau=0.129
Fraction of artifacts, q<0.05
all predicted: 0.018
w/o olfactory: 0.0069
Linear model, 4−way:
p(no. of mutations)=2e−30
p(CDS length)=4.1e−23
p(known driver)=1.3e−37
p(known artifact)=0.46
TCGA:COAD
Spearman rank R=0.235
Kendall tau=0.167
Fraction of artifacts, q<0.05
all predicted: 0.018
w/o olfactory: 0.0043
Linear model, 4−way:
p(no. of mutations)=9.1e−170
p(CDS length)=2.5e−15
p(known driver)=2.1e−67
p(known artifact)=0.25
TCGA:BRCA
Spearman rank R=0.214
Kendall tau=0.154
Fraction of artifacts, q<0.05
all predicted: 0.011
w/o olfactory: 0.0045
Linear model, 4−way:
p(no. of mutations)=3.6e−69
p(CDS length)=1.4e−43
p(known driver)=7.9e−72
p(known artifact)=0.98
Olfactory
Lawrence2013.FPs
Martincorena2017.artifacts
IntOGen.KnownArtifacts
−mean(inverse normal transform(MutSet&PathReg q))
No. of mutations per cohort
TTN
NEB
RYR2
RYR3
RYR1
CACNA1A
SCN2A
AHNAK
KRTAP4−8
CD209
GNAQ
KRT14
DTD2
AQP7
RGPD8

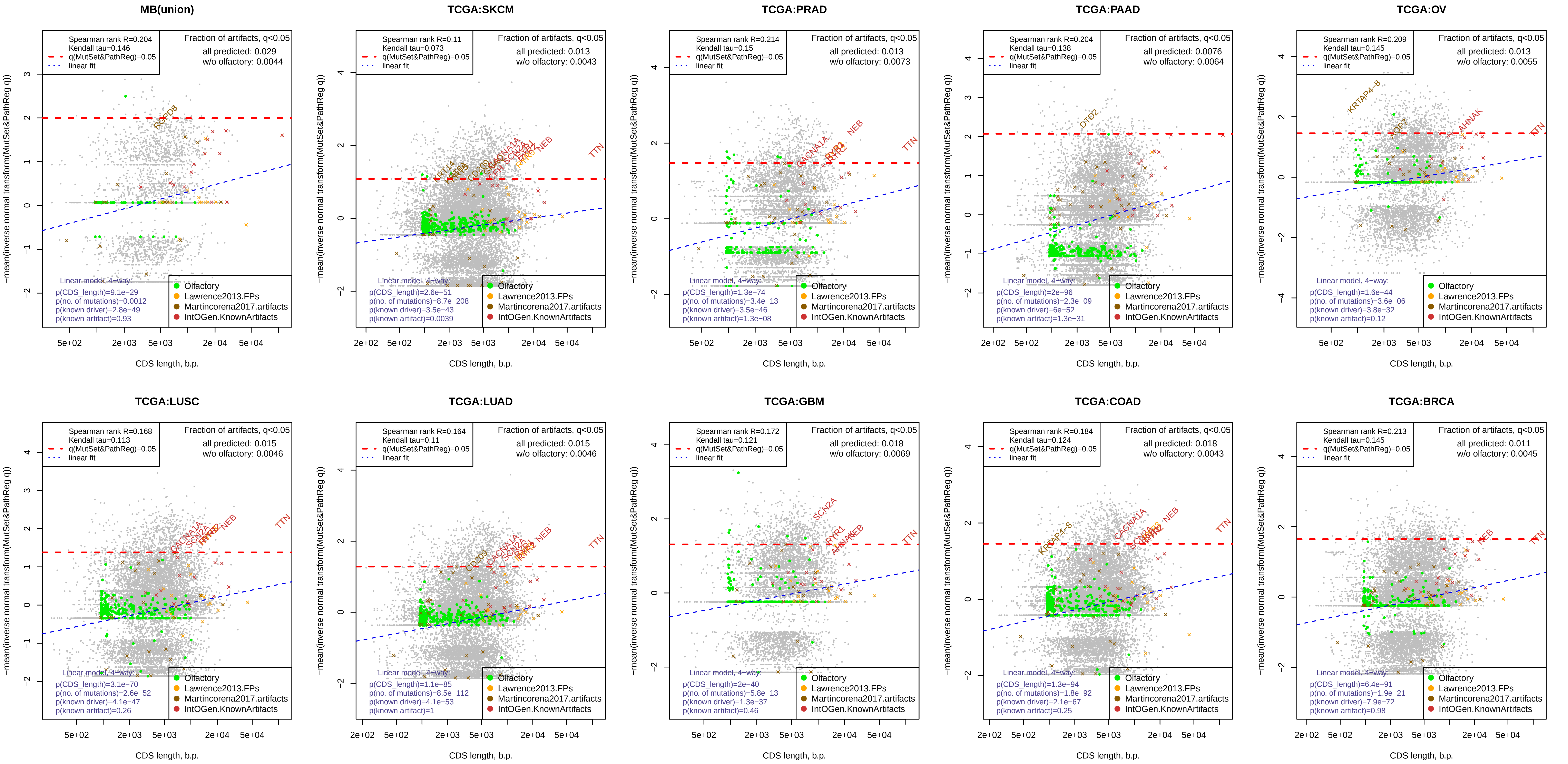
MB(union)
Spearman rank R=0.204
Kendall tau=0.146
q(MutSet&PathReg)=0.05
linear fit
Fraction of artifacts, q<0.05
all predicted: 0.029
w/o olfactory: 0.0044
Linear model, 4-way:
p(CDS_length)=9.1e-29
p(no. of mutations)=0.0012
p(known driver)=2.8e-49
p(known artifact)=0.93
TCGA:SKCM
Spearman rank R=0.11
Kendall tau=0.073
q(MutSet&PathReg)=0.05
linear fit
Fraction of artifacts, q<0.05
all predicted: 0.013
w/o olfactory: 0.0043
Linear model, 4-way:
p(CDS_length)=2.6e-51
p(no. of mutations)=8.7e-208
p(known driver)=3.5e-43
p(known artifact)=0.0039
TCGA:PRAD
Spearman rank R=0.214
Kendall tau=0.15
q(MutSet&PathReg)=0.05
linear fit
Fraction of artifacts, q<0.05
all predicted: 0.013
w/o olfactory: 0.0073
Linear model, 4-way:
p(CDS_length)=1.3e-74
p(no. of mutations)=3.4e-13
p(known driver)=3.5e-46
p(known artifact)=1.3e-08
TCGA:PAAD
Spearman rank R=0.204
Kendall tau=0.138
q(MutSet&PathReg)=0.05
linear fit
Fraction of artifacts, q<0.05
all predicted: 0.0076
w/o olfactory: 0.0064
Linear model, 4-way:
p(CDS_length)=2e-96
p(no. of mutations)=2.3e-09
p(known driver)=6e-52
p(known artifact)=1.3e-31
TCGA:OV
Spearman rank R=0.209
Kendall tau=0.145
q(MutSet&PathReg)=0.05
linear fit
Fraction of artifacts, q<0.05
all predicted: 0.013
w/o olfactory: 0.0055
Linear model, 4-way:
p(CDS_length)=1.6e-44
p(no. of mutations)=3.6e-06
p(known driver)=3.8e-32
p(known artifact)=0.12
TCGA:LUSC
Spearman rank R=0.168
Kendall tau=0.113
q(MutSet&PathReg)=0.05
linear fit
Fraction of artifacts, q<0.05
all predicted: 0.015
w/o olfactory: 0.0046
Linear model, 4-way:
p(CDS_length)=3.1e-70
p(no. of mutations)=2.6e-52
p(known driver)=4.1e-47
p(known artifact)=0.26
TCGA:LUAD
Spearman rank R=0.164
Kendall tau=0.11
q(MutSet&PathReg)=0.05
linear fit
Fraction of artifacts, q<0.05
all predicted: 0.015
w/o olfactory: 0.0046
Linear model, 4-way:
p(CDS_length)=1.1e-85
p(no. of mutations)=8.5e-112
p(known driver)=4.1e-53
p(known artifact)=1
TCGA:GBM
Spearman rank R=0.172
Kendall tau=0.121
q(MutSet&PathReg)=0.05
linear fit
Fraction of artifacts, q<0.05
all predicted: 0.018
w/o olfactory: 0.0069
Linear model, 4-way:
p(CDS_length)=2e-40
p(no. of mutations)=5.8e-13
p(known driver)=1.3e-37
p(known artifact)=0.46
TCGA:COAD
Spearman rank R=0.184
Kendall tau=0.124
q(MutSet&PathReg)=0.05
linear fit
Fraction of artifacts, q<0.05
all predicted: 0.018
w/o olfactory: 0.0043
Linear model, 4-way:
p(CDS_length)=1.3e-94
p(no. of mutations)=1.8e-92
p(known driver)=2.1e-67
p(known artifact)=0.25
TCGA:BRCA
Spearman rank R=0.213
Kendall tau=0.145
q(MutSet&PathReg)=0.05
linear fit
Fraction of artifacts, q<0.05
all predicted: 0.011
w/o olfactory: 0.0045
Linear model, 4-way:
p(CDS_length)=6.4e-91
p(no. of mutations)=1.9e-21
p(known driver)=7.9e-72
p(known artifact)=0.98
Olfactory
Lawrence2013.FPs
Martincorena2017.artifacts
IntOGen.KnownArtifacts
−mean(inverse normal transform(MutSet&PathReg q))
CDS length, b.p.
TTN
NEB
RYR1
RYR2
CACNA1A
SCN2A
CD209
KRTAP4-8
DTD2
AHNAK
SCN2A

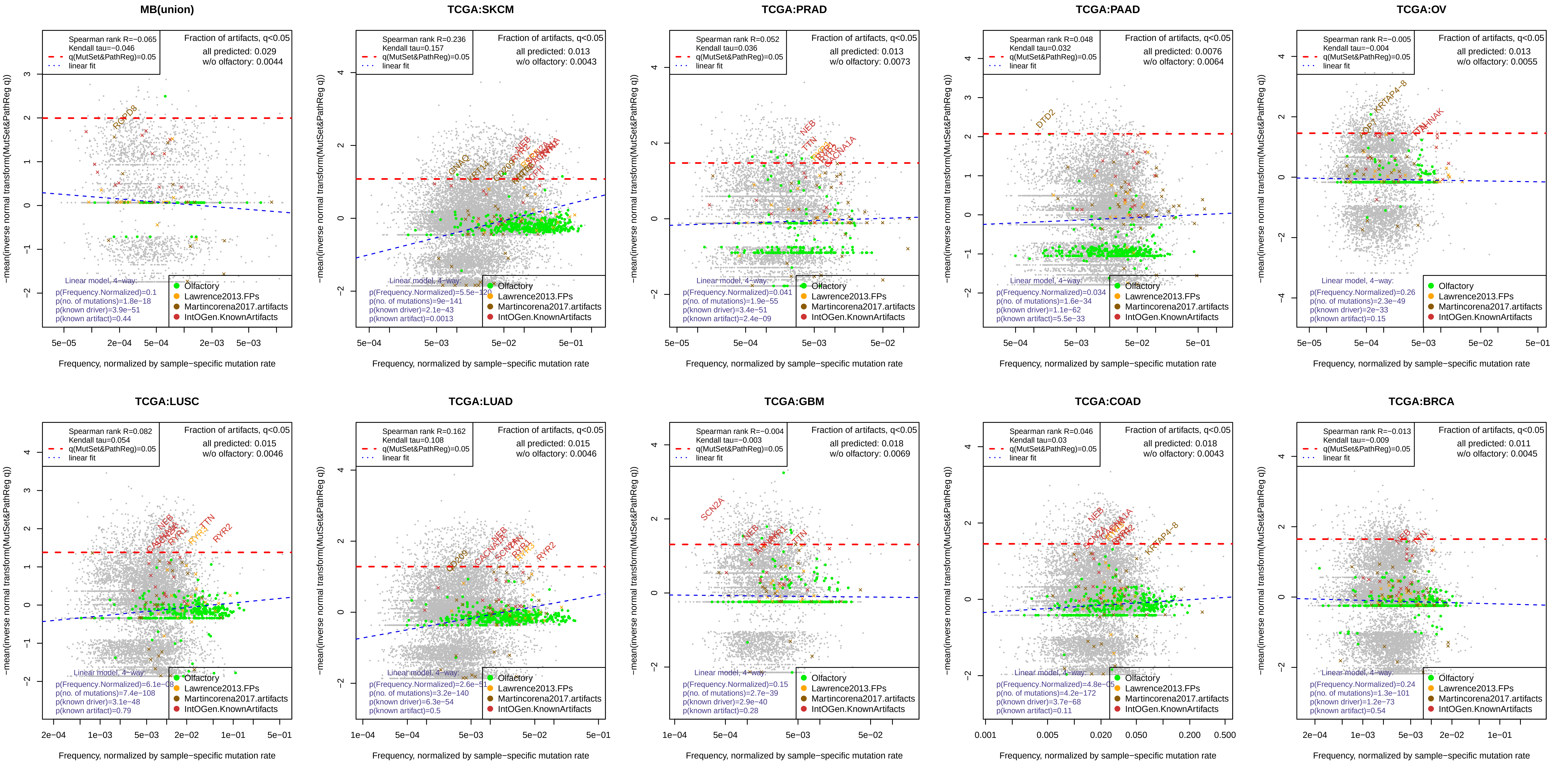

MB(union)
Spearman rank R=−0.065
Kendall tau=−0.046
q(MutSet&PathReg)=0.05
linear fit
Fraction of artifacts, q<0.05
all predicted: 0.029
w/o olfactory: 0.0044
Linear model, 4−way:
p(Frequency.Normalized)=0.1
p(no. of mutations)=1.8e−18
p(known driver)=3.9e−51
p(known artifact)=0.44
TCGA:SKCM
Spearman rank R=0.236
Kendall tau=0.157
all predicted: 0.013
w/o olfactory: 0.0043
p(Frequency.Normalized)=5.5e−120
p(no. of mutations)=9e−141
p(known driver)=2.1e−43
p(known artifact)=0.0013
TCGA:PRAD
Spearman rank R=0.052
Kendall tau=0.036
all predicted: 0.013
w/o olfactory: 0.0073
p(Frequency.Normalized)=0.041
p(no. of mutations)=1.9e−55
p(known driver)=3.4e−51
p(known artifact)=2.4e−09
TCGA:PAAD
Spearman rank R=0.048
Kendall tau=0.032
all predicted: 0.0076
w/o olfactory: 0.0064
p(Frequency.Normalized)=0.034
p(no. of mutations)=1.6e−34
p(known driver)=1.1e−62
p(known artifact)=5.5e−33
TCGA:OV
Spearman rank R=−0.005
Kendall tau=−0.004
all predicted: 0.013
w/o olfactory: 0.0055
p(Frequency.Normalized)=0.26
p(no. of mutations)=2.3e−49
p(known driver)=2e−33
p(known artifact)=0.15
TCGA:LUSC
Spearman rank R=0.082
Kendall tau=0.054
all predicted: 0.015
w/o olfactory: 0.0046
p(Frequency.Normalized)=6.1e−08
p(no. of mutations)=7.4e−108
p(known driver)=3.1e−48
p(known artifact)=0.79
TCGA:LUAD
Spearman rank R=0.162
Kendall tau=0.108
all predicted: 0.015
w/o olfactory: 0.0046
p(Frequency.Normalized)=2.6e−51
p(no. of mutations)=3.2e−140
p(known driver)=6.3e−54
p(known artifact)=0.5
TCGA:GBM
Spearman rank R=−0.004
Kendall tau=−0.003
all predicted: 0.018
w/o olfactory: 0.0069
p(Frequency.Normalized)=0.15
p(no. of mutations)=2.7e−39
p(known driver)=2.9e−40
p(known artifact)=0.28
TCGA:COAD
Spearman rank R=0.046
Kendall tau=0.03
all predicted: 0.018
w/o olfactory: 0.0043
p(Frequency.Normalized)=4.8e−05
p(no. of mutations)=4.2e−172
p(known driver)=3.7e−68
p(known artifact)=0.11
TCGA:BRCA
Spearman rank R=−0.013
Kendall tau=−0.009
all predicted: 0.011
w/o olfactory: 0.0045
p(Frequency.Normalized)=0.24
p(no. of mutations)=1.3e−101
p(known driver)=1.2e−73
p(known artifact)=0.54
Olfactory
Lawrence2013.FPs
Martincorena2017.artifacts
IntOGen.KnownArtifacts
Frequency, normalized by sample−specific mutation rate
−mean(inverse normal transform(MutSet&PathReg q))

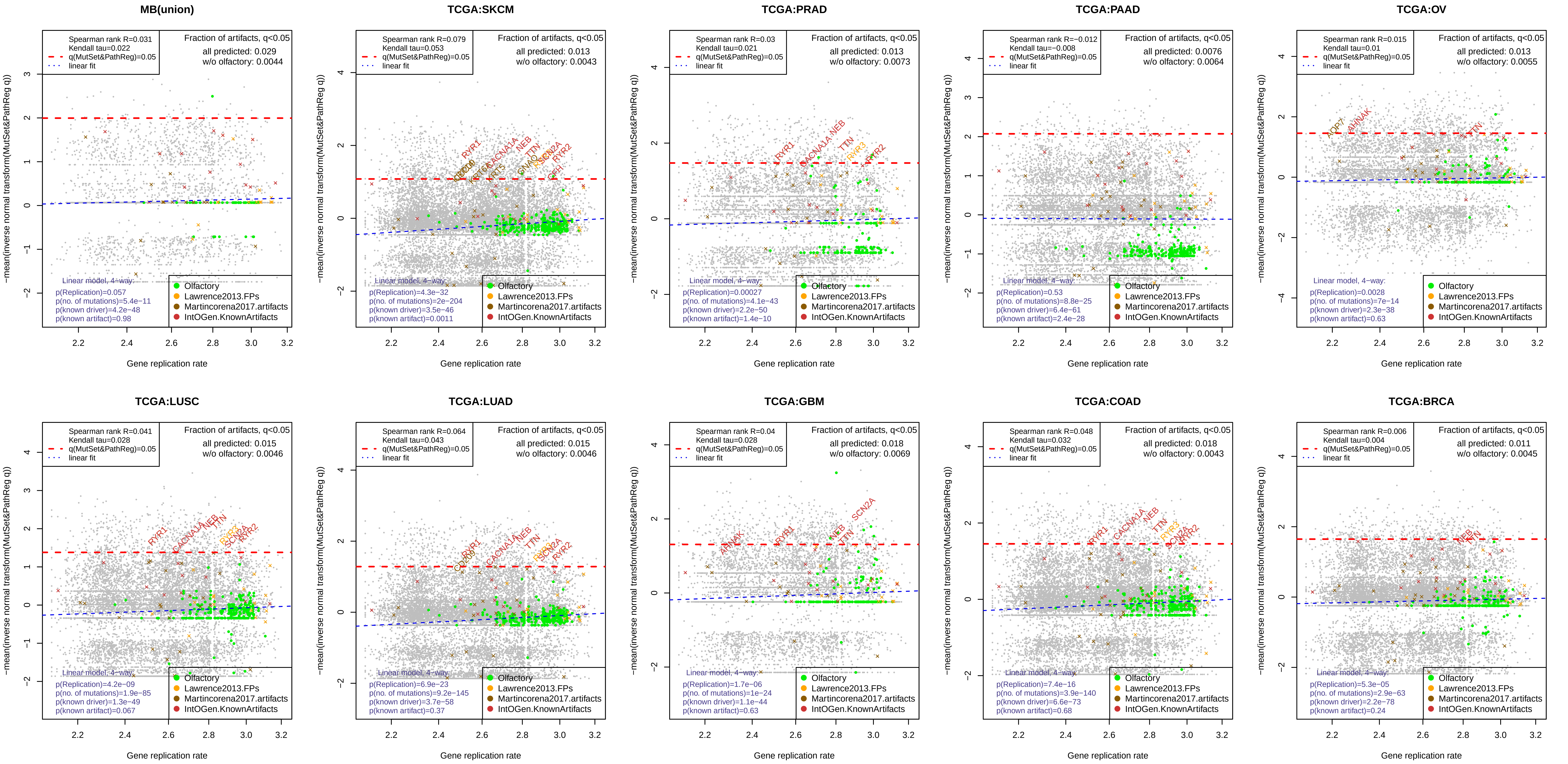

MB(union)
Spearman rank R=0.031
Kendall tau=0.022
q(MutSet&PathReg)=0.05
linear fit
Fraction of artifacts, q<0.05
all predicted: 0.029
w/o olfactory: 0.0044
Linear model, 4-way:
p(Replication)=0.057
p(no. of mutations)=5.4e−11
p(known driver)=4.2e−48
p(known artifact)=0.98
Olfactory
Lawrence2013.FPs
Martincorena2017.artifacts
IntOGen.KnownArtifacts
TCGA:SKCM
Spearman rank R=0.079
Kendall tau=0.053
q(MutSet&PathReg)=0.05
linear fit
Fraction of artifacts, q<0.05
all predicted: 0.013
w/o olfactory: 0.0043
Linear model, 4-way:
p(Replication)=4.3e−32
p(no. of mutations)=2e−204
p(known driver)=3.5e−46
p(known artifact)=0.0011
Olfactory
Lawrence2013.FPs
Martincorena2017.artifacts
IntOGen.KnownArtifacts
TCGA:PRAD
Spearman rank R=0.03
Kendall tau=0.021
q(MutSet&PathReg)=0.05
linear fit
Fraction of artifacts, q<0.05
all predicted: 0.013
w/o olfactory: 0.0073
Linear model, 4-way:
p(Replication)=0.00027
p(no. of mutations)=4.1e−43
p(known driver)=2.2e−50
p(known artifact)=1.4e−10
Olfactory
Lawrence2013.FPs
Martincorena2017.artifacts
IntOGen.KnownArtifacts
TCGA:PAAD
Spearman rank R=−0.012
Kendall tau=−0.008
q(MutSet&PathReg)=0.05
linear fit
Fraction of artifacts, q<0.05
all predicted: 0.0076
w/o olfactory: 0.0064
Linear model, 4-way:
p(Replication)=0.53
p(no. of mutations)=8.8e−25
p(known driver)=6.4e−61
p(known artifact)=2.4e−28
Olfactory
Lawrence2013.FPs
Martincorena2017.artifacts
IntOGen.KnownArtifacts
TCGA:OV
Spearman rank R=0.015
Kendall tau=0.01
q(MutSet&PathReg)=0.05
linear fit
Fraction of artifacts, q<0.05
all predicted: 0.013
w/o olfactory: 0.0055
Linear model, 4-way:
p(Replication)=0.0028
p(no. of mutations)=7e−14
p(known driver)=2.3e−38
p(known artifact)=0.63
Olfactory
Lawrence2013.FPs
Martincorena2017.artifacts
IntOGen.KnownArtifacts
TCGA:LUSC
Spearman rank R=0.041
Kendall tau=0.028
q(MutSet&PathReg)=0.05
linear fit
Fraction of artifacts, q<0.05
all predicted: 0.015
w/o olfactory: 0.0046
Linear model, 4-way:
p(Replication)=4.2e−09
p(no. of mutations)=1.9e−85
p(known driver)=1.3e−49
p(known artifact)=0.067
Olfactory
Lawrence2013.FPs
Martincorena2017.artifacts
IntOGen.KnownArtifacts
TCGA:LUAD
Spearman rank R=0.064
Kendall tau=0.043
q(MutSet&PathReg)=0.05
linear fit
Fraction of artifacts, q<0.05
all predicted: 0.015
w/o olfactory: 0.0046
Linear model, 4-way:
p(Replication)=6.9e−23
p(no. of mutations)=9.2e−145
p(known driver)=3.7e−58
p(known artifact)=0.37
Olfactory
Lawrence2013.FPs
Martincorena2017.artifacts
IntOGen.KnownArtifacts
TCGA:GBM
Spearman rank R=0.04
Kendall tau=0.028
q(MutSet&PathReg)=0.05
linear fit
Fraction of artifacts, q<0.05
all predicted: 0.018
w/o olfactory: 0.0069
Linear model, 4-way:
p(Replication)=1.7e−06
p(no. of mutations)=1e−24
p(known driver)=1.1e−44
p(known artifact)=0.63
Olfactory
Lawrence2013.FPs
Martincorena2017.artifacts
IntOGen.KnownArtifacts
TCGA:COAD
Spearman rank R=0.048
Kendall tau=0.032
q(MutSet&PathReg)=0.05
linear fit
Fraction of artifacts, q<0.05
all predicted: 0.018
w/o olfactory: 0.0043
Linear model, 4-way:
p(Replication)=7.4e−16
p(no. of mutations)=3.9e−140
p(known driver)=6.6e−73
p(known artifact)=0.68
Olfactory
Lawrence2013.FPs
Martincorena2017.artifacts
IntOGen.KnownArtifacts
TCGA:BRCA
Spearman rank R=0.006
Kendall tau=0.004
q(MutSet&PathReg)=0.05
linear fit
Fraction of artifacts, q<0.05
all predicted: 0.011
w/o olfactory: 0.0045
Linear model, 4-way:
p(Replication)=5.3e−05
p(no. of mutations)=2.9e−63
p(known driver)=2.2e−78
p(known artifact)=0.24
Olfactory
Lawrence2013.FPs
Martincorena2017.artifacts
IntOGen.KnownArtifacts
−mean(inverse normal transform(MutSet&PathReg q))
Gene replication rate

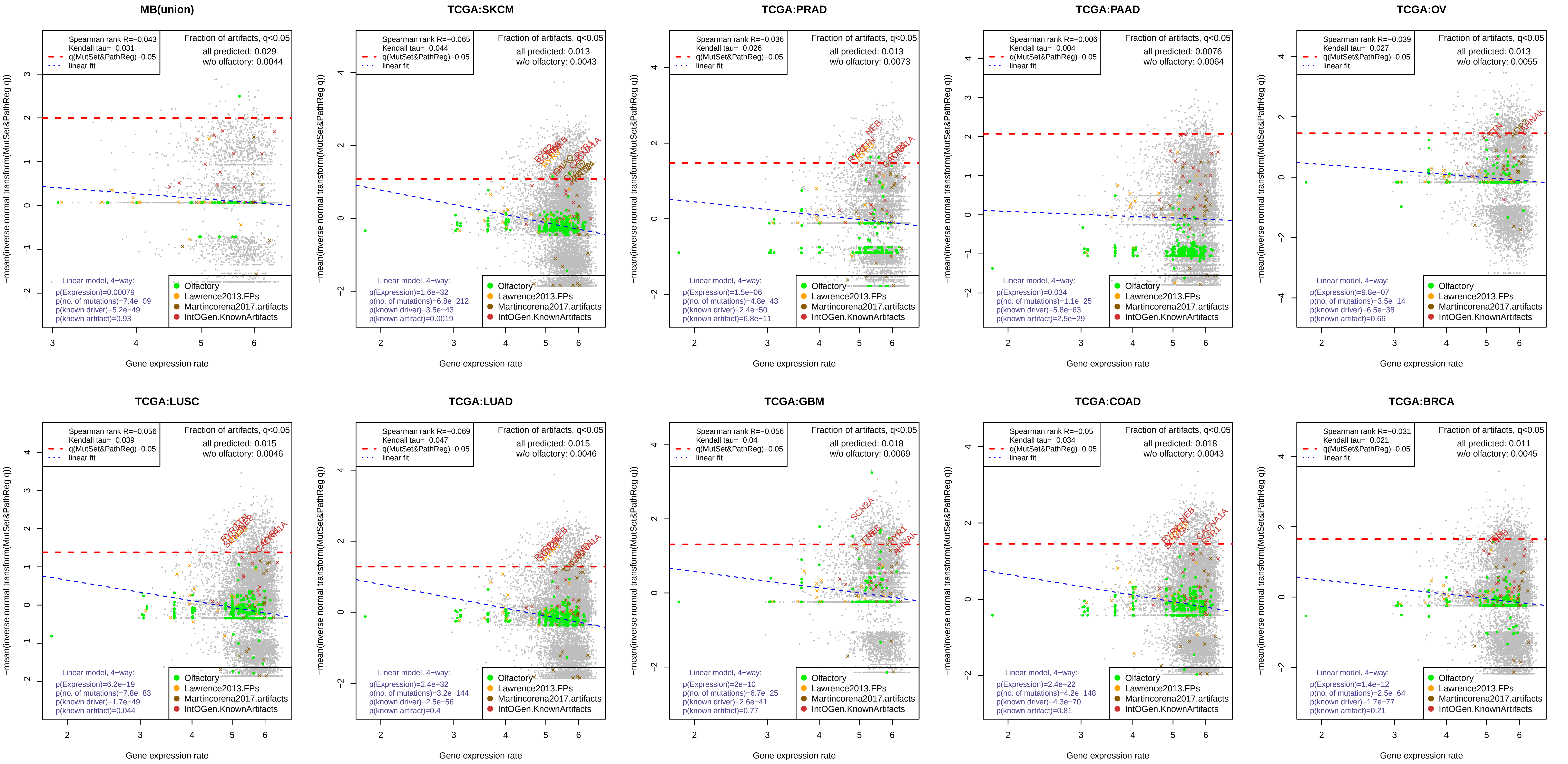
MB(union)
Spearman rank R=−0.043
Kendall tau=−0.031
q(MutSet&PathReg)=0.05
linear fit
Fraction of artifacts, q<0.05
all predicted: 0.029
w/o olfactory: 0.0044
Linear model, 4−way:
p(Expression)=0.00079
p(no. of mutations)=7.4e−09
p(known driver)=5.2e−49
p(known artifact)=0.93
TCGA:SKCM
Spearman rank R=−0.065
Kendall tau=−0.044
q(MutSet&PathReg)=0.05
linear fit
Fraction of artifacts, q<0.05
all predicted: 0.013
w/o olfactory: 0.0043
Linear model, 4−way:
p(Expression)=1.6e−32
p(no. of mutations)=6.8e−212
p(known driver)=3.5e−43
p(known artifact)=0.0019
TCGA:PRAD
Spearman rank R=−0.036
Kendall tau=−0.026
q(MutSet&PathReg)=0.05
linear fit
Fraction of artifacts, q<0.05
all predicted: 0.013
w/o olfactory: 0.0073
Linear model, 4−way:
p(Expression)=1.5e−06
p(no. of mutations)=4.8e−43
p(known driver)=2.4e−50
p(known artifact)=6.8e−11
TCGA:PAAD
Spearman rank R=−0.006
Kendall tau=−0.004
q(MutSet&PathReg)=0.05
linear fit
Fraction of artifacts, q<0.05
all predicted: 0.0076
w/o olfactory: 0.0064
Linear model, 4−way:
p(Expression)=0.034
p(no. of mutations)=1.1e−25
p(known driver)=5.8e−63
p(known artifact)=2.5e−29
TCGA:OV
Spearman rank R=−0.039
Kendall tau=−0.027
q(MutSet&PathReg)=0.05
linear fit
Fraction of artifacts, q<0.05
all predicted: 0.013
w/o olfactory: 0.0055
Linear model, 4−way:
p(Expression)=9.8e−07
p(no. of mutations)=3.5e−14
p(known driver)=6.5e−38
p(known artifact)=0.66
TCGA:LUSC
Spearman rank R=−0.056
Kendall tau=−0.039
q(MutSet&PathReg)=0.05
linear fit
Fraction of artifacts, q<0.05
all predicted: 0.015
w/o olfactory: 0.0046
Linear model, 4−way:
p(Expression)=6.2e−19
p(no. of mutations)=7.8e−83
p(known driver)=1.7e−49
p(known artifact)=0.044
TCGA:LUAD
Spearman rank R=−0.069
Kendall tau=−0.047
q(MutSet&PathReg)=0.05
linear fit
Fraction of artifacts, q<0.05
all predicted: 0.015
w/o olfactory: 0.0046
Linear model, 4−way:
p(Expression)=2.4e−32
p(no. of mutations)=3.2e−144
p(known driver)=2.5e−56
p(known artifact)=0.4
TCGA:GBM
Spearman rank R=−0.056
Kendall tau=−0.04
q(MutSet&PathReg)=0.05
linear fit
Fraction of artifacts, q<0.05
all predicted: 0.018
w/o olfactory: 0.0069
Linear model, 4−way:
p(Expression)=2e−10
p(no. of mutations)=6.7e−25
p(known driver)=2.6e−41
p(known artifact)=0.77
TCGA:COAD
Spearman rank R=−0.05
Kendall tau=−0.034
q(MutSet&PathReg)=0.05
linear fit
Fraction of artifacts, q<0.05
all predicted: 0.018
w/o olfactory: 0.0043
Linear model, 4−way:
p(Expression)=2.4e−22
p(no. of mutations)=4.2e−148
p(known driver)=4.3e−70
p(known artifact)=0.81
TCGA:BRCA
Spearman rank R=−0.031
Kendall tau=−0.021
q(MutSet&PathReg)=0.05
linear fit
Fraction of artifacts, q<0.05
all predicted: 0.011
w/o olfactory: 0.0045
Linear model, 4−way:
p(Expression)=1.4e−12
p(no. of mutations)=2.5e−64
p(known driver)=1.7e−77
p(known artifact)=0.21
Olfactory
Lawrence2013.FPs
Martincorena2017.artifacts
IntOGen.KnownArtifacts
Gene expression rate
−mean(inverse normal transform(MutSet&PathReg q))
SCN2A
TTN
NEB
RYR1
AHNAK
CACNA1A
