# Supplementary figures and images for: Individualized discovery of rare cancer drivers in global network context (part 2 of 2)
Source: eLife. 2022 May 20;11:e74010. doi: 10.7554/eLife.74010 (PMC9159755; doi:10.7554/eLife.74010)

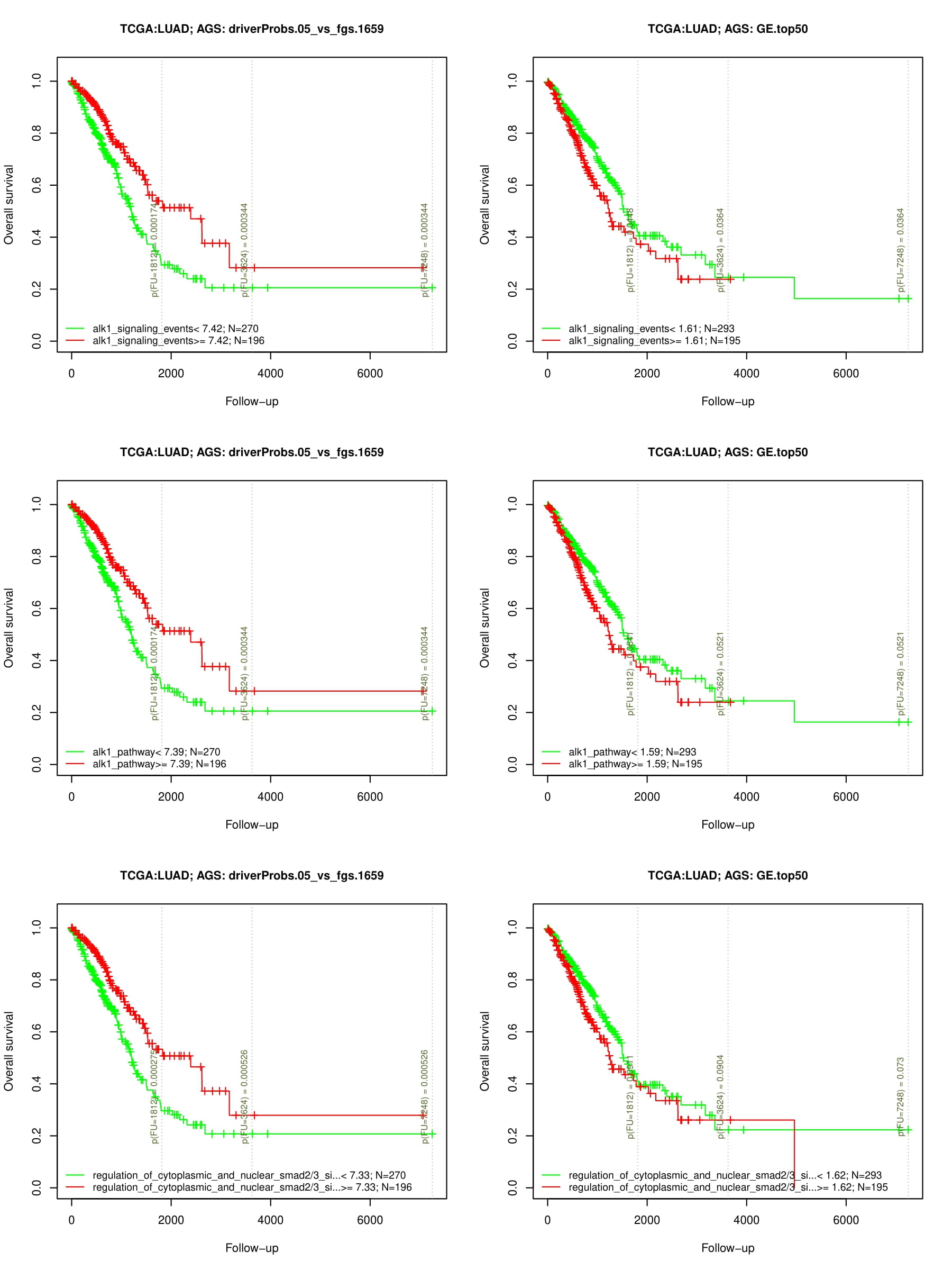

Supplement: Supplementary file 6. [file elife-74010-supp6.zip › SupplementaryFile6-73.jpg]

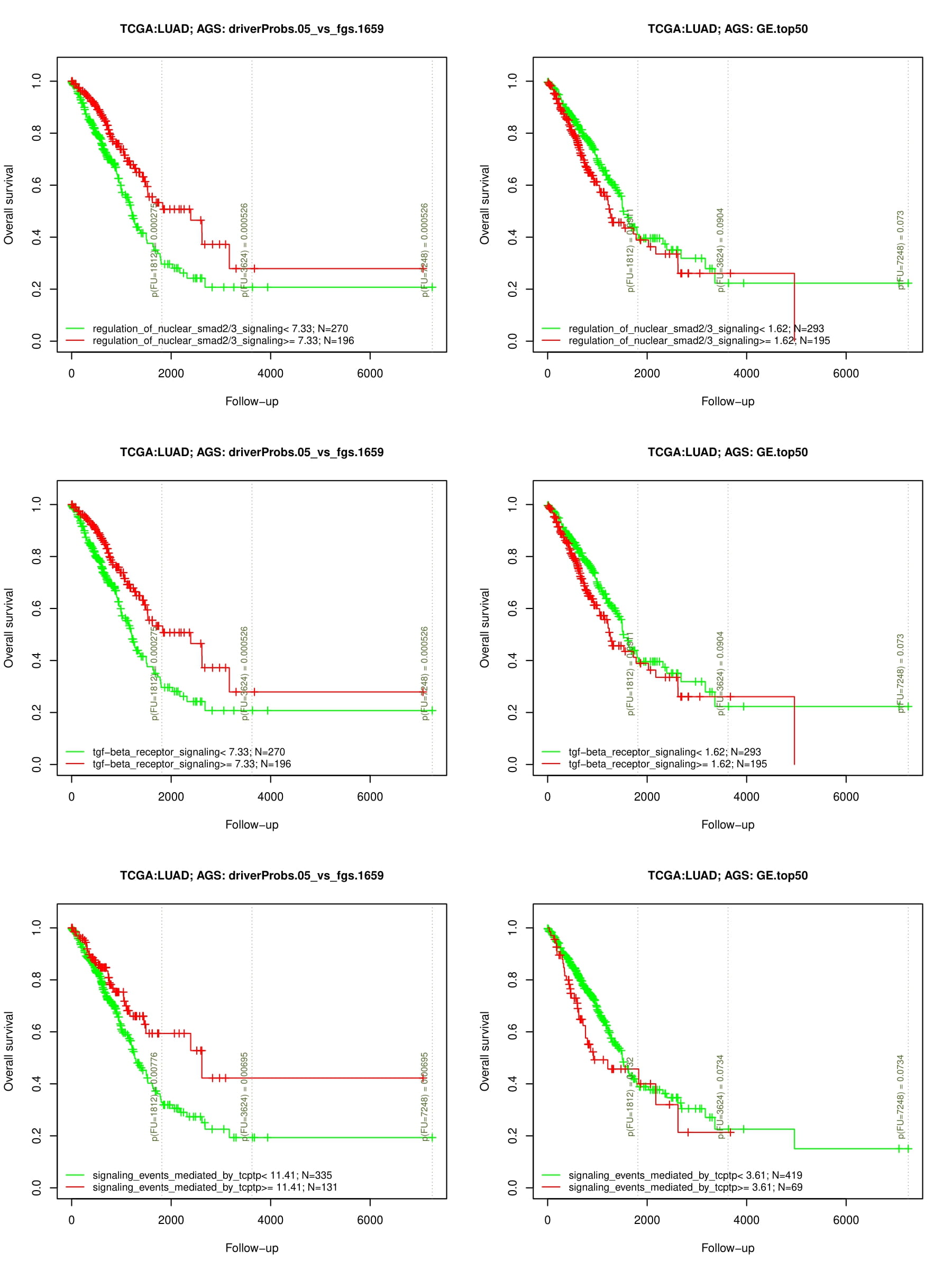

Supplement: Supplementary file 6. [file elife-74010-supp6.zip › SupplementaryFile6-74.jpg]

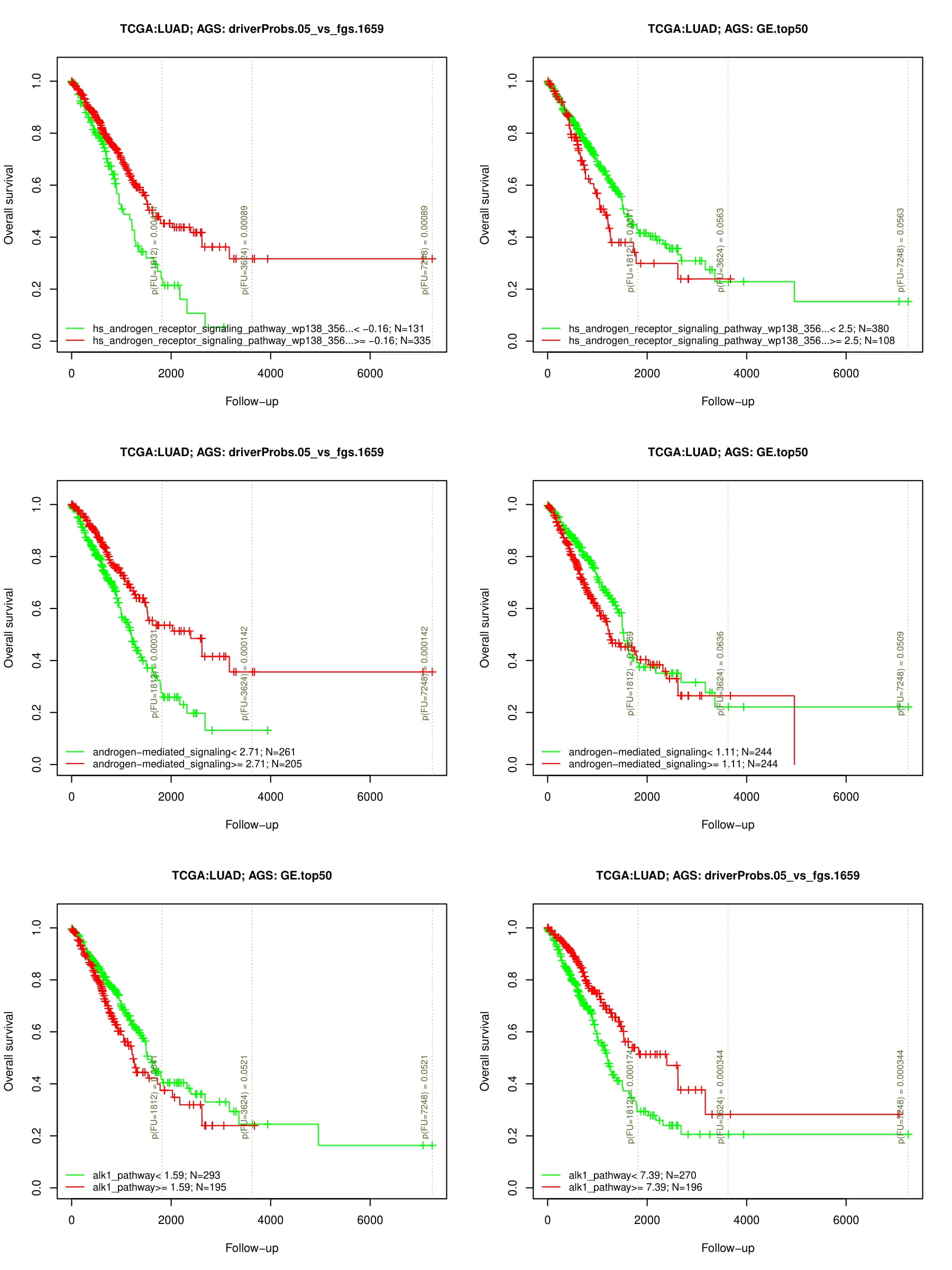

Supplement: Supplementary file 6. [file elife-74010-supp6.zip › SupplementaryFile6-75.jpg]

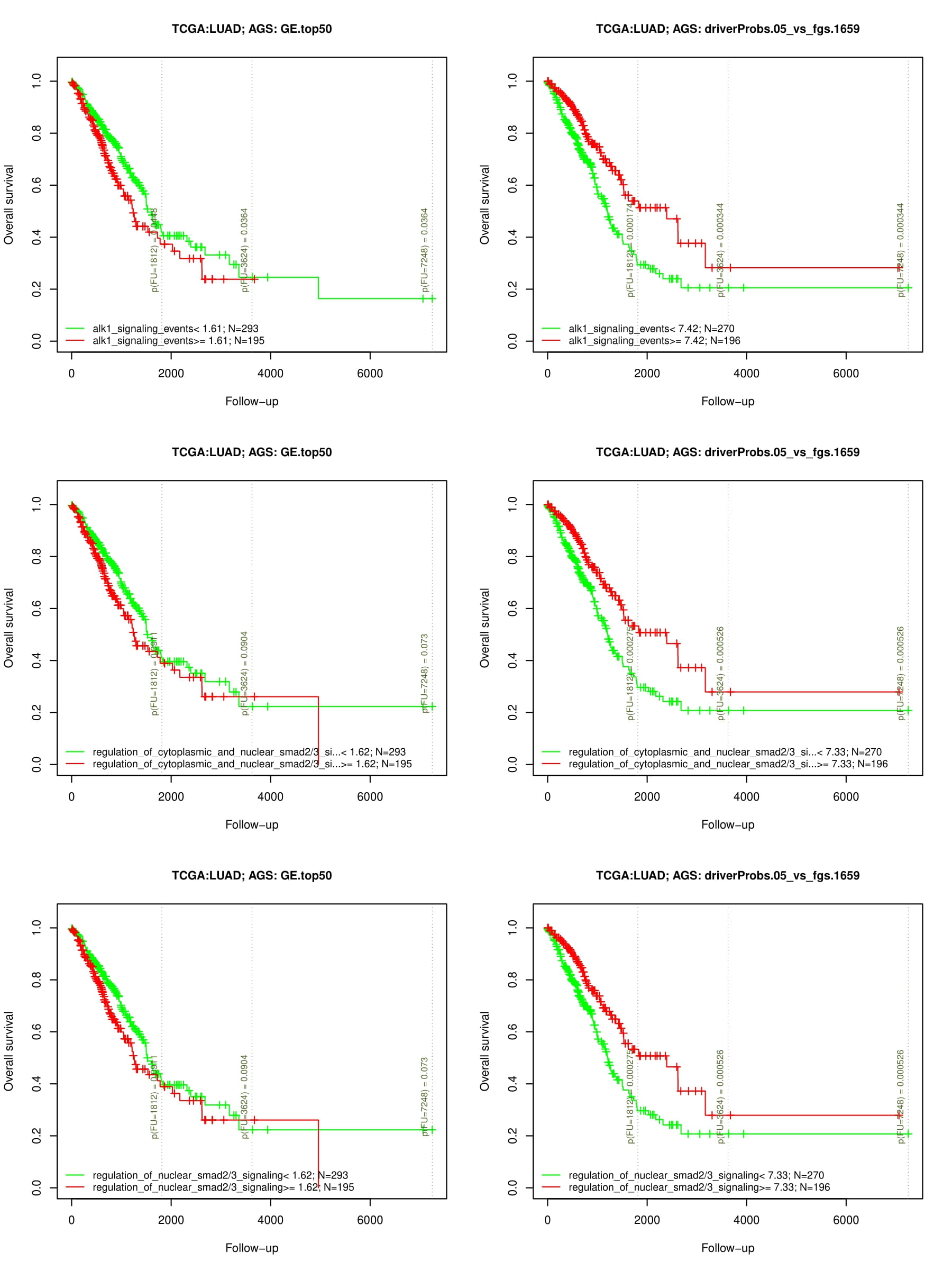

Supplement: Supplementary file 6. [file elife-74010-supp6.zip › SupplementaryFile6-76.jpg]

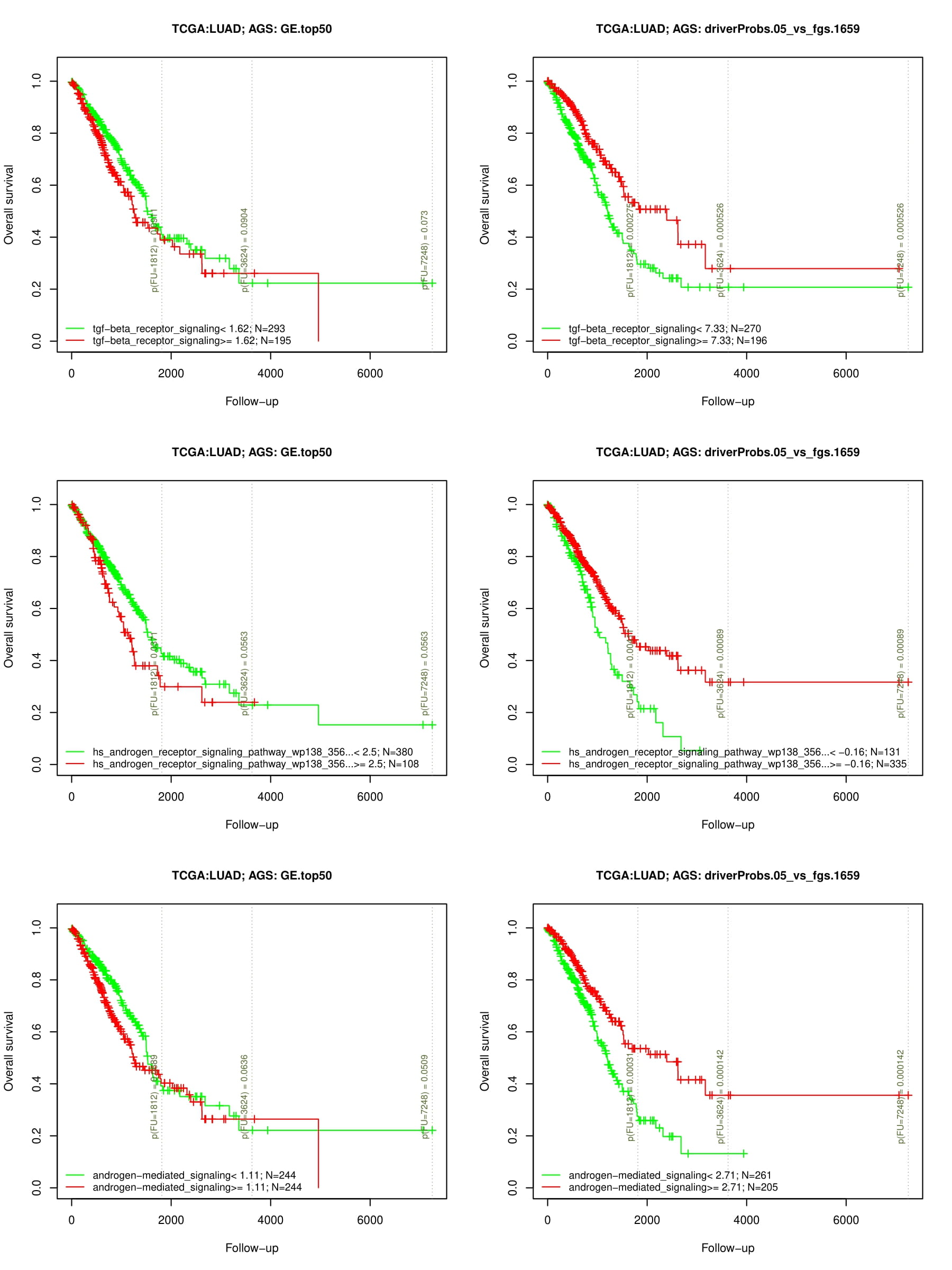

Supplement: Supplementary file 6. [file elife-74010-supp6.zip › SupplementaryFile6-77.jpg]

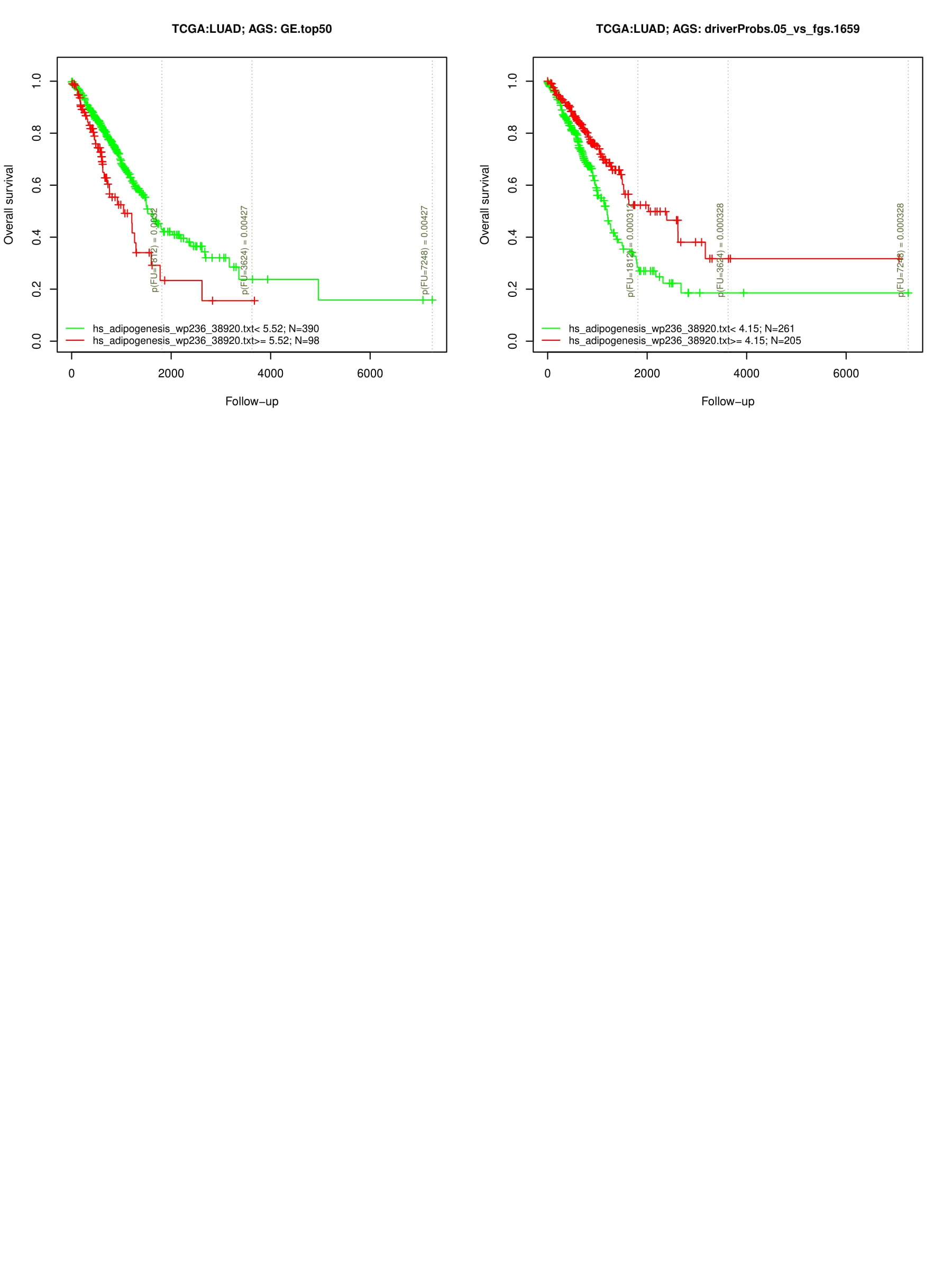

Supplement: Supplementary file 6. [file elife-74010-supp6.zip › SupplementaryFile6-78.jpg]

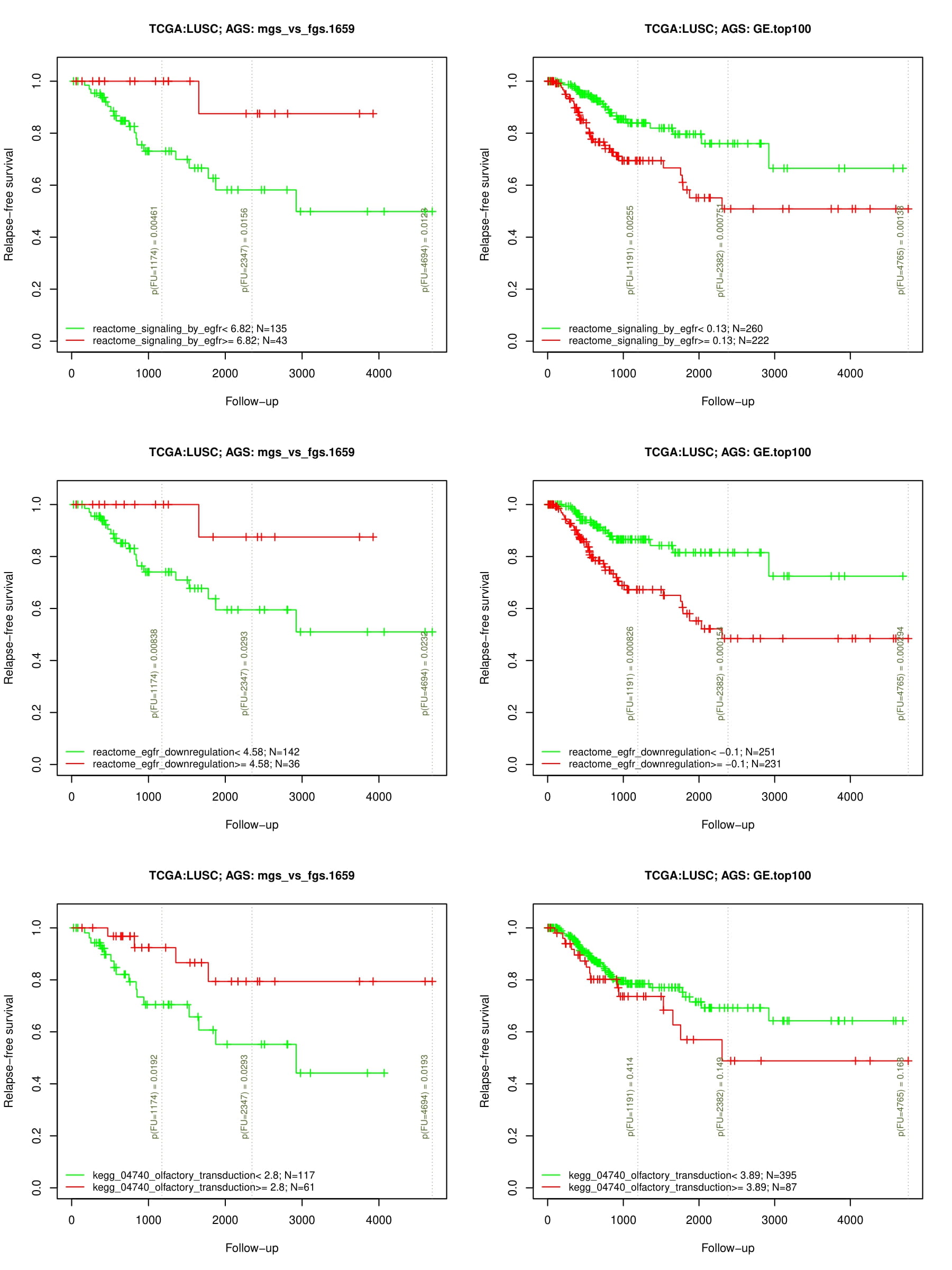

Supplement: Supplementary file 6. [file elife-74010-supp6.zip › SupplementaryFile6-79.jpg]

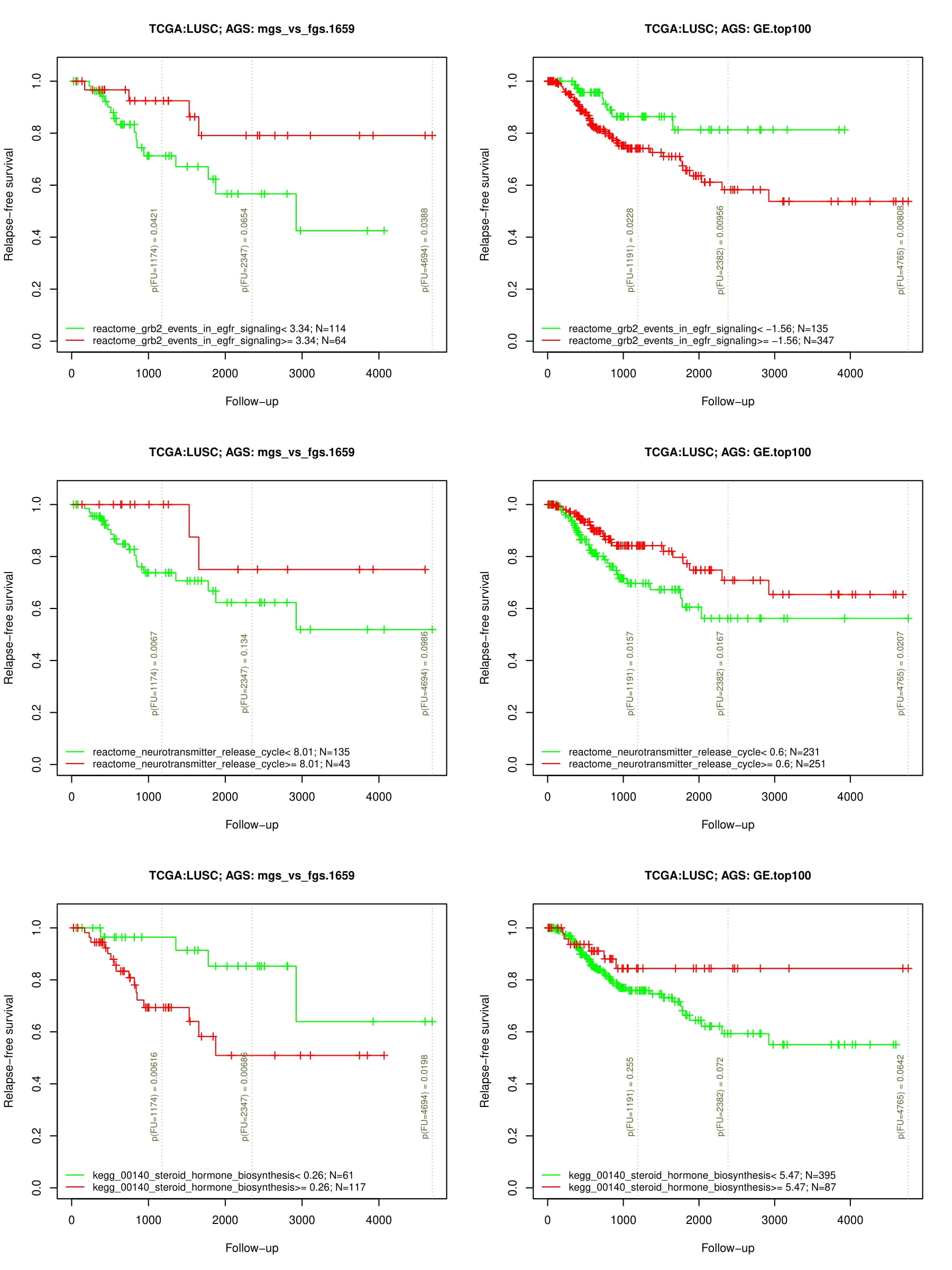

Supplement: Supplementary file 6. [file elife-74010-supp6.zip › SupplementaryFile6-80.jpg]

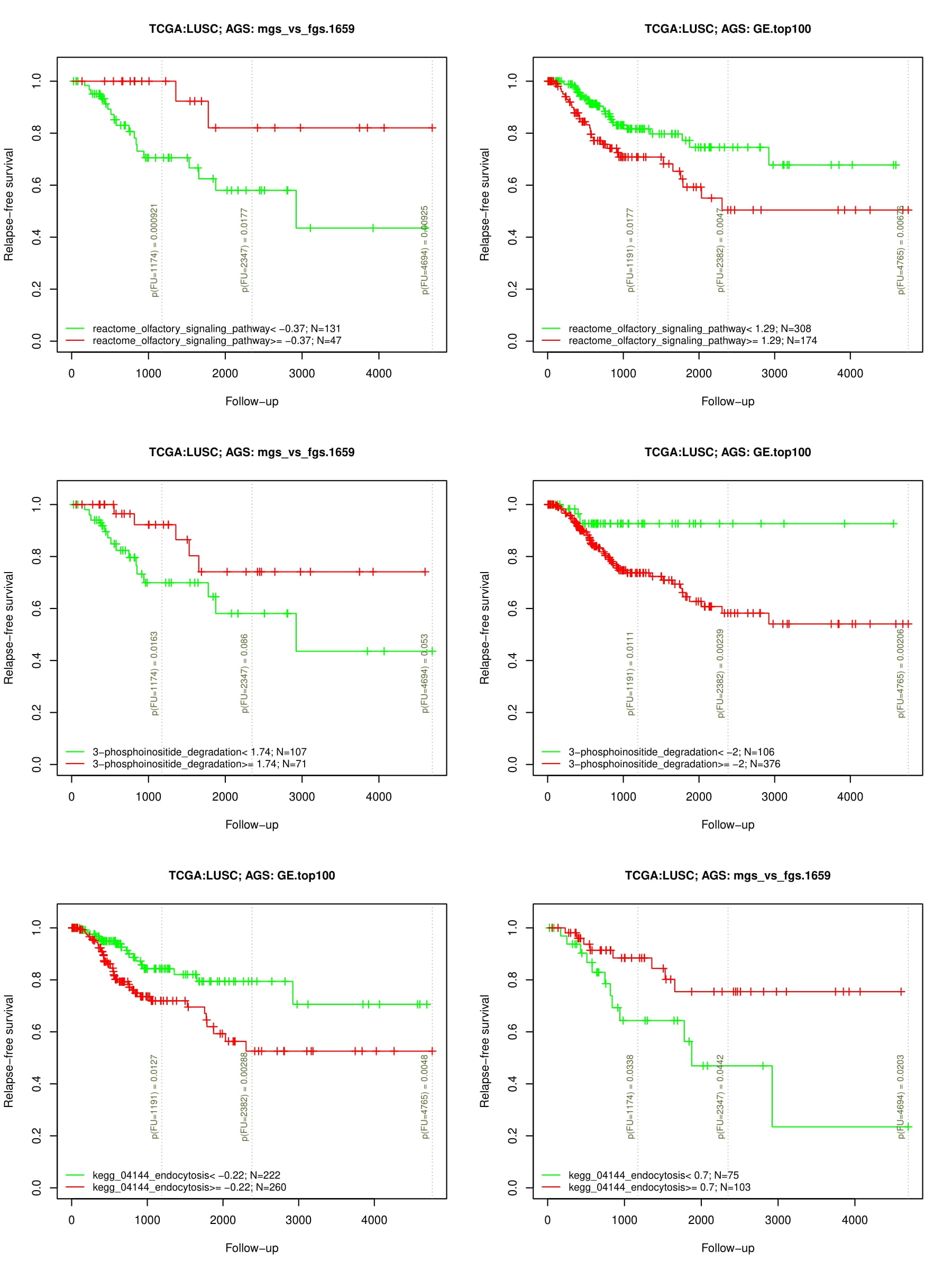

Supplement: Supplementary file 6. [file elife-74010-supp6.zip › SupplementaryFile6-81.jpg]

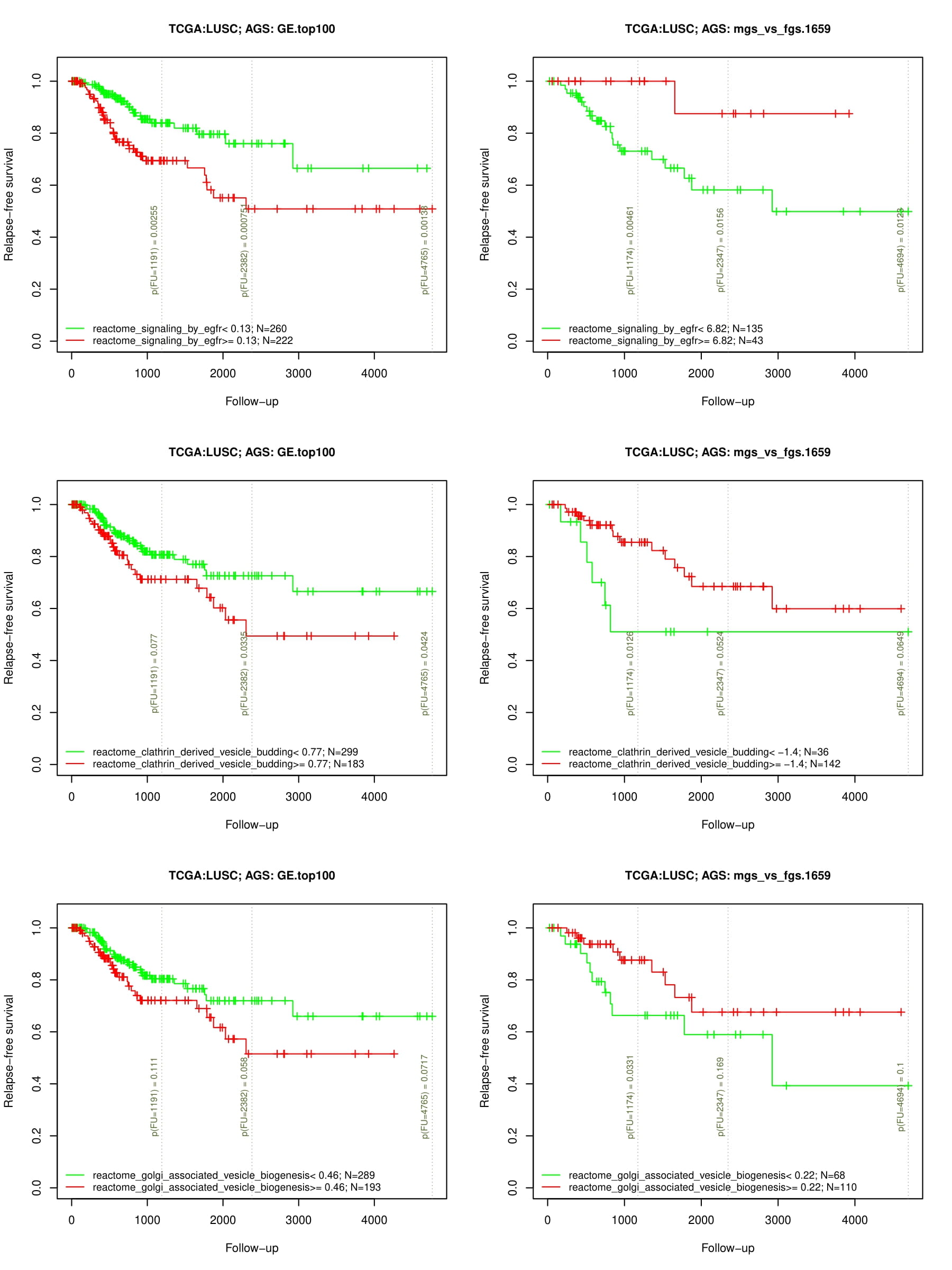

Supplement: Supplementary file 6. [file elife-74010-supp6.zip › SupplementaryFile6-82.jpg]

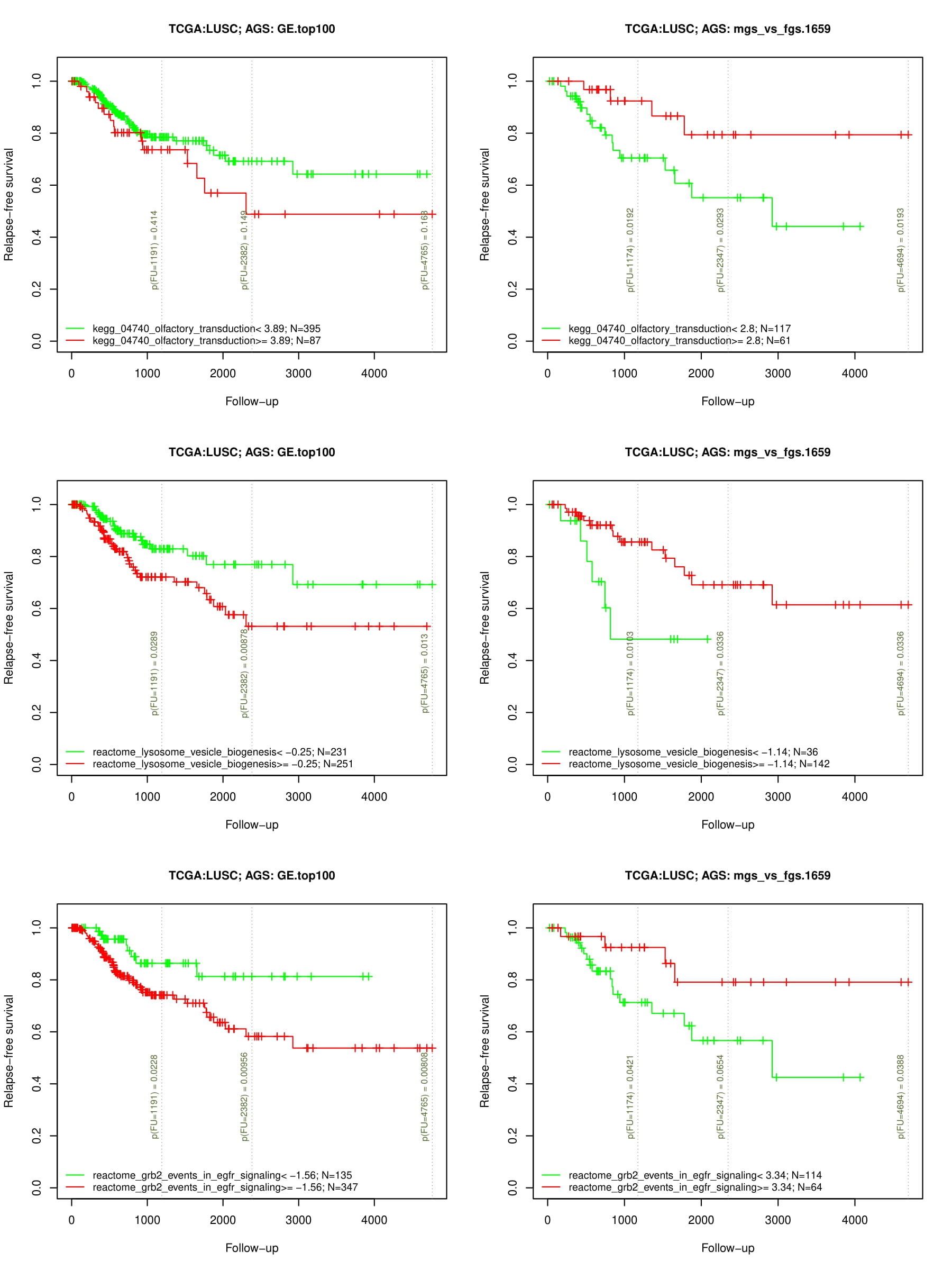

Supplement: Supplementary file 6. [file elife-74010-supp6.zip › SupplementaryFile6-83.jpg]

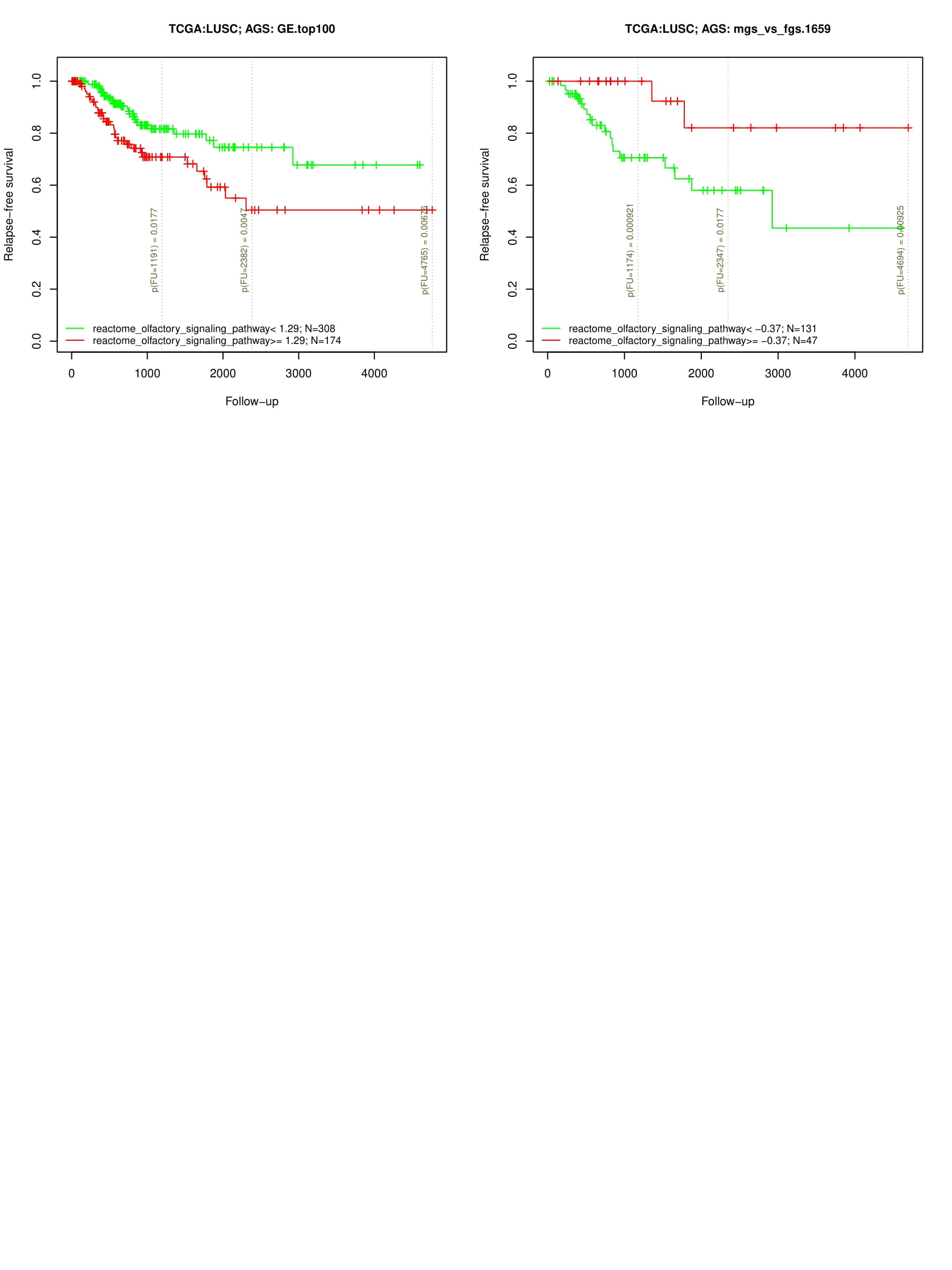

Supplement: Supplementary file 6. [file elife-74010-supp6.zip › SupplementaryFile6-84.jpg]

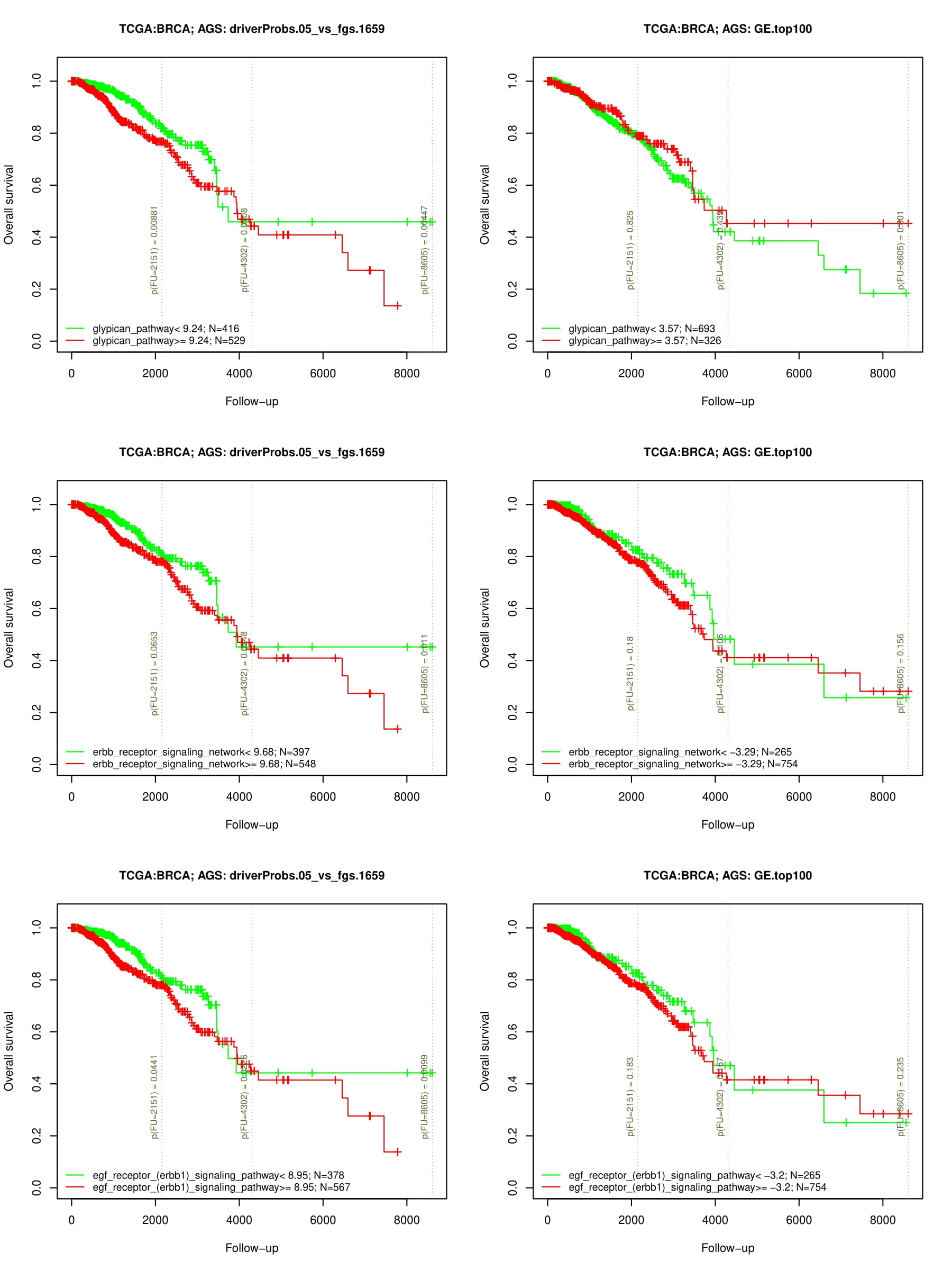

Supplement: Supplementary file 6. [file elife-74010-supp6.zip › SupplementaryFile6-85.jpg]

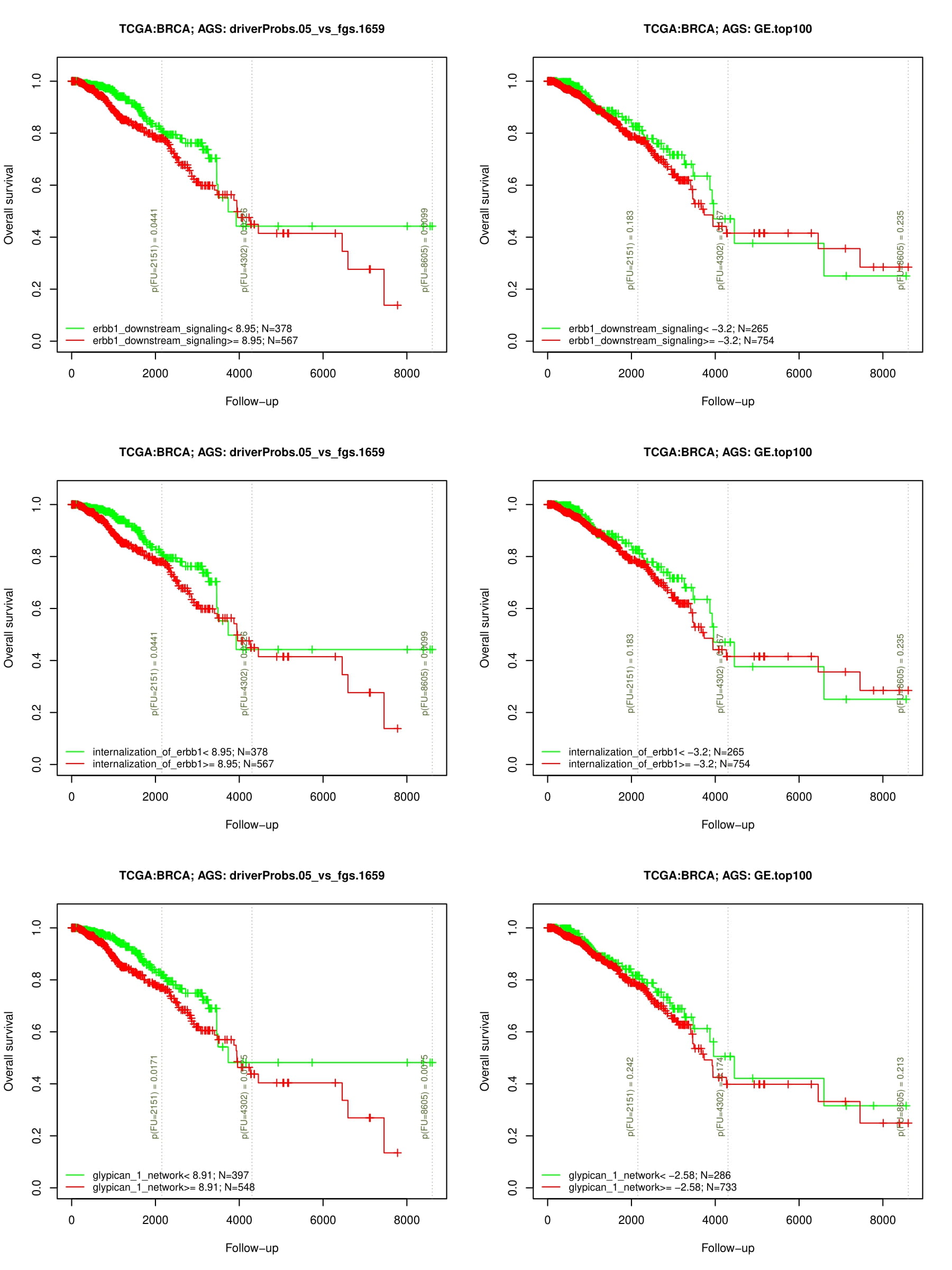

Supplement: Supplementary file 6. [file elife-74010-supp6.zip › SupplementaryFile6-86.jpg]

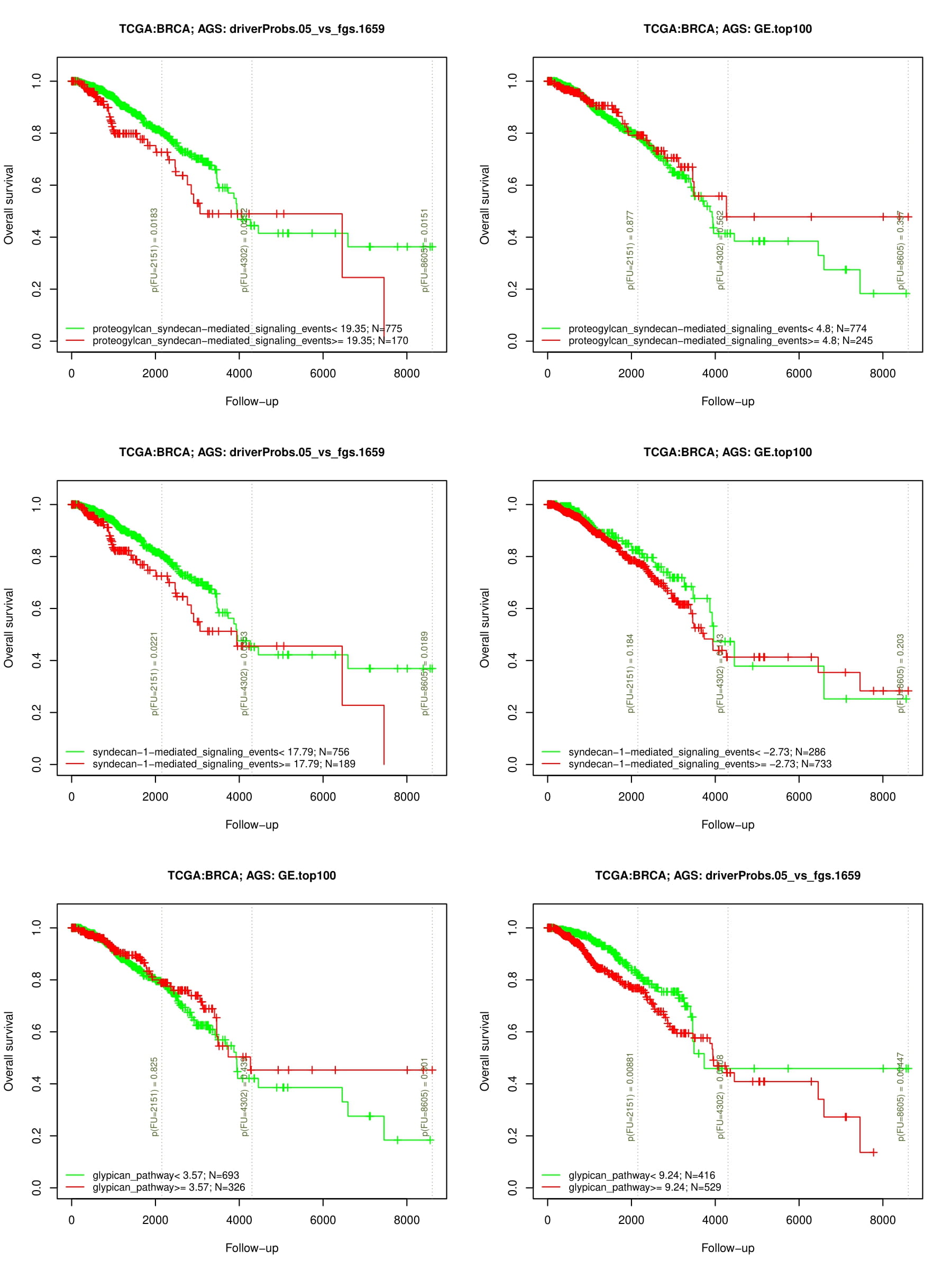

Supplement: Supplementary file 6. [file elife-74010-supp6.zip › SupplementaryFile6-87.jpg]

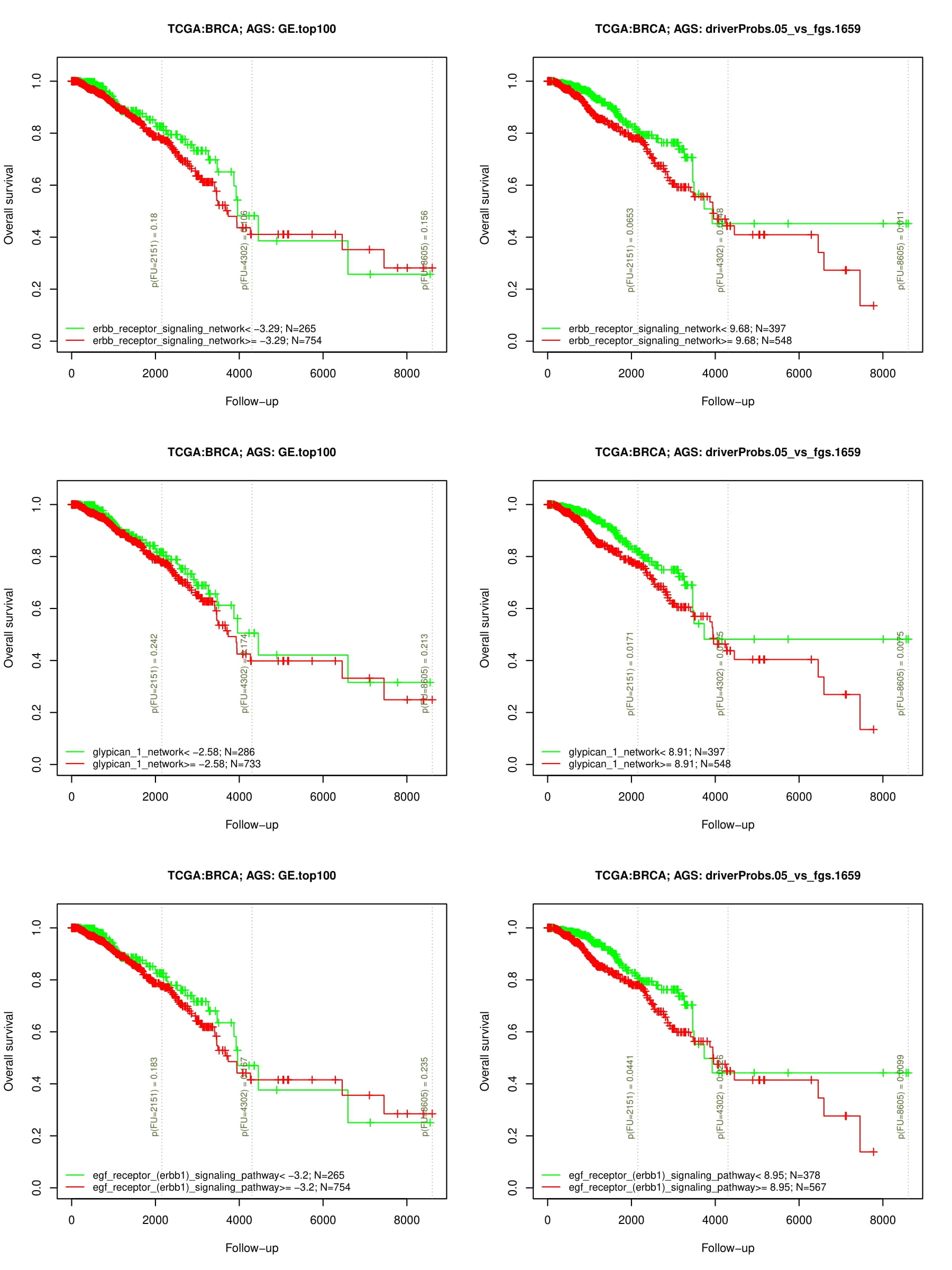

Supplement: Supplementary file 6. [file elife-74010-supp6.zip › SupplementaryFile6-88.jpg]

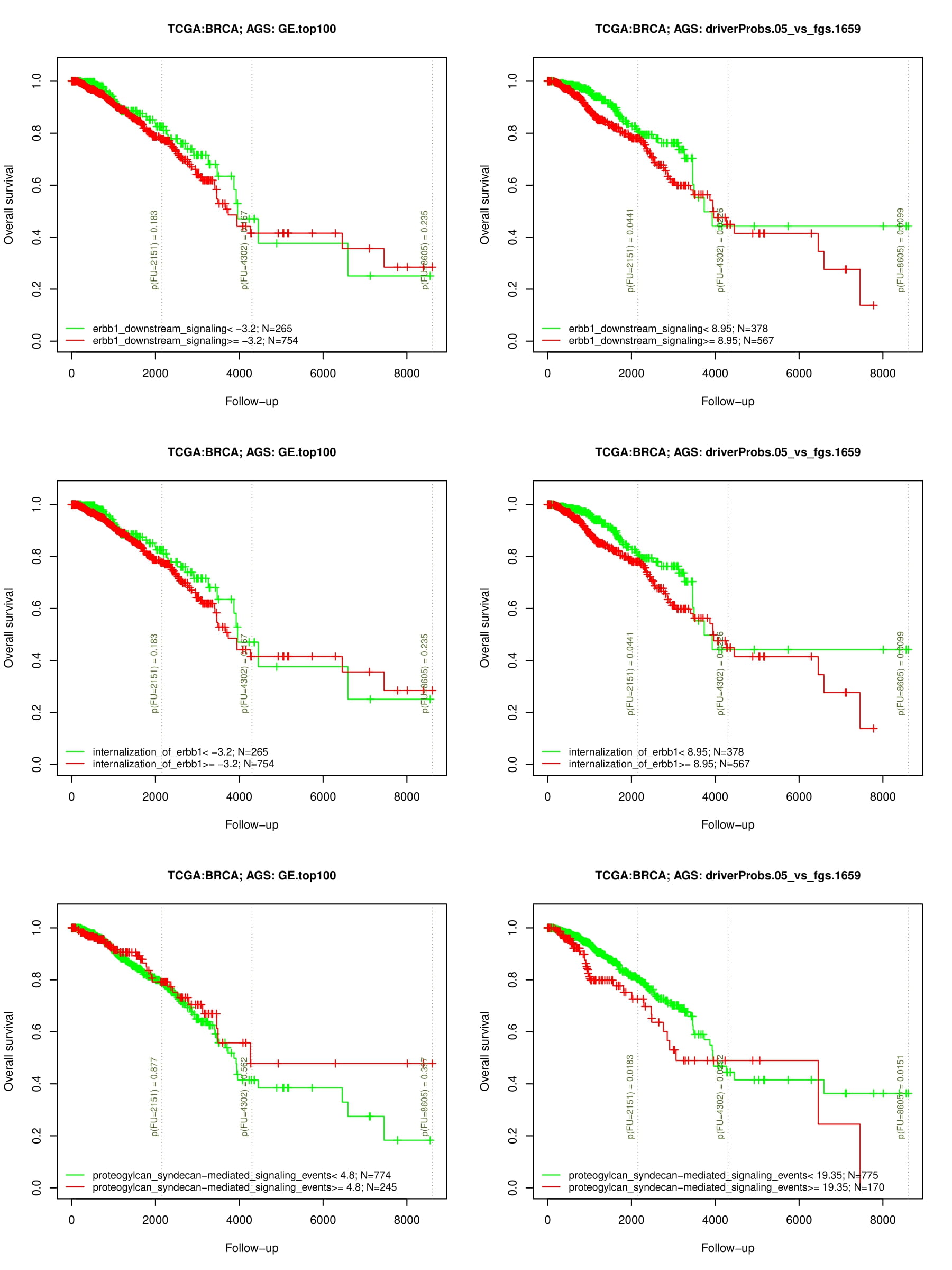

Supplement: Supplementary file 6. [file elife-74010-supp6.zip › SupplementaryFile6-89.jpg]

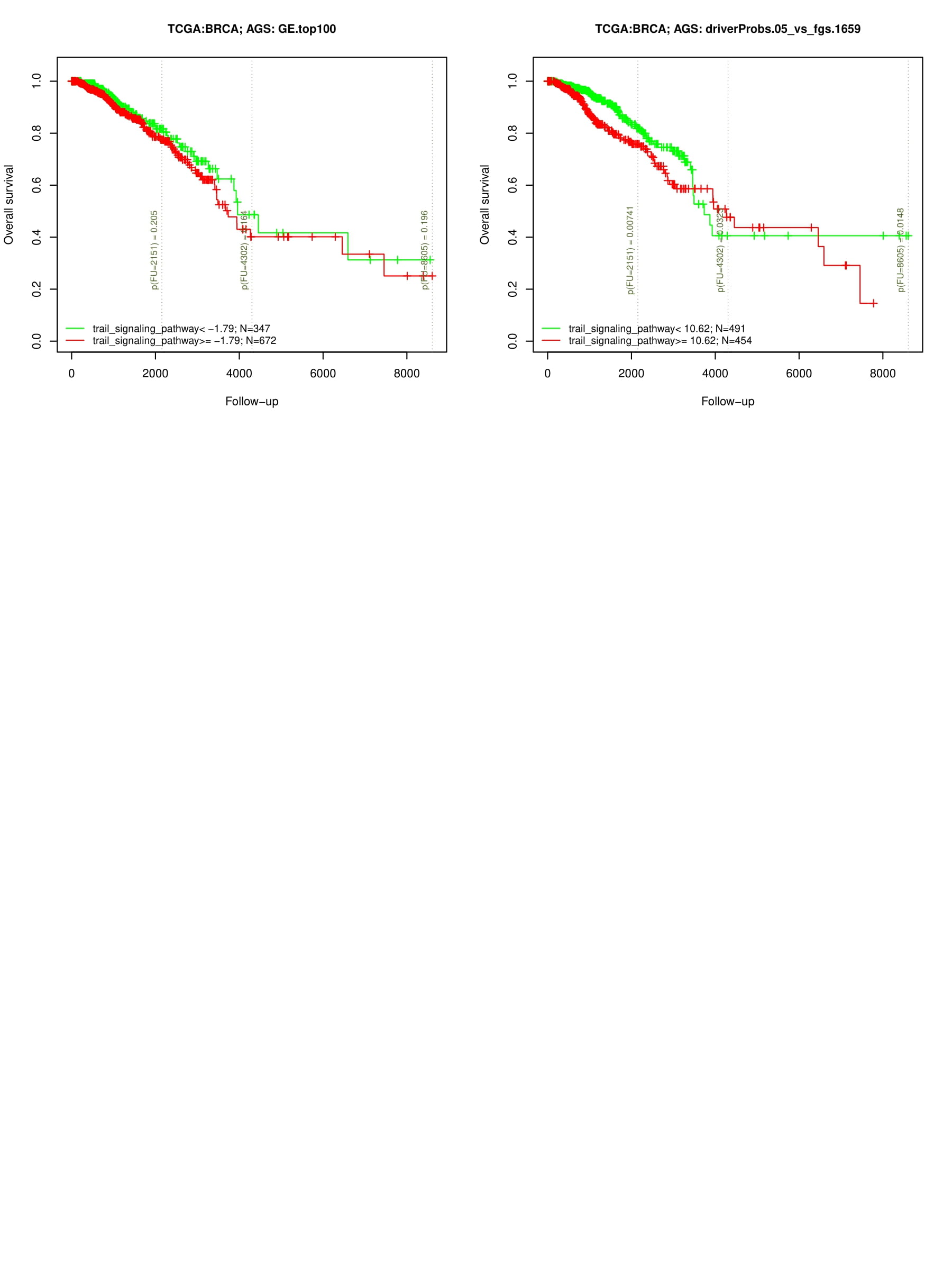

Supplement: Supplementary file 6. [file elife-74010-supp6.zip › SupplementaryFile6-90.jpg]

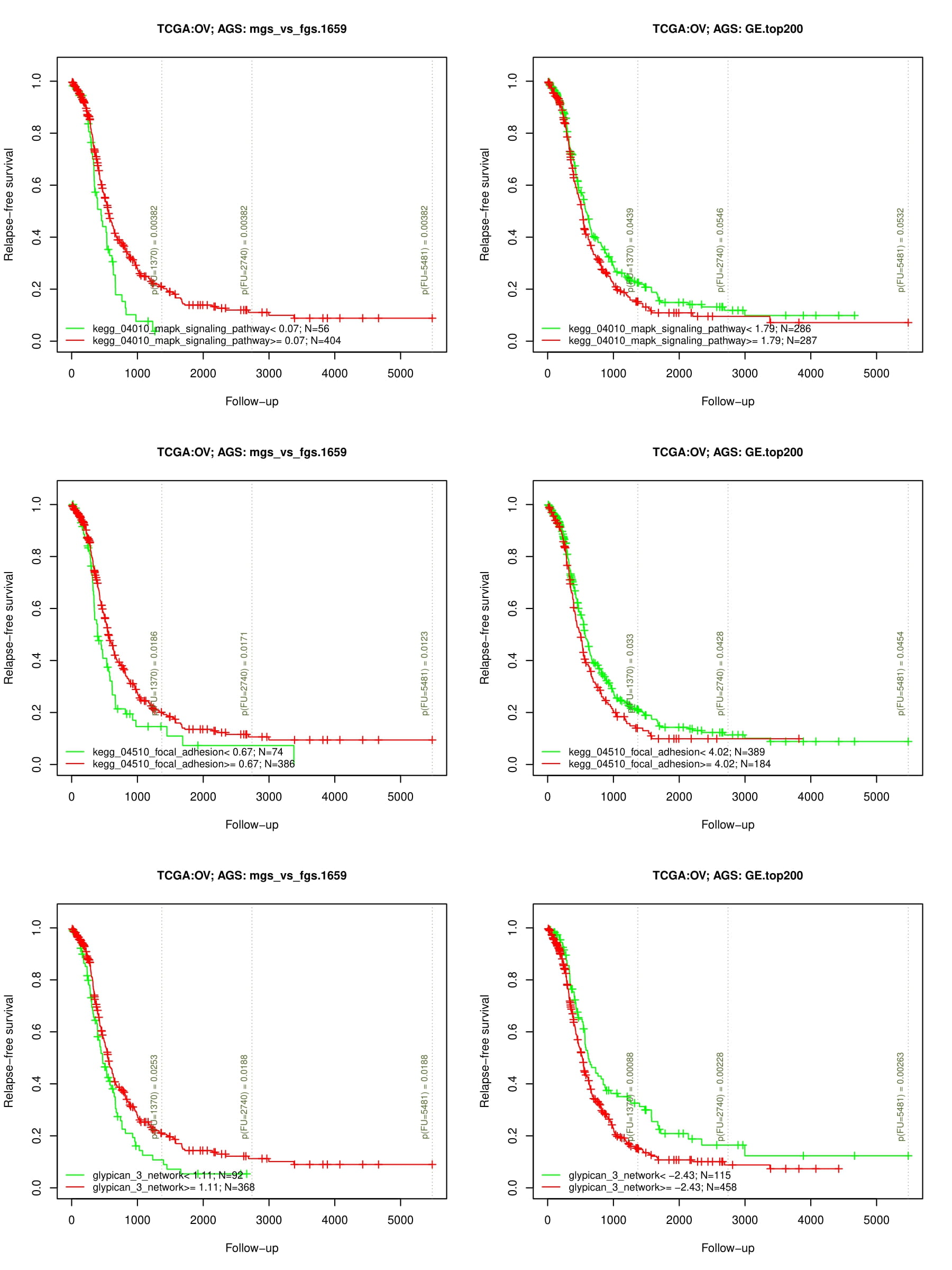

Supplement: Supplementary file 6. [file elife-74010-supp6.zip › SupplementaryFile6-91.jpg]

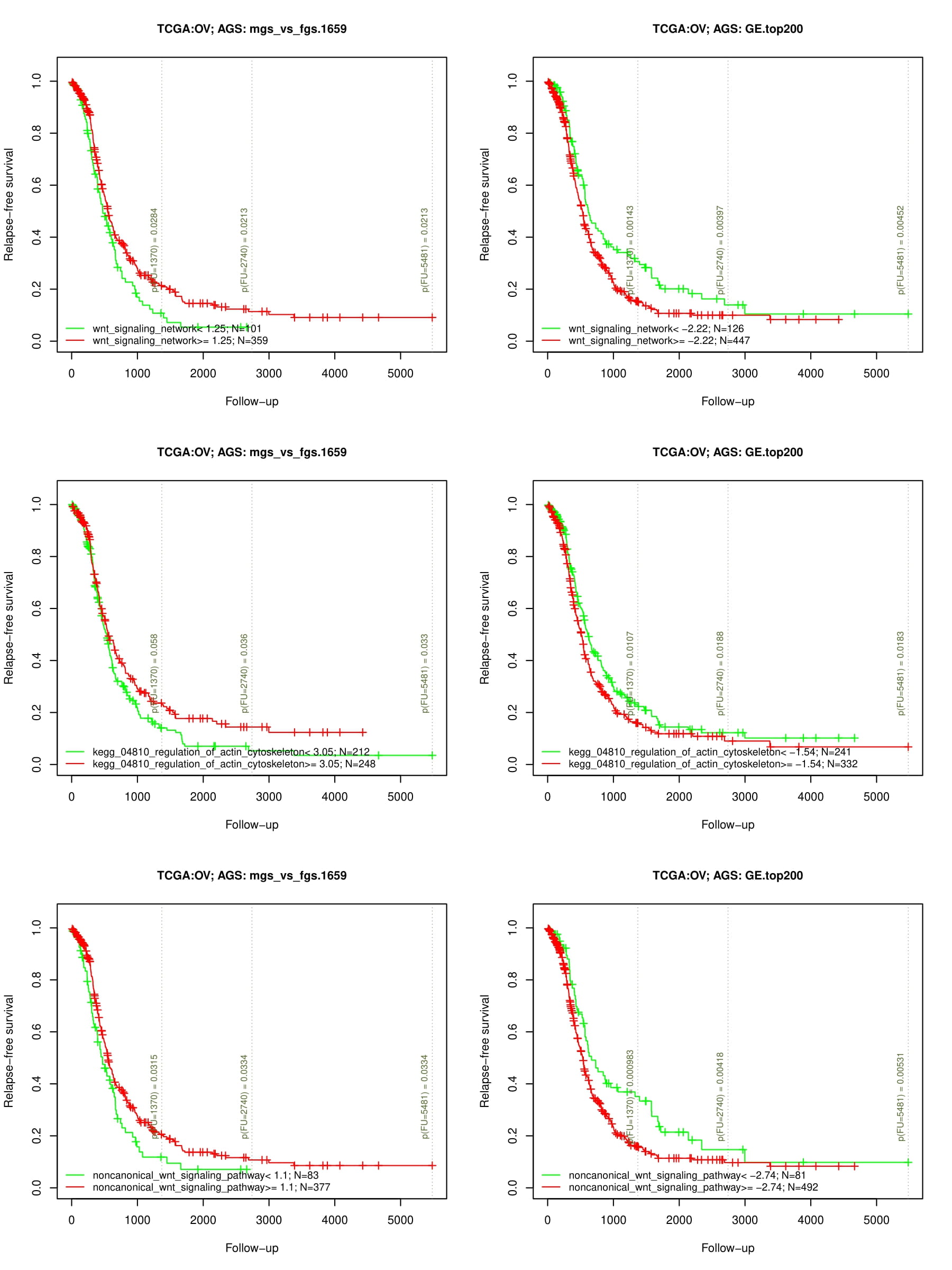

Supplement: Supplementary file 6. [file elife-74010-supp6.zip › SupplementaryFile6-92.jpg]

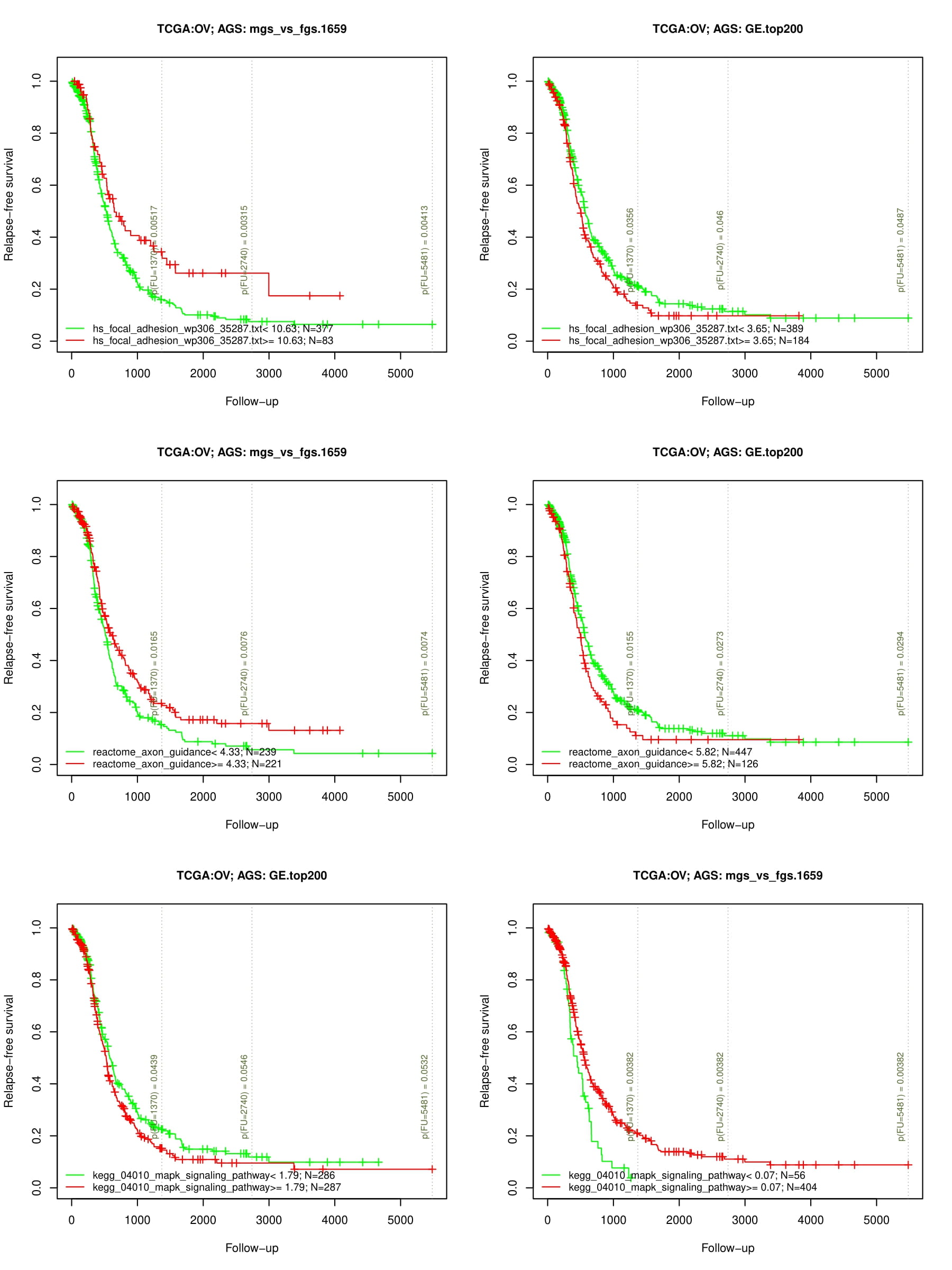

Supplement: Supplementary file 6. [file elife-74010-supp6.zip › SupplementaryFile6-93.jpg]

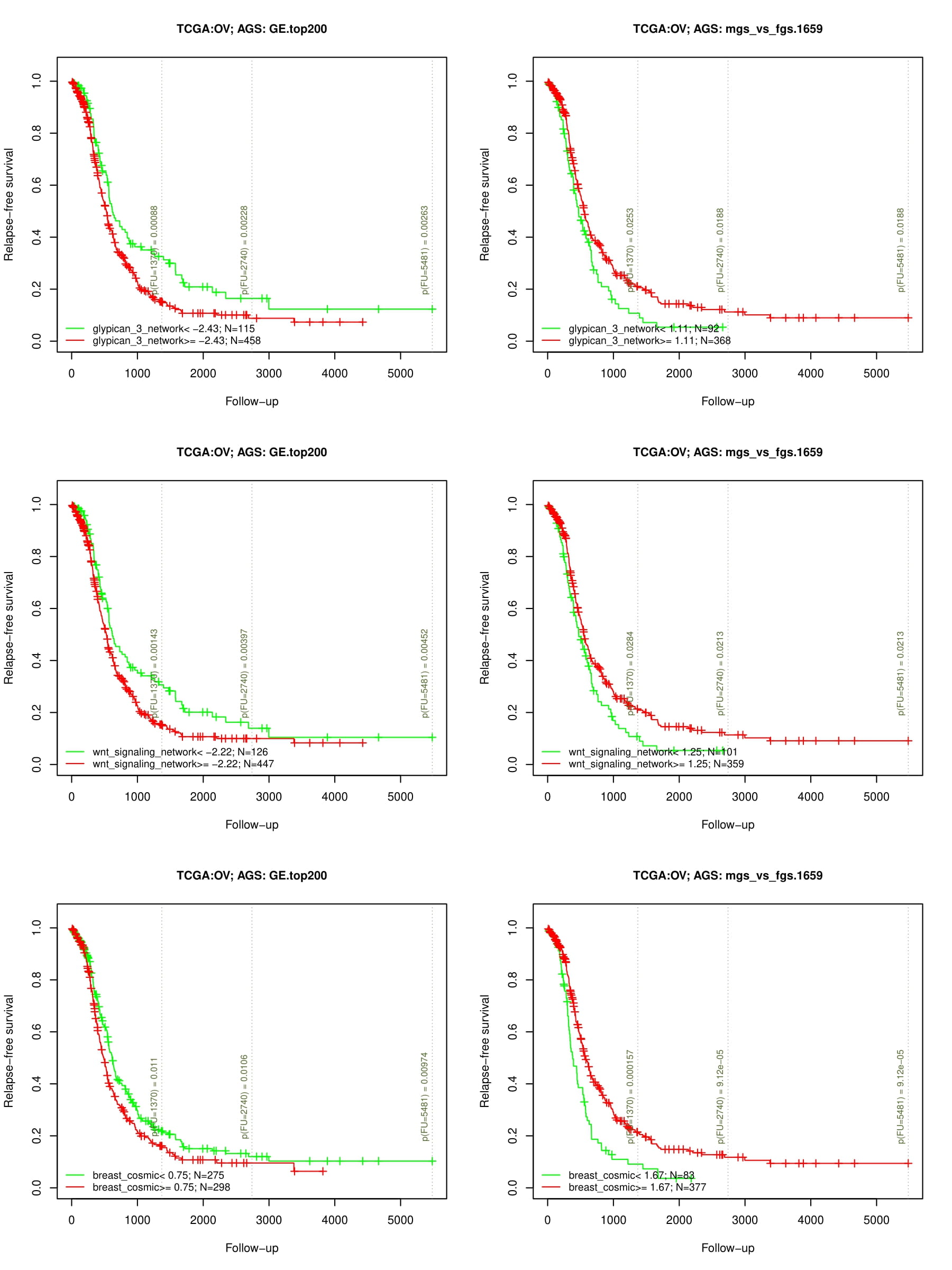

Supplement: Supplementary file 6. [file elife-74010-supp6.zip › SupplementaryFile6-94.jpg]

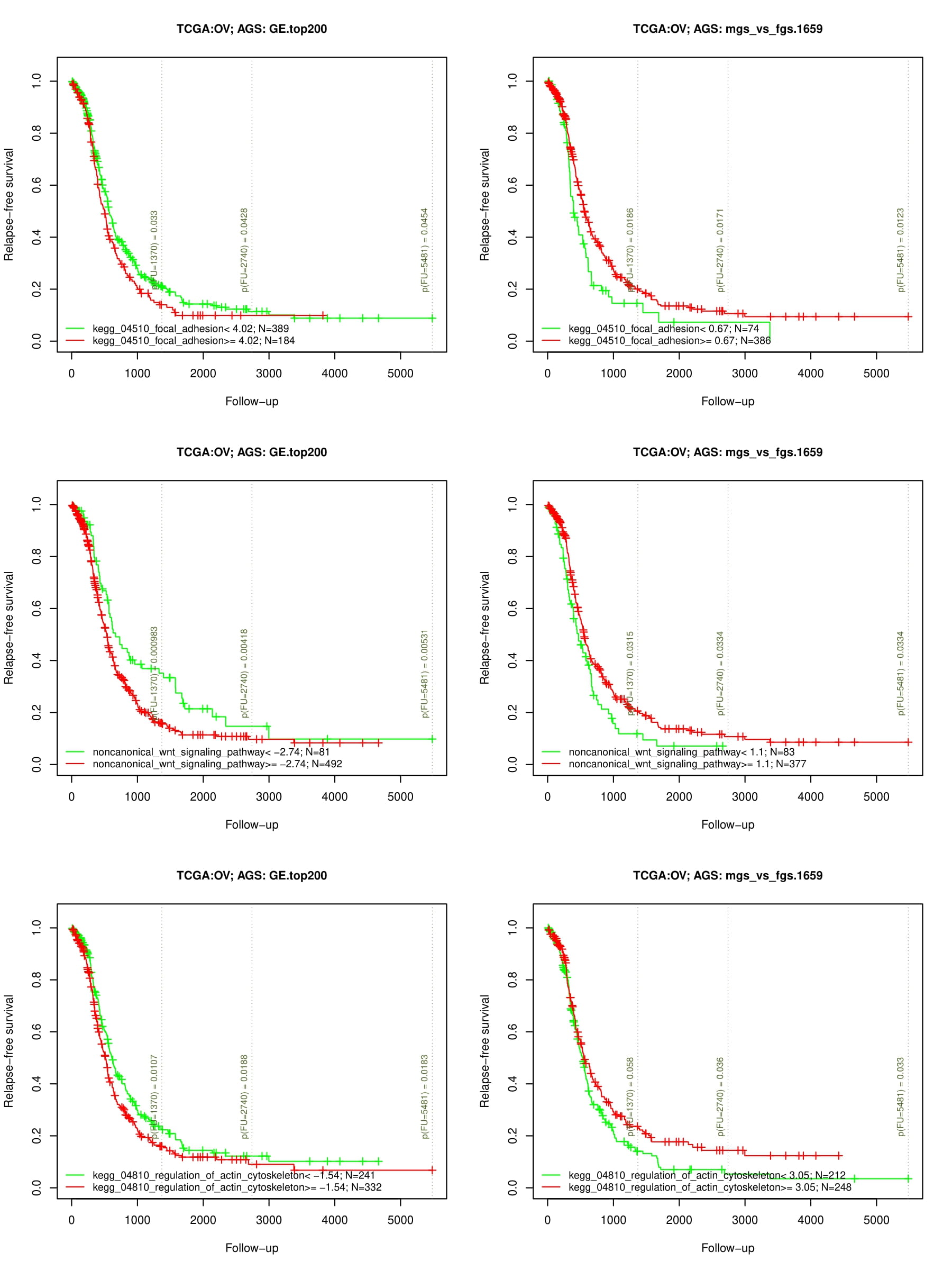

Supplement: Supplementary file 6. [file elife-74010-supp6.zip › SupplementaryFile6-95.jpg]

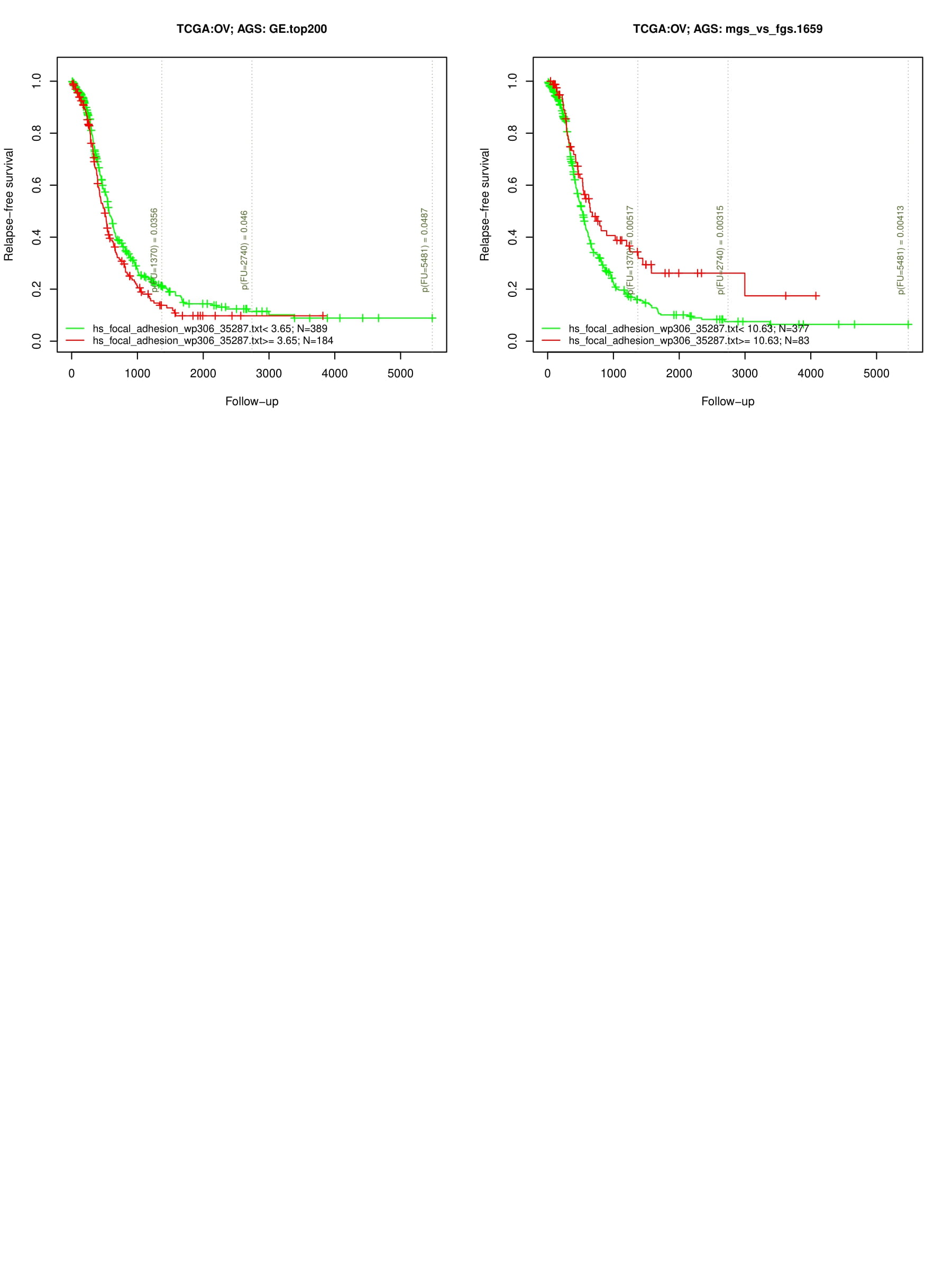

Supplement: Supplementary file 6. [file elife-74010-supp6.zip › SupplementaryFile6-96.jpg]

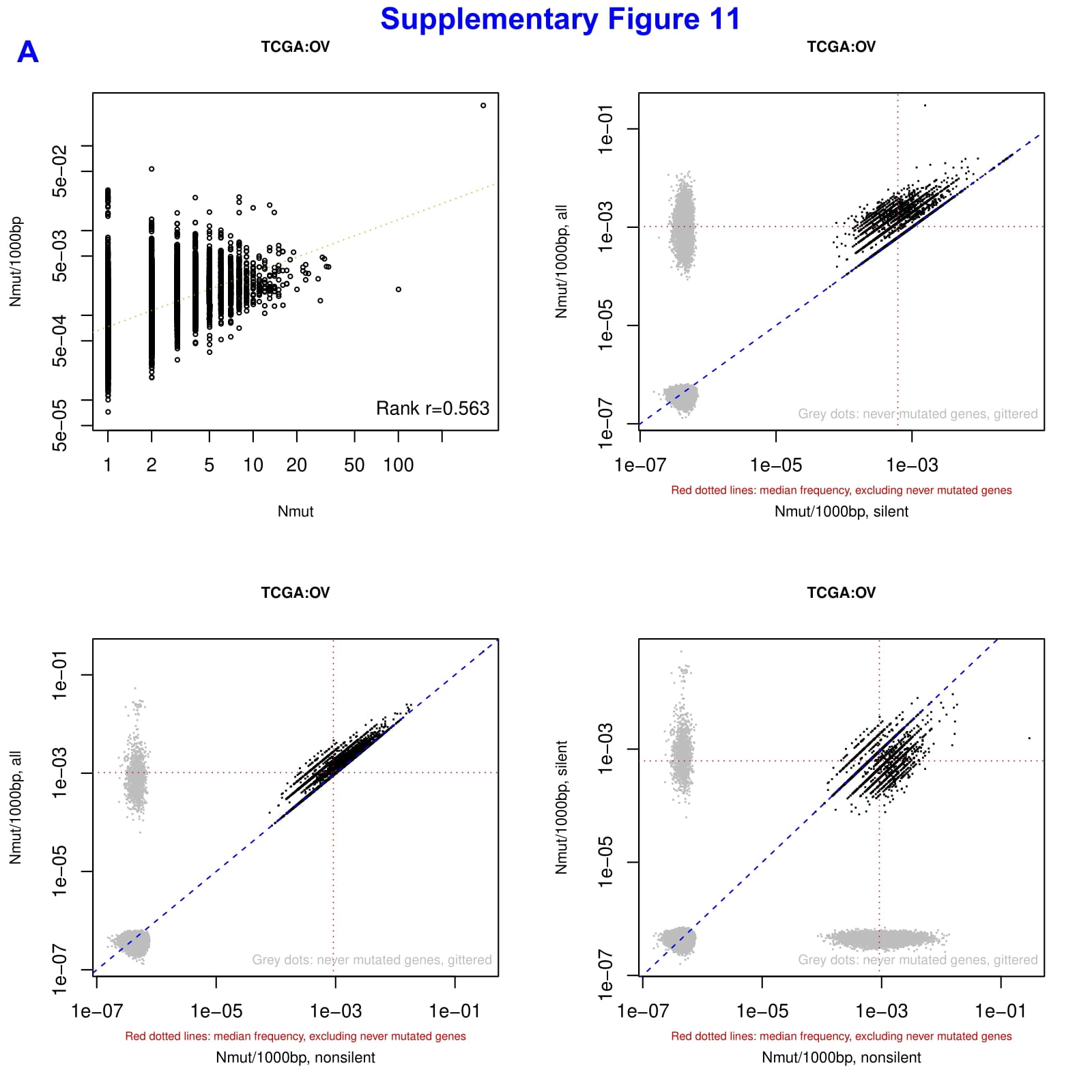

Supplement: Supplementary file 7. [file elife-74010-supp7.zip › SupplementaryFigure7.Nmut_vs_frequency-01.jpg]

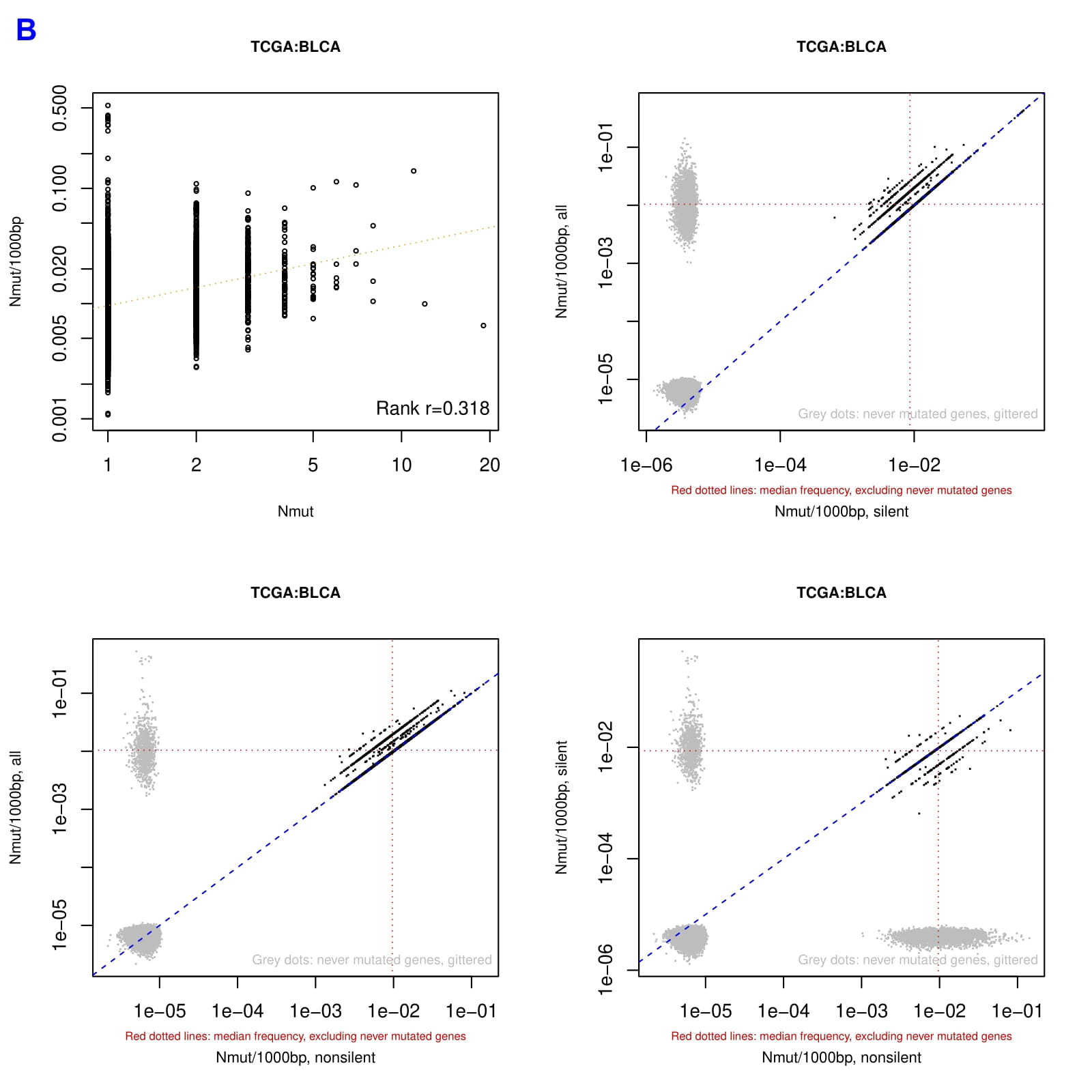

Supplement: Supplementary file 7. [file elife-74010-supp7.zip › SupplementaryFigure7.Nmut_vs_frequency-02.jpg]

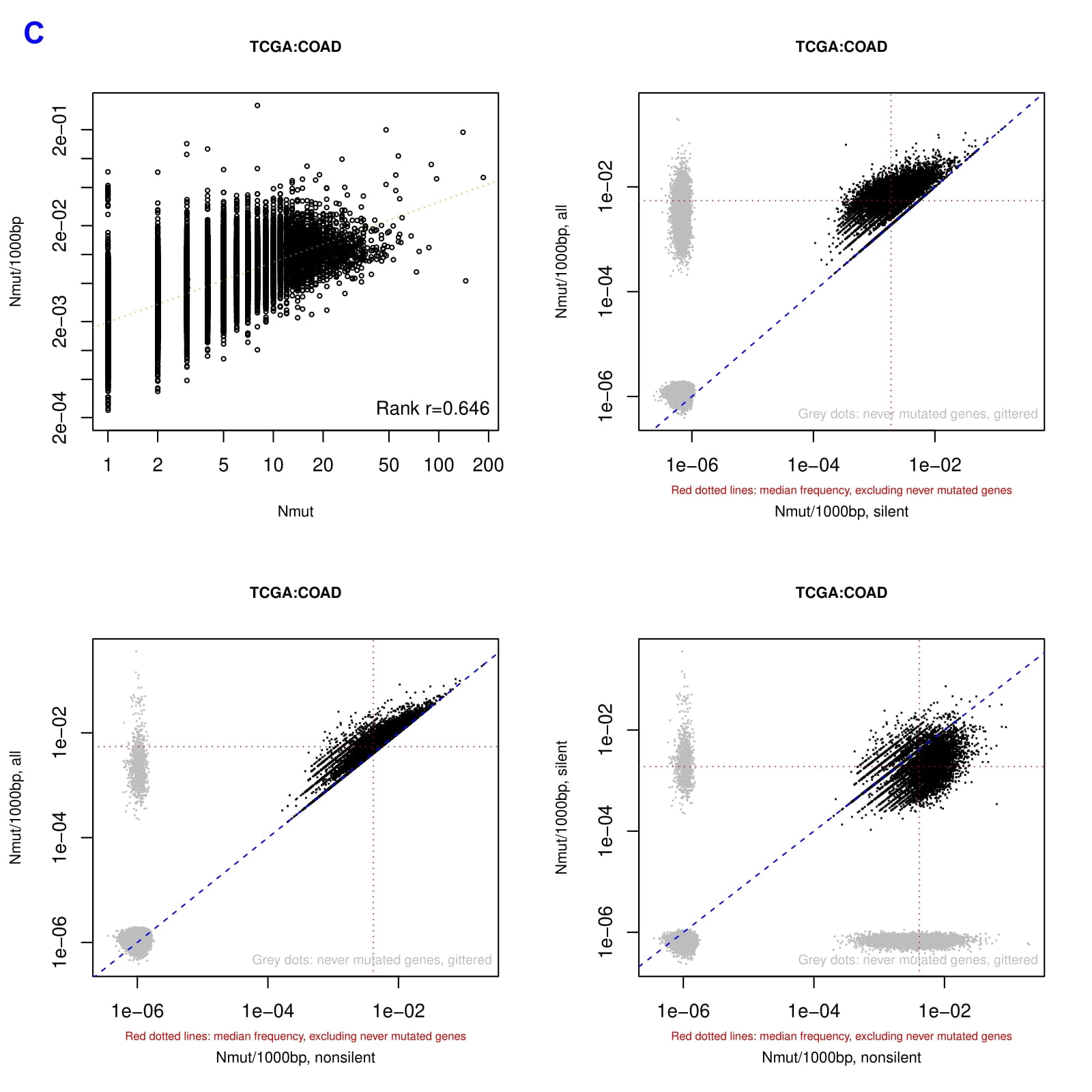

Supplement: Supplementary file 7. [file elife-74010-supp7.zip › SupplementaryFigure7.Nmut_vs_frequency-03.jpg]

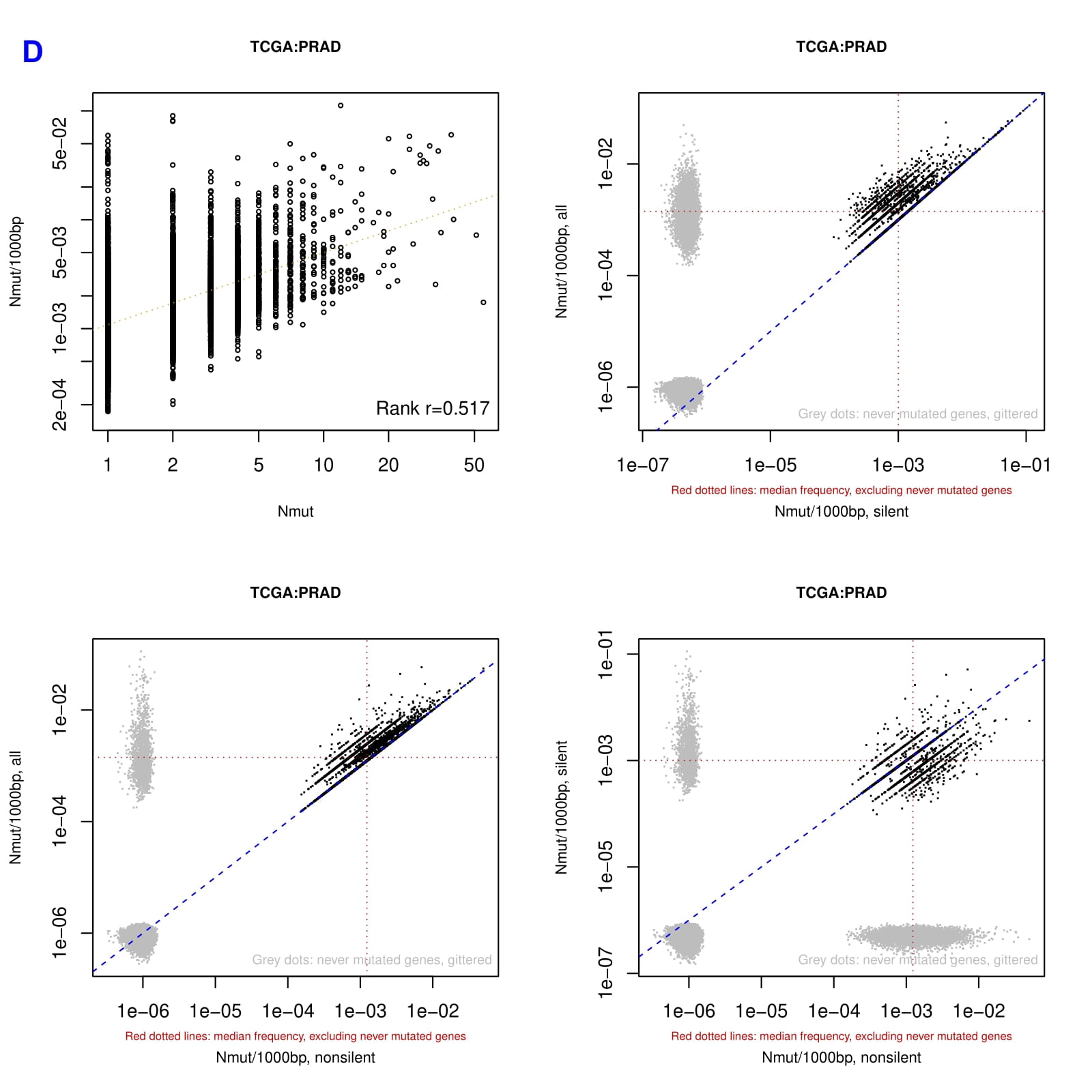

Supplement: Supplementary file 7. [file elife-74010-supp7.zip › SupplementaryFigure7.Nmut_vs_frequency-04.jpg]

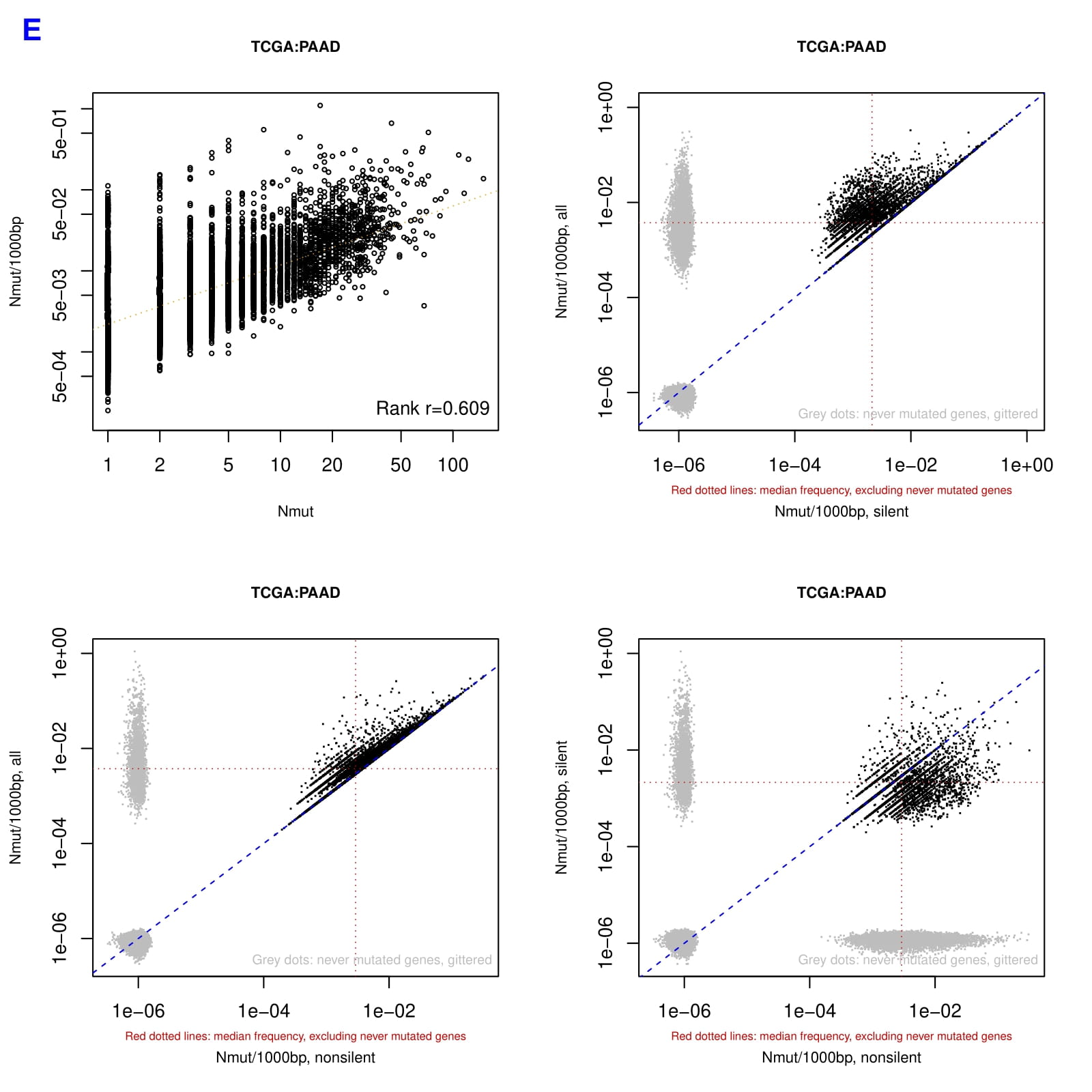

Supplement: Supplementary file 7. [file elife-74010-supp7.zip › SupplementaryFigure7.Nmut_vs_frequency-05.jpg]

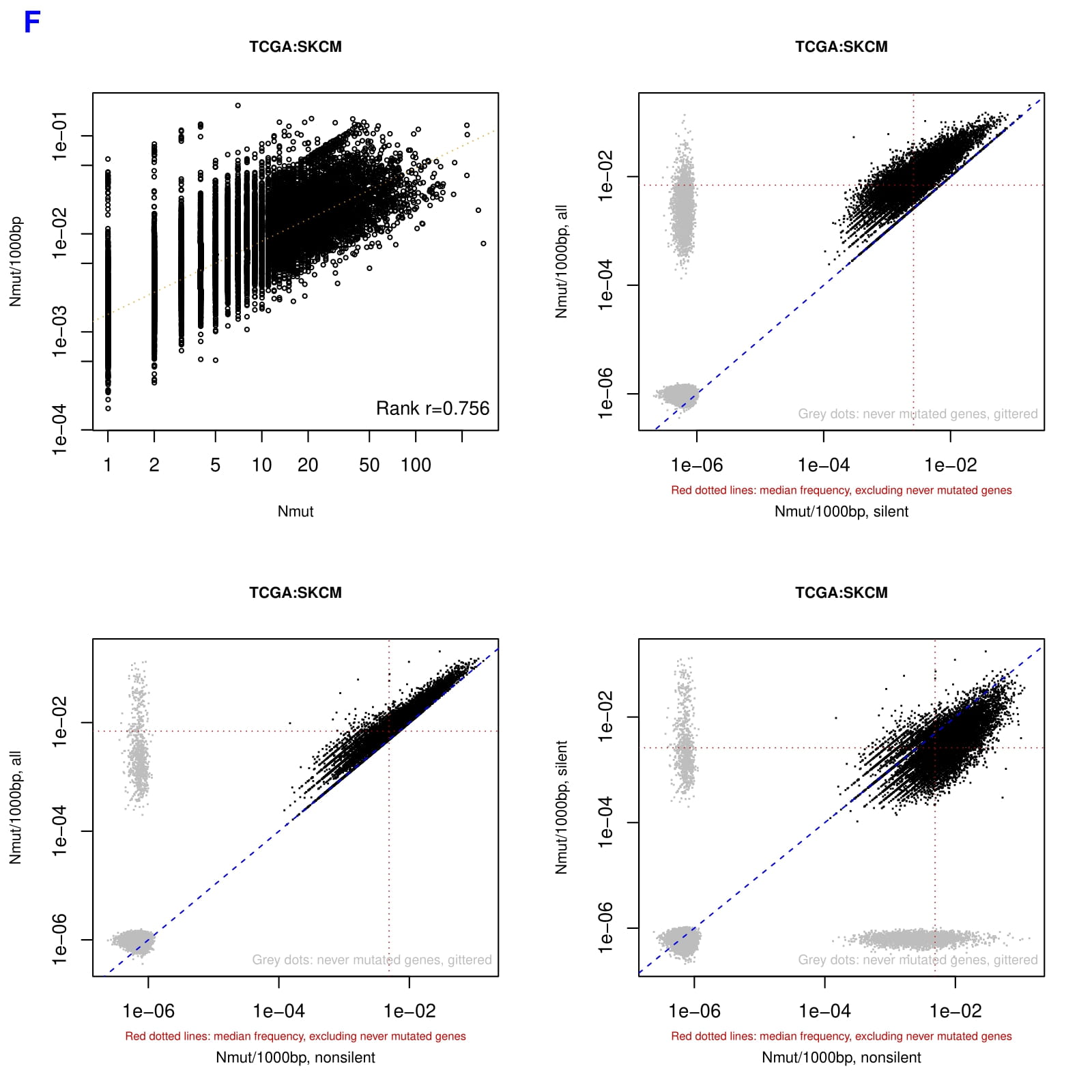

Supplement: Supplementary file 7. [file elife-74010-supp7.zip › SupplementaryFigure7.Nmut_vs_frequency-06.jpg]

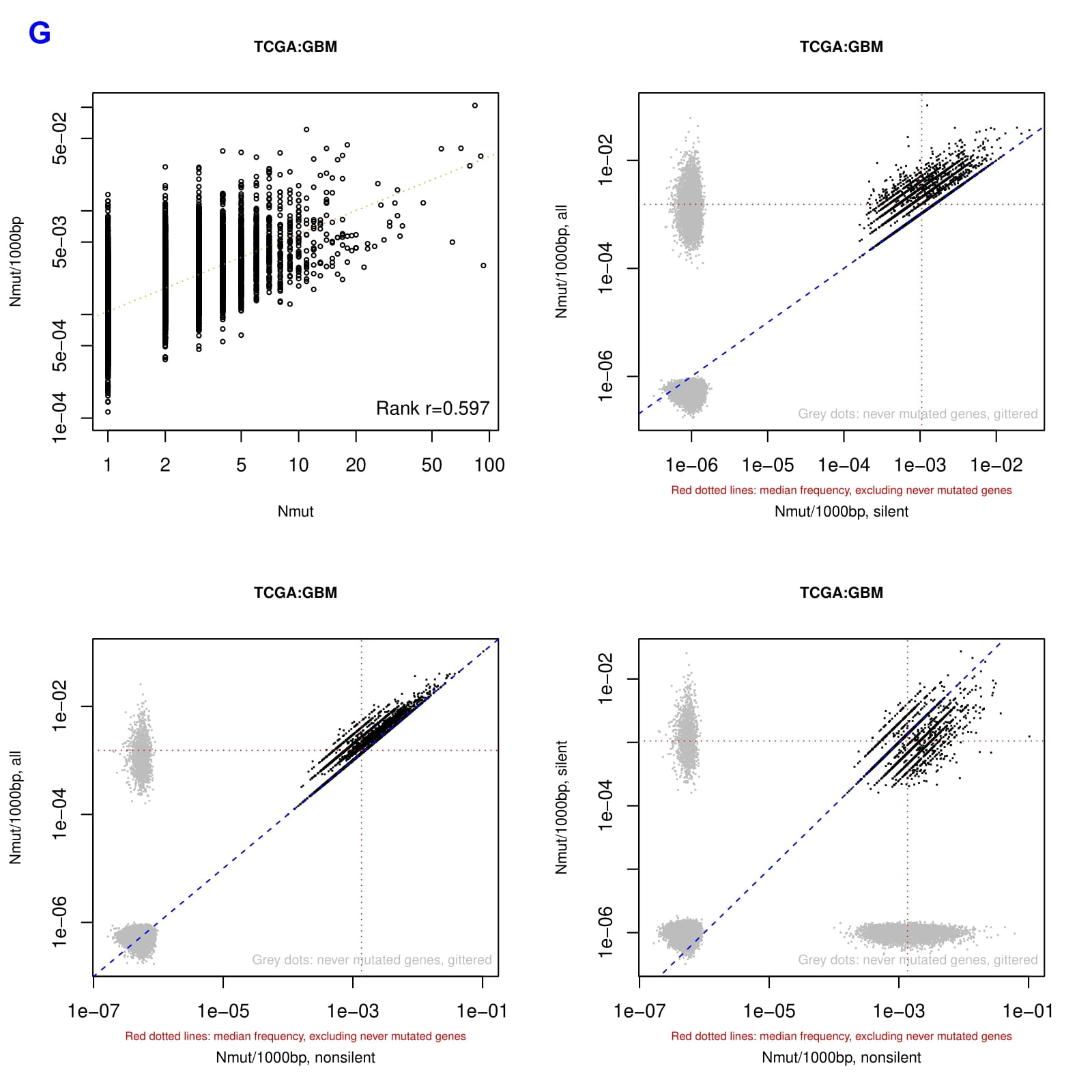

Supplement: Supplementary file 7. [file elife-74010-supp7.zip › SupplementaryFigure7.Nmut_vs_frequency-07.jpg]

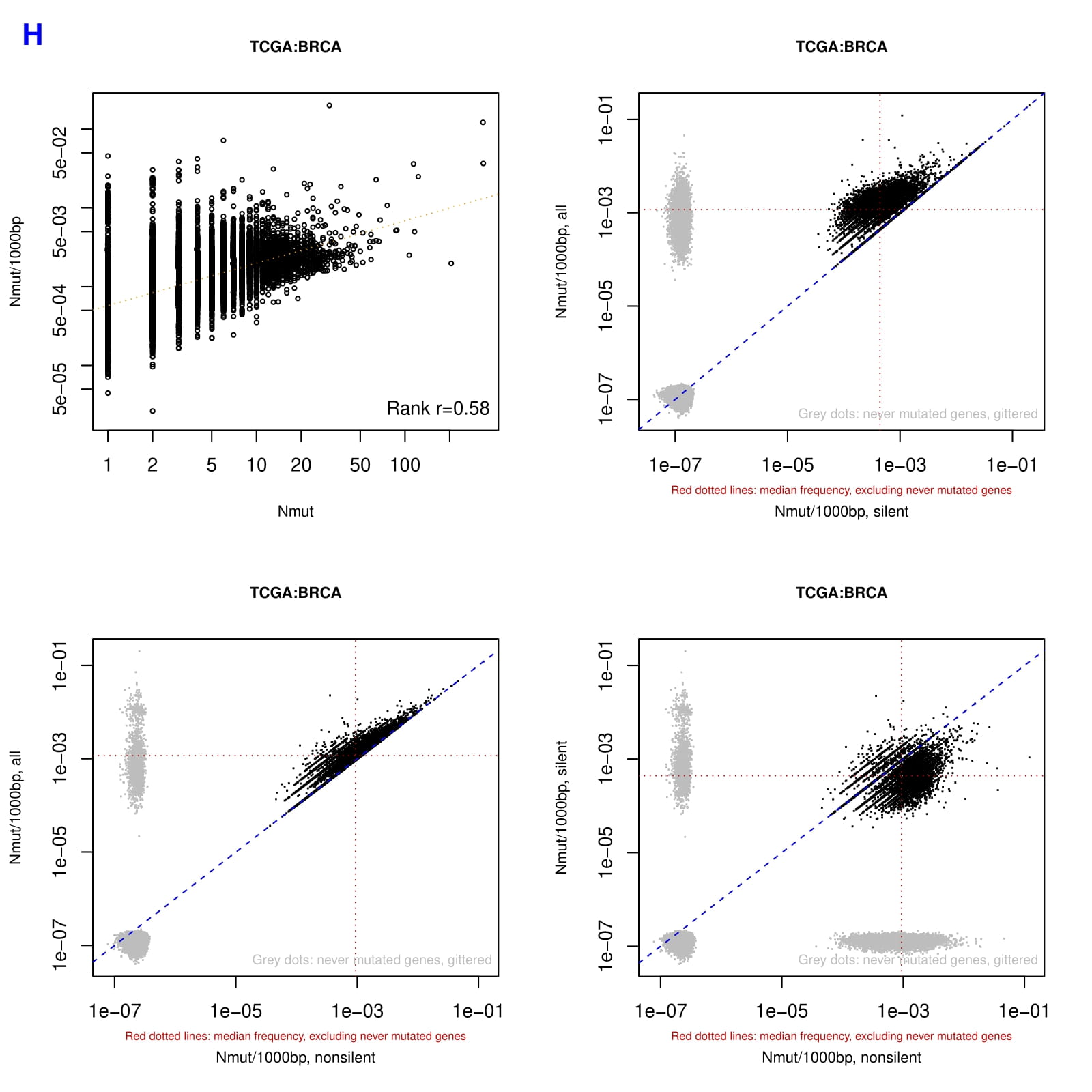

Supplement: Supplementary file 7. [file elife-74010-supp7.zip › SupplementaryFigure7.Nmut_vs_frequency-08.jpg]

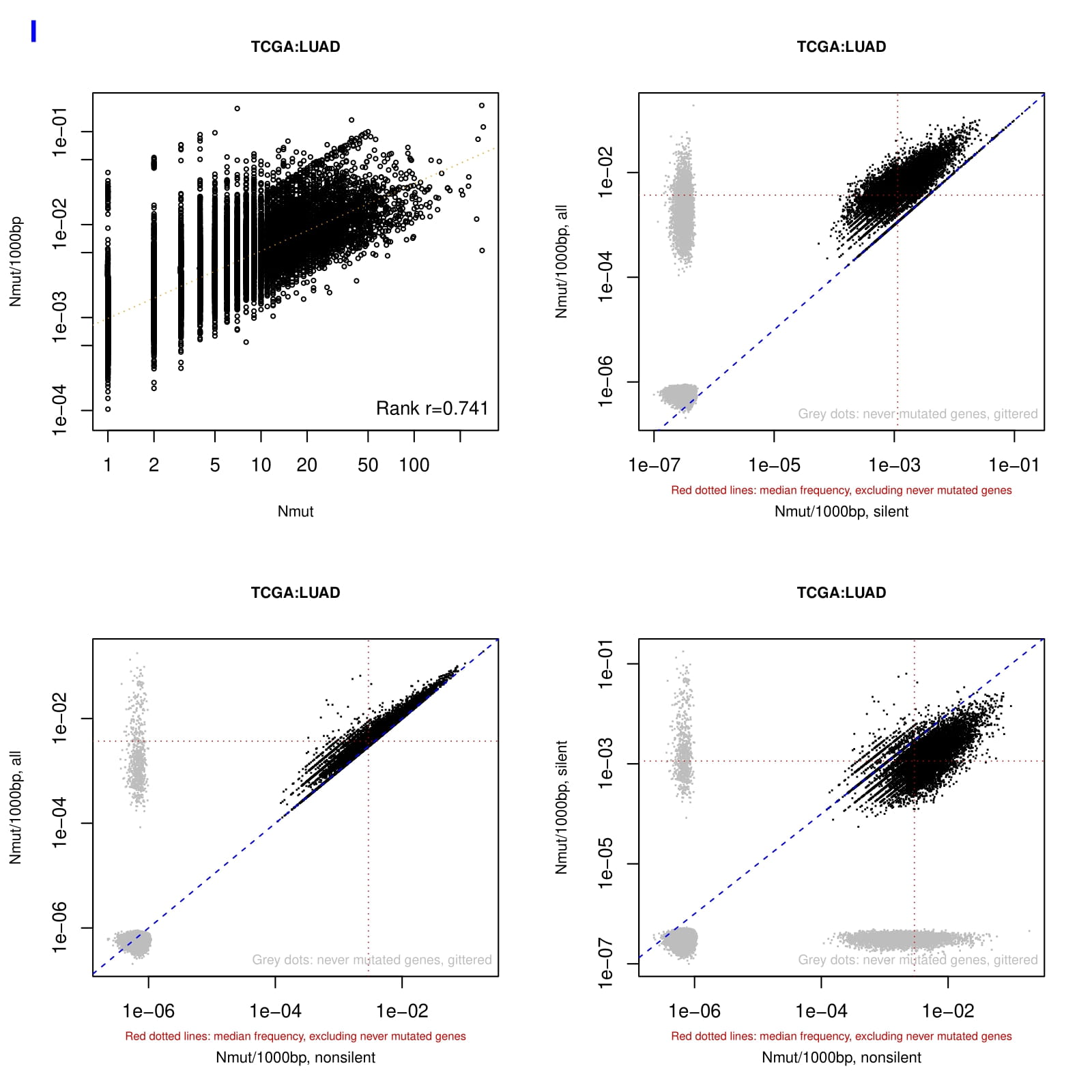

Supplement: Supplementary file 7. [file elife-74010-supp7.zip › SupplementaryFigure7.Nmut_vs_frequency-09.jpg]

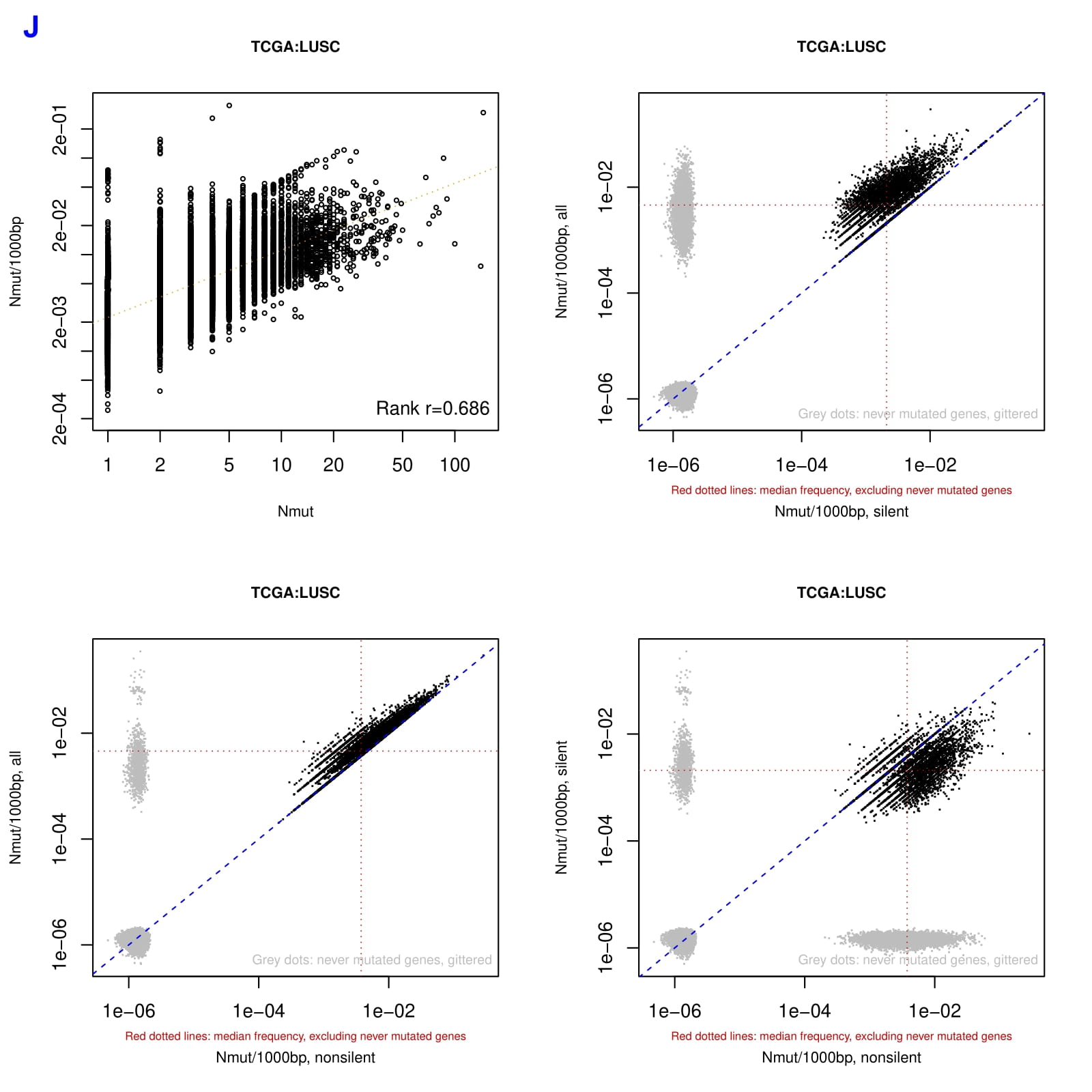

Supplement: Supplementary file 7. [file elife-74010-supp7.zip › SupplementaryFigure7.Nmut_vs_frequency-10.jpg]

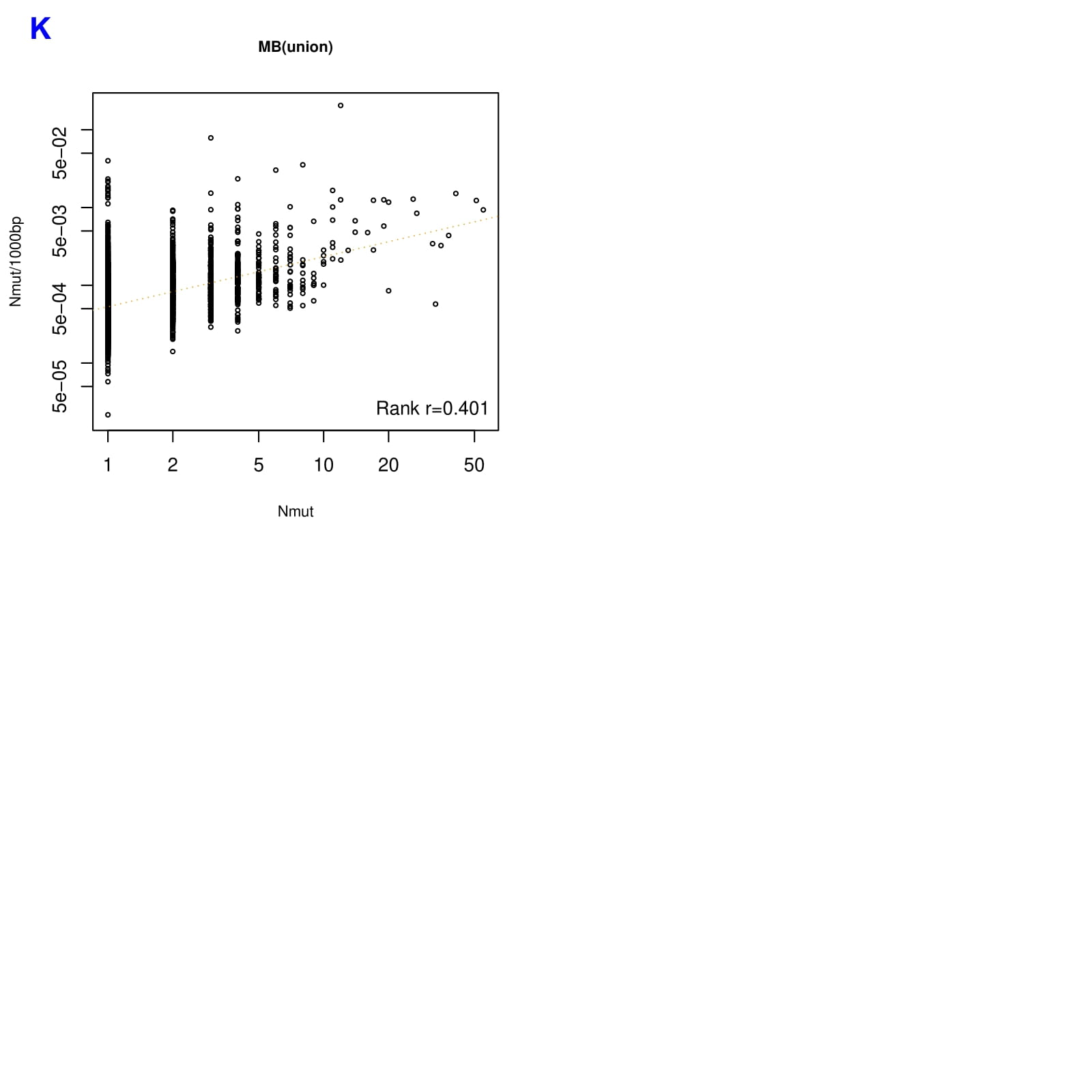

Supplement: Supplementary file 7. [file elife-74010-supp7.zip › SupplementaryFigure7.Nmut_vs_frequency-11.jpg]
