# Supplementary material for: Individualized discovery of rare cancer drivers in global network context
Source: eLife. 2022 May 20;11:e74010. doi: 10.7554/eLife.74010 (PMC9159755; doi:10.7554/eLife.74010)
Supplement: Supplementary file 7. [file elife-74010-supp7.zip › SupplementaryFigure7.Nmut_vs_frequency.pdf]

Supplementary Figure 11

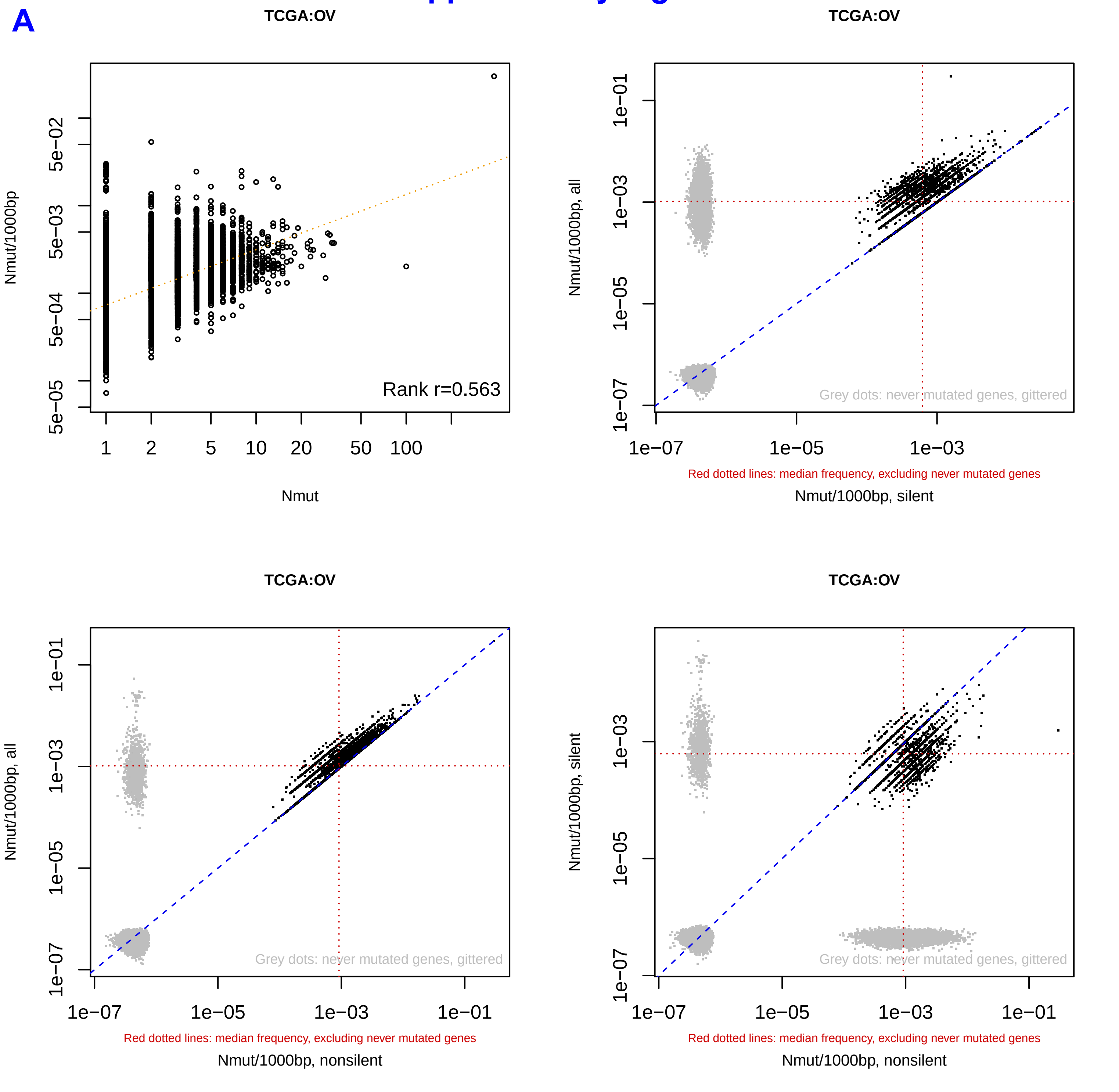

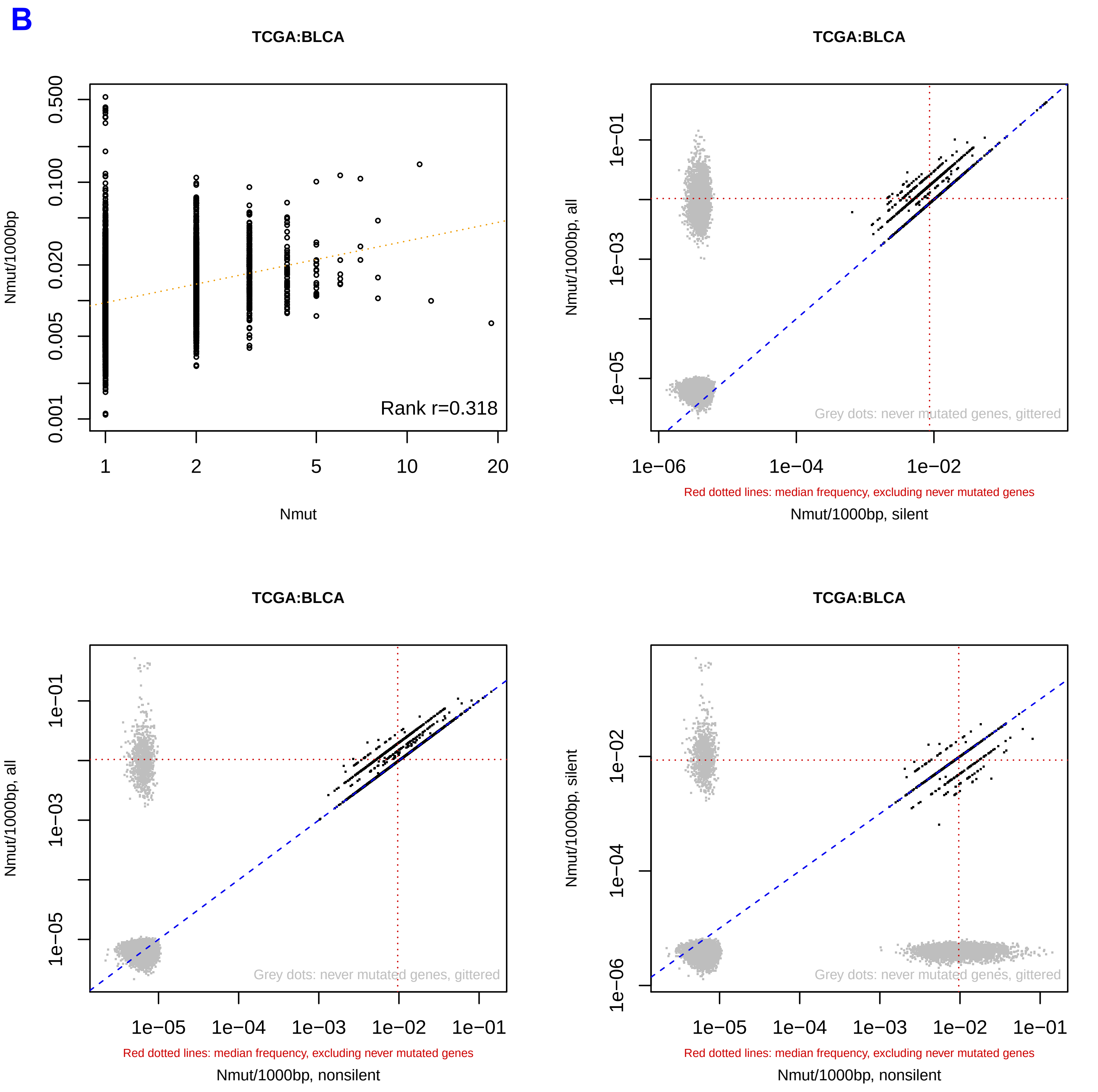
B
TCGA:BLCA
Nmut/1000bp
Nmut
Rank r=0.318
TCGA:BLCA
Nmut/1000bp, all
Nmut/1000bp, silent
Grey dots: never mutated genes, gittered
Red dotted lines: median frequency, excluding never mutated genes
TCGA:BLCA
Nmut/1000bp, all
Nmut/1000bp, nonsilent
Grey dots: never mutated genes, gittered
Red dotted lines: median frequency, excluding never mutated genes
TCGA:BLCA
Nmut/1000bp, silent
Nmut/1000bp, nonsilent
Grey dots: never mutated genes, gittered
Red dotted lines: median frequency, excluding never mutated genes

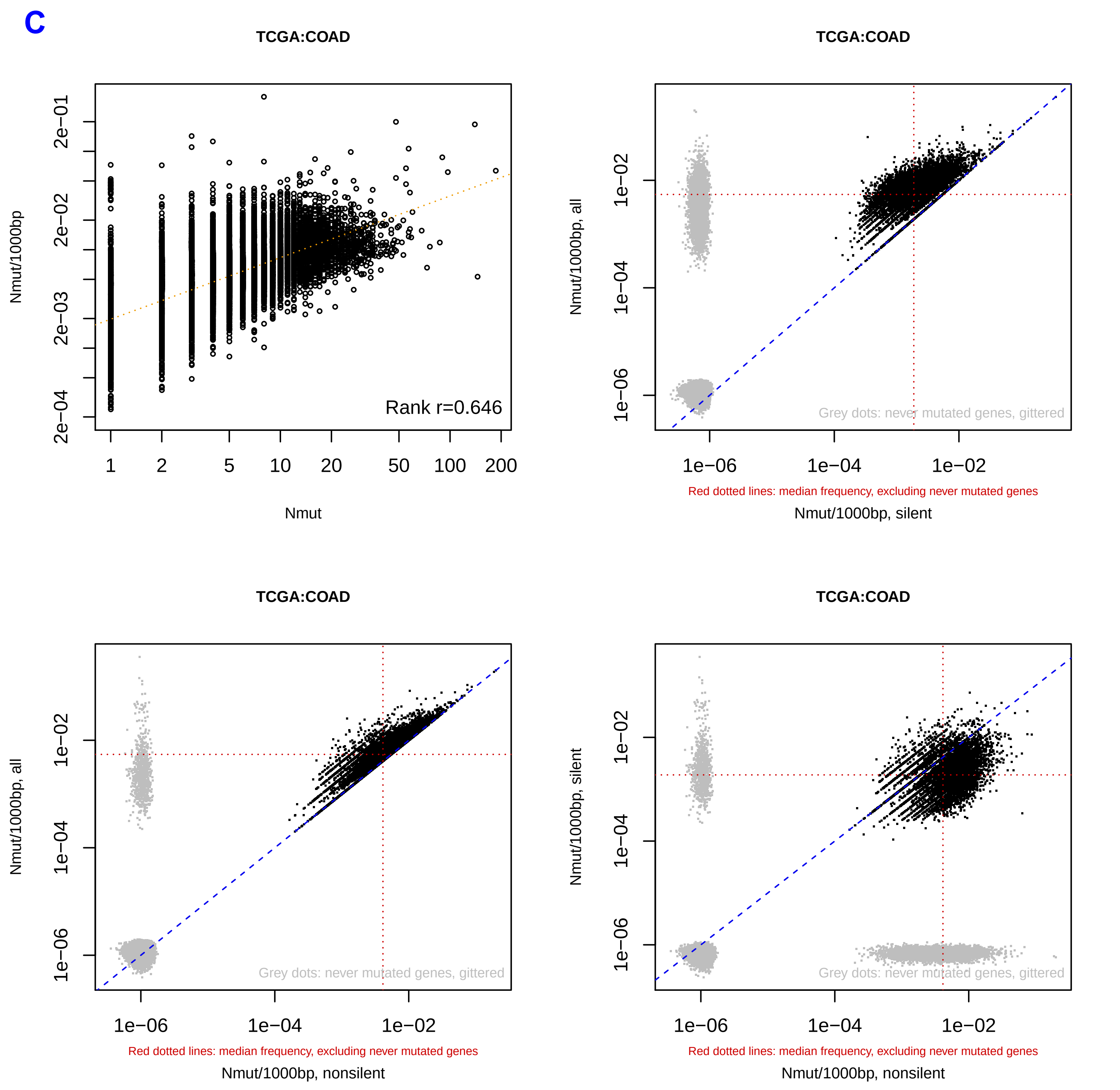

C
TCGA:COAD
Nmut/1000bp
2e−01
2e−02
2e−03
2e−04
1
2
5
10
20
50
100
200
Nmut
Rank r=0.646
TCGA:COAD
Nmut/1000bp, all
1e−02
1e−04
1e−06
1e−06
1e−04
1e−02
Grey dots: never mutated genes, gittered
Red dotted lines: median frequency, excluding never mutated genes
Nmut/1000bp, silent
TCGA:COAD
Nmut/1000bp, all
Grey dots: never mutated genes, gittered
Red dotted lines: median frequency, excluding never mutated genes
Nmut/1000bp, nonsilent
TCGA:COAD
Nmut/1000bp, silent
Grey dots: never mutated genes, gittered
Red dotted lines: median frequency, excluding never mutated genes
Nmut/1000bp, nonsilent

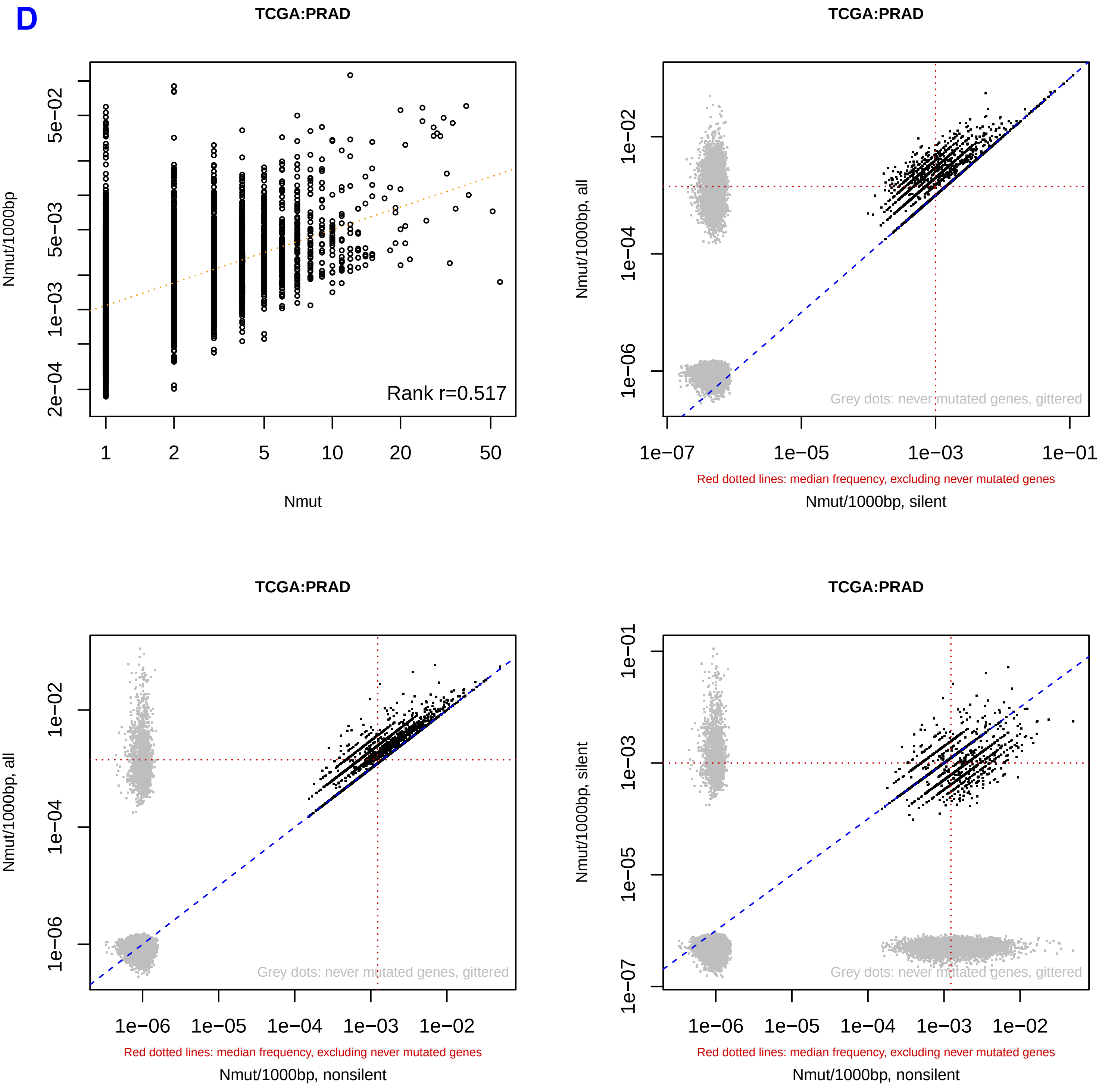
D
TCGA:PRAD
Nmut/1000bp
Nmut
Rank r=0.517
1
2
5
10
20
50
2e−04
1e−03
5e−03
5e−02
TCGA:PRAD
Nmut/1000bp, all
Nmut/1000bp, silent
Red dotted lines: median frequency, excluding never mutated genes
Grey dots: never mutated genes, gittered
1e−07
1e−05
1e−03
1e−01
1e−06
1e−04
1e−02
TCGA:PRAD
Nmut/1000bp, all
Nmut/1000bp, nonsilent
Red dotted lines: median frequency, excluding never mutated genes
Grey dots: never mutated genes, gittered
1e−06
1e−05
1e−04
1e−03
1e−02
1e−06
1e−04
1e−02
TCGA:PRAD
Nmut/1000bp, silent
Nmut/1000bp, nonsilent
Red dotted lines: median frequency, excluding never mutated genes
Grey dots: never mutated genes, gittered
1e−06
1e−05
1e−04
1e−03
1e−02
1e−07
1e−05
1e−03
1e−01

E

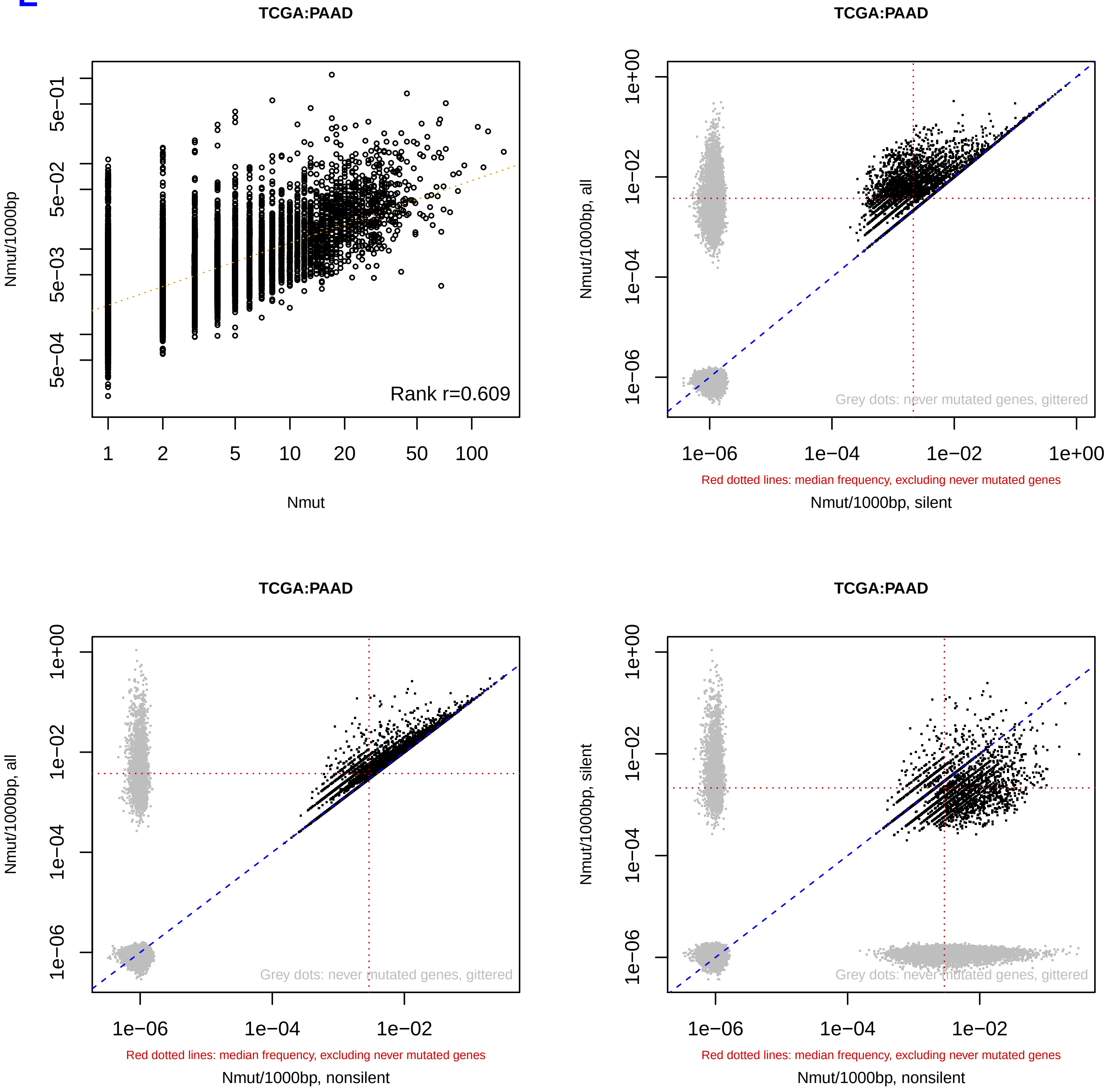

TCGA:PAAD
Nmut/1000bp
5e−01
5e−02
5e−03
5e−04
Rank r=0.609
1
2
5
10
20
50
100
Nmut
TCGA:PAAD
Nmut/1000bp, all
1e+00
1e−02
1e−04
1e−06
Grey dots: never mutated genes, gittered
1e−06
1e−04
1e−02
1e+00
Red dotted lines: median frequency, excluding never mutated genes
Nmut/1000bp, silent
TCGA:PAAD
Nmut/1000bp, all
1e+00
1e−02
1e−04
1e−06
Grey dots: never mutated genes, gittered
1e−06
1e−04
1e−02
Red dotted lines: median frequency, excluding never mutated genes
Nmut/1000bp, nonsilent
TCGA:PAAD
Nmut/1000bp, silent
1e+00
1e−02
1e−04
1e−06
Grey dots: never mutated genes, gittered
1e−06
1e−04
1e−02
Red dotted lines: median frequency, excluding never mutated genes
Nmut/1000bp, nonsilent

F

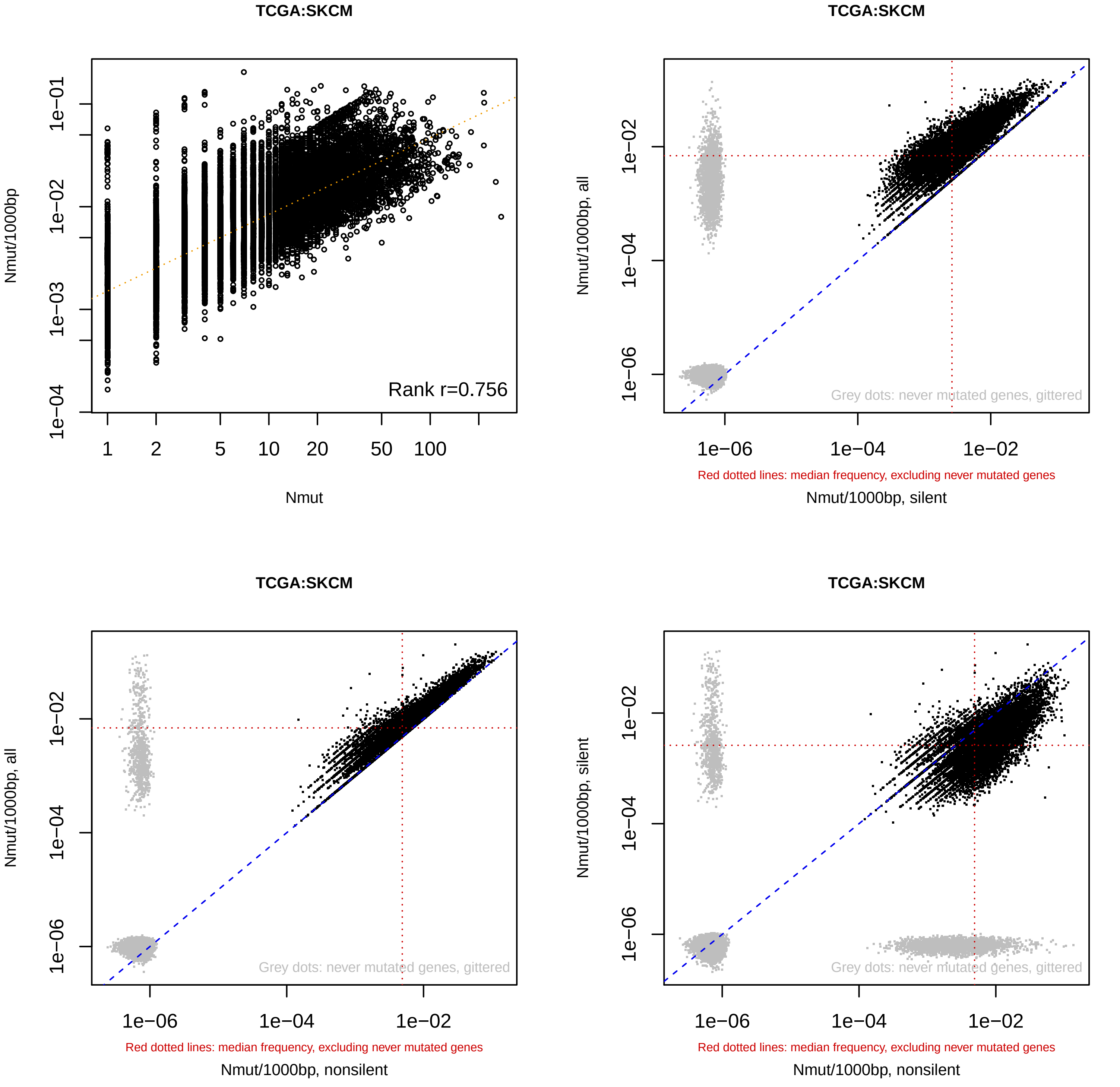

TCGA:SKCM
Rank r=0.756
Nmut/1000bp
Nmut
1e−01
1e−02
1e−03
1e−04
1
2
5
10
20
50
100
TCGA:SKCM
Nmut/1000bp, all
Nmut/1000bp, silent
1e−02
1e−04
1e−06
Grey dots: never mutated genes, gittered
Red dotted lines: median frequency, excluding never mutated genes
TCGA:SKCM
Nmut/1000bp, all
Nmut/1000bp, nonsilent
Grey dots: never mutated genes, gittered
Red dotted lines: median frequency, excluding never mutated genes
TCGA:SKCM
Nmut/1000bp, silent
Nmut/1000bp, nonsilent
Grey dots: never mutated genes, gittered
Red dotted lines: median frequency, excluding never mutated genes

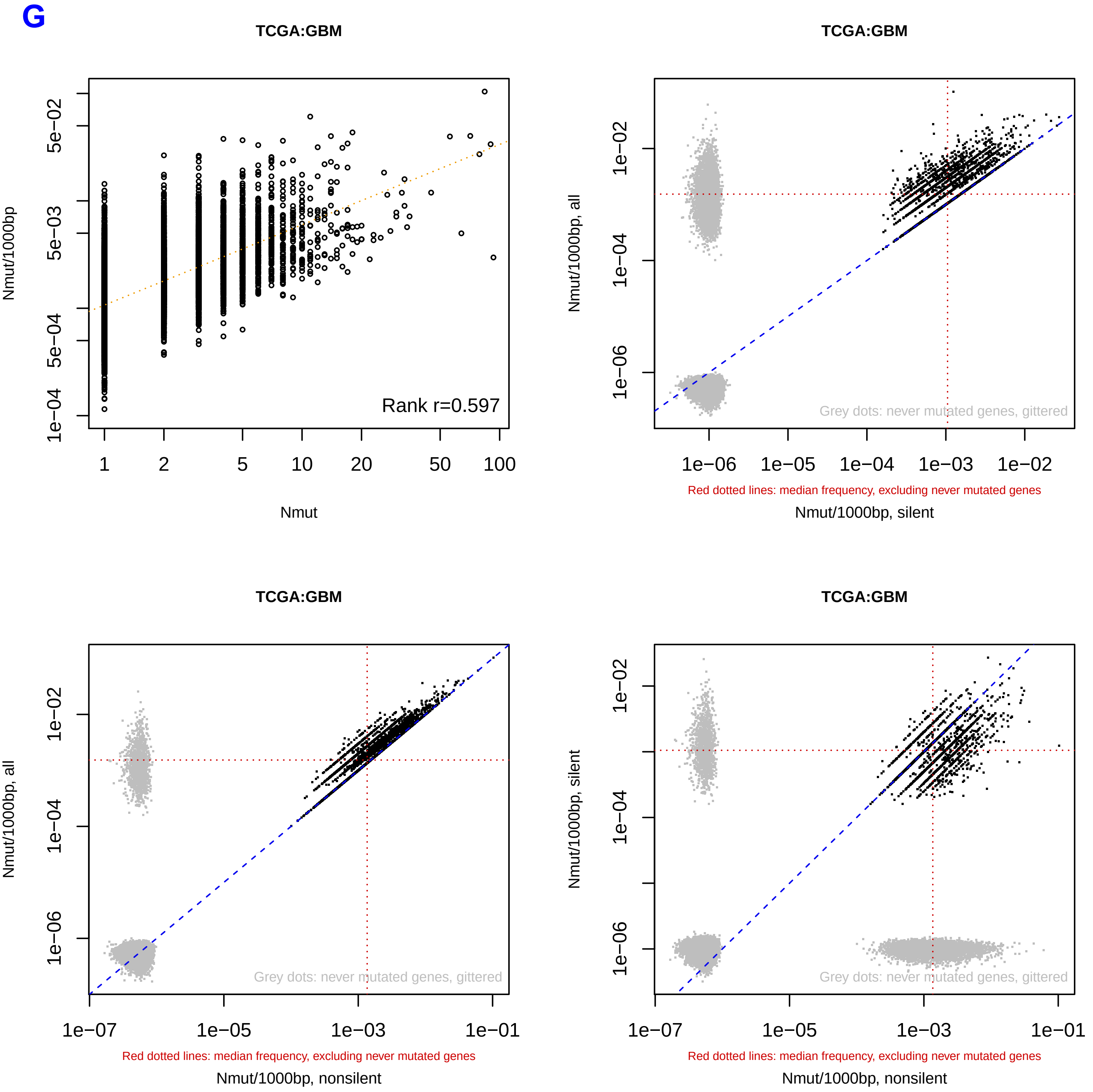
G
TCGA:GBM
Nmut/1000bp
5e−02
5e−03
5e−04
1e−04
Rank r=0.597
1
2
5
10
20
50
100
Nmut
TCGA:GBM
Nmut/1000bp, all
1e−02
1e−04
1e−06
Grey dots: never mutated genes, gittered
1e−06
1e−05
1e−04
1e−03
1e−02
Red dotted lines: median frequency, excluding never mutated genes
Nmut/1000bp, silent
TCGA:GBM
Nmut/1000bp, all
1e−02
1e−04
1e−06
Grey dots: never mutated genes, gittered
1e−07
1e−05
1e−03
1e−01
Red dotted lines: median frequency, excluding never mutated genes
Nmut/1000bp, nonsilent
TCGA:GBM
Nmut/1000bp, silent
1e−02
1e−04
1e−06
Grey dots: never mutated genes, gittered
1e−07
1e−05
1e−03
1e−01
Red dotted lines: median frequency, excluding never mutated genes
Nmut/1000bp, nonsilent

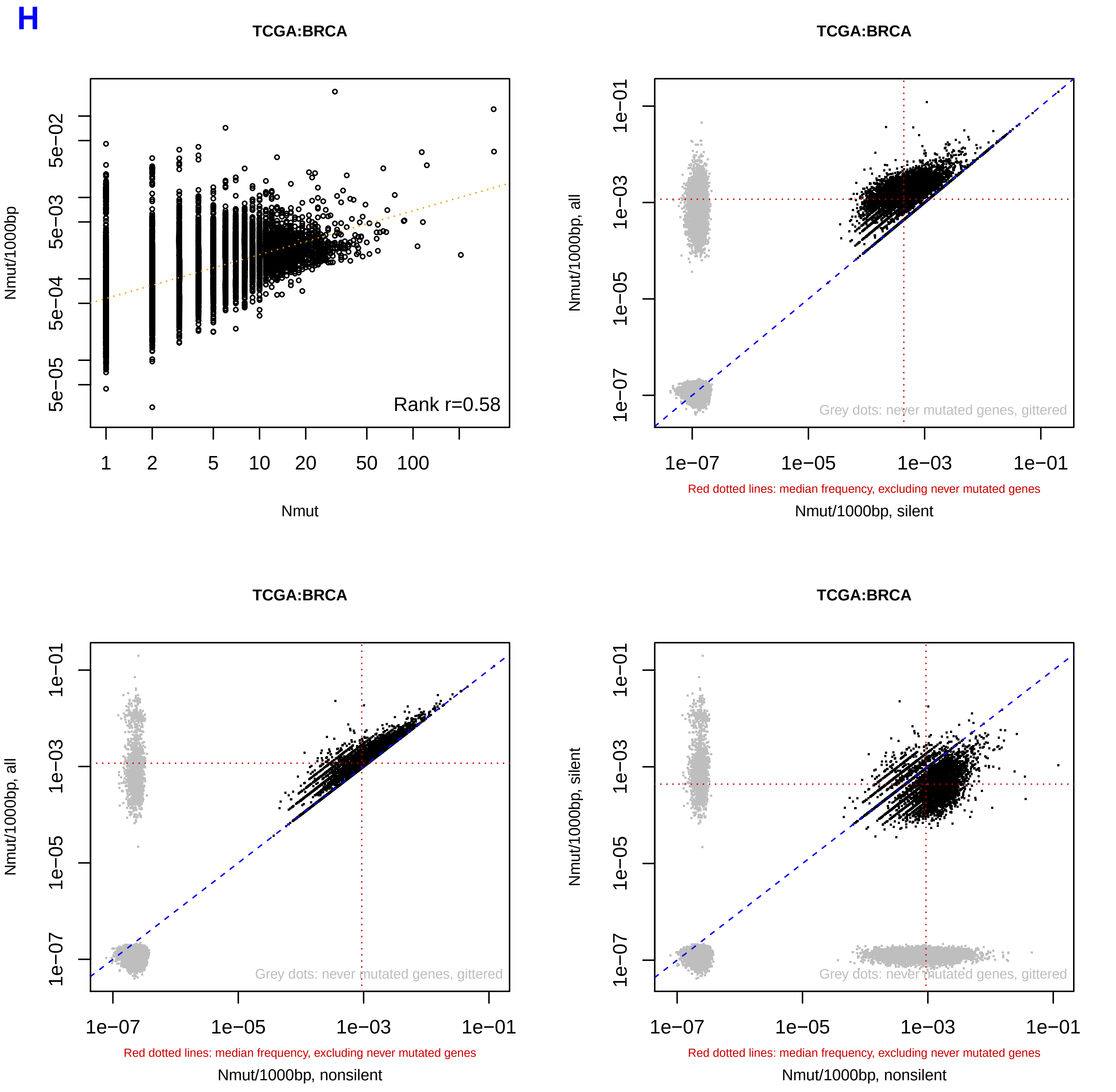

H
TCGA:BRCA
Rank r=0.58
Nmut
Nmut/1000bp
5e−05
5e−04
5e−03
5e−02
1
2
5
10
20
50
100
TCGA:BRCA
Grey dots: never mutated genes, gittered
Red dotted lines: median frequency, excluding never mutated genes
Nmut/1000bp, silent
Nmut/1000bp, all
1e−07
1e−05
1e−03
1e−01
TCGA:BRCA
Grey dots: never mutated genes, gittered
Red dotted lines: median frequency, excluding never mutated genes
Nmut/1000bp, nonsilent
Nmut/1000bp, all
TCGA:BRCA
Grey dots: never mutated genes, gittered
Red dotted lines: median frequency, excluding never mutated genes
Nmut/1000bp, nonsilent
Nmut/1000bp, silent

I

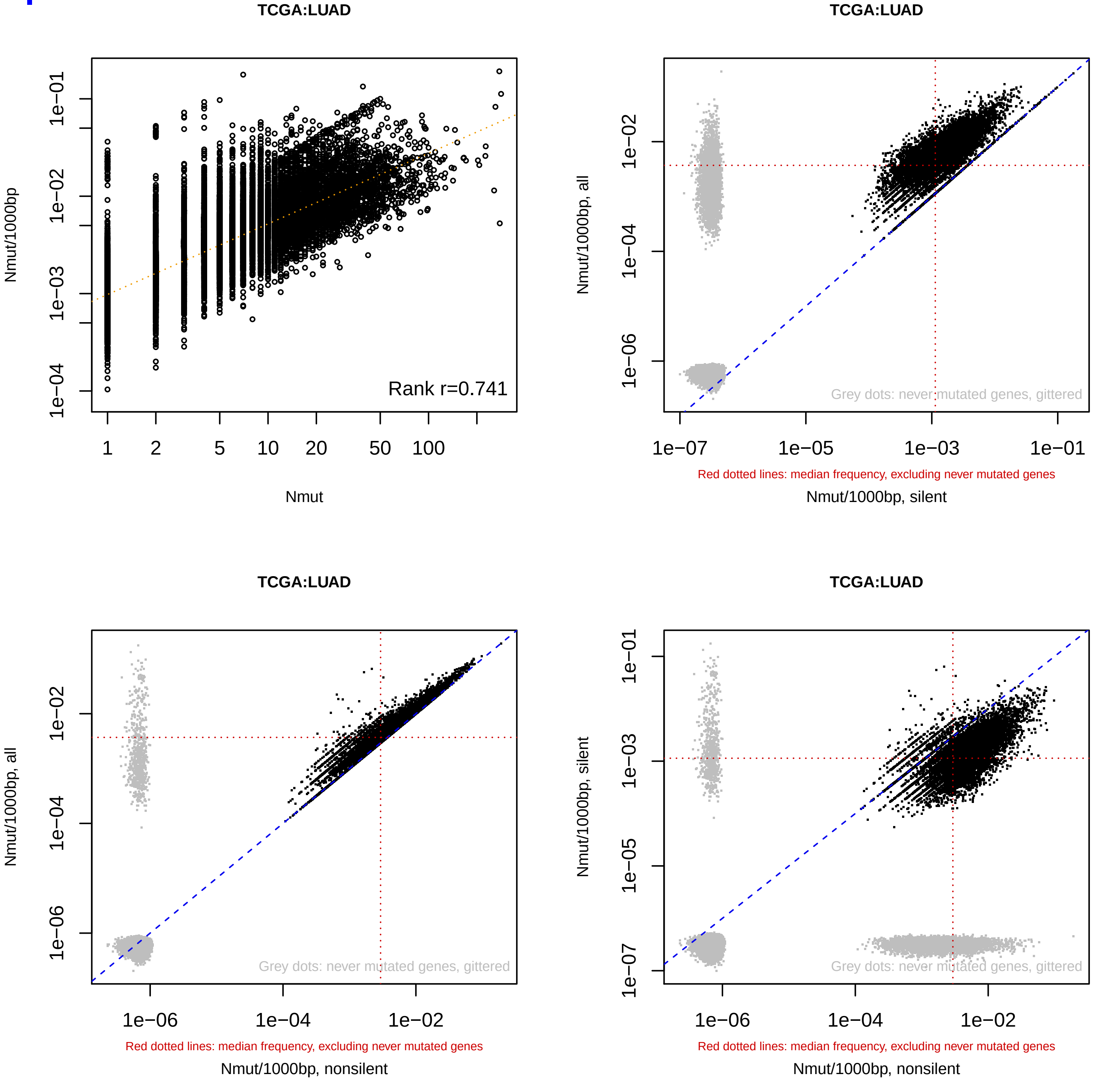

TCGA:LUAD
Nmut/1000bp
1e−01
1e−02
1e−03
1e−04
Rank r=0.741
1
2
5
10
20
50
100
Nmut
TCGA:LUAD
Nmut/1000bp, all
1e−02
1e−04
1e−06
Grey dots: never mutated genes, gittered
1e−07
1e−05
1e−03
1e−01
Red dotted lines: median frequency, excluding never mutated genes
Nmut/1000bp, silent
TCGA:LUAD
Nmut/1000bp, all
1e−02
1e−04
1e−06
Grey dots: never mutated genes, gittered
1e−06
1e−04
1e−02
Red dotted lines: median frequency, excluding never mutated genes
Nmut/1000bp, nonsilent
TCGA:LUAD
Nmut/1000bp, silent
1e−01
1e−03
1e−05
1e−07
Grey dots: never mutated genes, gittered
1e−06
1e−04
1e−02
Red dotted lines: median frequency, excluding never mutated genes
Nmut/1000bp, nonsilent

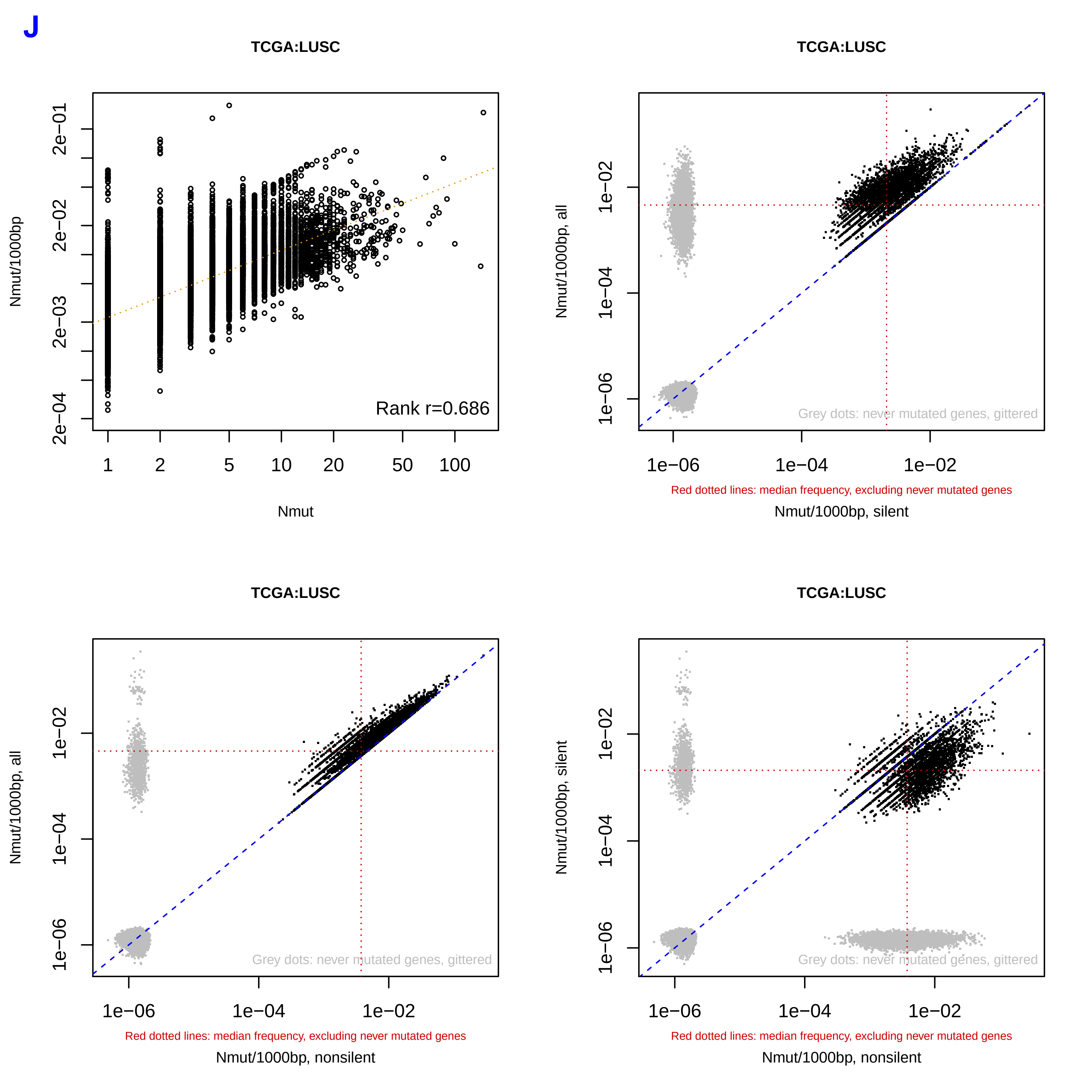
J
TCGA:LUSC
Nmut/1000bp
2e−01
2e−02
2e−03
2e−04
Rank r=0.686
1
2
5
10
20
50
100
Nmut
TCGA:LUSC
Nmut/1000bp, all
1e−02
1e−04
1e−06
Grey dots: never mutated genes, gittered
1e−06
1e−04
1e−02
Red dotted lines: median frequency, excluding never mutated genes
Nmut/1000bp, silent
TCGA:LUSC
Nmut/1000bp, all
1e−02
1e−04
1e−06
Grey dots: never mutated genes, gittered
1e−06
1e−04
1e−02
Red dotted lines: median frequency, excluding never mutated genes
Nmut/1000bp, nonsilent
TCGA:LUSC
Nmut/1000bp, silent
1e−02
1e−04
1e−06
Grey dots: never mutated genes, gittered
1e−06
1e−04
1e−02
Red dotted lines: median frequency, excluding never mutated genes
Nmut/1000bp, nonsilent

K

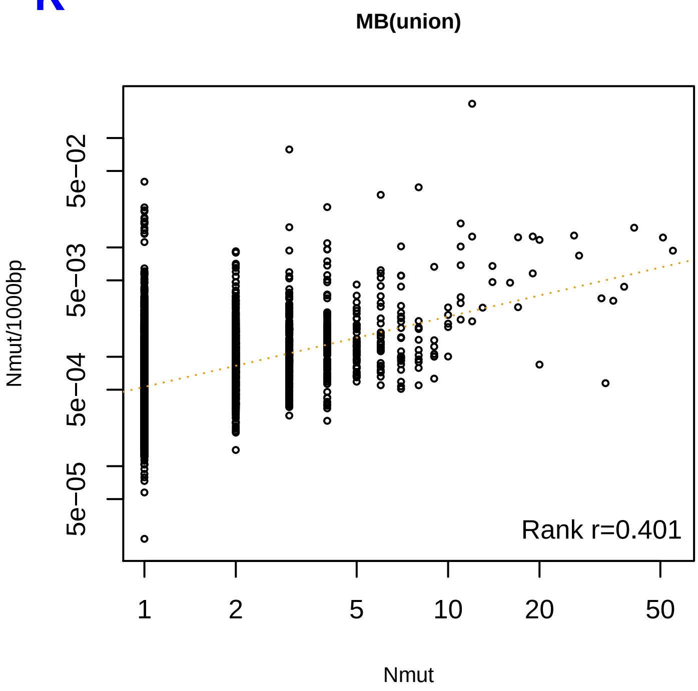
MB(union)
Nmut/1000bp
5e−05
5e−04
5e−03
5e−02
Rank r=0.401
1
2
5
10
20
50
Nmut
